# Supplementary material for: Oxygen mediated oxidative couplings of flavones in alkaline water
Source: Nat Commun. 2022 Oct 28;13:6424. doi: 10.1038/s41467-022-34123-w (PMC9614196; doi:10.1038/s41467-022-34123-w)
Supplement: Supplementary file 1 — Supplementary information [file 41467_2022_34123_MOESM1_ESM.pdf]

Supplementary Information for

Oxygen Mediated Oxidative Couplings of Flavones in Alkaline Water

Xin Yang[1], Sophie Hui Min Lim[1], Jiachen Lin[1], Jie Wu[2,3], Haidi Tang[2],[3] Fengyue Zhao[4], Fang Liu[4], Chenghua Sun[5], Xiangcheng Shi[2], Yulong Kuang[2], Joanne Yi Hui Toy[1], Ke Du[1], Yuannian Zhang[1], Xiang Wang[1], Mingtai Sun[1], Zhixuan Song[1], Tian Wang[2], Ji'en Wu[2], K. N. Houk[*6], Dejian Huang*[1,3]

Affiliations:

[1] Department of Food Science and Technology, National University of Singapore; 2 Science Drive 2, Singapore 117542, Republic of Singapore

[2] Department of Chemistry, National University of Singapore; 3 Science Drive 3, Singapore, 117543, Republic of Singapore

[3] National University of Singapore (Suzhou) Research Institute; 377 Linquan Street, Suzhou, Jiangsu 215123, China

[4] College of Sciences, Nanjing Agricultural University; Nanjing 210095, China

[5] Department of Chemistry and Biotechnology, Swinburne University of Technology; Hawthorn, Victoria 3122 Australia

[6] Department of Chemistry and Biochemistry, University of California; Los Angeles, CA 90095, USA

*Corresponding authors. Email: houk@chem.ucla.edu (K. N Houk) and dejian@nus.edu.sg (Dejian Huang)

Table of Contents

1. **Supplementary Notes** **3**
1.1 Yields and spectroscopic data of flavonoid dimers and trimers synthesized 3
1.2 Supplementary Figures 34
1.4 Supplementary Tables 116
1.5 X-Ray Crystallographic Data 119
1.6 ^{1}H and ^{13}C Spectra of products 146

1. Supplementary Notes

1.1 Yields and spectroscopic data of flavonoid dimers and trimers synthesized

Dicranolomin, 2a, 6-(6-(5,7-dihydroxy-4-oxo-4H-chromen-2-yl)-2,3-dihydroxyphenyl)-

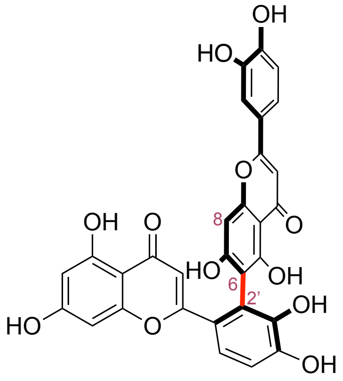

2-(3,4-dihydroxyphenyl)-5,7-dihydroxy-4H-chromen-4-one:

68 mg, 48% yield, brown solid. HPLC (Luna 5 µm C18(2) 100A, LC Column 250 × 4.6 mm, ACN/Water = 71.5: 28.5, flow rate 1.0 mL/min, λ = 300 nm), injection volume was 10 µL, *tr*(**2a**) = 19.045 min, concentration of **2a** = 1.0 mM. ^{1}H NMR (500 MHz, DMSO-d_6) δ 13.15 (s, 1H), 12.79 (s, 1H), 10.73 (s, 1H), 10.68 (s, 1H), 10.13 (s, 1H), 9.91 (s, 1H), 9.42 (s, 1H), 8.45 (s, 1H), 7.48 – 7.40 (m, 2H), 7.19 (d, *J* = 8.4 Hz, 1H), 6.95 (d, *J* = 8.4 Hz, 1H), 6.90 (d, *J* = 8.4 Hz, 1H), 6.70 (s, 1H), 6.54 (s, 1H), 6.09 (d, *J* = 2.1 Hz, 1H), 6.06 (s, 1H), 5.97 (d, *J* = 2.1 Hz, 1H). ^{13}C NMR (126 MHz, DMSO-d_6) δ 182.12, 181.76, 166.95, 164.45, 164.09, 161.76, 159.24, 157.85, 156.77, 150.12, 148.86, 146.20, 144.86, 124.19, 121.99, 120.70, 120.28, 119.44, 116.52, 114.70, 113.78, 108.53, 106.69, 103.86, 103.77, 103.33, 99.14, 93.79, 93.73. HRMS (ESI-TOF) calcd for $C_{30}H_{18}O_{12}$ = 569.0725, found 569.0717.

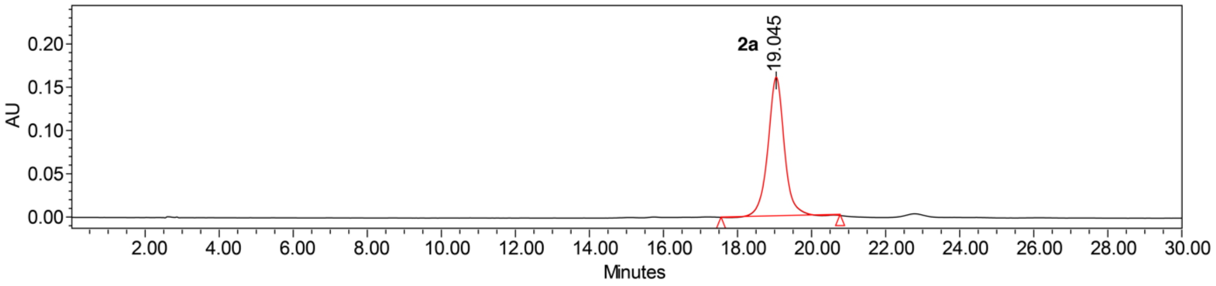

Philonotisflavone, 2a′, 8-(6-(5,7-dihydroxy-4-oxo-4*H*-chromen-2-yl)-2,3-dihydroxyphenyl)-2-(3,4-dihydroxyphenyl)-5,7-dihydroxy-4*H*-chromen-4-one:

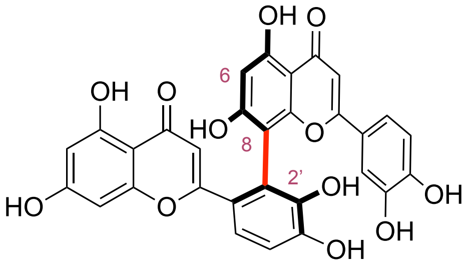

11 mg, 8% yield, brown solid. HPLC (Luna 5 μm C18(2) 100A, LC Column 250 × 4.6 mm, ACN/Water = 71.5: 28.5, flow rate 1.0 mL/min, λ = 300 nm), injection volume= 10 μL, *tr*(**2a′**) = 30.572 min, concentration of **2a′** = 6.5 mM. ^{1}H NMR (500 MHz, DMSO-d_6) δ 13.15 (s, 2H), 10.72 (s, 2H), 10.15 (s, 4H), δ 7.59 (d, J = 7.7 Hz, 2H), 7.20 (d, J = 8.3 Hz, 1H), 7.01 – 6.89 (m, 3H), 6.61 (s, 1H), 6.11 (d, J = 2.0 Hz, 1H), 6.05 (s, 1H), 5.99 (d, J = 2.0 Hz, 1H). ^{13}C NMR (75 MHz, DMSO-d_6) δ 182.24, 181.68, 166.78, 164.38, 163.88, 163.42, 162.15, 161.67, 159.14, 157.77, 156.72, 151.09, 148.70, 148.41, 144.63, 124.11, 121.90, 120.76, 120.66, 120.11, 116.15, 114.71, 110.57, 108.35, 106.67, 103.83, 103.76, 103.65, 99.07, 93.73. HRMS (ESI-TOF) calcd for $C_{30}H_{18}O_{12}$ = 569.0725, found 569.0715.

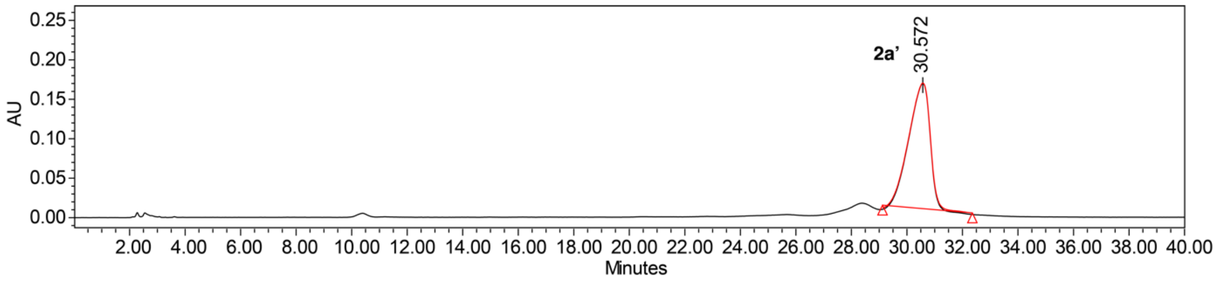

Dehydrohegoflavone B, 2a″, 6-(2-(5,7-dihydroxy-4-oxo-4*H*-chromen-2-yl)-4,5-dihydroxyphenyl)-2-(3,4-dihydroxyphenyl)-5,7-dihydroxy-4*H*-chromen-4-one:

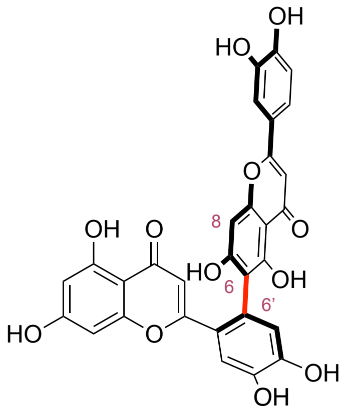

3 mg, 2% yield, brown solid. HPLC (Luna 5 μm C18(2) 100A, LC Column 250 × 4.6 mm, ACN/Water = 71.5: 28.5, flow rate 1.0 mL/min, λ = 300 nm), injection volume= 10 μL, *tr*(**2a″**) = 15.922 min, concentration of **2a″** = 3.14 mM. ^{1}H NMR (500 MHz, DMSO-d_6) δ

13.07 (s, 1H), 12.72 (s, 1H), 10.14 (s, 3H), 9.62 (s, 3H), 7.29 (s, 1H), 7.12 – 6.97 (m, 2H), 6.83 (s, 1H), 6.76 (d, *J* = 8.4 Hz, 1H), 6.61 (s, 1H), 6.31 (s, 1H), 6.08 (d, *J* = 2.1 Hz, 1H), 5.98 (s, 1H), 5.81 (d, *J* = 2.1 Hz, 1H). ^{13}C NMR (126 MHz, DMSO-d_6) δ 182.29, 181.61, 166.41, 164.57, 164.29, 161.68, 160.65, 157.69, 154.31, 150.28, 148.75, 146.12, 145.54, 123.66, 123.48, 123.34, 121.80, 120.38, 119.04, 116.34, 116.06, 114.03, 107.51, 106.65, 103.91, 103.72, 102.88, 99.21, 98.93, 93.67. HRMS (ESI-TOF) calcd for $C_{30}H_{18}O_{12}$ = 569.0725, found 569.0727.

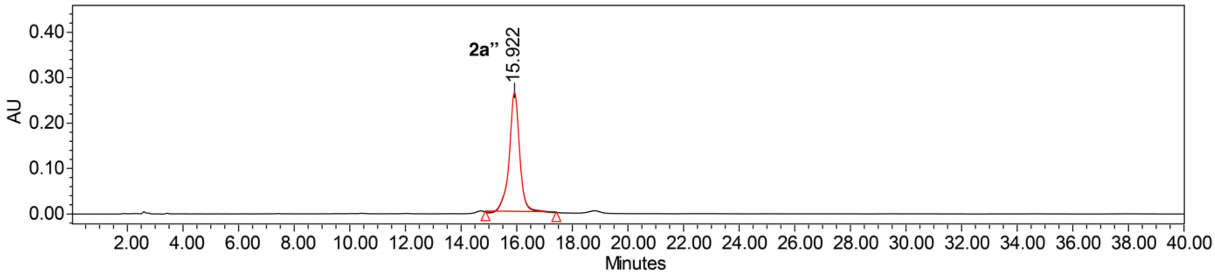

3′′′-Desoxydicranolomin, 2b, 6-(6-(5,7-dihydroxy-4-oxo-4H-chromen-2-yl)-2,3-dihydroxyphenyl)-5,7-dihydroxy-2-(4-hydroxyphenyl)-4H-chromen-4-one:

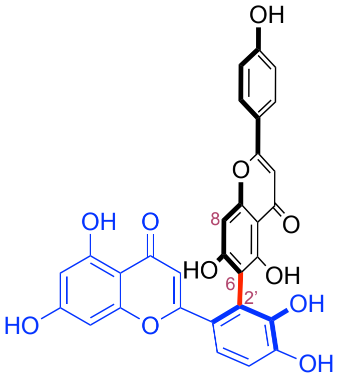

61 mg, 44% yield, brown solid. HPLC (Luna 5 μm C18(2) 100A, LC Column 250 × 4.6 mm, ACN/Water = 71.5: 28.5, flow rate 1.0 mL/min, λ = 300 nm), injection volume= 10 μL, *tr*(**2b**) = 30.447 min, concentration of **2b** = 1.66 mM. ^{1}H NMR (500 MHz, DMSO-d_6) δ 13.15 (s, 1H), 12.80 (s, 1H), 10.34 (s, 5H), 7.95 (d, *J* = 8.4 Hz, 2H), 7.17 (d, *J* = 8.4 Hz, 1H), 7.02 – 6.86 (m, 3H), 6.77 (s, 1H), 6.54 (s, 1H), 6.09 (d, *J* = 2.1 Hz, 1H), 6.03 (s, 1H), 5.98 (d, *J* = 2.1 Hz, 1H). ^{13}C NMR (126 MHz, DMSO-d_6) δ 182.13, 181.76, 167.11, 164.62, 164.43, 163.85, 161.75, 161.56, 159.22, 157.87, 156.86, 149.00, 145.11, 128.92, 128.92, 124.21, 121.72, 120.61, 120.47, 116.43, 116.43, 114.52, 108.77, 106.60, 103.85, 103.52, 103.26, 99.13, 94.09, 93.81. HRMS (ESI-TOF) calcd for $C_{30}H_{17}O_{11}$ = 553.0776, found 553.0782.

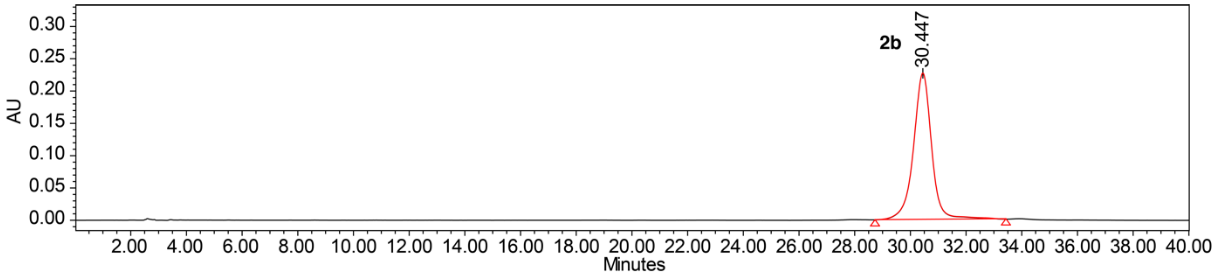

Lu-Ap (2′-8), 2b′, (Ap: apigenin), 8-(6-(5,7-dihydroxy-4-oxo-4*H*-chromen-2-yl)-2,3-dihydroxyphenyl)-5,7-dihydroxy-2-(4-hydroxyphenyl)-4*H*-chromen-4-one:

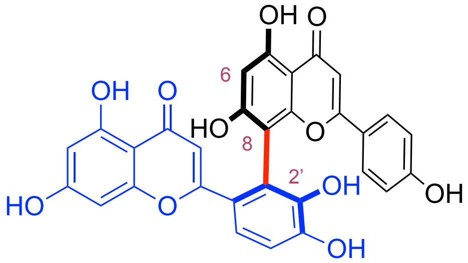

4 mg, 3% yield, brown solid. HPLC (Luna 5 µm C18(2) 100A, LC Column 250 × 4.6 mm, ACN/Water = 71.5: 28.5, flow rate 1.0 mL/min, λ = 300 nm), injection volume= 10 µL, *tr*(**2b′**) = 23.852 min, concentration of **2b′** = 8.3 mM. ^{1}H NMR (500 MHz, DMSO-d_6) δ 13.01 (s, 1H), 12.75 (s, 1H), 10.76 (s, 3H), 10.33 (s, 2H), 7.53 (d, J = 9.1 Hz, 2H), 7.33 – 7.22 (d, J = 8.2 Hz, 1H), 7.03 (d, J = 8.2 Hz, 1H), 6.81 (d, J = 9.1 Hz, 2H), 6.76 (s, 1H), 6.28 (d, J = 1.4 Hz, 1H), 6.08 (d, J = 2.1 Hz, 1H), 6.04 (d, J = 1.4 Hz, 1H), 5.76 (d, J = 2.1 Hz, 1H). ^{13}C NMR (126 MHz, DMSO-d_6) δ 207.07, 182.45, 181.71, 167.05, 164.43, 164.02, 162.11, 161.70, 161.51, 160.89, 157.71, 154.72, 148.99, 144.94, 128.59, 124.43, 121.73, 121.12, 119.26, 116.27, 115.08, 106.92, 104.00, 103.76, 103.58, 102.96, 99.17, 98.85, 93.63. HRMS (ESI-TOF) calcd for $C_{30}H_{17}O_{11}$ = 553.0776, found 553.0782.

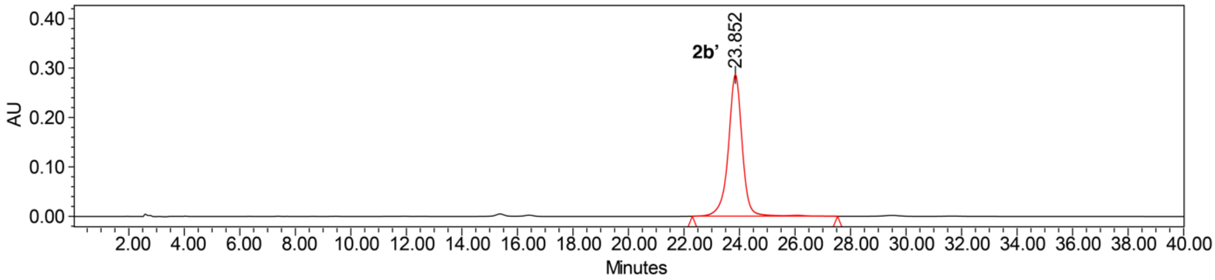

Lu-Dio (2′-6), 2c, (Dio: diosmetin), 6-(6-(5,7-dihydroxy-4-oxo-4H-chromen-2-yl)-2,3-dihydroxyphenyl)-5,7-dihydroxy-2-(3-hydroxy-4-methoxyphenyl)-4H-chromen-4-one:

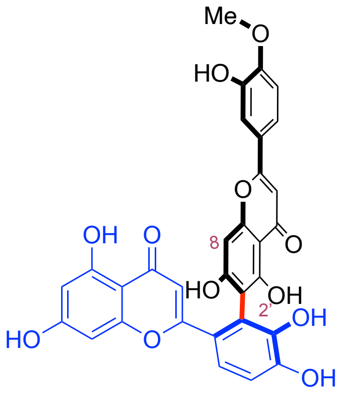

95 mg, 65% yield, brown solid. HPLC (Luna 5 µm C18(2) 100A, LC Column 250 × 4.6 mm, ACN/Water = 64: 36, flow rate 1.0 mL/min, λ = 300 nm), injection volume= 10 µL, *tr*(**2c**) = 11.018 min, concentration of **2c** = 0.81 mM. ^{1}H NMR (500 MHz, DMSO-d_6) δ 13.14 (s, 1H), 12.82 (s, 1H), 10.52 (s, 4H), 9.51 (s, 1H), 7.56 (dd, *J* = 8.7, 2.3 Hz, 1H), 7.45 (d, *J* = 2.4 Hz, 1H), 7.17 (d, *J* = 8.3 Hz, 1H), 7.10 (d, *J* = 8.7 Hz, 1H), 6.92 (d, *J* = 8.3 Hz, 1H), 6.73 (s, 1H), 6.51 (s, 1H), 6.09 (d, *J* = 2.1 Hz, 1H), 6.04 (s, 1H), 5.99 (d, *J* = 2.1 Hz, 1H), 3.88 (s, 3H). ^{13}C NMR (126 MHz, DMSO-d_6) δ 182.00, 181.77, 167.22, 164.43, 163.54, 161.75, 159.21, 157.87, 156.90, 151.52, 149.11, 147.27, 145.32, 124.21, 123.59, 120.57, 119.12, 114.42, 113.35, 112.68, 109.01, 106.52, 103.88, 103.85, 103.39, 99.12, 94.19, 93.81, 56.23. HRMS (ESI-TOF) calcd for $C_{31}H_{19}O_{12}$ = 583.0882, found 583.0898.

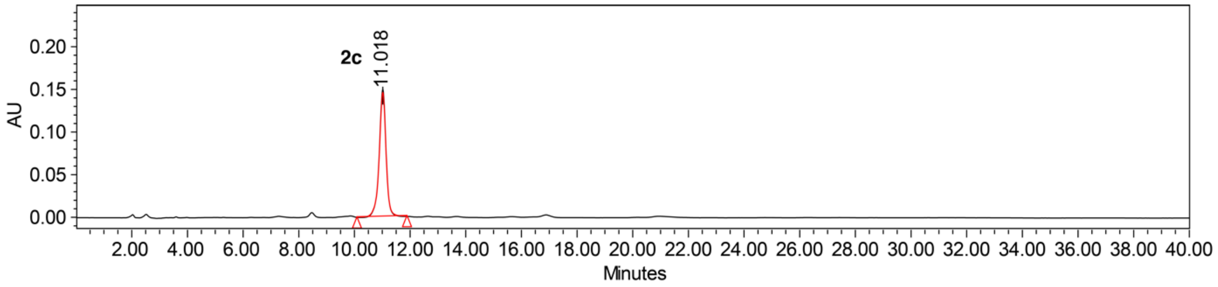

Lu-Chry (2′-6), 2d, (Chry: chrysin), 6-(6-(5,7-dihydroxy-4-oxo-4H-chromen-2-yl)-2,3-dihydroxyphenyl)-5,7-dihydroxy-2-phenyl-4H-chromen-4-one:

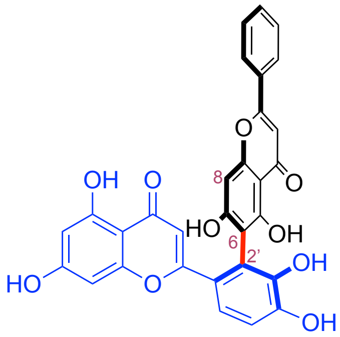

62 mg, 46% yield, brown solid. HPLC (Luna 5 μm C18(2) 100A, LC Column 250 × 4.6 mm, ACN/Water = 60: 40, flow rate 1.0 mL/min, λ = 300 nm), injection volume= 10 μL, *tr*(**2d**) = 12.096 min, concentration of **2d** = 2.40 mM. ^{1}H NMR (400 MHz, DMSO-d_6) δ 13.01 (s, 1H), 12.79 (s, 1H), 11.14 – 10.09 (m, 4H), δ 8.16 – 8.05 (m, 2H), 7.68 – 7.53 (m, 3H), 7.20 (d, J = 8.4 Hz, 1H), 7.00 (s, 1H), 6.95 (d, J = 8.4 Hz, 1H), 6.62 (s, 1H), 6.09 (d, J = 2.1 Hz, 1H), 6.07 (s, 1H), 5.98 (d, J = 2.1 Hz, 1H). ^{13}C NMR (126 MHz, DMSO-d_6) δ 182.38, 181.76, 166.90, 164.47, 163.46, 162.78, 161.77, 159.23, 157.85, 156.96, 148.84, 144.79, 132.47, 131.20, 129.63, 129.63, 126.91, 126.91, 124.19, 120.75, 120.13, 114.79, 108.75, 106.74, 105.68, 104.13, 103.86, 99.16, 93.97, 93.80. HRMS (ESI-TOF) calcd for $C_{30}H_{17}O_{10}$ = 537.0827, found 537.0824.

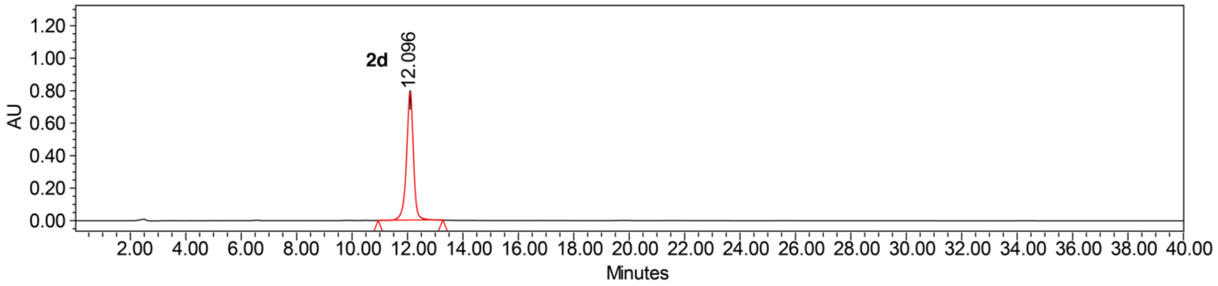

Lu-Wo (2′-6), 2e, (Wo: wogonin), 6-(6-(5,7-dihydroxy-4-oxo-4H-chromen-2-yl)-2,3-dihydroxyphenyl)-5,7-dihydroxy-8-methoxy-2-phenyl-4H-chromen-4-one:

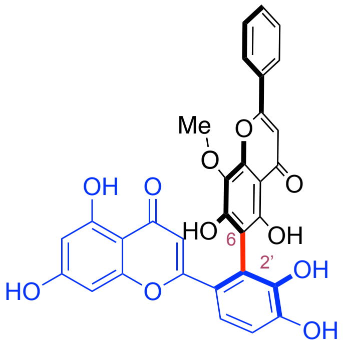

121 mg, 85% yield, brown solid. HPLC (Luna 5 µm C18(2) 100A, LC Column 250 × 4.6 mm, ACN/Water = 60: 40, flow rate 1.0 mL/min, λ = 300 nm), injection volume= 10 µL, *tr*(**2e**) = 25.651 min, concentration of **2e** = 1.95 mM. ^{1}H NMR (500 MHz, DMSO-d_6) δ 12.80 (s, 1H), 12.73 (s, 1H), 10.73 (s, 1H), 10.29 (s, 1H), 10.18 (s, 1H), 8.53 (s, 1H), δ 8.18 – 8.05 (m, 2H), 7.68 – 7.55 (m, 3H), 7.22 (d, *J* = 8.4 Hz, 1H), 7.06 (s, 1H), 6.97 (d, *J* = 8.4 Hz, 1H), 6.09 (d, *J* = 2.1 Hz, 1H), 6.08 (s, 1H), 5.91 (d, *J* = 2.1 Hz, 1H), 3.82 (s, 3H). ^{13}C NMR (126 MHz, DMSO-d_6) δ 182.56, 181.77, 166.86, 164.44, 163.29, 161.76, 157.78, 155.48, 154.52, 149.05, 148.86, 144.75, 132.57, 131.28, 129.77, 127.91, 126.76, 124.07, 120.78, 119.76, 114.90, 108.89, 106.79, 105.69, 103.85, 103.82, 99.14, 93.75, 61.95. HRMS (ESI-TOF) calcd for $C_{31}H_{19}O_{11}$ = 567.0933, found 567.0921.

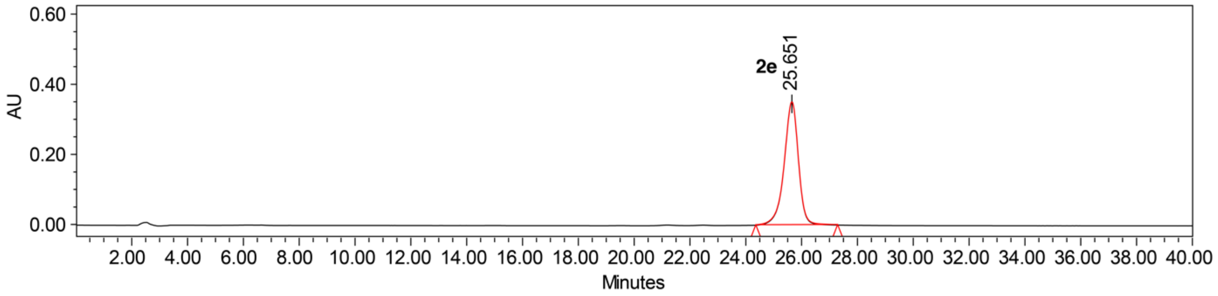

Lu-56 (2′-7), 2f, (56: 5,6-dihydroxyflavone), 7-(6-(5,7-dihydroxy-4-oxo-4*H*-chromen-2-yl)-2,3-dihydroxyphenyl)-5,6-dihydroxy-2-phenyl-4*H*-chromen-4-one:

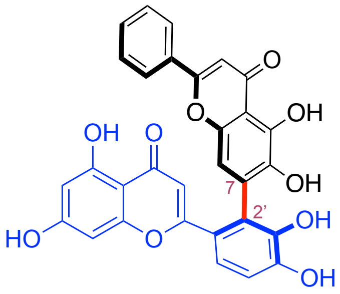

77 mg, 57% yield, brown solid. HPLC (Luna 5 µm C18(2) 100A, LC Column 250 × 4.6 mm, ACN/Water = 64: 36, flow rate 1.0 mL/min, λ = 300 nm), injection volume= 10 µL, *tr*(**2f**) = 19.290 min, concentration of **2f** = 1.95 mM. ^{1}H NMR (500 MHz, DMSO-d_6) δ 13.25 (s, 1H), 12.84 (s, 1H), 11.04 (s, 1H), 9.95 (s, 1H), 9.58 (s, 1H), 9.12 (s, 1H), 7.68 – 7.57 (m, 2H), 7.52 – 7.44 (m, 1H), 7.44 – 7.29 (m, 3H), 7.04 (s, 1H), 6.96 – 6.84 (m, 2H), 6.71 (s, 1H), 6.66 (d, *J* = 8.9 Hz, 1H), 6.50 (s, 1H). ^{13}C NMR (126 MHz, DMSO-d_6) δ 184.38, 182.45, 164.32, 164.10, 162.30, 161.40, 154.81, 150.11, 146.87, 146.39, 146.10, 141.03, 132.58, 131.18, 129.52, 126.42, 126.37, 121.82, 118.75, 116.04, 113.72, 111.16, 110.67, 104.86,

104.21, 103.06, 102.12, 99.19. HRMS (ESI-TOF) calcd for $C_{30}H_{17}O_{10}$ = 537.0827, found 537.0837.

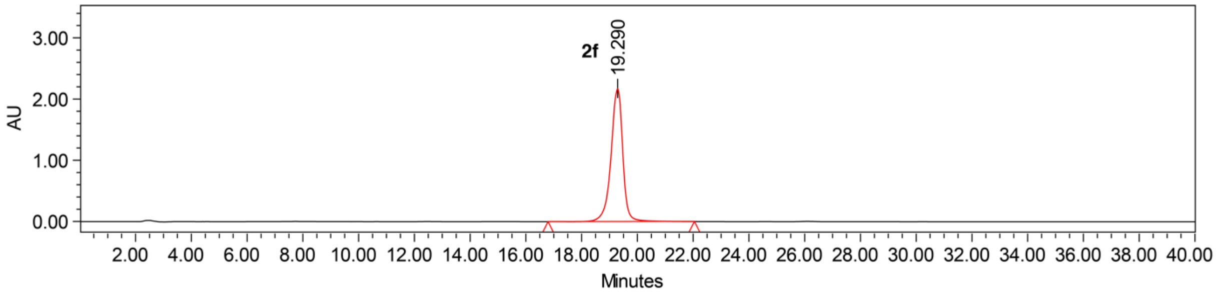

Lu-56 (2′-8), 2f′, (56: 5,6-dihydroxyflavone), 8-(6-(5,7-dihydroxy-4-oxo-4*H*-chromen-2-yl)-2,3-dihydroxyphenyl)-5,6-dihydroxy-2-phenyl-4*H*-chromen-4-one:

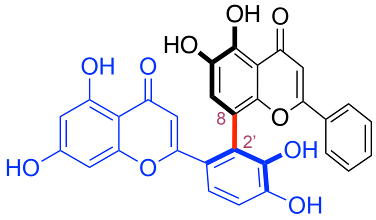

8 mg, 6% yield, brown solid. HPLC (Luna 5 µm C18(2) 100A, LC Column 250 × 4.6 mm, ACN/Water = 64: 36, flow rate 1.0 mL/min, λ = 300 nm), injection volume = 10 µL, *tr*(**2f′**) = 25.948 min, concentration of **2f′** = 2.50 mM. ^{1}H NMR (500 MHz, DMSO-d_6) δ 13.52 (s, 1H), 12.73 (s, 1H), 9.51 (s, 4H), 7.82 (d, J = 8.2 Hz, 2H), 7.53 (d, J = 7.2 Hz, 1H), 7.48 (q, J = 8.2, 7.2 Hz, 4H), 7.25 (s, 1H), 7.05 (s, 1H), 6.93 (s, 1H), 6.77 (s, 1H), 6.73 (s, 1H). ^{13}C NMR (126 MHz, DMSO-d_6) δ 184.37, 182.28, 164.42, 164.23, 162.54, 159.59, 157.07, 150.30, 146.54, 146.27, 140.78, 132.59, 131.42, 129.66, 126.71, 126.36, 121.89, 119.59, 116.56, 113.87, 111.51, 111.05, 106.90, 104.85, 103.99, 103.38, 93.96. HRMS (ESI-TOF) calcd for $C_{30}H_{17}O_{10}$ = 537.0827, found 537.0830.

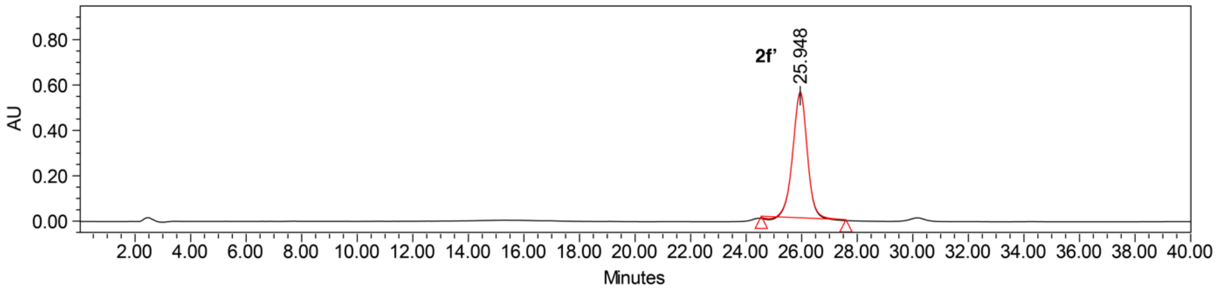

Lu-Ge (2′-6), 2g, (Ge: genistein), 6-(6-(5,7-dihydroxy-4-oxo-4H-chromen-2-yl)-2,3-dihydroxyphenyl) -5,7-dihydroxy-3-(4-hydroxyphenyl)-4H-chromen-4-one:

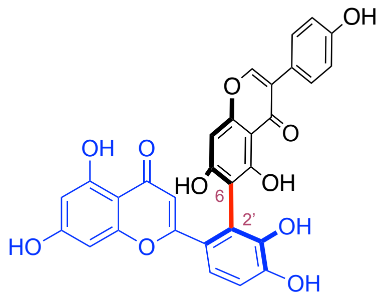

39 mg, 28 % yield, brown solid. HPLC (Luna 5 μm C18(2) 100A, LC Column 250 × 4.6 mm, ACN/Water = 71.5: 28.5, flow rate 1.0 mL/min, λ = 300 nm), injection volume= 10 μL, *tr*(**2g**) = 28.867 min, concentration of **2g** = 1.13 mM. ^{1}H NMR (500 MHz, DMSO-d_6) δ 13.13 (s, 1H), 12.78 (s, 1H), 10.73 (s, 3H), 10.15 (s, 1H), 9.58 (s, 2H), 8.34 (s, 1H), 7.42 – 7.34 (m, 2H), 7.18 (d, *J* = 8.4 Hz, 1H), 6.94 (d, *J* = 8.4 Hz, 1H), 6.86 – 6.75 (m, 2H), 6.48 (s, 1H), 6.10 (d, *J* = 2.1 Hz, 1H), 6.03 (s, 1H), 5.99 (d, *J* = 2.1 Hz, 1H). ^{13}C NMR (126 MHz, DMSO-d_6) δ 181.75, 180.73, 166.83, 164.48, 162.64, 161.77, 159.68, 157.85, 157.85, 157.07, 154.28, 148.81, 144.75, 130.67, 130.67, 124.19, 122.75, 121.74, 120.74, 120.19, 115.50, 115.50, 114.77, 108.59, 106.75, 104.62, 103.87, 99.16, 93.82, 93.47. HRMS (ESI-TOF) calcd for $C_{30}H_{17}O_{11}$ =553.0776, found 553.0781.

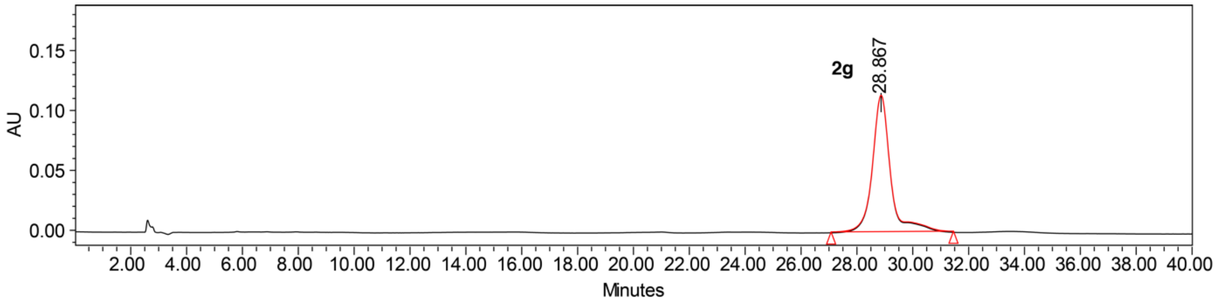

Lu-Ge (2′-8), 2g′, (Ge: genistein), 8-(6-(5,7-dihydroxy-4-oxo-4H-chromen-2-yl)-2,3-dihydroxyphenyl)-5,7-dihydroxy-3-(4-hydroxyphenyl)-4H-chromen-4-one:

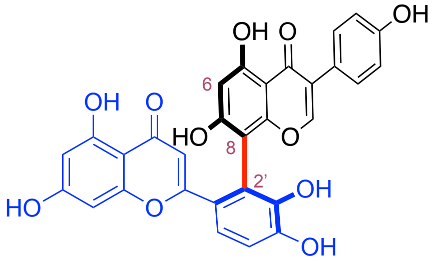

19 mg, 14% yield, brown solid. HPLC (Luna 5 μm C18(2) 100A, LC Column 250 × 4.6 mm, ACN/Water = 71.5: 28.5, flow rate 1.0 mL/min, λ = 300 nm), injection volume= 10 μL,

tr(**2g′**) = 29.696 min, concentration of **2g′** = 0.51 mM. ^{1}H NMR (500 MHz, DMSO-d_6) δ 13.08 (s, 1H), 12.80 (s, 1H), 10.70 (s, 3H), 9.59 (s, 2H), 8.30 (s, 1H), 7.38 – 7.32 (m, 2H), 7.21 (d, *J* = 8.4 Hz, 1H), 6.97 (d, *J* = 8.4 Hz, 1H), 6.84 – 6.77 (m, 2H), 6.30 (s, 1H), 6.11 (d, *J* = 2.1 Hz, 1H), 6.08 (s, 1H), 5.85 (d, *J* = 2.1 Hz, 1H). ^{13}C NMR (126 MHz, DMSO-d_6) δ 181.78, 180.87, 166.98, 164.49, 162.41, 161.77, 161.45, 157.84, 157.77, 155.50, 154.50, 149.08, 145.12, 130.57, 130.57, 124.42, 122.46, 121.66, 121.10, 119.26, 115.51, 115.51, 115.00, 106.92, 104.78, 103.83, 103.48, 99.18, 99.12, 93.74. HRMS (ESI-TOF) calcd for $C_{30}H_{17}O_{11}$ = 553.0776, found 553.0776.

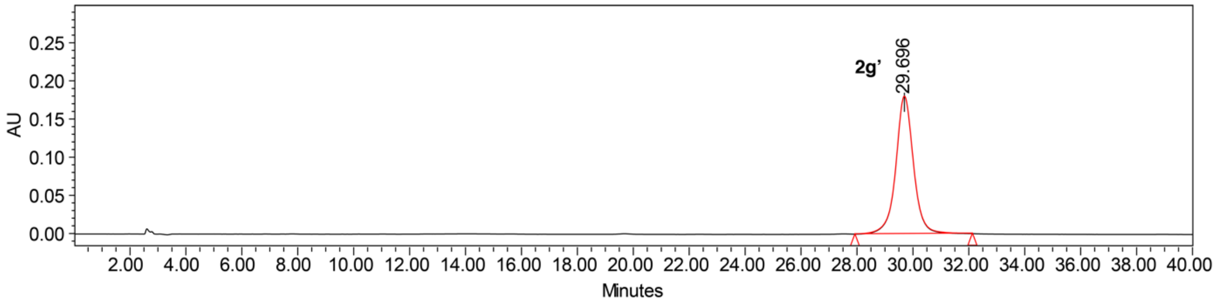

534-Lu (2′-6), 2h, (534: 5,3′,4′-trihydroxyflavone), 6-(2,3-dihydroxy-6-(5-hydroxy-4-oxo-4*H*-chromen-2-yl)phenyl)-2-(3,4-dihydroxyphenyl)-5,7-dihydroxy-4*H*-chromen-4-one:

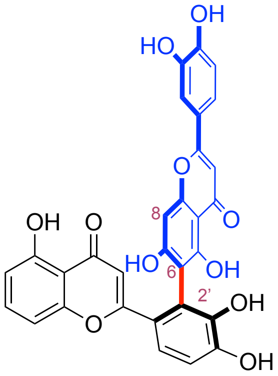

60 mg, 43% yield, brown solid. HPLC (Luna 5 μm C18(2) 100A, LC Column 250 × 4.6 mm, ACN/Water = 64: 36, flow rate 1.0 mL/min, λ = 300 nm), injection volume= 10 μL, *tr*(**2h**) = 14.195 min, concentration of **2h** = 4.29 mM. ^{1}H NMR (500 MHz, DMSO-d_6) δ 13.16 (s, 1H), 12.64 (s, 1H), 9.76 (s, 5H), 7.53 (t, *J* = 8.2 Hz, 1H), 7.48 – 7.40 (m, 2H), 7.25 (d, *J* = 8.4 Hz, 1H), 6.97 (d, *J* = 8.4 Hz, 1H), 6.91 (d, *J* = 8.2 Hz, 1H), 6.74 – 6.67 (m, 2H), 6.65 (d, *J* = 8.4, 1H), 6.56 (s, 1H), 6.20 (s, 1H). ^{13}C NMR (126 MHz, DMSO-d_6) δ 182.95, 182.16, 167.91, 164.19, 162.43, 160.24, 159.28, 156.83, 156.36, 150.19, 149.14, 146.22, 144.86, 136.16, 124.01, 121.94, 121.01, 120.36, 119.49, 116.54, 114.84, 113.78, 111.12, 110.09, 108.40, 107.34, 107.18, 103.87, 103.33, 93.71. HRMS (ESI-TOF) calcd for $C_{30}H_{19}O_{11}$ = 553.0776, found 553.0735.

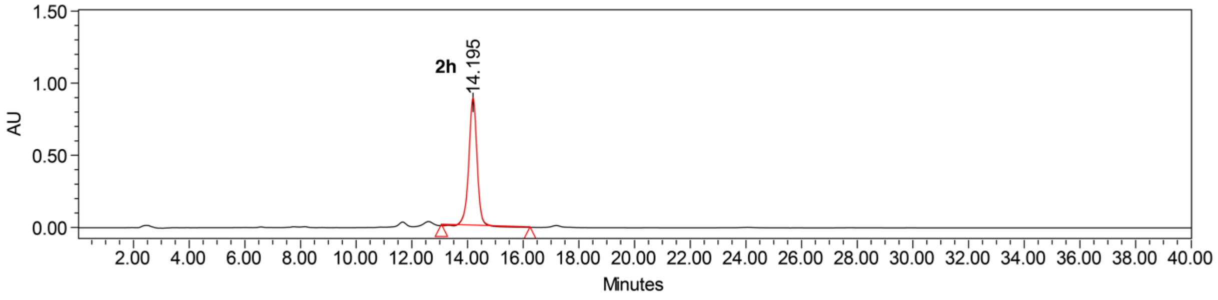

634-Lu (2′-6), 2i, (634: 6,3′,4′-trihydroxyflavone**),** 6-(2,3-dihydroxy-6-(6-hydroxy-4-oxo-4*H*-chromen-2-yl)phenyl)-5,7-dihydroxy-2-(4-hydroxyphenyl)-4*H*-chromen-4-one:

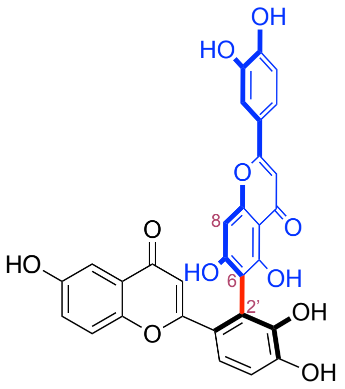

51 mg, 37% yield, brown solid. HPLC (Luna 5 µm C18(2) 100A, LC Column 250 × 4.6 mm, ACN/Water = 72.5: 27.5, flow rate 1.0 mL/min, λ = 300 nm), injection volume= 10 µL, *tr*(**2i**) = 9.532 min, concentration of **2i** = 6.99 mM. ^{1}H NMR (500 MHz, DMSO-d_6) δ 13.12 (s, 1H), 10.70 (s, 1H), 10.06 (s, 1H), 9.92 (s, 1H), 9.45 (s, 1H), 8.43 (s, 1H), 8.14 (s, 1H), 7.50 – 7.39 (m, 2H), 7.26 – 7.08 (m, 4H), 6.96 (d, J = 8.4 Hz, 1H), 6.91 (d, J = 8.2 Hz, 1H), 6.69 (s, 1H), 6.54 (s, 1H), 6.05 (s, 1H). ^{13}C NMR (126 MHz, DMSO-d_6) δ 182.16, 176.92, 165.67, 164.16, 163.51, 162.40, 159.31, 156.74, 155.05, 150.15, 149.90, 148.35, 146.21, 144.69, 125.02, 124.29, 123.24, 121.97, 120.42, 120.15, 119.55, 116.53, 114.77, 113.79, 108.57, 108.07, 107.81, 103.87, 103.33, 93.62. HRMS (ESI-TOF) calcd for $C_{30}H_{19}O_{11}$ = 553.0776, found 553.0769.

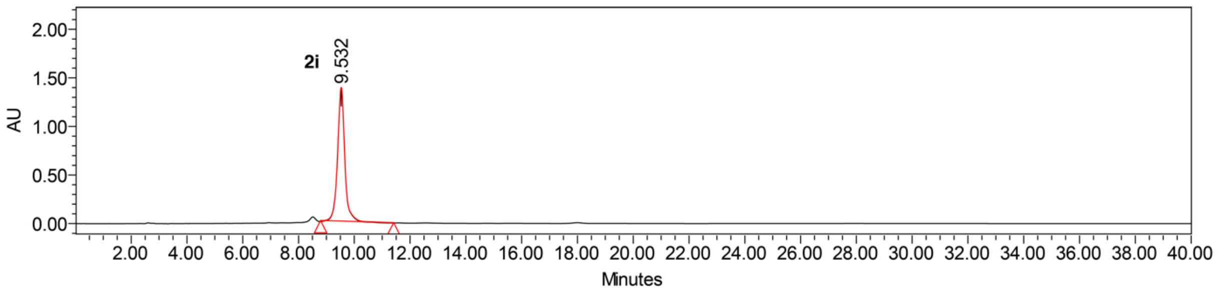

734-Lu (2′-6), 2j, (734: 7,3′,4′-trihydroxyflavone), 6-(2,3-dihydroxy-6-(7-hydroxy-4-oxo-4*H*-chromen-2-yl)phenyl)-2-(3,4-dihydroxyphenyl)-5,7-dihydroxy-4*H*-chromen-4-one:

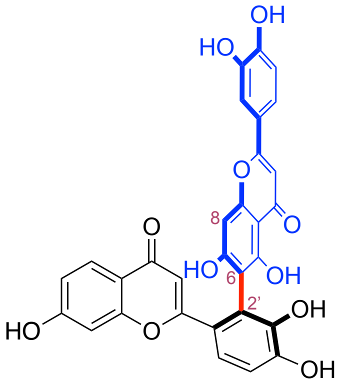

59 mg, 43% yield, brown solid. HPLC (Luna 5 µm C18(2) 100A, LC Column 250 × 4.6 mm, ACN/Water = 72.5: 27.5, flow rate 1.0 mL/min, λ = 300 nm), injection volume= 10 µL, *tr*(**2j**) = 9.790 min, concentration of **2j** = 9.25 mM. ^{1}H NMR (500 MHz, DMSO-d_6) δ 13.14 (s, 1H), 10.69 (d, *J* = 8.2 Hz, 2H), 10.06 (s, 1H), 9.94 (s, 1H), 9.46 (s, 1H), 8.42 (s, 1H), 7.76 (d, *J* = 8.6 Hz, 1H), 7.45 (d, *J* = 8.6 Hz, 2H), 7.18 (d, *J* = 8.3 Hz, 1H), 6.96 (d, *J* = 8.3 Hz, 1H), 6.92 (d, *J* = 8.1 Hz, 1H), 6.82 (dd, *J* = 8.1, 2.1 Hz, 1H), 6.70 (s, 1H), 6.55 (s, 1H), 6.50 (d, *J* = 2.1 Hz, 1H), 6.03 (s, 1H). ^{13}C NMR (126 MHz, DMSO-d_6) δ 207.17, 182.21, 176.60, 165.55, 164.17, 162.85, 162.35, 159.29, 157.97, 156.76, 150.15, 148.31, 146.22, 144.67, 126.83, 124.93, 122.02, 120.47, 120.11, 119.49, 116.56, 116.22, 115.22, 114.80, 113.78, 108.63, 108.59, 103.90, 103.37, 102.36, 93.64. HRMS (ESI-TOF) calcd for $C_{30}H_{19}O_{11}$ = 553.0776, found 555.0732.

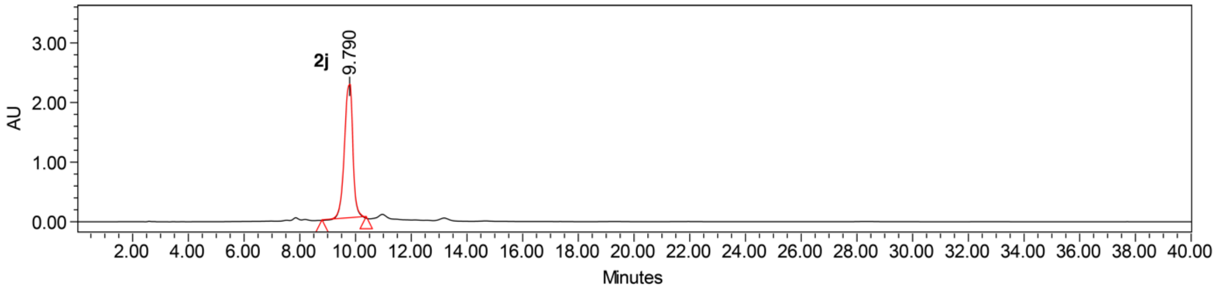

34-Lu (2′-6), 2k, (34: 3′,4′-dihydroxyflavone), 6-(2,3-dihydroxy-6-(4-oxo-4*H*-chromen-2-yl)phenyl)-2-(3,4-dihydroxyphenyl)-5,7-dihydroxy-4*H*-chromen-4-one:

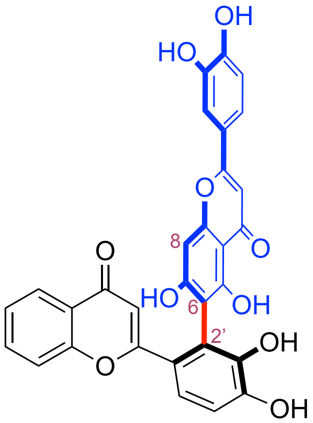

67 mg, 50% yield, brown solid. HPLC (Luna 5 µm C18(2) 100A, LC column 250 × 4.6 mm, ACN/Water = 71.5: 28.5, flow rate 1.0 mL/min, λ = 300 nm), injection volume= 10 µL, *tr*(**2k**) = 18.289 min, concentration of **2k** = 3.25 mM. ^{1}H NMR (400 MHz, DMSO-d_6) δ 13.14 (s, 1H), 9.69 (s, 5H), 7.92 (d, *J* = 7.9 Hz, 1H), 7.68 (d, *J* = 7.9 Hz, 1H), 7.47 – 7.35 (m, 3H), 7.25 (dd, *J* = 16.9, 8.3 Hz, 2H), 6.97 (d, *J* = 8.3 Hz, 1H), 6.90 (d, *J* = 8.3 Hz, 1H), 6.68 (s, 1H), 6.54 (s, 1H), 6.13 (s, 1H). ^{13}C NMR (126 MHz, DMSO-d_6) δ 182.14, 177.05, 166.20, 164.14, 162.64, 159.31, 156.78, 156.17, 150.17, 148.62, 146.22, 144.82, 134.46, 125.66, 125.15, 124.73, 123.41, 121.95, 120.57, 120.28, 119.48, 118.23, 116.53, 114.75, 113.77, 108.98, 108.61, 103.80, 103.30, 93.71. HRMS (ESI-TOF) calcd for $C_{30}H_{17}O_{10}$ = 537.0827, found 537.0818.

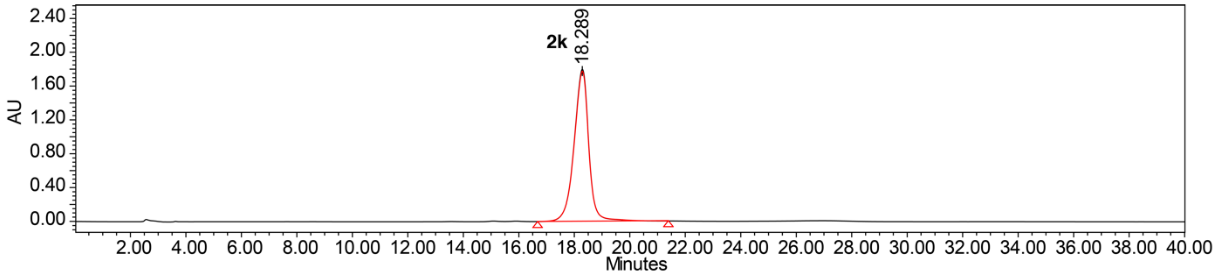

534-Ap (2′-6), 2l, 6-(2,3-dihydroxy-6-(5-hydroxy-4-oxo-4*H*-chromen-2-yl)phenyl)-5,7-dihydroxy-2-(4-hydroxyphenyl)-4*H*-chromen-4-one:

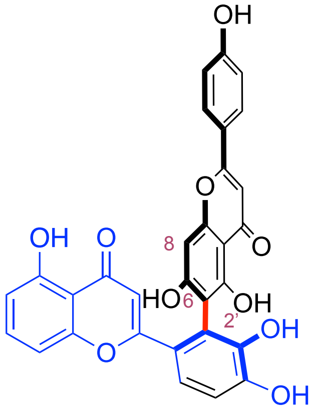

73 mg, 54% yield, brown solid. HPLC (Luna 5 µm C18(2) 100A, LC column 250 × 4.6 mm, ACN/Water = 64: 36, flow rate 1.0 mL/min, λ = 300 nm), injection volume= 10 µL, *tr*(**2l**) = 21.513 min, concentration of **2l** = 15.80 mM. ^{1}H NMR (500 MHz, DMSO-d_6) δ 13.19 (s, 1H), 12.63 (s, 1H), 10.81 (s, 1H), 10.40 (s, 1H), 10.26 (s, 1H), 8.55 (s, 1H), 7.94 (d, *J* = 8.0 Hz, 2H), 7.49 (d, *J* = 10.1 Hz, 1H), 7.27 (d, *J* = 8.0 Hz, 1H), 6.98 (m, 3H), 6.80 (s, 1H), 6.73 – 6.55 (m, 3H), 6.21 (s, 1H). ^{13}C NMR (126 MHz, DMSO-d_6) δ 182.94, 182.28, 167.85, 164.11, 162.35, 161.66, 160.25, 159.31, 156.88, 156.37, 149.11, 144.85, 136.08, 128.96, 124.09, 121.62, 121.03, 120.38, 116.48, 114.90, 111.09, 110.10, 108.39, 107.41, 107.18, 103.96, 103.32, 93.85. HRMS (ESI-TOF) calcd for $C_{30}H_{17}O_{10}$ = 537.0827, found 537.0821.

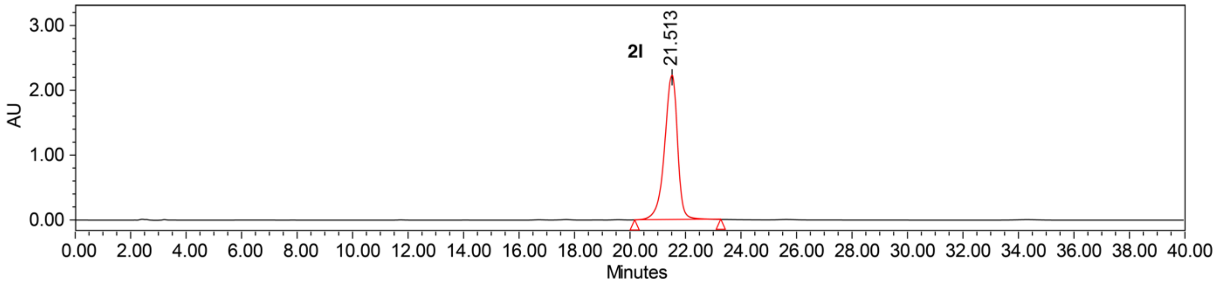

634-Ap (2′-6), 2m, 6-(2,3-dihydroxy-6-(6-hydroxy-4-oxo-4*H*-chromen-2-yl)phenyl)-5,7-dihydroxy-2-(4-hydroxyphenyl)-4*H*-chromen-4-one:

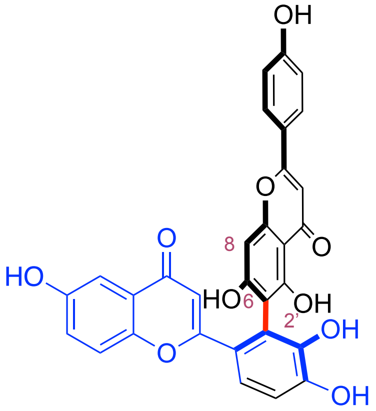

82 mg, 61% yield, brown solid. HPLC (Luna 5 µm C18(2) 100A, LC column 250 × 4.6 mm, ACN/Water = 72.5: 27.5, flow rate 1.0 mL/min, λ = 300 nm), injection volume= 10 µL, *tr*(**2m**) = 14.506 min, concentration of **2m** = 13.71 mM. ^{1}H NMR (500 MHz, DMSO-d_6) δ 13.14 (s, 1H), 9.99 (s, 5H), 7.95 (d, *J* = 8.2 Hz, 2H), 7.25 – 7.09 (m, 4H), 6.97 (dd, *J* = 12.2, 8.2 Hz, 3H), 6.79 (s, 1H), 6.60 (s, 1H), 6.08 (s, 1H). ^{13}C NMR (126 MHz, DMSO-d_6) δ 182.29, 177.02, 165.76, 164.10, 162.51, 161.66, 159.34, 156.84, 155.09, 149.96, 148.42, 144.75, 129.00, 125.09, 124.32, 123.31, 121.69, 120.49, 120.23, 119.60, 116.51, 114.83, 108.65, 108.13, 107.86, 103.92, 103.35, 93.83. HRMS (ESI-TOF) calcd for $C_{30}H_{17}O_{10}$ = 537.0827, found 537.0821.

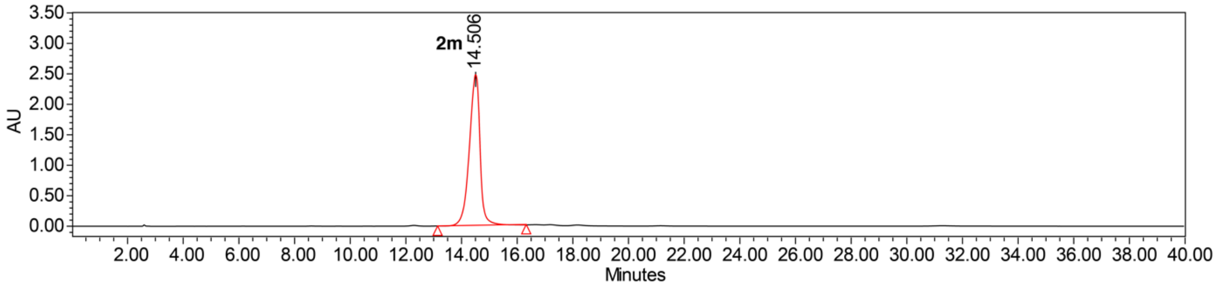

634-Ap (2′-8), 2m′, 8-(2,3-dihydroxy-6-(6-hydroxy-4-oxo-4*H*-chromen-2-yl)phenyl)-5,7-dihydroxy-2-(4-hydroxyphenyl)-4*H*-chromen-4-one:

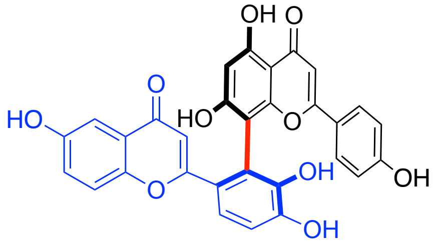

4 mg, 3% yield, brown solid. HPLC (Luna 5 µm C18(2) 100A, LC column 250 × 4.6 mm, ACN/Water = 72.5: 27.5, flow rate 1.0 mL/min, λ = 300 nm), injection volume= 10 µL,

tr(**2m′**) = 12.177 min, concentration of **2m′** = 2.32 mM. ^{1}H NMR (500 MHz, DMSO-d_6) δ 12.98 (s, 1H), 9.94 (s, 5H), 7.52 (d, *J* = 8.5 Hz, 2H), 7.25 (d, *J* = 8.2 Hz, 1H), 7.16 (d, *J* = 3.0 Hz, 1H), 7.10 – 7.01 (m, 2H), 6.87 (d, *J* = 8.2 Hz, 1H), 6.79 (d, *J* = 8.5 Hz, 2H), 6.71 (s, 1H), 6.26 (s, 1H), 5.99 (s, 1H). ^{13}C NMR (126 MHz, DMSO-d_6) δ 182.38, 176.80, 165.90, 163.89, 161.52, 160.85, 155.03, 154.73, 149.67, 148.73, 145.16, 128.61, 125.16, 124.17, 123.18, 121.69, 120.64, 119.44, 119.23, 116.23, 114.96, 108.09, 107.79, 103.94, 103.79, 102.83, 99.07. HRMS (ESI-TOF) calcd for $C_{30}H_{17}O_{10}$ = 537.0827, found 537.0818.

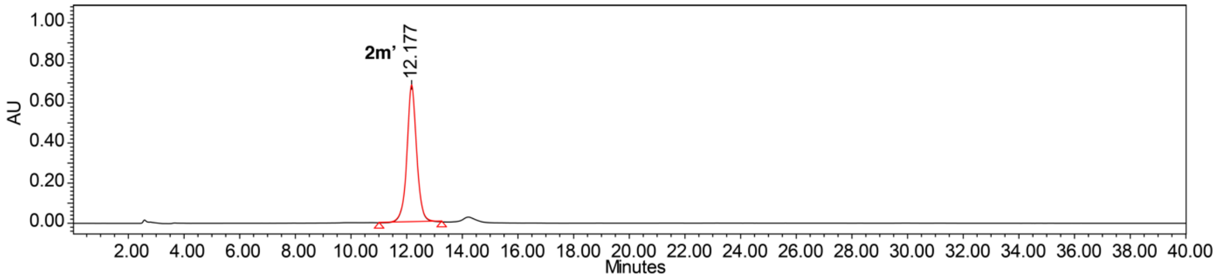

734-Ap (2′-6), 2n, 6-(2,3-dihydroxy-6-(7-hydroxy-4-oxo-4*H*-chromen-2-yl)phenyl)-5,7-dihydroxy-2-(4-hydroxyphenyl) -4*H*-chromen-4-one:

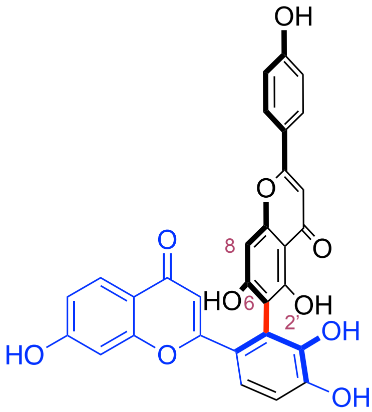

65 mg, 48% yield, brown solid. HPLC (Luna 5 µm C18(2) 100A, LC column 250 × 4.6 mm, ACN/Water = 72.5: 27.5, flow rate 1.0 mL/min, λ = 300 nm), injection volume= 10 µL, *tr*(**2n**) = 14.399 min, concentration of **2n** = 22.53 mM. ^{1}H NMR (500 MHz, DMSO-d_6) δ 13.13 (s, 1H), 10.32 (s, 5H), 7.96 (d, *J* = 8.5 Hz, 2H), 7.75 (d, *J* = 8.5 Hz, 1H), 7.18 (d, *J* = 8.2 Hz, 1H), 7.03 – 6.87 (m, 3H), 6.82 (d, *J* = 8.2 Hz, 2H), 6.58 (s, 1H), 6.50 (s, 1H), 6.02 (s, 1H). ^{13}C NMR (126 MHz, DMSO-d_6) δ 182.28, 176.51, 165.49, 164.02, 162.85, 162.41, 161.62, 159.28, 157.97, 156.77, 148.31, 144.68, 128.97, 126.80, 124.92, 121.68, 120.41, 120.13, 116.46, 116.22, 115.19, 114.74, 108.65, 108.59, 103.88, 103.34, 102.37, 93.76. HRMS (ESI-TOF) calcd for $C_{30}H_{17}O_{10}$ = 537.0827, found 537.0824.

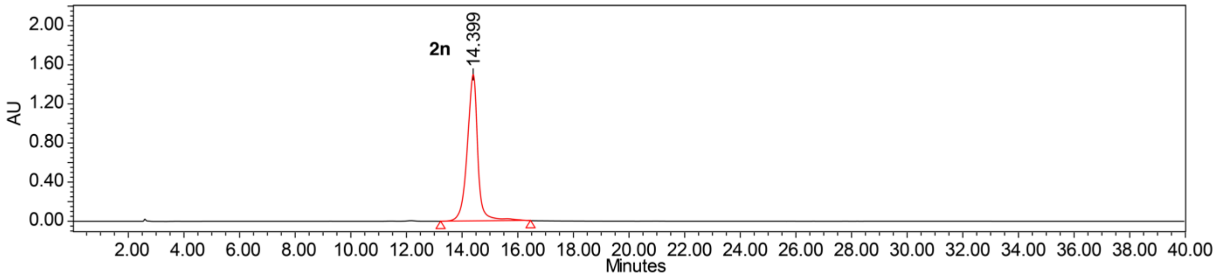

734-Ap (2′-8), 2n′, 8-(2,3-dihydroxy-6-(7-hydroxy-4-oxo-4*H*-chromen-2-yl)phenyl)-5,7-dihydroxy-2-(4-hydroxyphenyl)-4*H*-chromen-4-one:

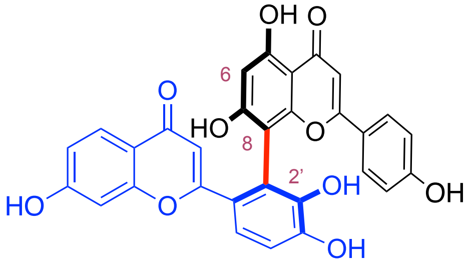

4 mg, 3% yield, brown solid. HPLC (Luna 5 µm C18(2) 100A, LC column 250 × 4.6 mm, ACN/Water = 72.5: 27.5, flow rate 1.0 mL/min, λ = 300 nm), injection volume= 10 µL, *tr*(**2n′**) = 12.139 min, concentration of **2n′** = 3.34 mM. ^{1}H NMR (500 MHz, DMSO-d_6) δ 12.98 (s, 1H), 10.42 (s, 5H), 7.70 (d, J = 8.5 Hz, 1H), 7.52 (d, J = 8.5 Hz, 2H), 7.23 (d, J = 8.3 Hz, 1H), 7.02 (d, J = 8.3 Hz, 1H), 6.78 (d, J = 8.5 Hz, 3H), 6.72 (s, 1H), 6.26 (d, J = 8.5 Hz, 1H), 6.25 (s, 1H), 5.95 (s, 1H). ^{13}C NMR (126 MHz, DMSO-d_6) δ 182.38, 176.36, 165.70, 163.88, 162.78, 161.50, 160.81, 157.78, 154.71, 148.63, 145.08, 128.59, 126.76, 125.13, 121.71, 120.66, 119.37, 116.22, 116.08, 115.16, 114.93, 108.68, 103.93, 103.80, 102.85, 102.13, 99.03. HRMS (ESI-TOF) calcd for $C_{30}H_{17}O_{10}$ = 537.0827, found 537.0822.

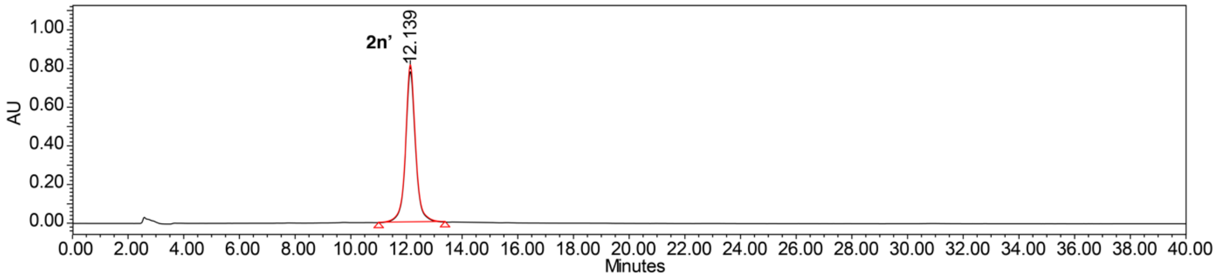

34-Ap (2′-6), 2o, 6-(2,3-dihydroxy-6-(4-oxo-4*H*-chromen-2-yl)phenyl)-5,7-dihydroxy-2-(4-hydroxyphenyl)-4*H*-chromen-4-one:

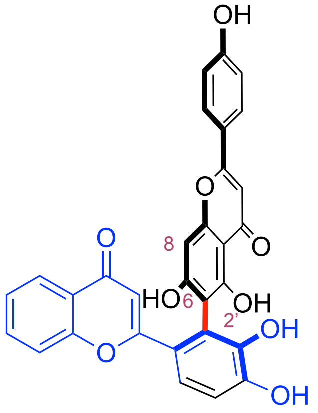

60 mg, 46% yield, brown solid. HPLC (Luna 5 μm C18(2) 100A, LC column 250 × 4.6 mm, ACN/Water = 71.5: 28.5, flow rate 1.0 mL/min, λ = 300 nm), injection volume= 10 μL, *tr*(**2o**) = 31.427 min, concentration of **2o** = 10.06 mM. ^{1}H NMR (500 MHz, DMSO-d_6) δ 13.16 (s, 1H), 10.36 (s, 4H), 7.93 (t, *J* = 8.6 Hz, 3H), 7.68 (t, *J* = 8.0 Hz, 1H), 7.42 – 7.32 (m, 1H), 7.32 – 7.18 (m, 2H), 6.99 (d, *J* = 8.3 Hz, 1H), 6.95 (d, *J* = 8.3 Hz, 2H), 6.79 (d, *J* = 3.7 Hz, 1H), 6.60 (d, *J* = 3.7 Hz, 1H), 6.15 (d, *J* = 3.7 Hz, 1H). ^{13}C NMR (126 MHz, DMSO-d_6) δ 182.26, 177.04, 166.14, 164.06, 162.44, 161.65, 159.33, 156.81, 156.18, 148.58, 144.76, 134.42, 128.97, 125.63, 125.14, 124.78, 123.41, 121.64, 120.59, 120.27, 118.24, 116.46, 114.83, 109.04, 108.56, 103.91, 103.32, 93.81. HRMS (ESI-TOF) calcd for $C_{30}H_{17}O_9$ = 521.0827, found 521.0871.

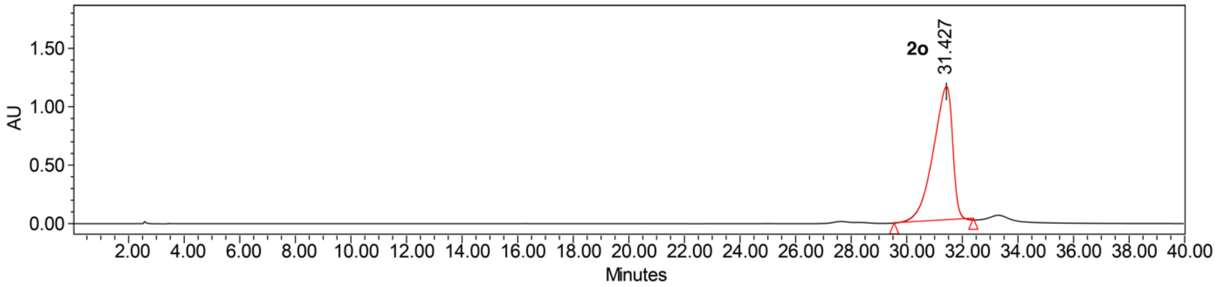

Distichumtriluteolin, **3a**, 6-(6-(5,7-dihydroxy-4-oxo-4*H*-chromen-2-yl)-2,3-dihydroxyphenyl)-2-(2-(2-(3,4-dihydroxyphenyl)-5,7-dihydroxy-4-oxo-4*H*-chromen-6-yl)-3,4-dihydroxyphenyl)-5,7-dihydroxy-4*H*-chromen-4-one:

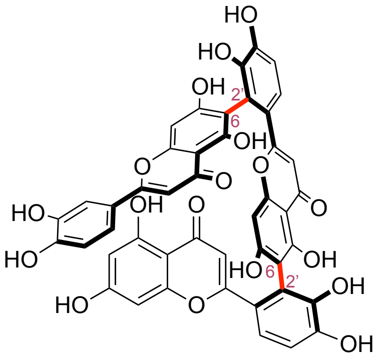

90 mg, 42% yield, brown solid. HPLC (Luna 5 µm C18(2) 100A, LC Column 250 × 4.6 mm, ACN/Water = 71.5: 28.5, flow rate 1.0 mL/min, λ = 300 nm), injection volume= 10 µL, *tr*(**3a**) = 22.742 min, concentration of **3a** = 10 mM. **3a** = **3a*** + **3a****. Atropisomer **3a***: ^{1}H NMR (500 MHz, Methanol-d_4) δ 7.41 (m, 2H), 7.27 (d, *J* = 8.4 Hz, 1H), 7.19 (d, *J* = 8.3 Hz, 1H), 7.00 (d, *J* = 8.3 Hz, 1H), 6.95 (d, *J* = 8.3 Hz, 1H), 6.59 (s, 1H), 6.58 (s, 1H), 6.57 (s, 1H), 6.23 (s, 1H), 6.17 (s, 1H), 6.13 (d, 1H), 6.04 (d, 1H), 6.01 (s, 1H). Atropisomer **3a****: ^{1}H NMR (500 MHz, Methanol-d_4) δ 7.41 (m, 2H), 7.27 (d, *J* = 8.4 Hz, 1H), 7.19 (dd, *J* = 8.3 Hz, 1H), 7.00 (d, *J* = 8.3 Hz, 1H), 6.95 (d, *J* = 8.3 Hz, 1H), 6.59 (s, 1H), 6.58 (s, 1H), 6.57 (s, 1H), 6.19 (s, 1H), 6.09 (s, 1H), 6.13 (d, 1H), 6.04 (d, 1H), 6.01 (s, 1H). ^{13}C NMR (126 MHz, DMSO-d_6) δ 182.21, 181.81, 181.72, 166.66, 166.56, 164.15, 164.09, 162.28, 161.75, 159.07, 157.87, 157.06, 156.81, 150.04, 148.72, 146.17, 144.64, 124.14, 122.09, 120.63, 120.29, 119.43, 116.50, 114.75, 113.86, 108.37, 108.29, 106.66, 103.89, 103.75, 103.64, 103.43, 99.27, 93.96, 93.87, 93.73. HRMS (ESI-TOF) calcd for $C_{45}H_{27}O_{18}$ = 855.1192, found 855.1182.

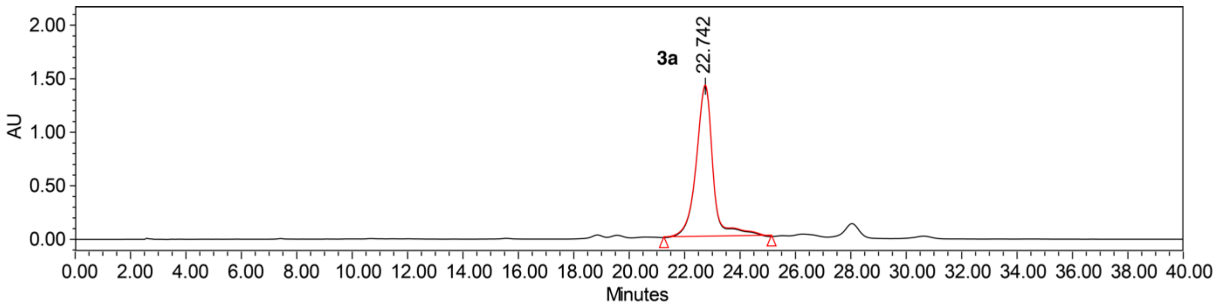

Lu_2-Ap (2′-6), 3b, 2-(2-(5,7-dihydroxy-2-(4-hydroxyphenyl)-4-oxo-4*H*-chromen-6-yl)-3,4-dihydroxyphenyl)-6-(6-(5,7-dihydroxy-4-oxo-4*H*-chromen-2-yl)-2,3-dihydroxyphenyl)-5,7-dihydroxy-4*H*-chromen-4-one:

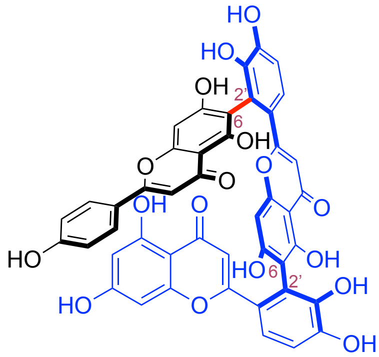

109 mg, 52% yield, brown solid. HPLC (Luna 5 µm C18(2) 100A, LC Column 250 × 4.6 mm, ACN/Water = 71.5: 28.5, flow rate 1.0 mL/min, λ = 300 nm), injection volume= 10 µL, *tr*(**3b***) = 32.142 min, *tr*(**3b****) = 33.539 min, concentration of **3b** = 0.89 mM. **3b** = **3b*** + **3b****. Atropisomer **3b***: ^{1}H NMR (500 MHz, DMSO-d_6) δ 7.90 (d, J = 8.9, 2H), 7.22 (d, J = 8.4 Hz, 1H), 7.15 (d, J = 8.4 Hz, 1H), 6.97 (d, J = 8.5 Hz, 1H), 6.94 (d, J = 8.9 Hz, 2H), 6.92 (d, J = 8.5 Hz, 1H), 6.69 (s, 1H), 6.57 (s, 1H), 6.21 (s, 1H), 6.09 (d, J = 7.8 Hz, 1H), 6.01 (s, 1H), 5.98 (d, J = 7.8 Hz, 1H), 5.97 (s, 1H). Atropisomer **3b****: ^{1}H NMR (500 MHz, DMSO-d_6) δ 7.90 (d, J = 8.9 Hz, 2H), 7.22 (d, J = 8.4, 1H), 7.15 (d, J = 8.4 Hz, 1H), 6.97 (d, J = 8.5 Hz, 1H), 6.94 (d, J = 8.9 Hz, 2H), 6.92 (d, J = 8.5 Hz, 1H), 6.68 (s, 1H), 6.55 (s, 1H), 6.16 (s, 1H), 6.09 (d, J = 7.8 Hz, 1H), 5.99 (s, 1H), 5.98 (d, J = 7.8 Hz, 1H), 5.96 (s, 1H). ^{13}C NMR (126 MHz, DMSO-d_6) δ 182.34, 181.82, 181.69, 166.65, 166.56, 164.53, 164.07, 162.21, 161.76, 161.57, 159.35, 159.09, 157.88, 156.93, 148.82, 144.74, 128.98, 124.18, 121.77, 120.63, 120.15, 116.42, 114.78, 108.41, 108.25, 106.73, 103.97, 103.83, 103.65, 103.45, 99.20, 94.03, 93.86, 93.78. HRMS (ESI-TOF) calcd for $C_{45}H_{25}O_{17}$ = 837.1090, found 837.1097.

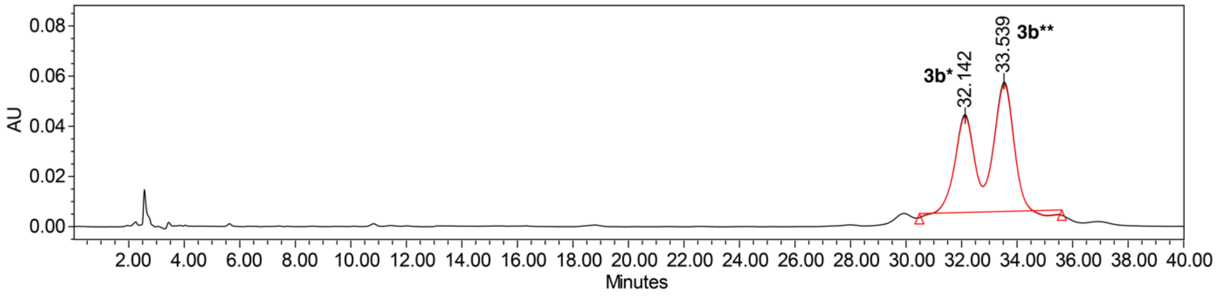

Lu$_2$-Dio (2′-6), 3c, 2-(2-(5,7-dihydroxy-2-(4-hydroxy-3-methoxyphenyl)-4-oxo-4*H*-chromen-6-yl)-3,4-dihydroxyphenyl)-6-(6-(5,7-dihydroxy-4-oxo-4*H*-chromen-2-yl)-2,3-dihydroxyphenyl)-5,7-dihydroxy-4*H*-chromen-4-one:

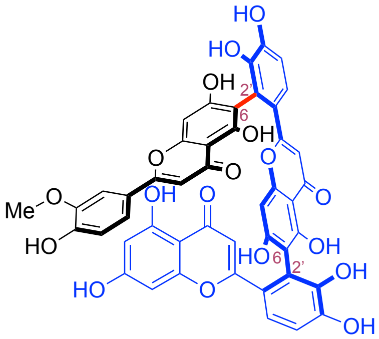

100 mg, 46% yield, brown solid. HPLC (Luna 5 µm C18(2) 100A, LC Column 250 × 4.6 mm, ACN/Water = 64: 36, flow rate 1.0 mL/min, λ = 300 nm), injection volume= 10 µL, *tr*(**3c***) = 10.787 min, *tr*(**3c****) = 10.787 min, concentration of **3c** = 0.50 mM. **3c** = **3c*** + **3c****. Atropisomer **3c***: ^{1}H NMR (500 MHz, DMSO-*d*$_6$) δ 13.19 (s, 1H), 13.00 (s, 1H), 12.79 (s, 1H), 10.76 (s, 4H), 10.16 (s, 2H), 8.44 (s, 2H), 7.62 (d, *J* = 8.6 Hz, 1H), 7.46 (m, 1H), 7.26 (d, *J* = 8.4, 1H), 7.16 (d, *J* = 8.7 Hz, 1H), 7.09 (d, *J* = 8.6 Hz, 1H), 6.98 (d, *J* = 8.5, 1H), 6.93 (d, *J* = 8.4 Hz, 1H), 6.78 (s, 1H), 6.60 (s, 1H), 6.28 (s, 1H), 6.12 (d, *J* = 8.1Hz, 1H), 6.09 (s, 1H), 6.02 (d, *J* = 8.1 Hz, 1H), 5.96 (s, 1H), 3.87 (s, 3H). Atropisomer **3c****: ^{1}H NMR (500 MHz, DMSO-*d*$_6$) δ 13.16 (s, 1H), 12.96 (s, 1H), 12.76 (s, 1H), 10.76 (s, 4H), 10.16 (s, 2H), 8.44 (s, 2H), 7.62 (d, *J* = 8.6 Hz, 1H), 7.46 (m, 1H), 7.26 (d, *J* = 8.4, 1H), 7.16 (d, *J* = 8.7 Hz, 1H), 7.09 (d, *J* = 8.6 Hz, 1H), 6.98 (d, *J* = 8.5, 1H), 6.93 (d, *J* = 8.4 Hz, 1H), 6.78 (s, 1H), 6.56 (s, 1H), 6.12 (d, *J* = 8.1 Hz, 3H), 6.09 (s, 1H), 6.02 (d, *J* = 8.1 Hz, 1H), 5.97 (s, 1H), 5.95 (s, 1H), 3.87 (s, 3H). ^{13}C NMR (126 MHz, DMSO-*d*$_6$) δ 182.33, 182.26, 182.18, 181.86, 181.75, 181.69, 181.63, 166.68, 166.63, 166.58, 166.54, 166.28, 164.67, 164.53, 164.48, 164.01, 163.87, 163.83, 162.27, 162.21, 162.07, 161.95, 161.77, 159.38, 159.32, 159.09, 157.89, 157.85, 157.80, 157.10, 156.93, 156.83, 151.61, 151.58, 151.55, 148.85, 148.79, 148.70, 147.26, 147.20, 144.73, 144.69, 144.63, 124.19, 124.06, 123.60, 123.57, 123.48, 120.83, 120.65, 120.19, 120.14, 120.10, 119.24, 119.20, 114.82, 114.74, 113.48, 113.42, 113.37, 112.65, 112.61, 112.55, 108.45, 108.32, 108.25, 108.18, 106.84, 106.76, 106.66, 104.24, 104.13, 104.06, 104.00, 103.95, 103.86, 103.82, 103.68, 103.62, 99.36, 99.31, 99.20, 94.40, 94.35, 93.90, 93.85, 93.80, 93.47, 93.29, 56.22, 56.17. HRMS (ESI-TOF) calcd for $C_{46}H_{27}O_{18}$ = 867.1203, found 867.1202.

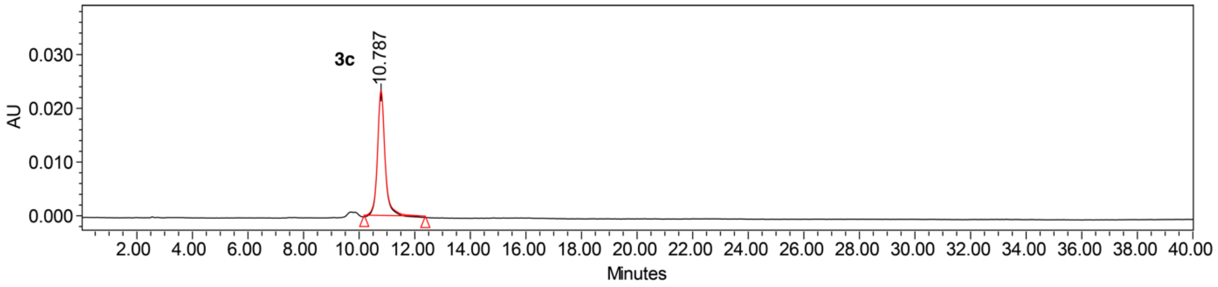

Lu$_2$-Chry (2′-6), 3d, 2-(2-(5,7-dihydroxy-4-oxo-2-phenyl-4*H*-chromen-6-yl)-3,4-dihydroxyphenyl)-6-(6-(5,7-dihydroxy-4-oxo-4*H*-chromen-2-yl)-2,3-dihydroxyphenyl)-5,7-dihydroxy-4*H*-chromen-4-one:

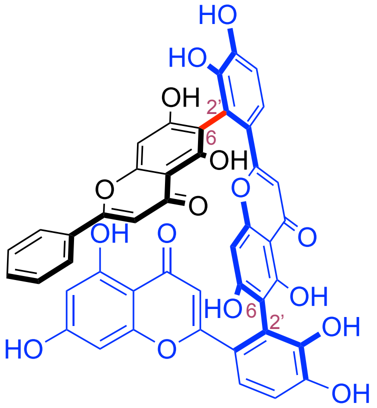

82 mg, 40% yield, brown solid. HPLC (Luna 5 µm C18(2) 100A, LC Column 250 × 4.6 mm, ACN/Water = 64: 36, flow rate 1.0 mL/min, λ = 300 nm), injection volume= 10 µL, *tr*(**3d***) = 9.863 min, *tr*(**3d****) = 9.863 min, concentration of **3d** = 2.13 mM. **3d** = **3d*** + **3d****. Atropisomer **3d***: ^{1}H NMR (500 MHz, DMSO-d_6) δ 8.07 – 8.00 (m, 2H), 7.65 – 7.53 (m, 3H), 7.23 (d, *J* = 8.4, Hz, 1H), 7.14 (dd, *J* = 8.3, 1H), 6.99 (d, *J* = 8.4 Hz, 1H), 6.93 (d, *J* = 8.4 Hz, 1H), 6.85 (s, 1H), 6.65 (s, 1H), 6.24 (s, 1H), 6.11 (d, *J* = 6.1 Hz, 1H), 6.06 (s, 1H), 6.02 (d, *J* = 6.1 Hz, 1H), 5.96 (s, 1H). Atropisomer **3d****: ^{1}H NMR (500 MHz, DMSO-d_6) δ 8.07 – 8.00 (m, 2H), 7.65 – 7.53 (m, 3H), 7.23 (d, *J* = 8.4 Hz, 1H), 7.14 (d, *J* = 8.3 Hz, 1H), 6.99 (d, *J* = 8.4 Hz, 1H), 6.93 (d, *J* = 8.3 Hz, 1H), 6.85 (s, 1H), 6.63 (s, 1H), 6.16 (s, 1H), 6.11 (d, *J* = 6.1 Hz, 1H), 6.02 (s, 1H), 6.01 (d, *J* = 6.1 Hz, 1H), 5.94 (s, 1H). ^{13}C NMR (126 MHz, DMSO-d_6) δ 182.48, 181.85, 181.69, 181.67, 166.67, 166.62, 166.32, 164.58, 163.57, 163.52, 162.20, 161.75, 159.32, 159.08, 159.04, 157.89, 157.08, 156.98, 156.93, 148.79, 148.70, 144.72, 144.62, 132.44, 131.28, 129.62, 126.93, 124.17, 120.64, 120.12, 120.08, 114.87, 114.77, 108.68, 108.54, 108.26, 108.20, 106.73, 105.77, 104.23, 103.82, 103.79, 103.65, 99.22, 93.89, 93.29. HRMS (ESI-TOF) calcd for $C_{45}H_{25}O_{16}$ = 821.1148, found 821.1135.

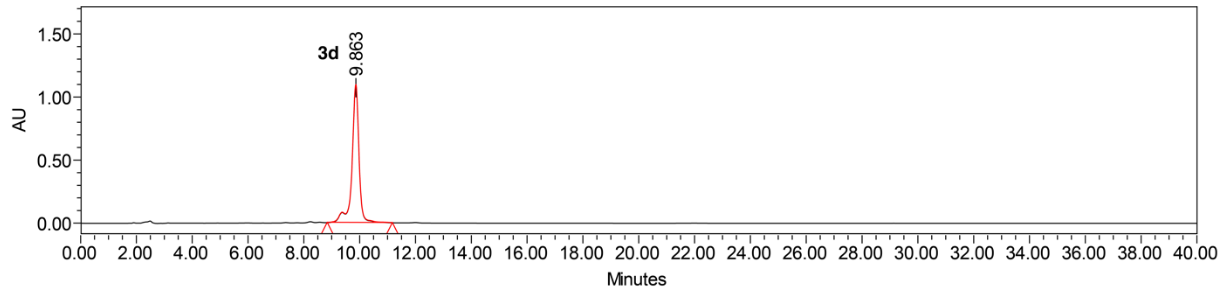

Lu$_2$-Wo (2′-6), 3e, 6-(6-(6-(6-(5,7-dihydroxy-4-oxo-4*H*-chromen-2-yl)-2,3-dihydroxyphenyl)-5,7-dihydroxy-4-oxo-4*H*-chromen-2-yl)-2,3-dihydroxyphenyl)-5,7-dihydroxy-8-methoxy-2-phenyl-4*H*-chromen-4-one:

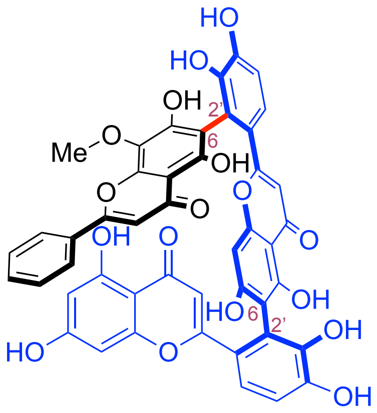

115 mg, 54% yield, brown solid. HPLC (Luna 5 µm C18(2) 100A, LC Column 250 × 4.6 mm, ACN/Water = 64: 36, flow rate 1.0 mL/min, λ = 300 nm), injection volume= 10 µL, *tr*(**3e***) = 21.393 min, *tr*(**3e****) = 22.891 min, concentration of **3e** = 1.90 mM. **3e** = **3e*** + **3e****. Atropisomer **3e***: ^{1}H NMR (500 MHz, DMSO-d_6) δ 12.96 (s, 1H), 12.79 (s, 1H), 12.75 (s, 1H), 10.77 (s, 1H), 10.63 (s, 1H), 10.31 (s, 1H), 10.26 (s, 1H), 8.51 (s, 1H), 8.38 (s, 1H), 8.09 (d, *J* = 7.7 Hz, 2H), 7.68 – 7.56 (m, 3H), 7.26 (d, *J* = 8.4, 1H), 7.14 (d, *J* = 8.4 Hz, 1H), 7.04 (s, 1H), 6.98 (d, *J* = 8.4, 1H), 6.91 (d, *J* = 8.4, 1H), 6.15 (s, 1H), 6.12 (d, *J* = 2.1 Hz, 1H), 6.04 (d, *J* = 2.1 Hz, 1H), 6.01 (s, 1H), 5.95 (s, 1H). Atropisomer **3e****: ^{1}H NMR (500 MHz, DMSO-d_6) δ 12.96 (s, 1H), 12.77 (s, 1H), 12.74 (s, 1H), 10.77 (s, 1H), 10.57 (s, 1H), 10.27 (s, 1H), 10.12 (s, 1H), 8.51 (s, 1H), 8.35 (s, 1H), 8.09 (d, *J* = 7.7 Hz, 2H), 7.68 – 7.56 (m, 3H), 7.26 (d, *J* = 8.4, 1H), 7.14 (d, *J* = 8.4 Hz, 1H), 7.04 (s, 1H), 6.98 (d, *J* = 8.4, 1H), 6.91 (d, *J* = 8.4, 1H), 6.11 (s, 1H), 6.08 (d, *J* = 2.1 Hz, 1H), 5.97 (s, 1H), 5.95 (d, *J* = 2.1 Hz, 1H), 5.92 (s, 1H). ^{13}C NMR (126 MHz, DMSO-d_6) δ 182.69, 182.59, 181.90, 181.78, 181.68, 181.64, 166.70, 166.59, 166.36, 164.47, 163.33, 162.21, 162.12, 161.76, 159.12, 159.00, 157.92, 157.84, 157.01, 156.78, 155.40, 154.58, 149.11, 148.88, 148.68, 144.76, 144.62, 132.52, 131.41, 129.75, 127.99, 127.87, 126.78, 124.30, 124.15, 124.01, 123.93, 120.74, 120.58,

120.14, 119.80, 114.91, 114.70, 108.86, 108.74, 108.20, 106.83, 106.71, 105.77, 103.90, 103.81, 103.64, 99.16, 93.93, 93.80, 93.38, 93.24, 61.97. HRMS (ESI-TOF) calcd for $C_{46}H_{27}O_{17}$ = 851.1254, found 851.1248.

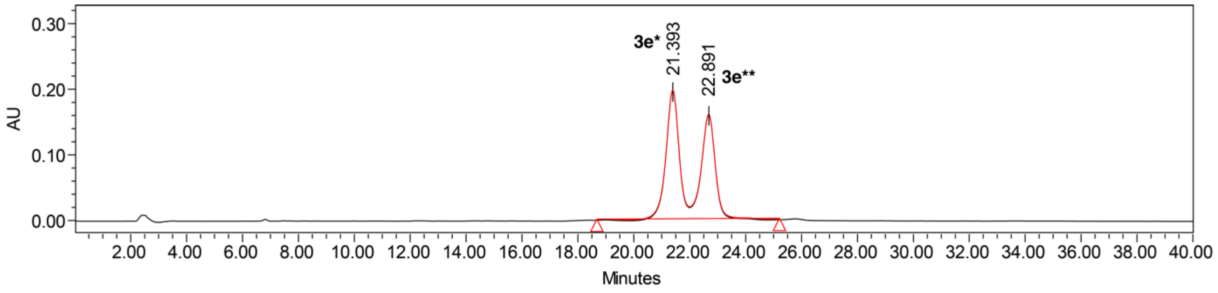

Lu$_2$-56 (2′-6), 3f, 2-(2-(5,6-dihydroxy-4-oxo-2-phenyl-4*H*-chromen-7-yl)-3,4-dihydroxyphenyl)-6-(6-(5,7-dihydroxy-4-oxo-4*H*-chromen-2-yl)-2,3-dihydroxyphenyl)-5,7-dihydroxy-4*H*-chromen-4-one:

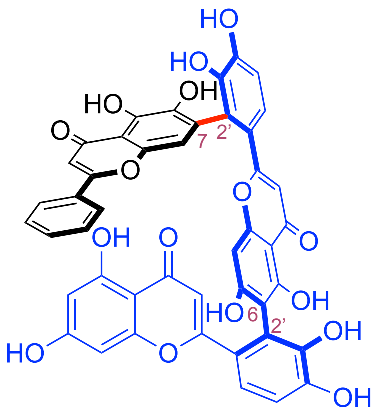

154 mg, 75% yield, brown solid. HPLC (Luna 5 µm C18(2) 100A, LC Column 250 × 4.6 mm, ACN/Water = 64: 36, flow rate 1.0 mL/min, λ = 300 nm), injection volume= 10 µL, *tr*(**3f***) = 9.731 min, *tr*(**3f****) = 9.731 min, concentration of **3f** = 3.50 mM. **3f** = **3f*** + **3f****. Atropisomer **3f***: ^{1}H NMR (400 MHz, DMSO-d_6) δ 7.67 (d, *J* = 7.1 Hz, 1H), 7.59 (d, *J* = 7.1 Hz, 1H), 7.50 (dt, *J* = 7.1, 3.8 Hz, 1H), 7.46 – 7.39 (m, 2H), 7.38 (d, *J* = 6.9 Hz, 1H), 7.33 (d, *J* = 7.1 Hz, 1H), 7.24 (s, 1H), 7.07 (s, 1H), 6.90 (d, *J* = 3.8 Hz, 1H), 6.70 (s, 2H), 6.60 (d, *J* = 6.9 Hz, 1H), 6.44 (d, *J* = 7.1 Hz, 1H), 6.37 (s, 1H), 5.89 (s, 1H). Atropisomer **3f****: ^{1}H NMR (400 MHz, DMSO-d_6) δ 7.67 (d, *J* = 7.1 Hz, 1H), 7.59 (d, *J* = 7.1 Hz, 1H), 7.50 (dt, *J* = 7.1, 3.8 Hz, 1H), 7.46 – 7.39 (m, 2H), 7.38 (d, *J* = 6.9 Hz, 1H), 7.33 (d, *J* = 7.1 Hz, 1H), 7.34 (s, 1H), 6.93 (s, 1H), 6.90 (d, *J* = 3.8 Hz, 1H), 6.70 (s, 2H), 6.60 (d, *J* = 6.9 Hz, 1H), 6.44 (d, *J* = 7.1 Hz, 1H), 6.43(s, 1H), 5.81 (s, 1H). ^{13}C NMR (126 MHz, DMSO-d_6) δ 184.34, 184.25, 182.02, 181.89, 181.84, 166.66, 166.04, 164.17, 164.08, 164.00, 163.95, 162.39, 162.31, 162.26, 161.65, 161.30, 159.52, 158.94, 156.81, 156.70, 155.37, 155.31, 150.17, 150.11,

148.78, 148.75, 146.74, 146.68, 146.49, 146.45, 146.16, 144.66, 144.62, 141.05, 140.92, 132.66, 132.57, 131.22, 129.61, 129.53, 126.44, 126.41, 126.32, 126.12, 124.09, 123.87, 121.97, 121.92, 120.21, 120.18, 119.92, 119.49, 119.41, 116.48, 114.37, 113.78, 111.13, 111.08, 110.67, 110.56, 107.74, 107.47, 107.00, 106.81, 104.88, 103.97, 103.93, 103.84, 103.78, 103.41, 103.29, 102.23, 102.17, 98.99, 93.80, 93.48. HRMS (ESI-TOF) calcd for $C_{45}H_{25}O_{16}$ = 821.1148, found 821.1133.

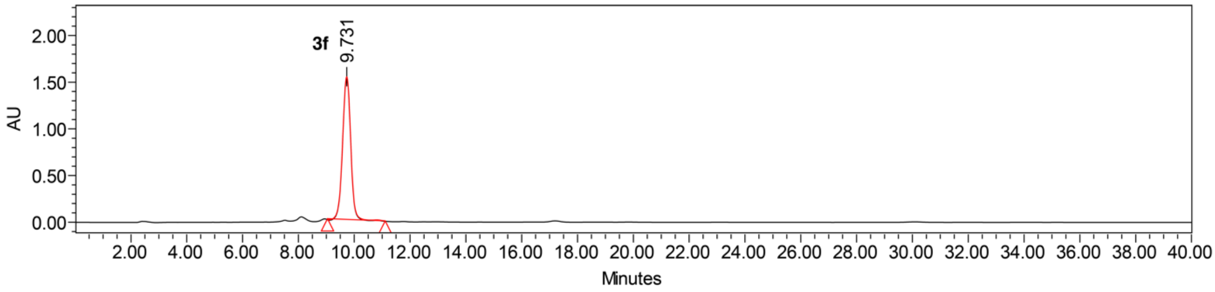

Lu$_2$-Ge (2′-6), 3g, 2-(2-(5,7-dihydroxy-3-(4-hydroxyphenyl)-4-oxo-4*H*-chromen-6-yl)-3,4-dihydroxyphenyl)-6-(6-(5,7-dihydroxy-4-oxo-4*H*-chromen-2-yl)-2,3-dihydroxyphenyl)-5,7-dihydroxy-4*H*-chromen-4-one:

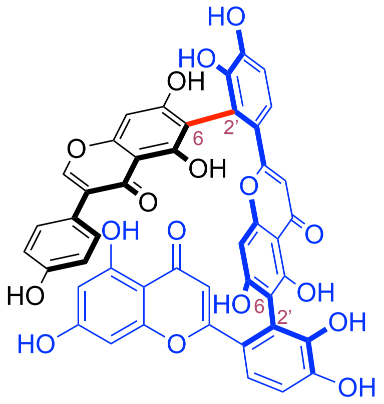

103 mg, 49% yield, brown solid. HPLC (Luna 5 µm C18(2) 100A, LC Column 250 × 4.6 mm, ACN/Water = 64: 36, flow rate 1.0 mL/min, λ = 300 nm), injection volume= 10 µL, *tr*(**3g***) = 10.218 min, *tr*(**3g****) = 11.126 min, concentration of **3g** = 3.88 mM. **3g** = **3g*** + **3g****. Atropisomer **3g***: ^{1}H NMR (400 MHz, DMSO-d_6) δ 8.31 (s, 1H), 7.39 (d, *J* = 8.2 Hz, 2H), 7.25 (d, *J* = 8.4 Hz, 1H), 7.18 (d, *J* = 8.3 Hz, 1H), 6.98 (d, *J* = 8.4 Hz, 1H), 6.94 (d, *J* = 8.3 Hz, 1H), 6.83 (d, *J* = 8.2 Hz, 2H), 6.52 (s, 1H), 6.28 (s, 1H), 6.13 (d, *J* = 2.1 Hz, 1H), 6.09 (s, 1H), 6.04 (d, *J* = 2.1 Hz, 1H), 5.96 (s, 1H). Atropisomer **3g****: ^{1}H NMR (400 MHz, DMSO-d_6) δ 8.34 (s, 1H), 7.42 (d, *J* = 8.4 Hz, 2H), 7.25 (d, *J* = 8.2 Hz, 1H), 7.17 (d, *J* = 8.3 Hz, 1H), 6.97 (d, *J* = 8.2 Hz, 1H), 6.93 (d, *J* = 8.3 Hz, 1H), 6.83 (d, *J* = 8.2 Hz, 2H), 6.48 (s, 1H), 6.28 (s, 1H), 6.11 (d, *J* = 2.1 Hz, 1H), 6.09 (s, 1H), 6.01 (d, *J* = 2.1 Hz, 1H), 5.97 (s, 1H). ^{13}C NMR (126 MHz, DMSO-d_6) δ 181.86, 181.75, 181.68, 180.85, 180.77, 166.67, 166.62,

166.60, 166.32, 164.51, 164.49, 162.42, 162.21, 162.17, 161.78, 161.75, 159.86, 159.75, 159.10, 157.90, 157.85, 157.83, 157.17, 157.09, 157.06, 156.91, 154.27, 148.82, 148.77, 148.71, 148.68, 144.70, 144.66, 144.64, 144.61, 130.73, 130.70, 130.63, 124.23, 124.17, 124.11, 122.84, 122.80, 121.86, 121.78, 120.66, 120.18, 120.12, 115.52, 115.49, 114.83, 108.51, 108.38, 108.22, 106.89, 106.79, 106.73, 104.77, 104.74, 103.86, 103.83, 103.68, 99.19, 93.89, 93.84, 93.62, 93.48, 93.27. HRMS (ESI-TOF) calcd for $C_{45}H_{25}O_{17}$ = 837.1097, found 837.1087.

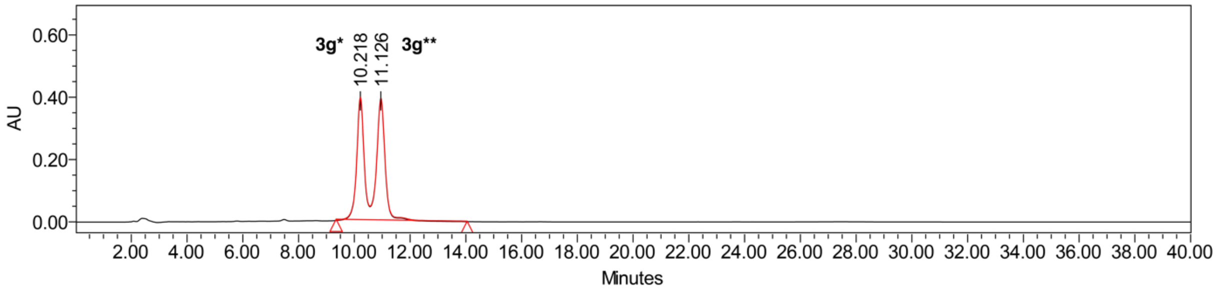

534-Lu₂ (2′-6), 3h, 6-(2,3-dihydroxy-6-(5-hydroxy-4-oxo-4*H*-chromen-2-yl)phenyl)-2-(2-(2-(3,4-dihydroxyphenyl)-5,7-dihydroxy-4-oxo-4*H*-chromen-6-yl)-3,4-dihydroxyphenyl)-5,7-dihydroxy-4*H*-chromen-4-one:

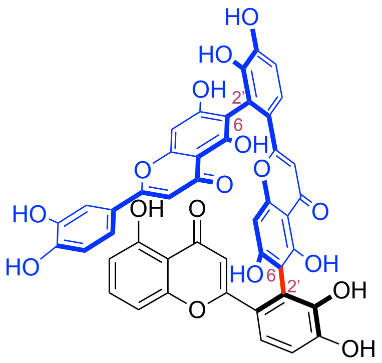

88 mg, 42% yield, brown solid. HPLC (Luna 5 μm C18(2) 100A, LC Column 250 × 4.6 mm, ACN/Water = 64: 36, flow rate 1.0 mL/min, λ = 300 nm), injection volume= 10 μL, *tr*(**3h***) = 11.579 min, *tr*(**3h****) = 12.618 min, concentration of **3h** = 3.43 mM. **3h** = **3h*** + **3h****. Atropisomer **3h***: ^{1}H NMR (500 MHz, DMSO-d_6) δ 7.46 (td, *J* = 8.3, 3.8 Hz, 1H), 7.44 – 7.37 (m, 2H), 7.22 (d, *J* = 8.3 Hz, 1H), 7.21 (d, *J* = 8.2 Hz, 1H), 6.98 (d, *J* = 8.3, 1H), 6.95 (d, *J* = 8.2 Hz, 1H), 6.93 (d, *J* = 8.2 Hz, 1H), 6.67 (d, *J* = 3.8 Hz, 1H), 6.61 (s, 1H), 6.58 (s, 1H), 6.52 (d, *J* = 8.3 Hz, 1H), 6.24 (s, 1H), 6.15 (s, 1H), 6.08 (s, 1H). Atropisomer **3h****: ^{1}H NMR (500 MHz, DMSO-d_6) δ 7.46 (td, *J* = 8.3, 3.8 Hz, 1H), 7.44 – 7.37 (m, 2H), 7.22 (d, *J* = 8.3, 1H), 7.21 (d, *J* = 8.2 Hz, 1H), 6.98 (d, *J* = 8.3 Hz, 1H), 6.95 (d, *J* = 8.2 Hz, 1H), 6.93 (d, *J* = 8.2 Hz, 1H), 6.67 (d, *J* = 8.1 Hz, 1H), 6.60 (s, 1H), 6.57 (s, 1H), 6.46 (d, *J* = 8.1 Hz, 1H),

6.19 (s, 1H), 6.15 (s, 1H), 6.02 (s, 1H). ^{13}C NMR (126 MHz, DMSO-d_6) δ 182.91, 182.86, 182.25, 182.21, 181.84, 181.74, 167.83, 166.72, 166.46, 164.20, 164.16, 162.25, 162.19, 162.05, 160.20, 159.27, 159.05, 159.00, 157.07, 156.91, 156.85, 156.77, 156.24, 156.19, 150.13, 150.08, 149.05, 148.81, 148.73, 146.18, 144.72, 144.68, 136.03, 135.97, 124.27, 124.05, 123.87, 123.80, 122.05, 122.00, 121.06, 120.90, 120.67, 120.27, 120.21, 120.17, 119.47, 116.52, 114.82, 113.79, 111.10, 110.08, 110.03, 108.42, 108.34, 108.27, 108.22, 107.21, 107.13, 107.05, 106.97, 106.85, 106.60, 103.95, 103.65, 103.62, 103.44, 93.73, 93.37, 93.24. HRMS (ESI-TOF) calcd for $C_{45}H_{25}O_{17}$ = 837.1097, found 837.1091.

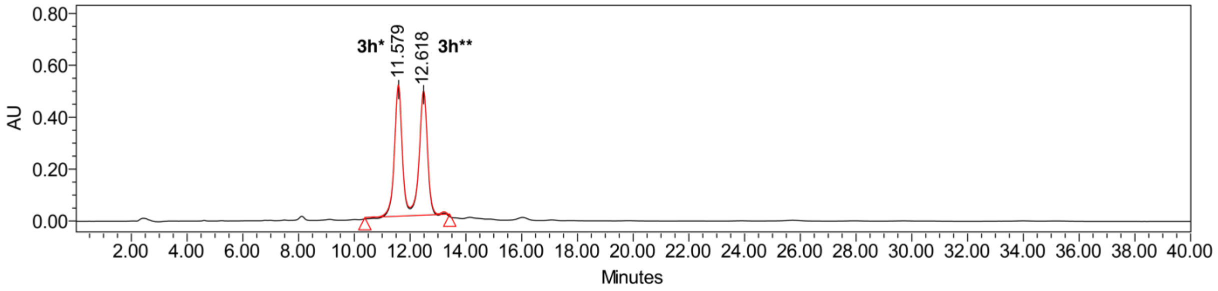

634-Lu₂ (2′-6), 3i, 6-(2,3-dihydroxy-6-(6-hydroxy-4-oxo-4*H*-chromen-2-yl)phenyl)-2-(2-(2-(3,4-dihydroxyphenyl)-5,7-dihydroxy-4-oxo-4*H*-chromen-6-yl)-3,4-dihydroxyphenyl)-5,7-dihydroxy-4*H*-chromen-4-one:

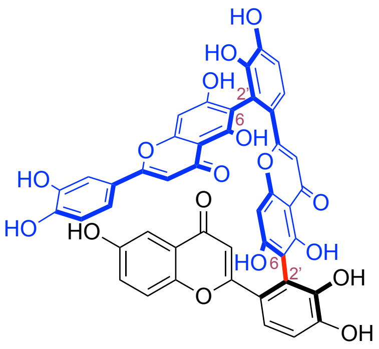

85 mg, 41% yield, brown solid. HPLC (Luna 5 µm C18(2) 100A, LC Column 250 × 4.6 mm, ACN/Water = 72.5: 27.5, flow rate 1.0 mL/min, λ = 300 nm), injection volume= 10 µL, *tr*(**3i***) = 10.218 min, *tr*(**3i****) = 11.126 min, concentration of **3i** = 3.73 mM. **3i** = **3i*** + **3i****. Atropisomer **3i***: ^{1}H NMR (400 MHz, DMSO-d_6) δ 7.44 (d, *J* = 8.5 Hz, 2H), 7.24 (d, *J* = 8.4, 1H), 7.21 (d, *J* = 8.3, 1H), 7.17 – 7.03 (m, 3H), 6.96 (d, *J* = 8.4, 1H), 6.93 (d, *J* = 8.3, 1H), 6.90 (d, *J* = 8.5 Hz, 1H), 6.70 (s, 1H), 6.56 (s, 1H), 6.24 (s, 1H), 6.02 (s, 1H), 5.93 (s, 1H). Atropisomer **3i****: ^{1}H NMR (400 MHz, DMSO-d_6) δ 7.44 (d, *J* = 8.5 Hz, 2H), 7.24 (d, *J* = 8.4, 11H), 7.21 (d, *J* = 8.3, 1H), 7.17 – 7.03 (m, 3H), 6.96 (d, *J* = 8.4, 1H), 6.93 (d, *J* = 8.3, 1H), 6.90 (d, *J* = 8.5 Hz, 1H), 6.70 (s, 1H), 6.54 (s, 1H), 6.09 (s, 1H), 6.05 (s, 1H), 5.97 (s,

1H). ^{13}C NMR (126 MHz, DMSO-d_6) δ 182.28, 182.23, 181.80, 181.72, 176.92, 176.89, 166.52, 166.31, 165.61, 164.24, 164.19, 162.33, 162.17, 161.97, 159.36, 159.32, 159.10, 159.06, 157.03, 156.89, 156.80, 155.05, 150.13, 150.09, 149.87, 149.83, 148.83, 148.77, 148.30, 146.19, 144.71, 144.68, 144.58, 124.91, 124.88, 124.28, 124.23, 124.18, 124.02, 123.25, 122.09, 122.06, 120.81, 120.65, 120.41, 120.19, 120.08, 120.04, 119.50, 119.44, 116.52, 114.81, 113.87, 108.50, 108.39, 108.28, 107.97, 107.92, 107.82, 107.79, 106.76, 106.58, 103.99, 103.62, 103.48, 93.80, 93.74, 93.38, 93.26. HRMS (ESI-TOF) calcd for $C_{45}H_{25}O_{17}$ = 837.1097, found 837.1091.

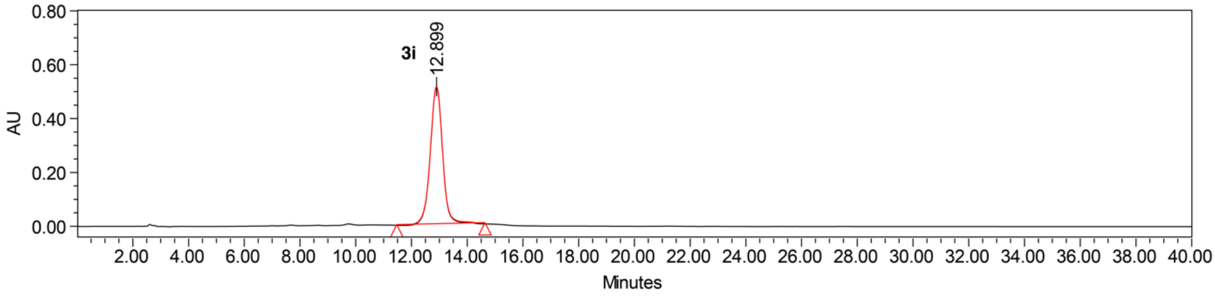

734-Lu₂ (2′-6), 3j, 6-(2,3-dihydroxy-6-(7-hydroxy-4-oxo-4*H*-chromen-2-yl)phenyl)-2-(2-(2-(3,4-dihydroxyphenyl)-5,7-dihydroxy-4-oxo-4*H*-chromen-6-yl)-3,4-dihydroxyphenyl)-5,7-dihydroxy-4*H*-chromen-4-one:

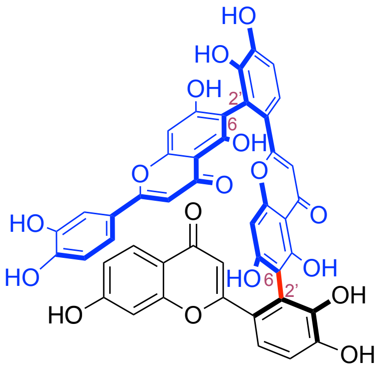

98 mg, 47% yield, brown solid. HPLC (Luna 5 µm C18(2) 100A, LC Column 250 × 4.6 mm, ACN/Water = 72.5: 27.5, flow rate 1.0 mL/min, λ = 300 nm), injection volume= 10 µL, *tr*(**3j***) = 13.331 min, *tr*(**3j****) = 13.331 min, concentration of **3j** = 2.54 mM. **3j** = **3j*** + **3j****.
Atropisomer **3j***: ^1H NMR (400 MHz, DMSO-d_6) δ 7.73 (td, J = 8.7, 6.5 Hz, 1H), 7.49 (d, J = 8.1 Hz, 2H), 7.24 (d, J = 8.4 Hz, 1H), 7.14 (d, J = 8.3 Hz, 1H), 6.97 (d, J = 8.4 Hz, 1H), 6.93 (d, J = 8.3 Hz, 1H), 6.92 (d, J = 8.1 Hz, 1H), 6.81 (dd, J = 8.7, 6.5 Hz, 1H), 6.71 (s, 1H), 6.56 (s, 1H), 6.52 (d, J = 6.5 Hz, 1H), 6.27 (s, 1H), 6.05 (s, 1H), 5.90 (s, 1H), 3.18 (s, 1H).
Atropisomer **3j****: ^1H NMR (400 MHz, DMSO-d_6) δ 7.73 (td, J = 8.7, 6.5 Hz, 1H), 7.49 (d, J = 8.1 Hz, 2H), 7.24 (d, J = 8.4 Hz, 1H), 7.14 (d, J = 8.3 Hz, 1H), 6.97 (d, J = 8.4 Hz, 1H),

6.93 (d, J = 8.3 Hz, 1H), 6.92 (d, J = 8.1 Hz, 1H), 6.81 (dd, J = 8.8, 6.5 Hz, 1H), 6.70 (s, 1H), 6.54 (s, 1H), 6.52 (d, J = 6.5 Hz, 1H), 6.09 (s, 1H), 5.93 (s, 1H), 5.92 (s, 1H), 3.18 (s, 1H). ^{13}C NMR (126 MHz, DMSO-d_6) δ 206.96, 182.26, 182.19, 181.80, 181.71, 176.37, 166.50, 166.20, 165.23, 165.15, 164.20, 164.16, 162.87, 162.28, 162.15, 161.95, 159.36, 159.31, 159.11, 157.97, 157.04, 156.90, 156.86, 156.77, 150.11, 150.07, 148.80, 148.74, 148.22, 148.18, 146.18, 144.68, 144.64, 144.52, 126.76, 126.74, 124.88, 124.20, 124.08, 122.08, 122.04, 120.27, 120.21, 120.06, 120.02, 119.47, 116.53, 116.18, 116.14, 115.19, 114.84, 113.89, 108.57, 108.40, 108.32, 108.21, 106.83, 106.66, 103.99, 103.96, 103.64, 103.45, 102.41, 93.85, 93.73, 93.30. HRMS (ESI-TOF) calcd for $C_{45}H_{25}O_{17}$ = 837.1097, found 837.1086.

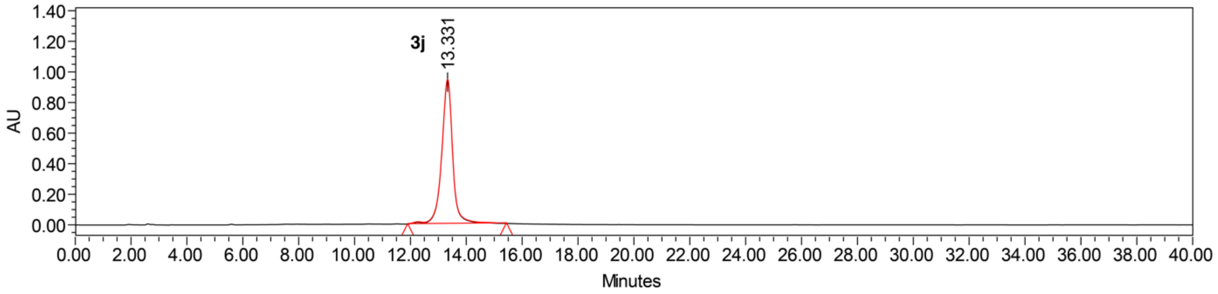

734-Lu-Ap (2′-6), 3k, 6-(2,3-dihydroxy-6-(7-hydroxy-4-oxo-4*H*-chromen-2-yl)phenyl)-2-(2-(2-(3,4-dihydroxyphenyl)-5,7-dihydroxy-4-oxo-4*H*-chromen-6-yl)-3,4-dihydroxyphenyl)-5,7-dihydroxy-4*H*-chromen-4-one:

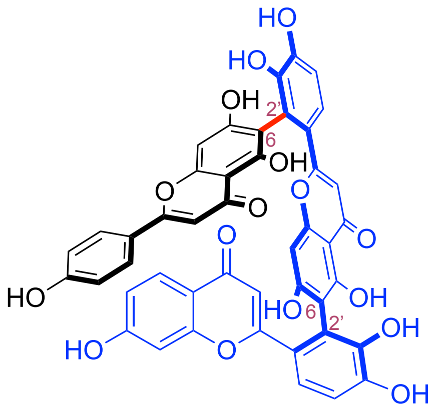

88 mg, 43% yield, brown solid. HPLC (Luna 5 μm C18(2) 100A, LC column 250 × 4.6 mm, ACN/Water = 71.5: 28.5, flow rate 1.0 mL/min, λ = 300 nm), injection volume= 10 μL, *tr*(**3k**) = 15.699 min, concentration of **3k** = 0.76 mM. **3k** = **3k*** + **3k****. Atropisomer **3k***: ^{1}H NMR (500 MHz, DMSO-d_6) δ 13.20 (d, J = 13.1 Hz, 1H), 12.94 (d, J = 20.1 Hz, 1H), 10.37 (s, 8H), 7.94 (d, J = 8.4 Hz, 2H), 7.71 (d, J = 8.4 Hz, 1H), 7.22 (d, J = 8.8 Hz, 1H), 7.13 (d, J = 7.9 Hz, 1H), 6.93 (q, J = 10.4 Hz, 3H), 6.78 (t, J = 5.9 Hz, 2H), 6.53 (s, *1*H), 6.44 (s, 1H),

6.22 (s, 1H), 5.98 (s, 1H), 5.86 (s, 1H). Atropisomer **3k****: ^{1}H NMR (500 MHz, DMSO-d_6) δ 13.20 (d, J = 13.1 Hz, 1H), 12.94 (d, J = 20.1 Hz, 1H), 10.37 (s, 8H), 7.94 (d, J = 8.4 Hz, 2H), 7.71 (d, J = 8.4 Hz, 1H), 7.22 (d, J = 8.8 Hz, 1H), 7.13 (d, J = 7.9 Hz, 1H), 6.93 (q, J = 10.4 Hz, 3H), 6.78 (t, J = 5.9 Hz, 2H), 6.53 (s, 1H), 6.44 (s, 1H), 6.12 (s, 1H), 5.91 (s, 1H), 5.89 (s, 1H). ^{13}C NMR (126 MHz, DMSO-d_6) δ 182.23, 181.77, 176.41, 166.57, 166.38, 165.29, 163.97, 162.50, 161.55, 159.38, 159.28, 159.10, 157.99, 156.96, 148.81, 148.26, 144.76, 144.62, 128.96, 126.68, 126.67, 124.76, 124.20, 121.80, 120.55, 120.24, 120.12, 116.41, 114.64, 108.47, 108.38, 106.67, 106.66, 103.54, 103.39, 102.35, 102.32, 93.97, 93.37. HRMS (ESI-TOF) calcd for $C_{45}H_{25}O_{16}$ = 821.1148, found 821.1132.

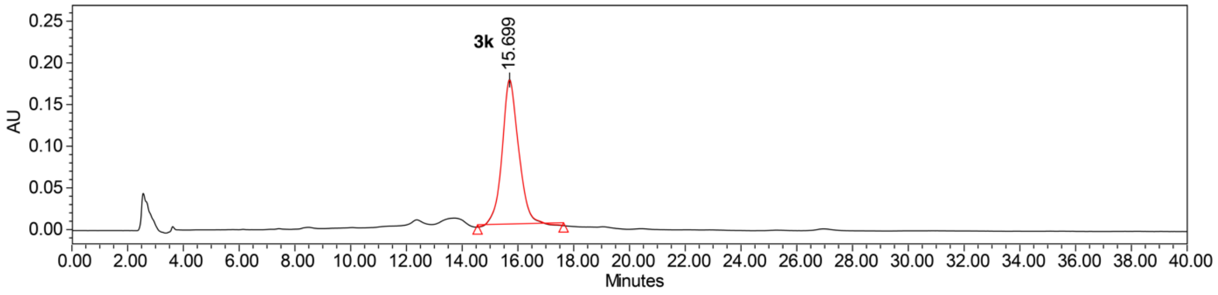

(6′-6)$_3$-cyclotriluteolin, ((6′-6)$_3$-CTL), 4a:

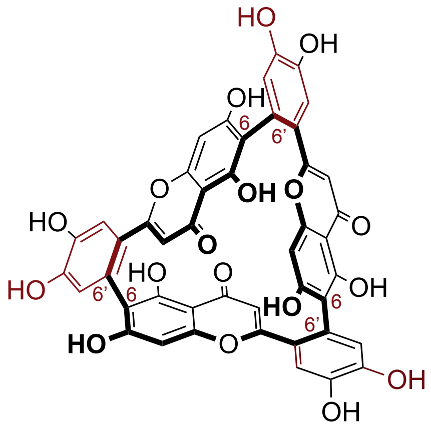

5 mg, 10% yield, white solid. ^{1}H NMR (500 MHz, DMSO-d_6) δ 12.74 (s, 1H), 12.70 (s, 1H), 12.69 (s, 1H), 10.56 (s, 1H), 10.49 (s, 1H), 9.96 (s, 1H), 9.59 (s, 1H), 9.39 (s, 1H), 8.45 (s, 1H), 6.94 (s, 1H), 6.89 (d, J = 2.4 Hz, 4H), 6.63 (s, 1H), 6.30 – 6.24 (m, 3H), 6.06 (d, J = 11.1 Hz, 3H). ^{13}C NMR (126 MHz, DMSO-d_6) δ 182.01, 181.98, 181.94, 168.03, 167.97, 167.67, 163.55, 163.46, 162.95, 158.62, 158.49, 158.46, 156.90, 156.67, 148.23, 148.19, 147.99, 145.20, 145.10, 145.00, 126.42, 126.38, 125.58, 123.87, 120.74, 120.60, 119.90, 119.79, 119.64, 116.96, 114.68, 111.40, 107.94, 107.80, 103.27, 103.10, 93.27. HRMS (ESI-TOF) calcd for $C_{45}H_{23}O_{18}$ = 851.0890, found 851.0901.

(2′-6)₂(6′-6)-cyclotriluteolin, ((2′-6)₂(6′-6)-CTL), 4b:

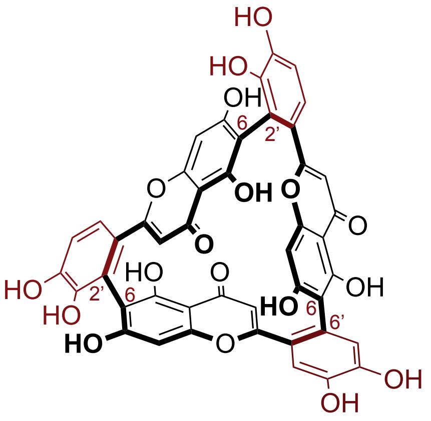

^{1}H NMR (500 MHz, DMSO-d_6) δ 14.73 (s, 1H), 13.17 (s, 1H), 11.93 (s, 1H), 10.69 (s, 1H), 10.54 (s, 1H), 10.24 (s, 1H), 9.98 (s, 1H), 9.69 (s, 1H), 9.43 (s, 1H), 8.43 (d, J = 12.2 Hz, 3H), 8.20 (s, 1H), 7.24 (d, J = 8.5 Hz, 1H), 7.09 (s, 1H), 6.95 – 6.84 (m, 4H), 6.59 (s, 1H), 6.22 (s, 1H), 5.98 (s, 1H), 5.72 (s, 1H), 5.26 (s, 1H). ^{13}C NMR (126 MHz, DMSO-d_6) δ 195.02, 183.13, 182.47, 169.26, 168.37, 165.97, 161.10, 160.11, 159.56, 158.24, 157.75, 157.01, 154.98, 154.58, 149.43, 148.15, 147.17, 145.73, 144.97, 144.74, 126.59, 123.16, 121.80, 121.47, 120.83, 120.08, 120.02, 118.56, 114.75, 114.63, 114.05, 112.68, 108.33, 108.29, 106.39, 106.36, 105.94, 105.81, 103.73, 102.11, 101.15, 95.19, 95.03, 92.67. HRMS (ESI-TOF) calcd for $C_{45}H_{23}O_{18}$ = 851.0890, found 851.0882.

1.2 Supplementary Figures

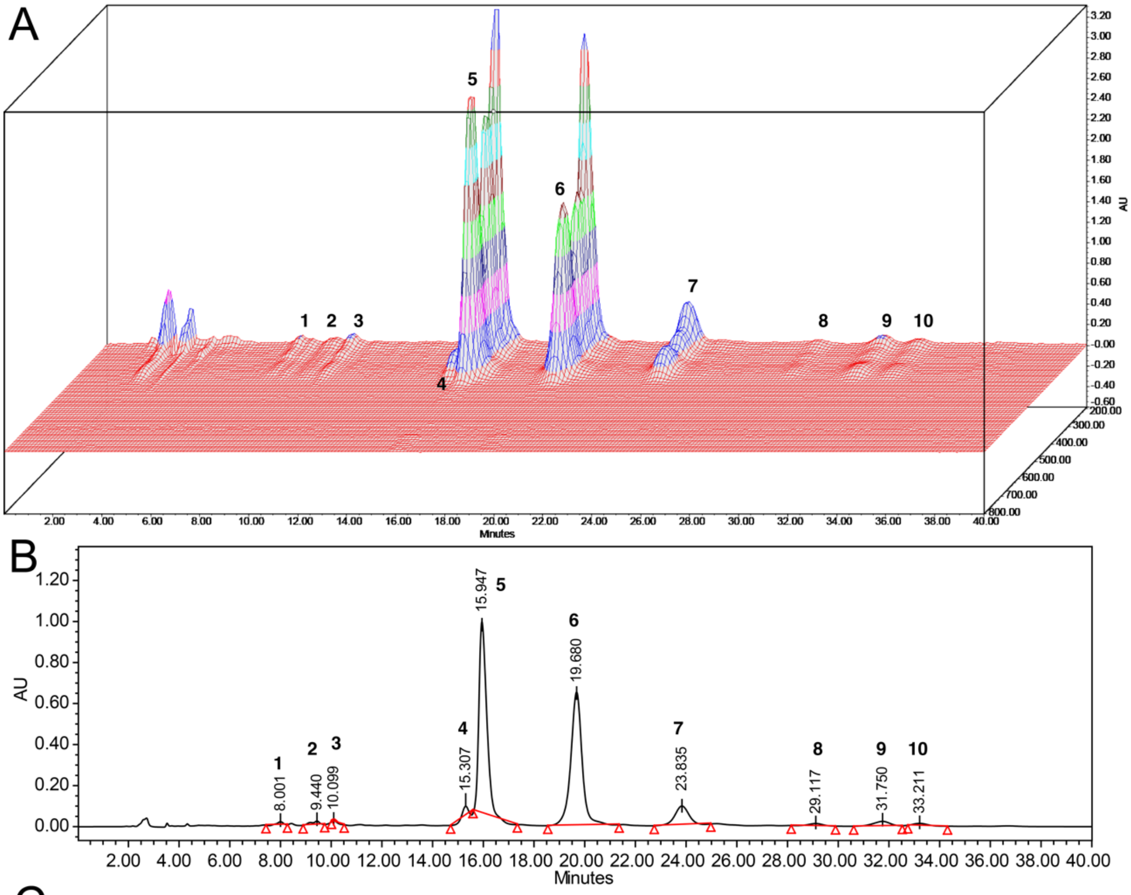

C

Peak No	1, 2, and 3	4	5	6	7	8	9	10
m/z(-)	851.1	569.21	286.157	569.21	853.21	569.21	569.21	569.21
Structures	Cyclic-Lu_3 isomers	Lu_2	Lu	Lu_2	Lu_3		Lu_2 isomers	
	To be determined	*Dehydrohegoflavone*		Dicranolomin	Distichumtriluteolin		Philonotisflavone	

Supplementary Fig. 1. Characterization of reaction products of luteolin in alkaline water. A) 3D HPLC chromatogram of the reaction mixture of luteolin in alkaline water at room temperature overnight. **B)** 2D HPLC chromatogram of the reaction mixture mixture of luteolin in alkaline water at room temperature overnight. **C)** The peak assignments were made based on the molecular weights and the spectra of isolated products (peak **4**, **6**, **7,** and **8**).

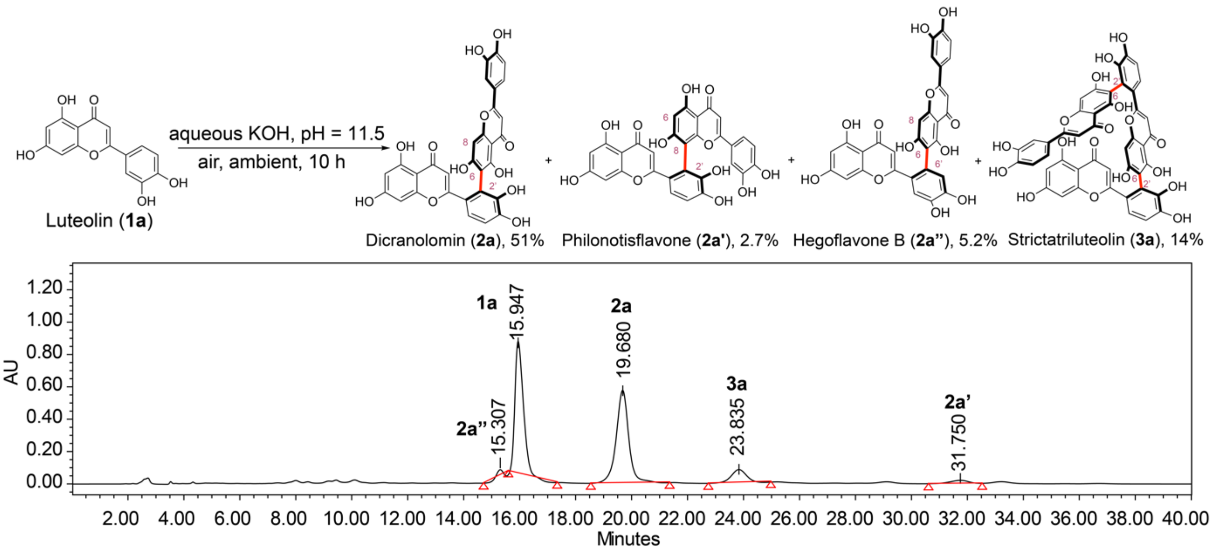

Supplementary Fig. 2. Semi-prep HPLC profile of products of luteolin-luteolin cross-coupling reaction. HPLC method: using 71.5% A isocratic method at 5 mL/min, injection volume is 500 µL. Mobile phase A is DI water with 0.1% formic acid and mobile phase B is acetonitrile with 0.1% formic acid. From left to right, the products were identified as dihydrohegoflavone (**2a″**) at 14.7 min, luteolin (**1a**) at 15.9 min, dicranolomin (**2a**) at 18.9 min and strictatriluteolin (**3a**) at 23.5 min and philonotisflavone (**2a′**) at 31.750 min.

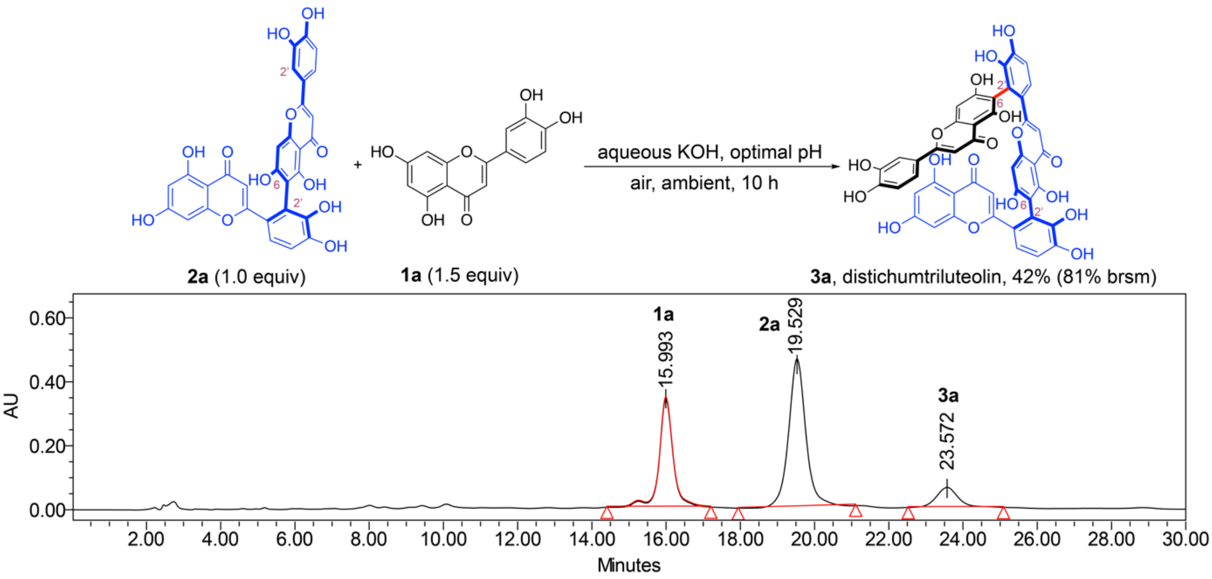

Supplementary Fig. 3. Semi-prep HPLC profile of products of dicranolomin-luteolin cross-coupling reaction. HPLC method: using 71.5% A isocratic method at 5 mL/min, injection volume is 500 µL. Mobile phase A is DI water with 0.1% formic acid and mobile phase B is acetonitrile with 0.1% formic acid. From left to right, the products were identified as luteolin (**1a**) at15.993 min, dicranolomin (**2a**) at 19.529 min and strictatriluteolin (**3a**) at 23.572 min.

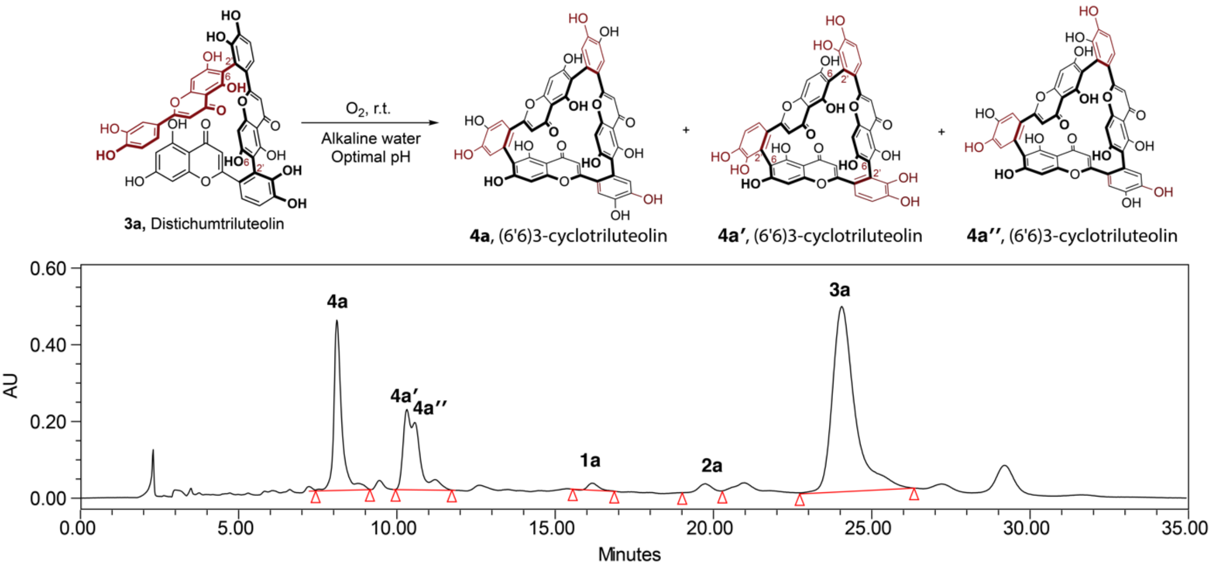

Supplementary Fig. 4. Semi-prep HPLC profile of products of ring intramolecular coupling reaction of distichumtriluteolin (**3a**). HPLC method: using 71.5% A isocratic method at 5 mL/min, injection volume is 500 µL. Mobile phase A is DI water with 0.1% formic acid and mobile phase B is acetonitrile with 0.1% formic acid. From left to right, the products were identified as **4a** at 9.638 min.

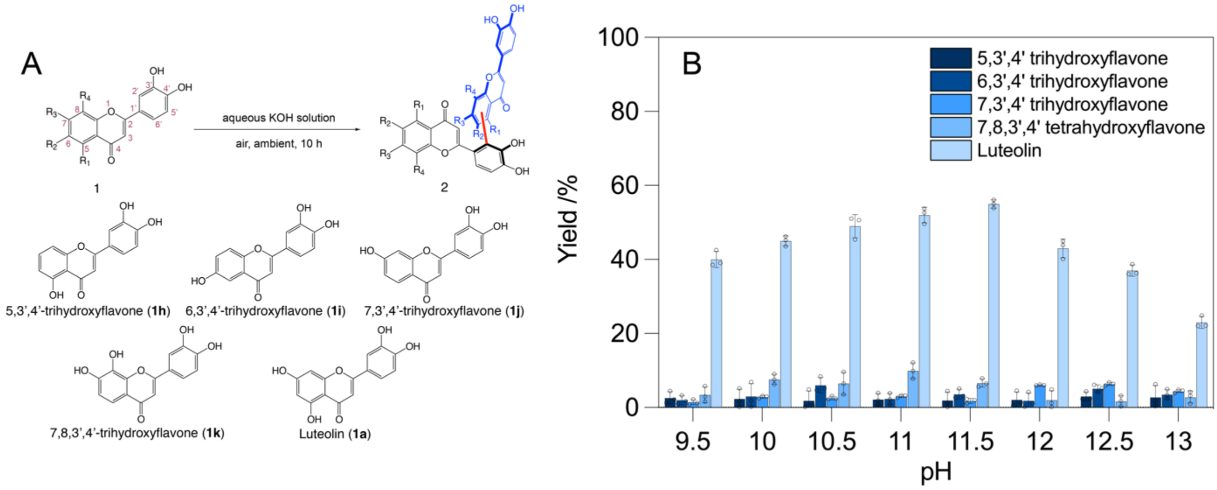

Supplementary Fig. 5. The yields of dimers from homo cross-coupling reaction of B-catechol flavones. **A**) The structure and reaction formular of homo cross-coupling reaction of B-catechol flavones**. B)** The isolated yield of dimer of 5,3',4'-trihydroxyflavone, 6,3',4'-trihydroxyflavone, 7,3',4'-trihydroxyflavone, 7,8,3',4'-tetrahydroxyflavone and luteolin (n = 3). Data are presented as mean ± SD. Reaction condition: B-catechol flavones (0.045 mmol) was dissolved in 3 mL alkaline solution a 15 mL centrifuge tube. The pH was adjusted to specific value with concentrated KOH before the tube was sealed and kept overnight at room temperature without stirring.

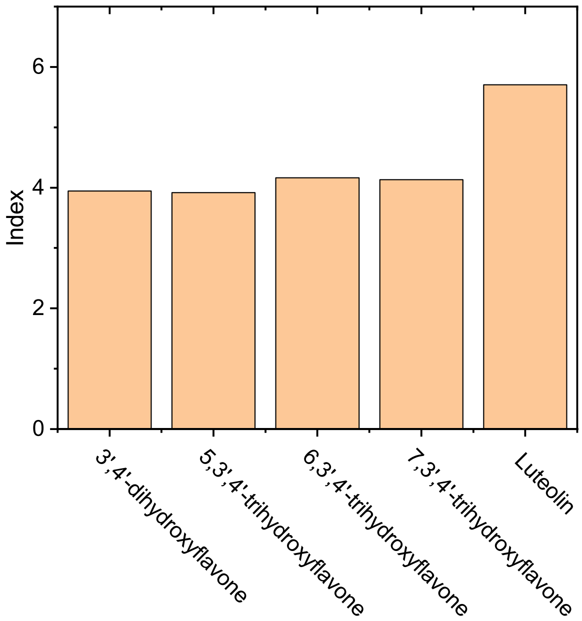

Supplementary Fig. 6. The calculated nucleophilicity index of B-catechol flavones. The global nucleophilicity index (N_{Nu}) was calculated by the DFT method at the B3LYP/6-311++G(d,p) level of theory. The energy of the HOMO was referenced against the HOMO energy of tetracyanoethylene (TCE) according to $N_{Nu} = E_{HOMO(Nu)} - E_{HOMO(TCE)}$.

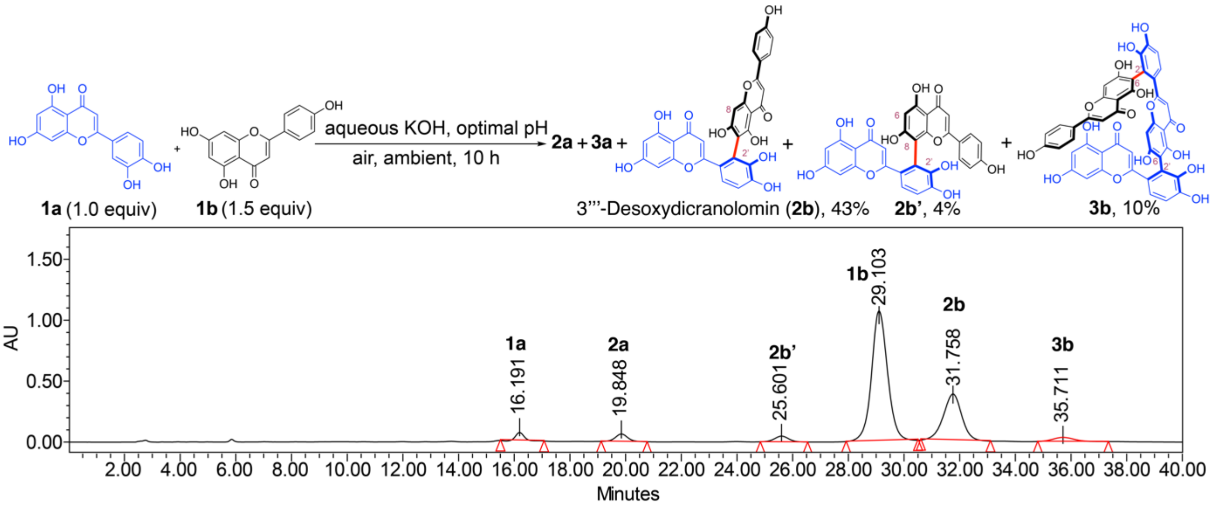

Supplementary Fig. 7. Semi-prep HPLC profile of products of luteolin-apigenin cross-coupling reaction. HPLC method: using 71.5% A isocratic method at 5 mL/min, injection volume is 500 µL. Mobile phase A is DI water with 0.1% formic acid and mobile phase B is acetonitrile with 0.1% formic acid. From left to right, the products were identified as **2b′** at 25.601 min, 3‴-desoxydicranolomin (**2b**) at 31.758 min and **3b** at 35.711 min. The PDA detector was used and the absorbance shown was at 280 nm.

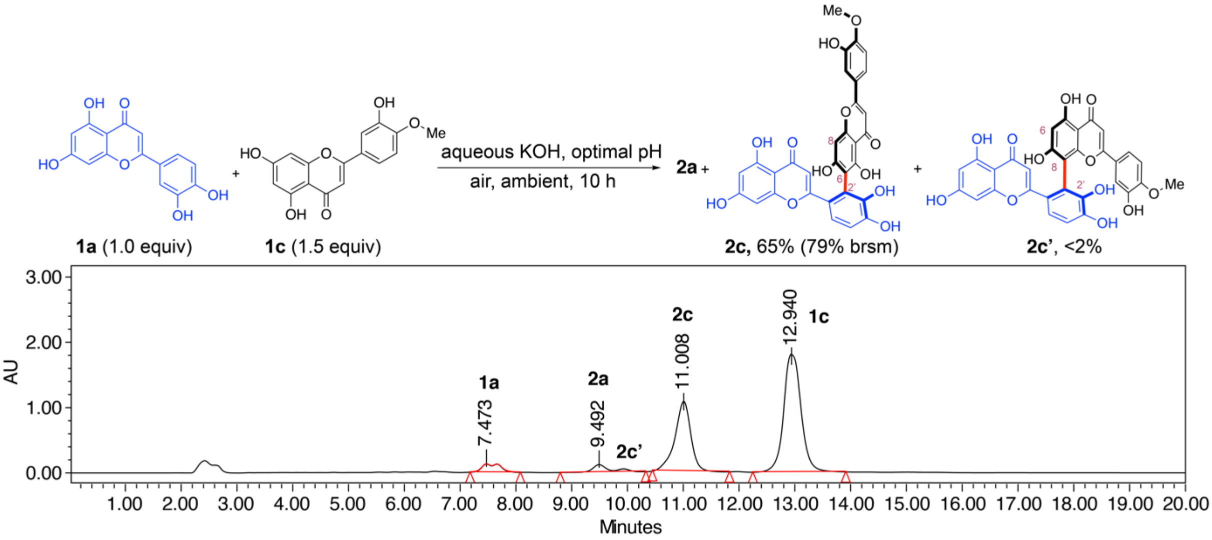

Supplementary Fig. 8. Semi-prep HPLC profile of products of luteolin-diosmetin cross-coupling reaction. HPLC method: using 64% A isocratic method at 5 mL/min, injection volume is 500 μL. Mobile phase A is DI water with 0.1% formic acid and mobile phase B is acetonitrile with 0.1% formic acid. The PDA detector was used and the absorbance shown was at 280 nm.

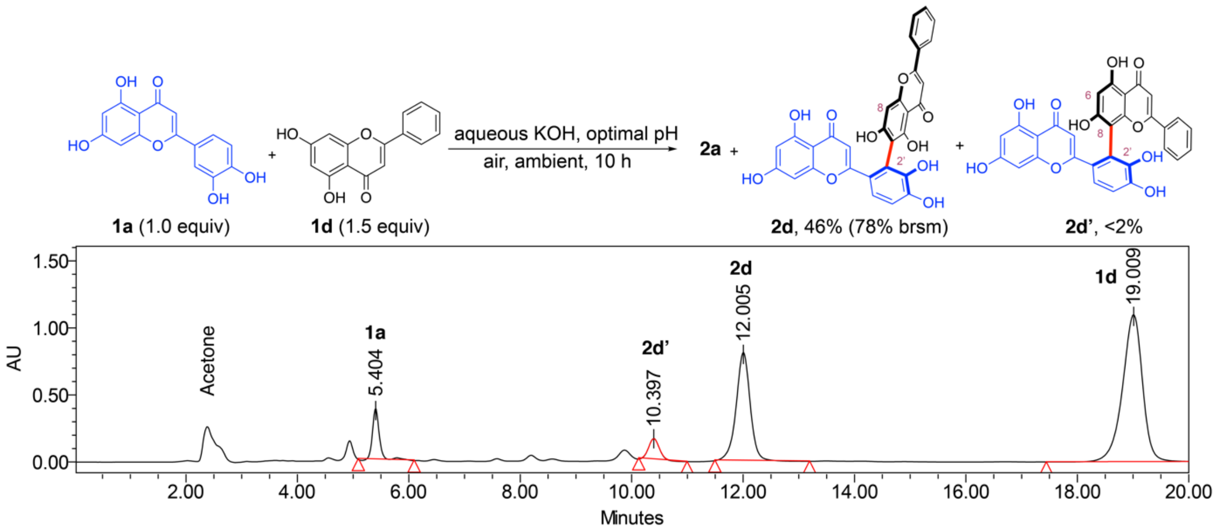

Supplementary Fig. 9. Semi-prep HPLC profile of products isolation of luteolin-chrysin cross-coupling reaction. HPLC method: using 60% A isocratic method at 5 mL/min, injection volume is 500 µL. Mobile phase A is DI water with 0.1% formic acid and mobile phase B is acetonitrile with 0.1% formic acid. The PDA detector was used and the absorbance shown was at 280 nm.

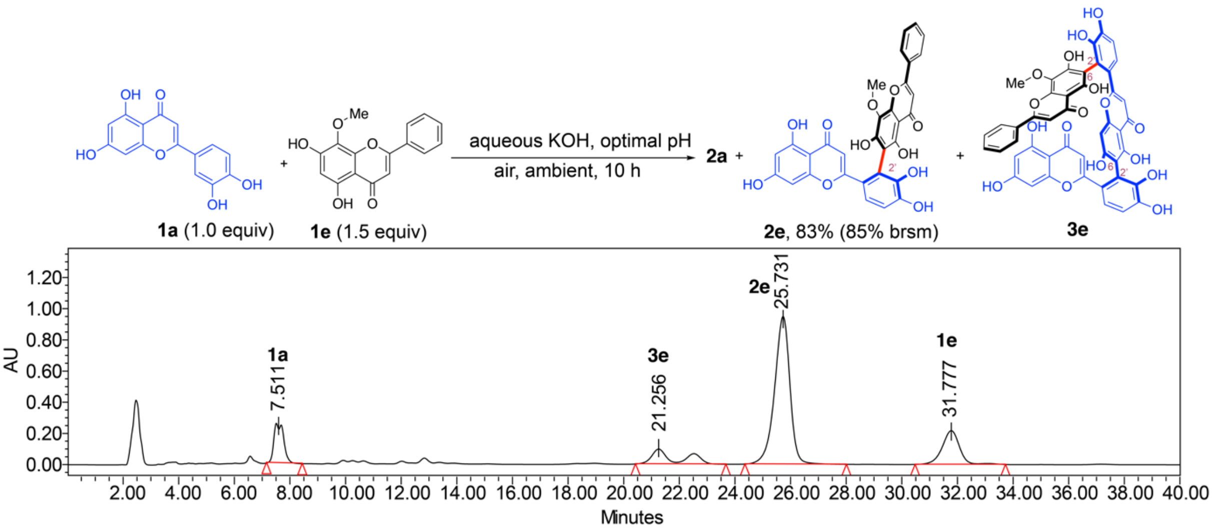

Supplementary Fig. 10. Semi-prep HPLC profile of products of luteolin-wogonin cross-coupling reaction. HPLC method: using 64% A isocratic method at 5 mL/min, injection volume is 500 µL. Mobile phase A is DI water with 0.1% formic acid and mobile phase B is acetonitrile with 0.1% formic acid. The PDA detector was used and the absorbance shown was at 280 nm.

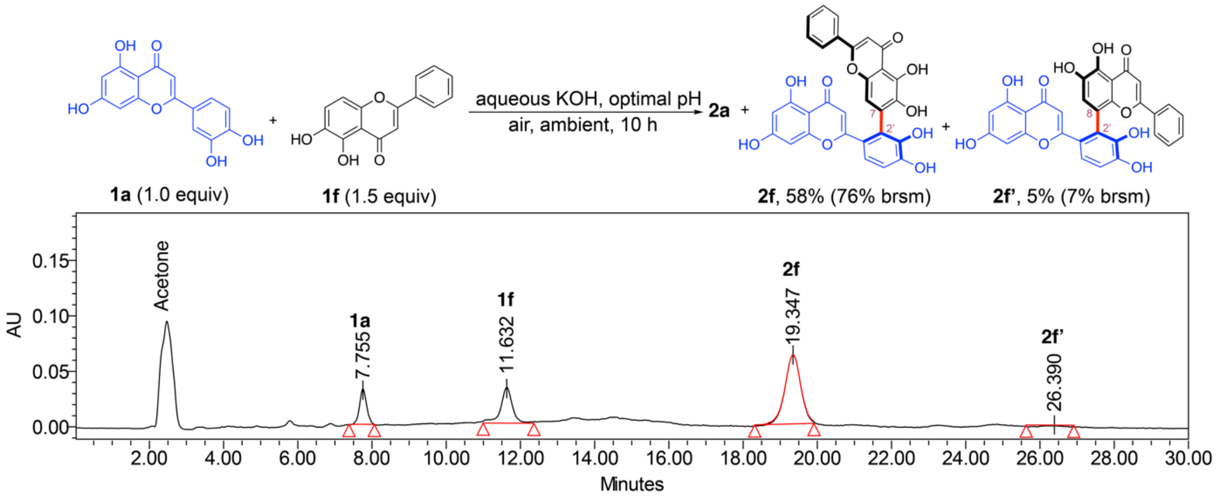

Supplementary Fig. 11. Semi-prep HPLC profile of products of luteolin-5,6-dihydroxyflavone cross-coupling reaction. HPLC method: using 64% A isocratic method at 5 mL/min, injection volume is 500 μL. Mobile phase A is DI water with 0.1% formic acid and mobile phase B is acetonitrile with 0.1% formic acid. The PDA detector was used and the absorbance shown was at 280 nm.

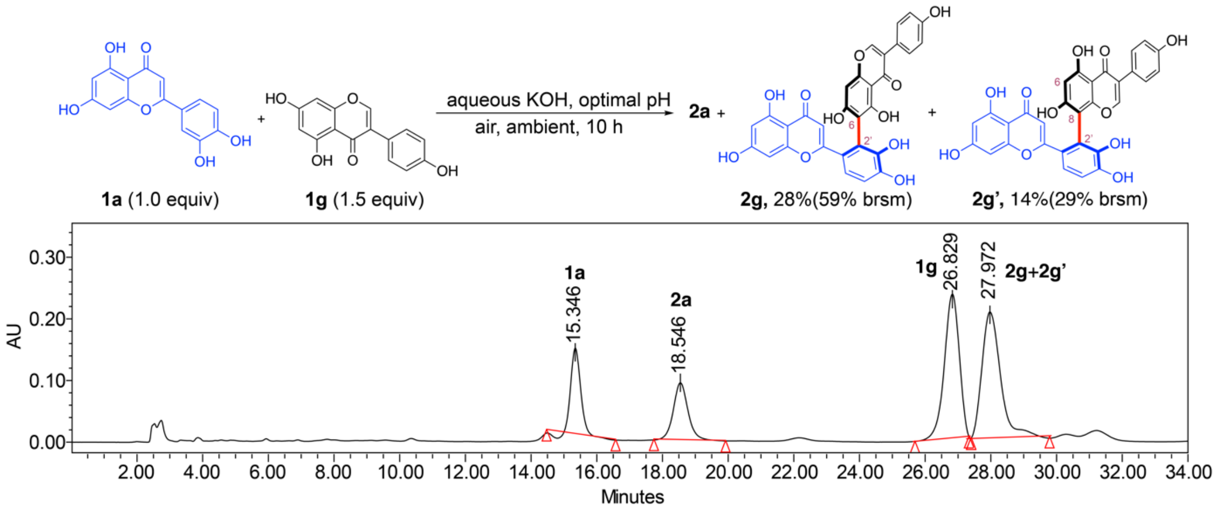

Supplementary Fig. 12. Semi-prep HPLC profile of products of luteolin-genistein cross-coupling reaction. HPLC method: using 71.5% A isocratic method at 5 mL/min, injection volume is 500 μL. Mobile phase A is DI water with 0.1% formic acid and mobile phase B is Acetonitrile with 0.1% formic acid. The PDA detector was used and the absorbance shown was at 280 nm.

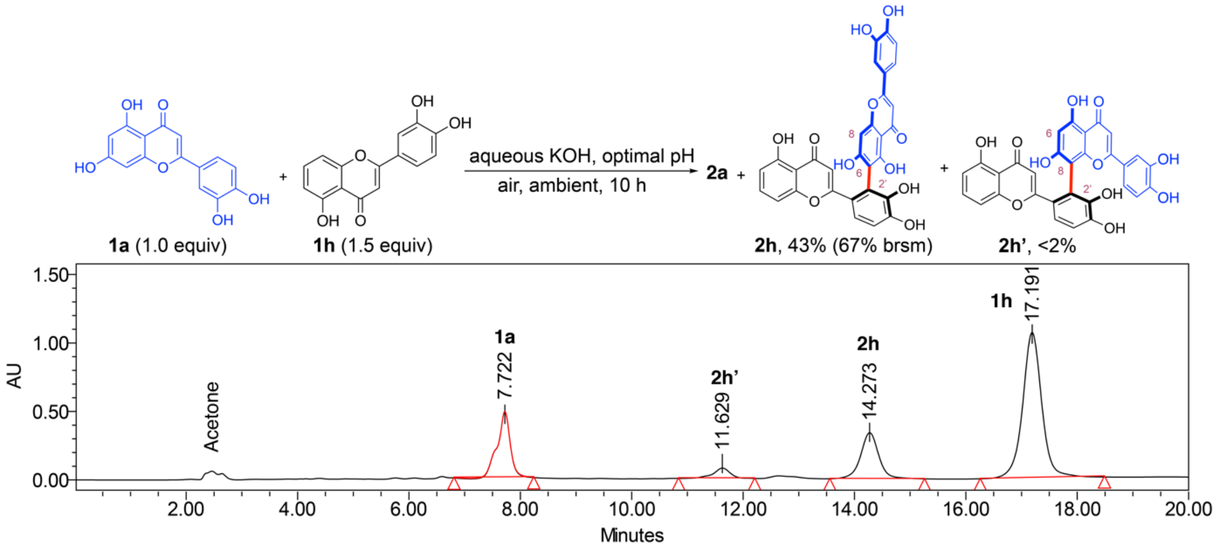

Supplementary Fig. 13. Semi-prep HPLC profile of products of luteolin-5,3′,4′-trihydroxyflavone cross-coupling reaction. HPLC method: using 64% A isocratic method at 5 mL/min, injection volume is 500 μL. Mobile phase A is DI water with 0.1% formic acid and mobile phase B is Acetonitrile with 0.1% formic acid. The PDA detector was used and the absorbance shown was at 280 nm.

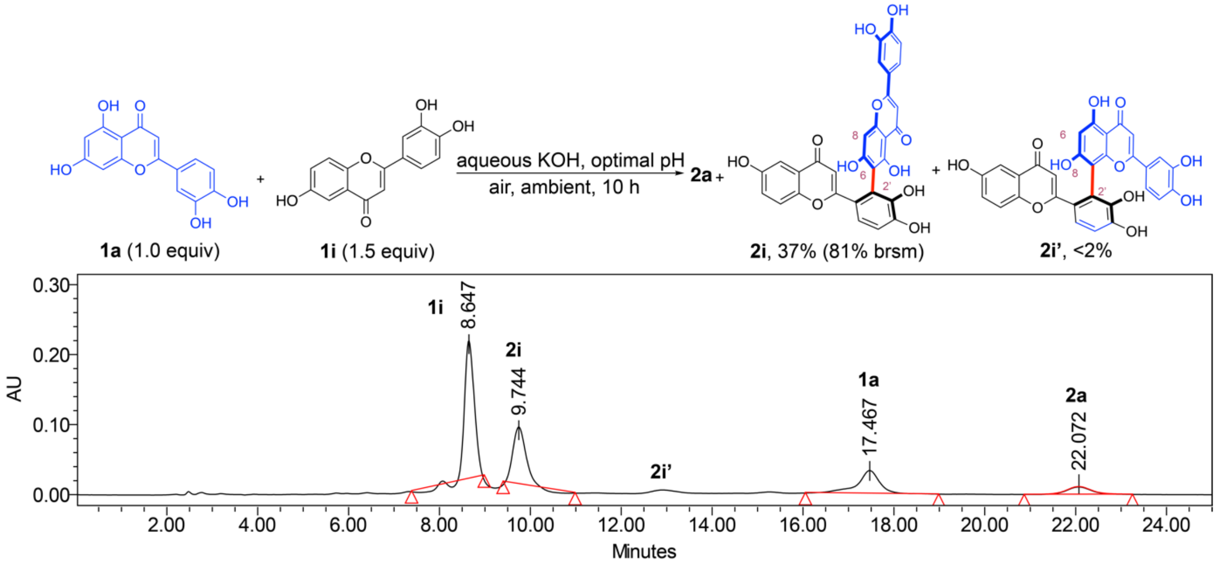

Supplementary Fig. 14. Semi-prep HPLC profile of products isolation of luteolin-6,3′,4′-trihydroxyflavone cross-coupling reaction. HPLC method: using 72.5% A isocratic method at 5 mL/min, injection volume is 500 μL. Mobile phase A is DI water with 0.1% formic acid and mobile phase B is Acetonitrile with 0.1% formic acid. The PDA detector was used and the absorbance shown was at 280 nm.

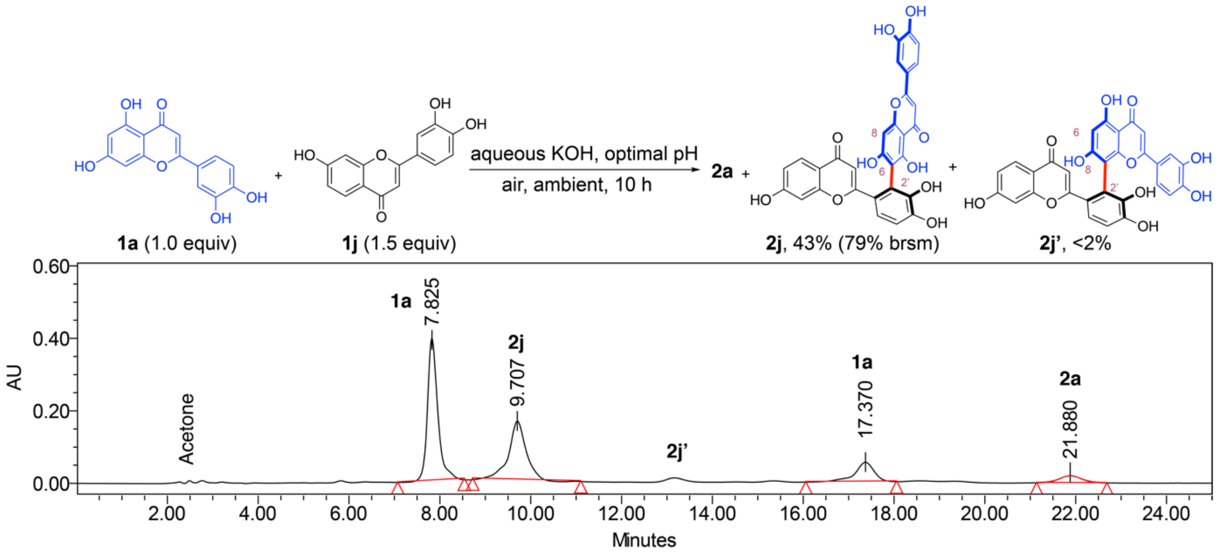

Supplementary Fig. 15. Semi-prep HPLC profile of products isolation of luteolin-7,3′,4′-trihydroxyflavone cross-coupling reaction. HPLC method: using 72.5% A isocratic method at 5 mL/min, injection volume is 500 µL. Mobile phase A is DI water with 0.1% formic acid and mobile phase B is Acetonitrile with 0.1% formic acid. The PDA detector was used and the absorbance shown was at 280 nm.

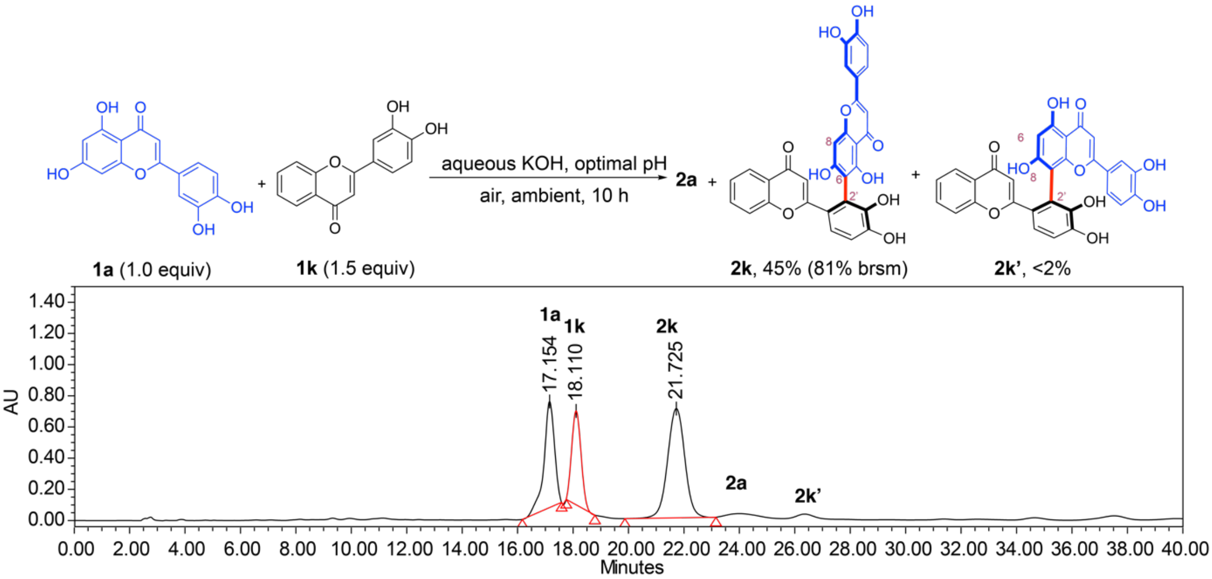

Supplementary Fig. 16. Semi-prep HPLC profile of products isolation of luteolin-3′,4′-dihydroxyflavone cross-coupling reaction. HPLC method: using 71.5% A isocratic method at 5 mL/min, injection volume is 500 μL. Mobile phase A is DI water with 0.1% formic acid and mobile phase B is acetonitrile with 0.1% formic acid. The PDA detector was used and the absorbance shown was at 280 nm.

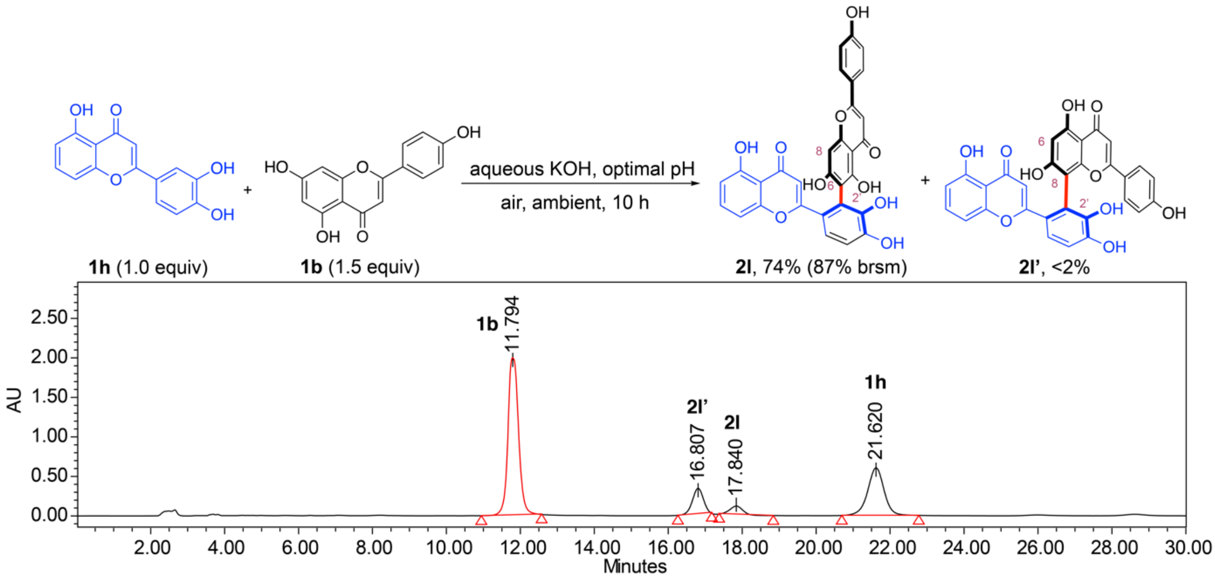

Supplementary Fig. 17. Semi-prep HPLC profile of products isolation of 5,3′,4′-trihydroxyflavone-apigenin cross-coupling reaction. HPLC method: using 64% A isocratic method at 5 mL/min, injection volume is 500 µL. Mobile phase A is DI water with 0.1% formic acid and mobile phase B is acetonitrile with 0.1% formic acid. The PDA detector was used and the absorbance shown was at 280 nm.

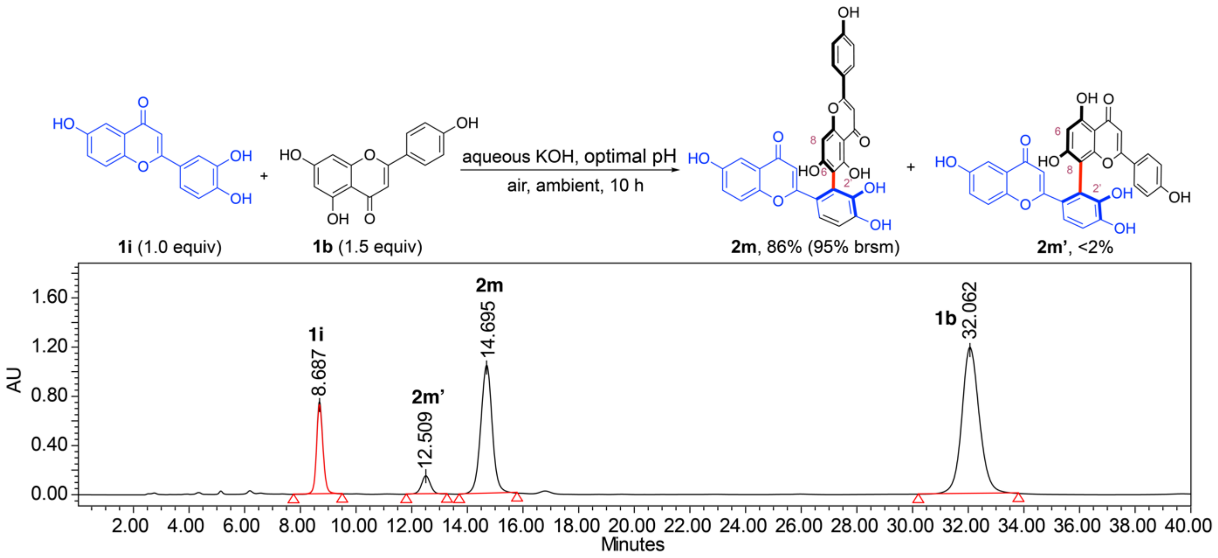

Supplementary Fig. 18. Semi-prep HPLC profile of products of 6,3′,4′-trihydroxyflavone-apigenin cross-coupling reaction. HPLC method: using 72.5% A isocratic method at 5 mL/min, injection volume is 500 μL. Mobile phase A is DI water with 0.1% formic acid and mobile phase B is acetonitrile with 0.1% formic acid. From left to right, the products were identified as **2m′** at 12.509 min and **2m** at 14.695 min. The PDA detector was used and the absorbance shown was at 280 nm.

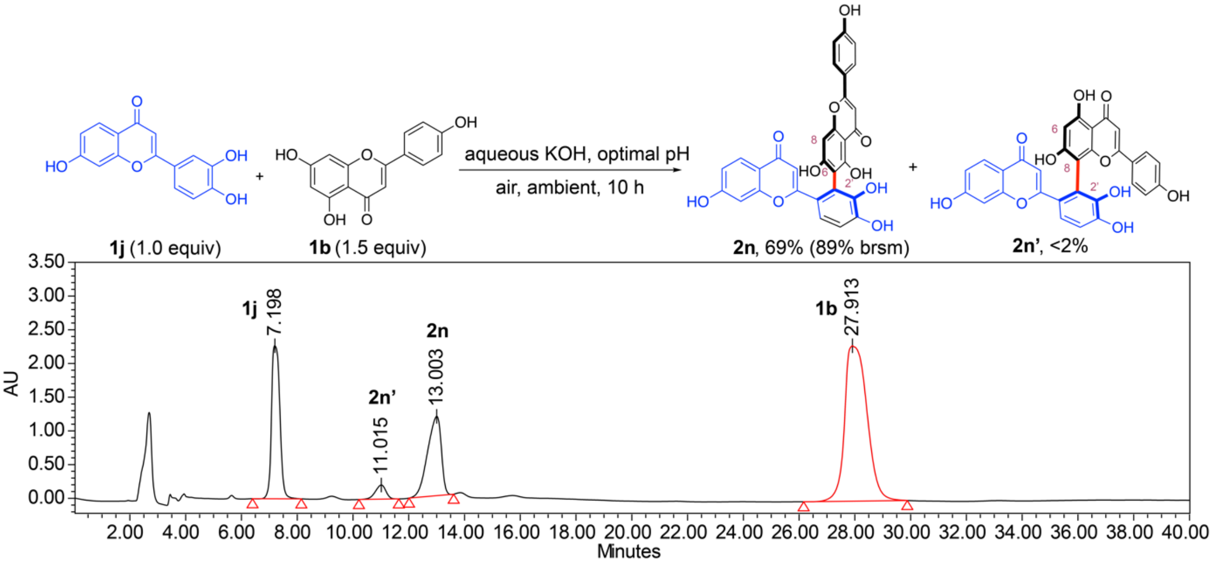

Supplementary Fig. 19. Semi-prep HPLC profile of products of 7,3′,4′-trihydroxyflavone-apigenin cross-coupling reaction. HPLC method: using 72.5% A isocratic method at 5 mL/min, injection volume is 500 µL. Mobile phase A is DI water with 0.1% formic acid and mobile phase B is acetonitrile with 0.1% formic acid. The PDA detector was used and the absorbance shown was at 280 nm.

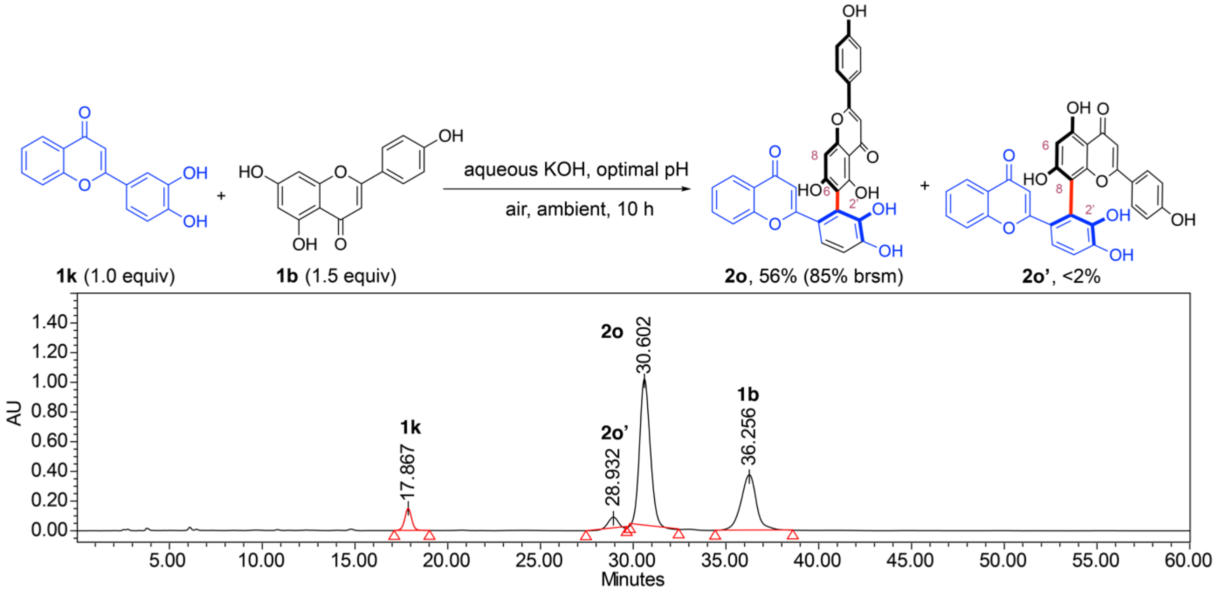

Supplementary Fig. 20. Semi-prep HPLC profile of products of 3′,4′-dihydroxyflavone-apigenin cross-coupling reaction. HPLC method: using 71.5% A isocratic method at 5 mL/min, injection volume is 500 µL. Mobile phase A is DI water with 0.1% formic acid and mobile phase B is acetonitrile with 0.1% formic acid. The PDA detector was used and the absorbance shown was at 280 nm.

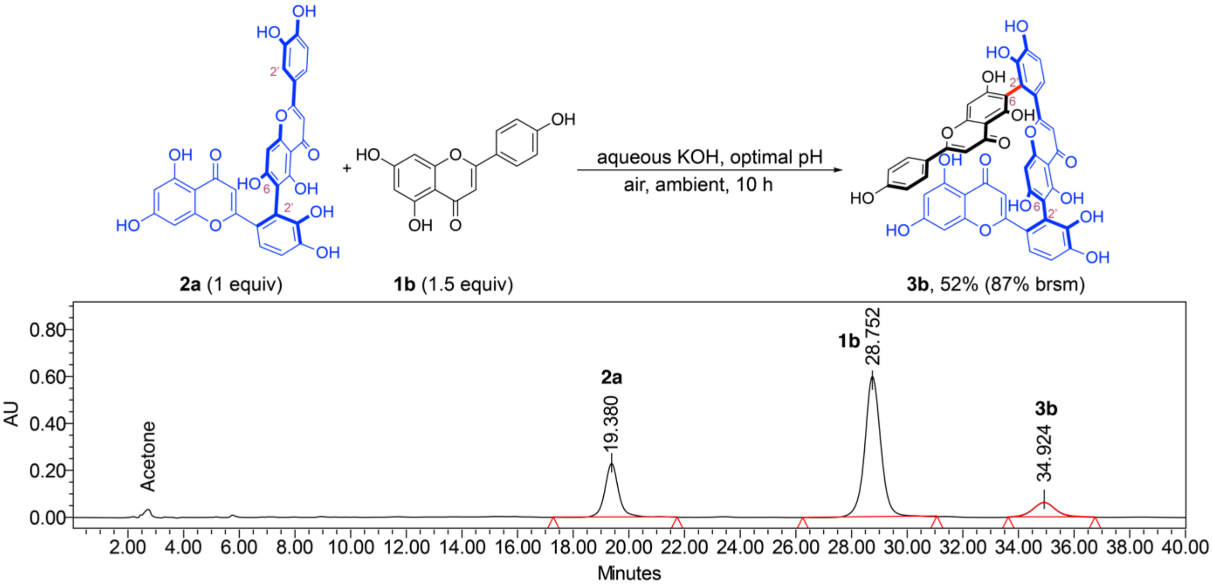

Supplementary Fig. 21. Semi-prep HPLC profile of products of dicranolomin-apigenin cross-coupling reaction. HPLC method: using 71.5% A isocratic method at 5 mL/min, injection volume is 500 µL. Mobile phase A is DI water with 0.1% formic acid and mobile phase B is acetonitrile with 0.1% formic acid. The PDA detector was used and the absorbance shown was at 280 nm.

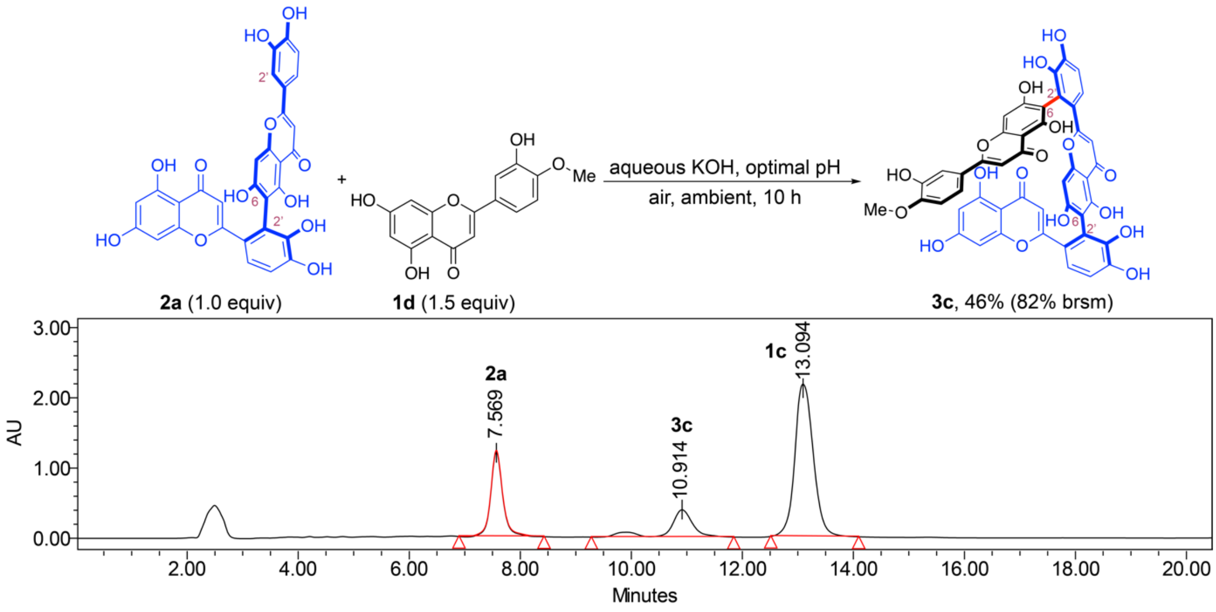

Supplementary Fig. 22. Semi-prep HPLC profile of products of dicranolomin-diosmetin cross-coupling reaction. HPLC method: using 64% A isocratic method at 5 mL/min, injection volume is 500 µL. Mobile phase A is DI water with 0.1% formic acid and mobile phase B is acetonitrile with 0.1% formic acid. The PDA detector was used and the absorbance shown was at 280 nm.

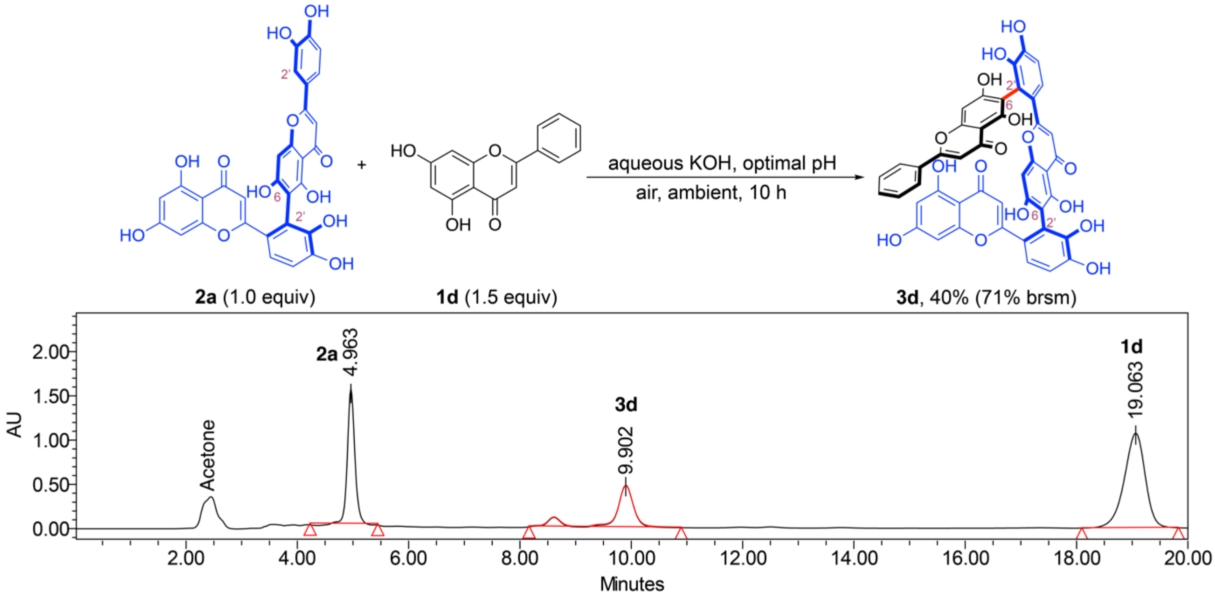

Supplementary Fig. 23. Semi-prep HPLC profile of products of dicranolomin-chrysin cross-coupling reaction. HPLC method: using 60% A isocratic method at 5 mL/min, injection volume is 500 µL. Mobile phase A is DI water with 0.1% formic acid and mobile phase B is acetonitrile with 0.1% formic acid. The PDA detector was used and the absorbance shown was at 280 nm.

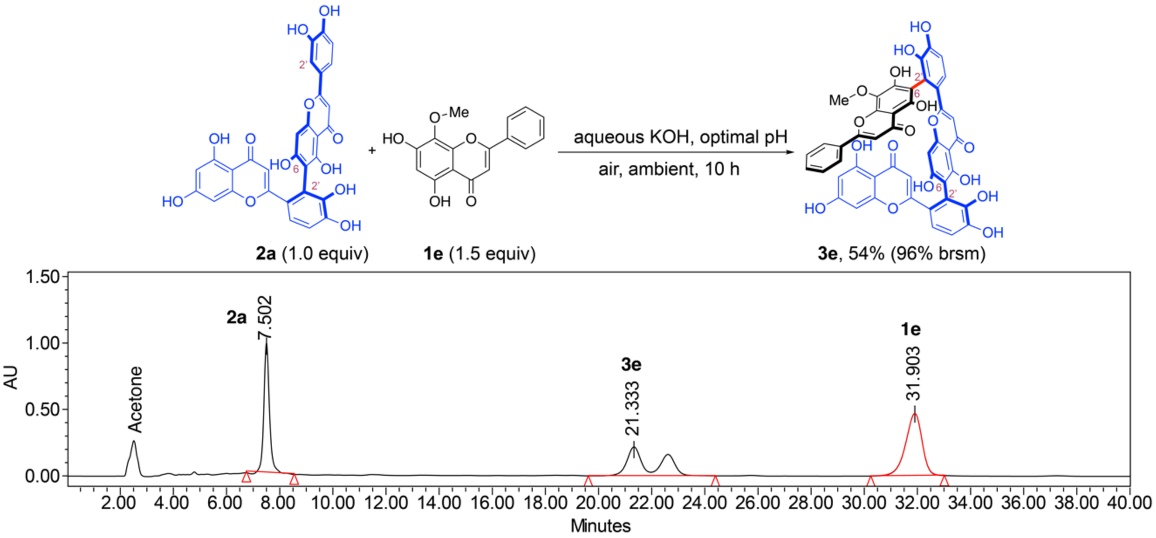

Supplementary Fig. 24. Semi-prep HPLC profile of products of dicranolomin-wogonin cross-coupling reaction. HPLC method: using 64% A isocratic method at 5 mL/min, injection volume is 500 µL. Mobile phase A is DI water with 0.1% formic acid and mobile phase B is acetonitrile with 0.1% formic acid. The PDA detector was used and the absorbance shown was at 280 nm.

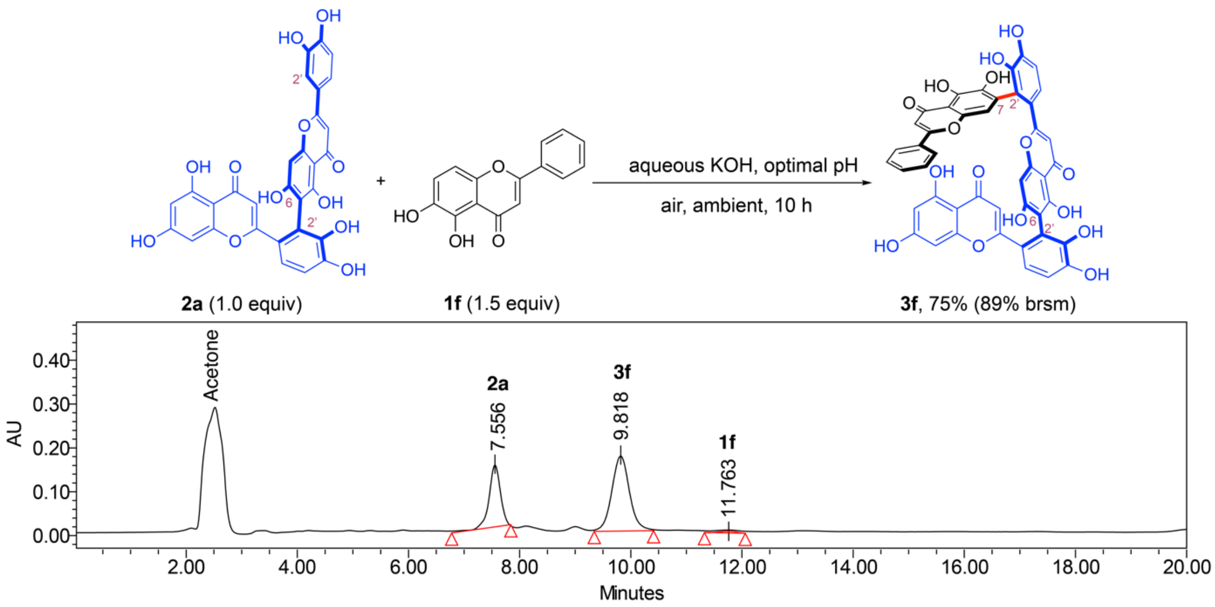

Supplementary Fig. 25. Semi-prep HPLC profile of products of dicranolomin-5,6 dihydroxyflavone cross-coupling reaction. HPLC method: using 64% A isocratic method at 5 mL/min, injection volume is 500 µL. Mobile phase A is DI water with 0.1% formic acid and mobile phase B is Acetonitrile with 0.1% formic acid. The PDA detector was used and the absorbance shown was at 280 nm.

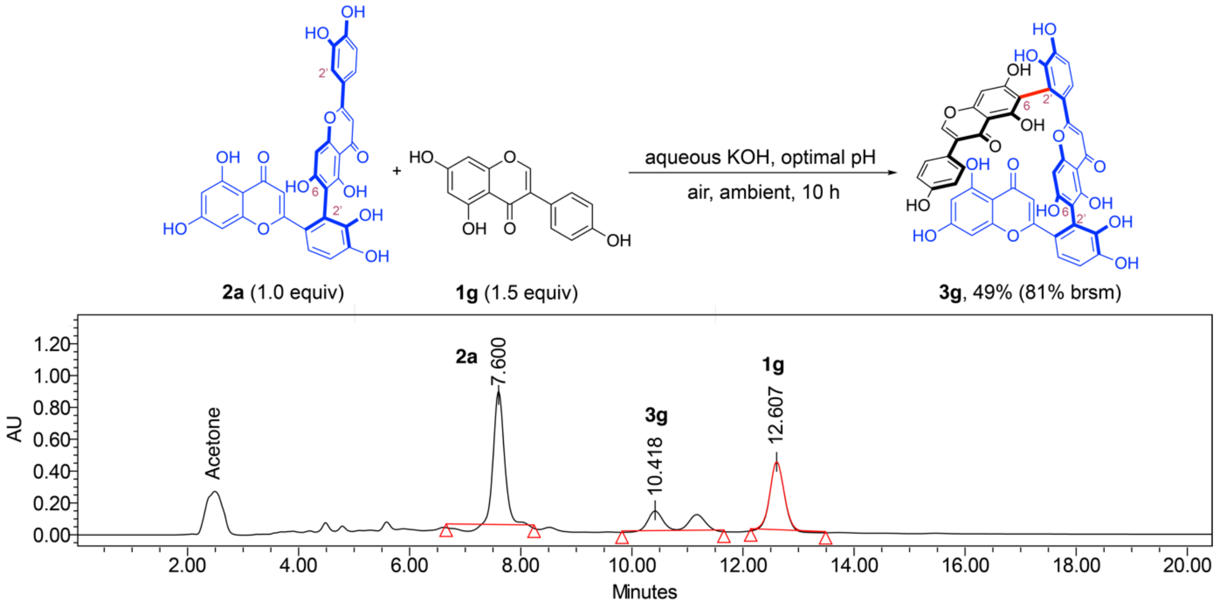

Supplementary Fig. 26. Semi-prep HPLC profile of products of dicranolomin-genistein cross-coupling reaction. HPLC method: using 64% A isocratic method at 5 mL/min, injection volume is 500 µL. Mobile phase A is DI water with 0.1% formic acid and mobile phase B is Acetonitrile with 0.1% formic acid. The PDA detector was used and the absorbance shown was at 280 nm.

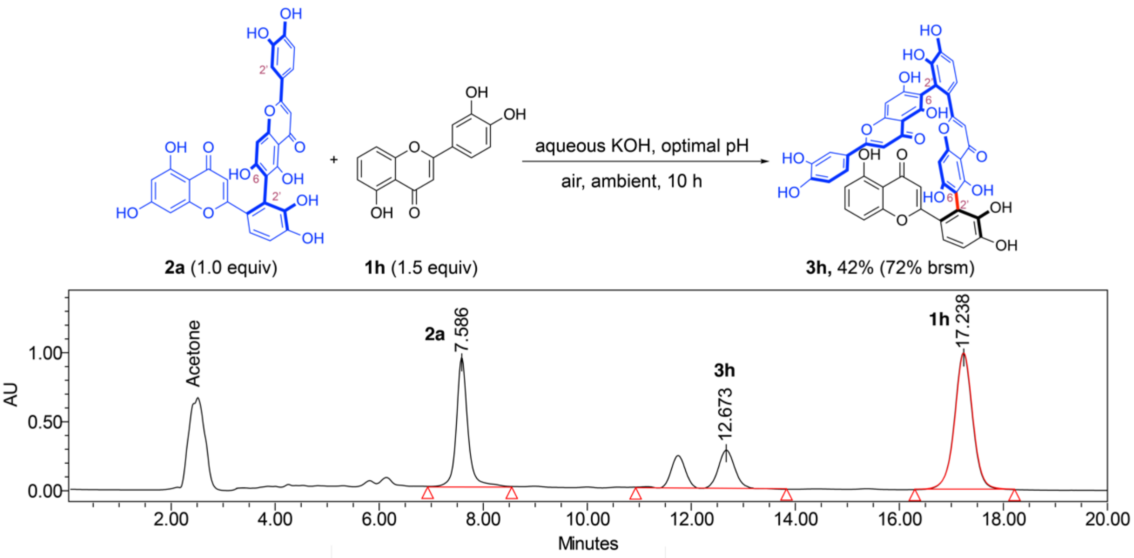

Supplementary Fig. 27. Semi-prep HPLC profile of products of 5,3′,4′-trihydroxyflavone-dicranolomin cross-coupling reaction. HPLC method: using 64% A isocratic method at 5 mL/min, injection volume is 500 µL. Mobile phase A is DI water with 0.1% formic acid and mobile phase B is acetonitrile with 0.1% formic acid. The PDA detector was used and the absorbance shown was at 280 nm.

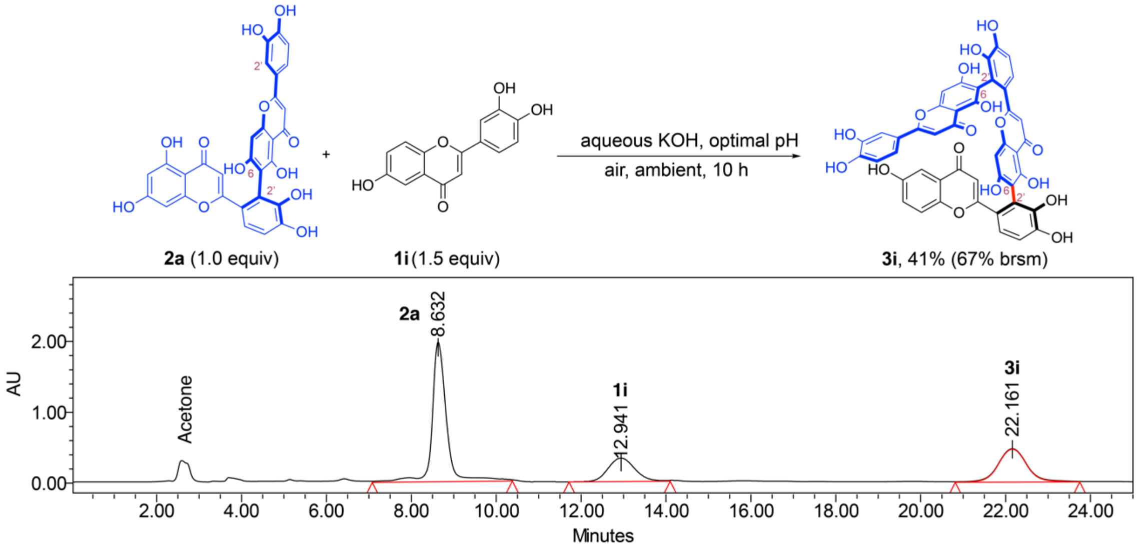

Supplementary Fig. 28. Semi-prep HPLC profile of products of 6,3′,4′-trihydroxyflavone-dicranolomin cross-coupling reaction. HPLC method: using 72.5% A isocratic method at 5 mL/min, injection volume is 500 µL. Mobile phase A is DI water with 0.1% formic acid and mobile phase B is Acetonitrile with 0.1% formic acid. The PDA detector was used and the absorbance shown was at 280 nm.

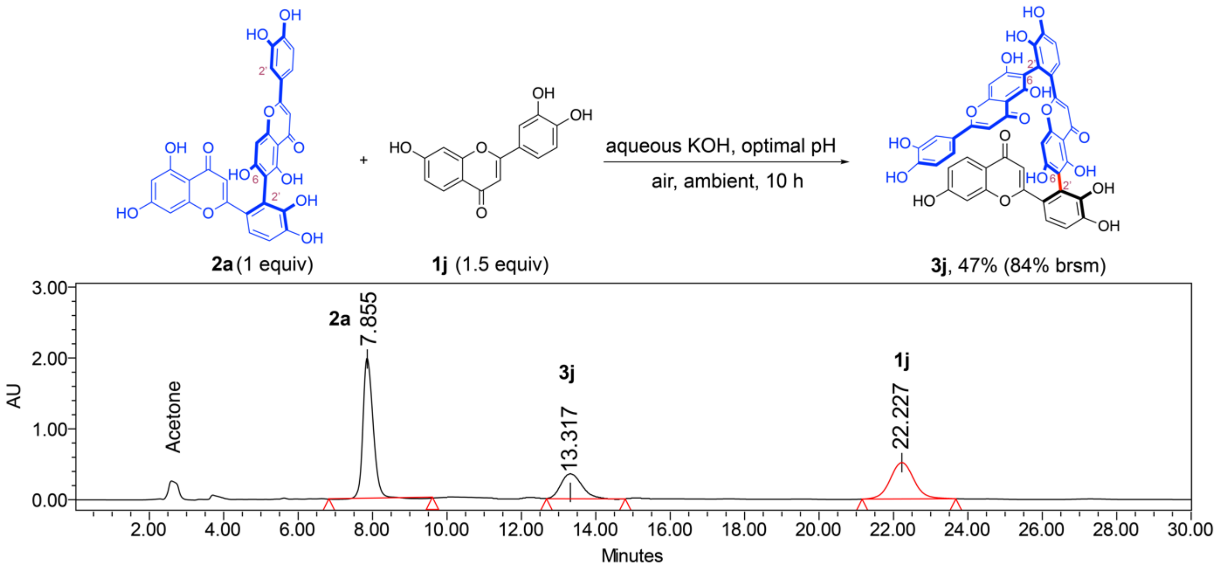

Supplementary Fig. 29. Semi-prep HPLC profile of products isolation of 7,3′,4′-trihydroxyflavone-dicranolomin cross-coupling reaction. HPLC method: using 72.5% A isocratic method at 5 mL/min, injection volume is 500 μL. Mobile phase A is DI water with 0.1% formic acid and mobile phase B is acetonitrile with 0.1% formic acid. The PDA detector was used and the absorbance shown was at 280 nm.

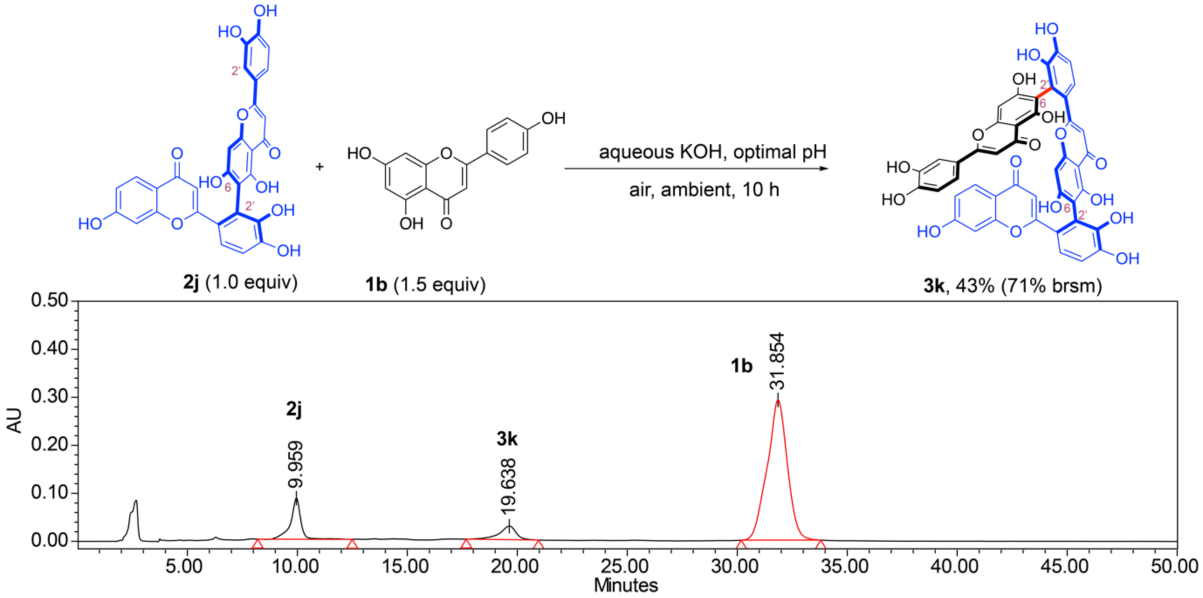

Supplementary Fig. 30. Semi-prep HPLC profile of products of 2j-apigenin cross-coupling reaction. HPLC method: using 71.5% A isocratic method at 5 mL/min, injection volume is 500 μL. Mobile phase A is DI water with 0.1% formic acid and mobile phase B is acetonitrile with 0.1% formic acid. The PDA detector was used and the absorbance shown was at 280 nm.

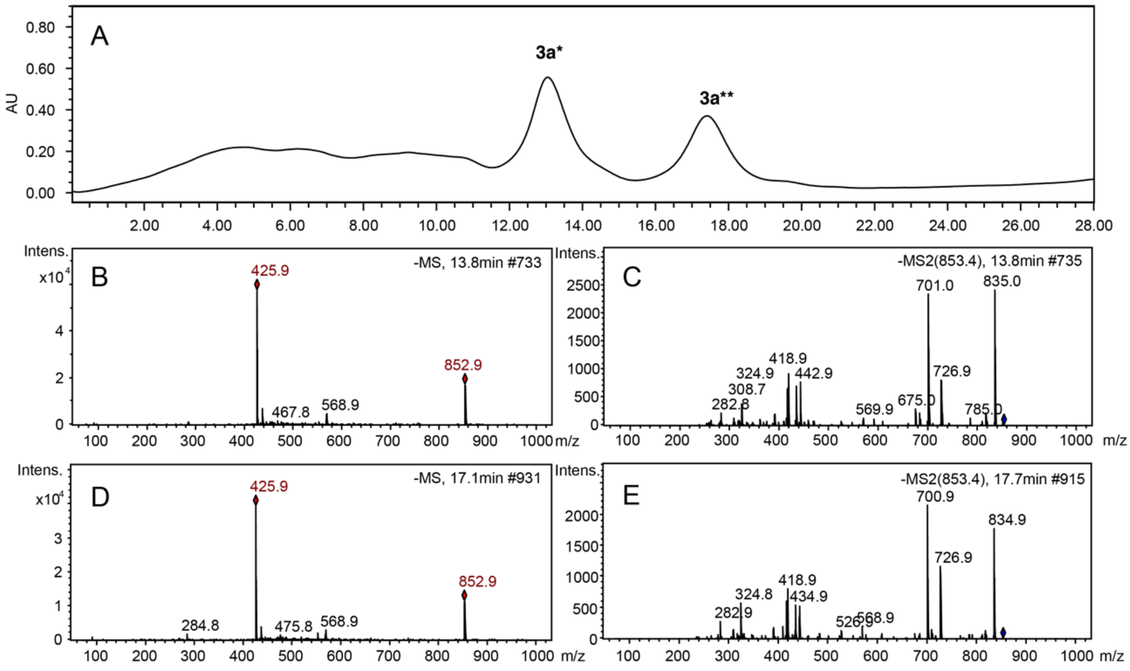

Supplementary Fig. 31. LC-MS spectra of trimer 3a and its atropisomers. A) The HPLC profile of trimer **3a**, atropisomers **3a*** and **3a**** were separated at retention time of 13.12 min and 17.39 min respectively. HPLC method: using 65% A isocratic method at 1.0 mL/min, injection volume is 50 µL. Mobile phase A is DI water with 0.1% formic acid and mobile phase B is Acetonitrile with 0.1% formic acid, tr (**3a***) = 13.12 minutes, tr (**3a****) = 17.39 minutes. Detector wavelength was at 280 nm. B) and C) are the LCMS and LCMSMS of trimer **3a***, while D) and E) are of trimer **3a****.

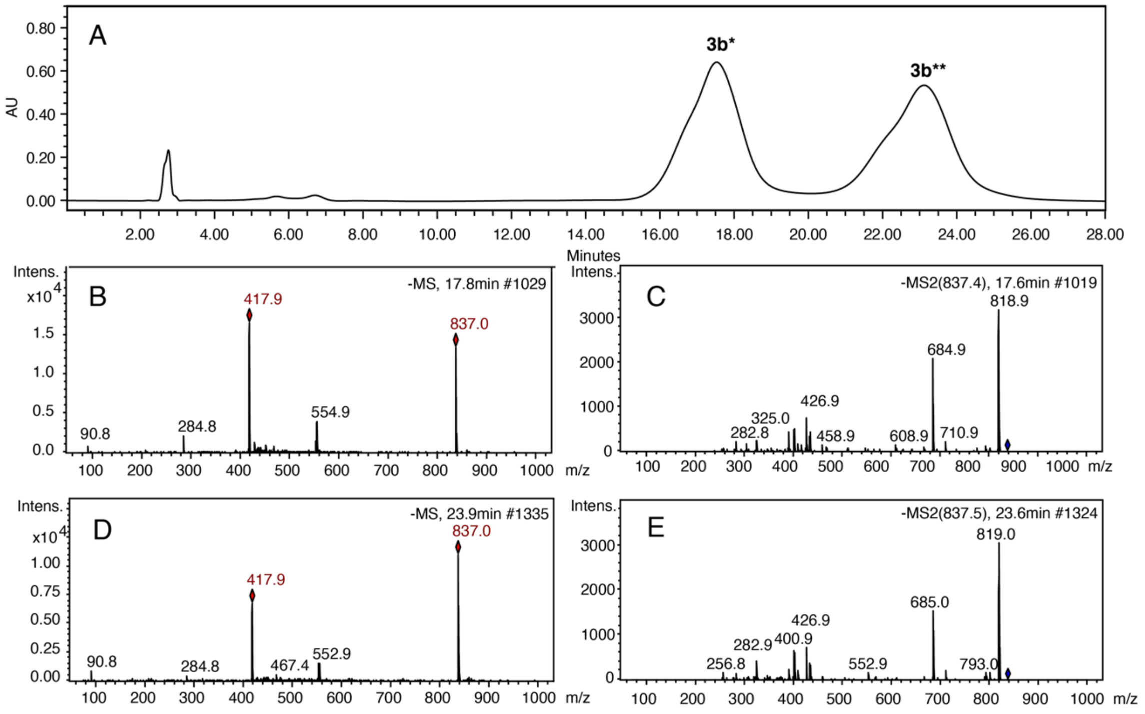

Supplementary Fig. 32. LC-MS spectra trimer 3b and its atropisomers. A) The HPLC profile of trimer **3b**, atropisomers **3b*** and **3b**** were separated at retention time of 17.32 min and 23.15 min respectively. HPLC method: using 65% A isocratic method at 1.0 mL/min, injection volume is 50 μL. Mobile phase A is DI water with 0.1% formic acid and mobile phase B is Acetonitrile with 0.1% formic acid, tr (**3b***) = 17.32 minutes, tr (**3b****) = 23.15 minutes. Detector wavelength was at 280 nm. B) and C) are the LCMS and LCMSMS of trimer **3b***, while D) and E) are of trimer **3b****.

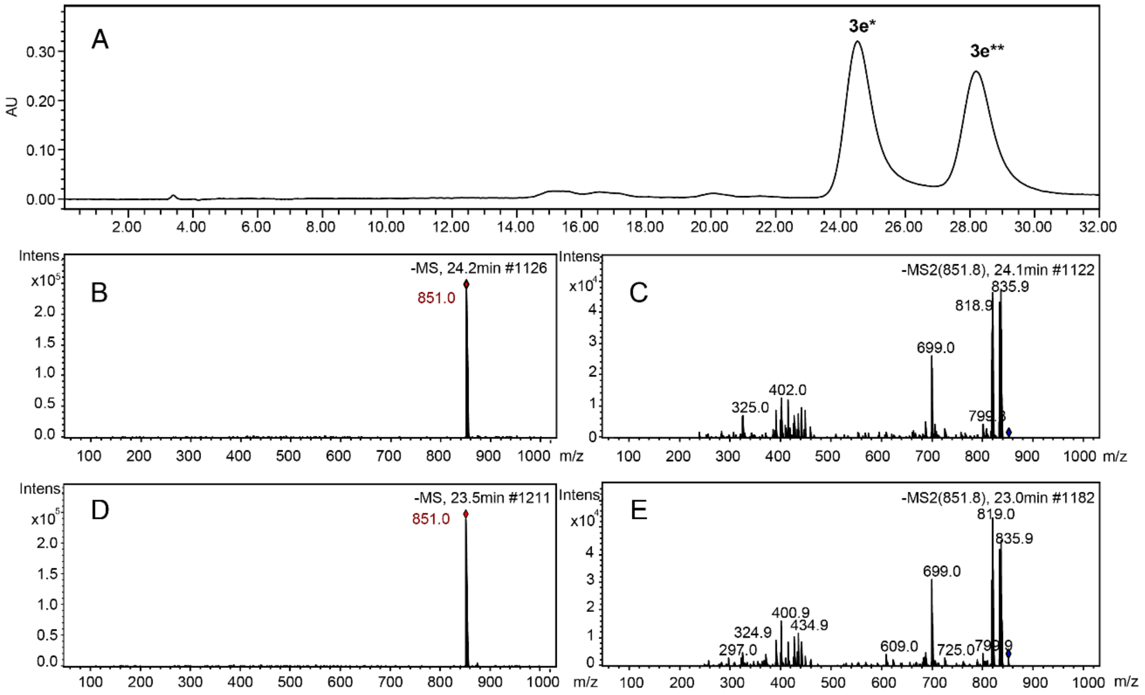

Supplementary Fig. 33. Identification of trimer 3e and its atropisomers. A) The HPLC profile of trimer **3e**, atropisomers **3e*** and **3e**** were separated at retention time of 24.79 min and 28.36 min respectively. HPLC method: using 60% A isocratic method at 1.0 mL/min, injection volume is 50 μL. Mobile phase A is DI water with 0.1% formic acid and mobile phase B is Acetonitrile with 0.1% formic acid, tr (**3e***) = 24.79 minutes, tr (**3e****) = 28.36 minutes. Detector wavelength was at 280 nm. B) and C) are the LCMS and LCMSMS of trimer **3e***, while D) and E) are of trimer **3e****.

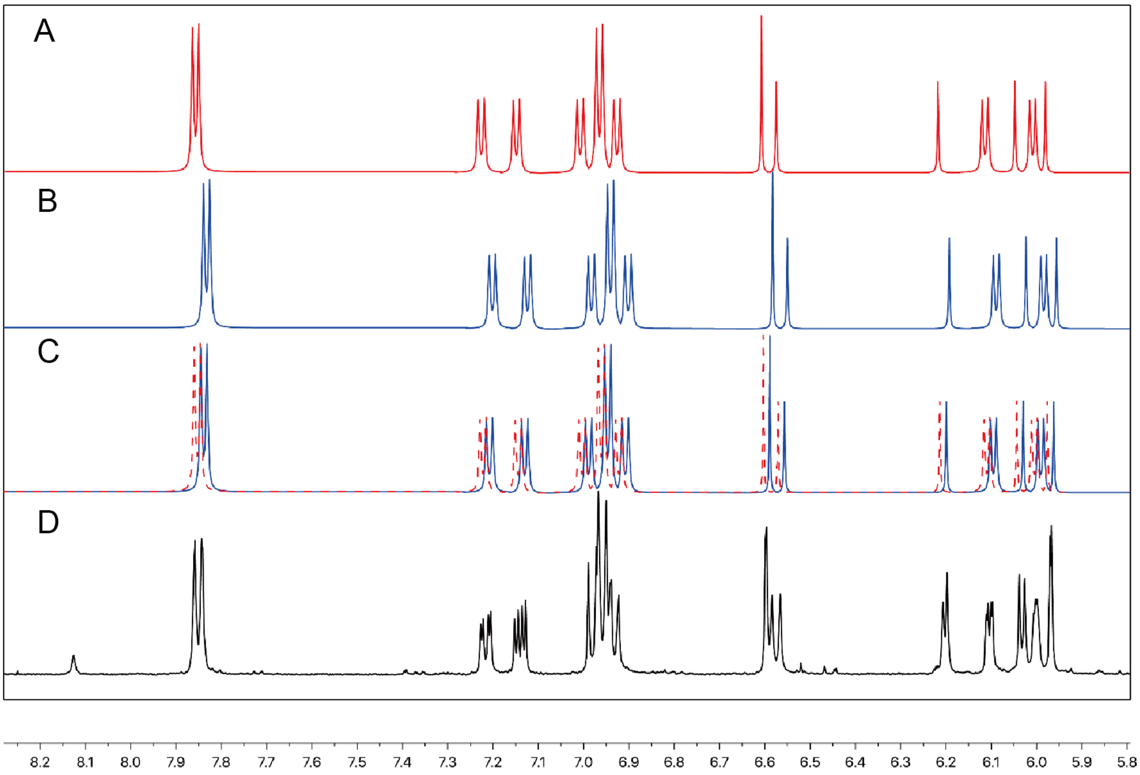

Supplementary Fig. 34. The simulated and experimental ^{1}H NMR spectra of atropisomers 3b* and 3b** for Lu_2Ap (3b). A) Simulated ^{1}H NMR spectrum of trimer **3b*****, B)** Simulation of trimer **3b******, C)** Simulated ^{1}H NMR spectrum of trimer **3b*** **and 3b******, D)** experimental ^{1}H NMR spectrum of trimer **3b*** **and 3b****.

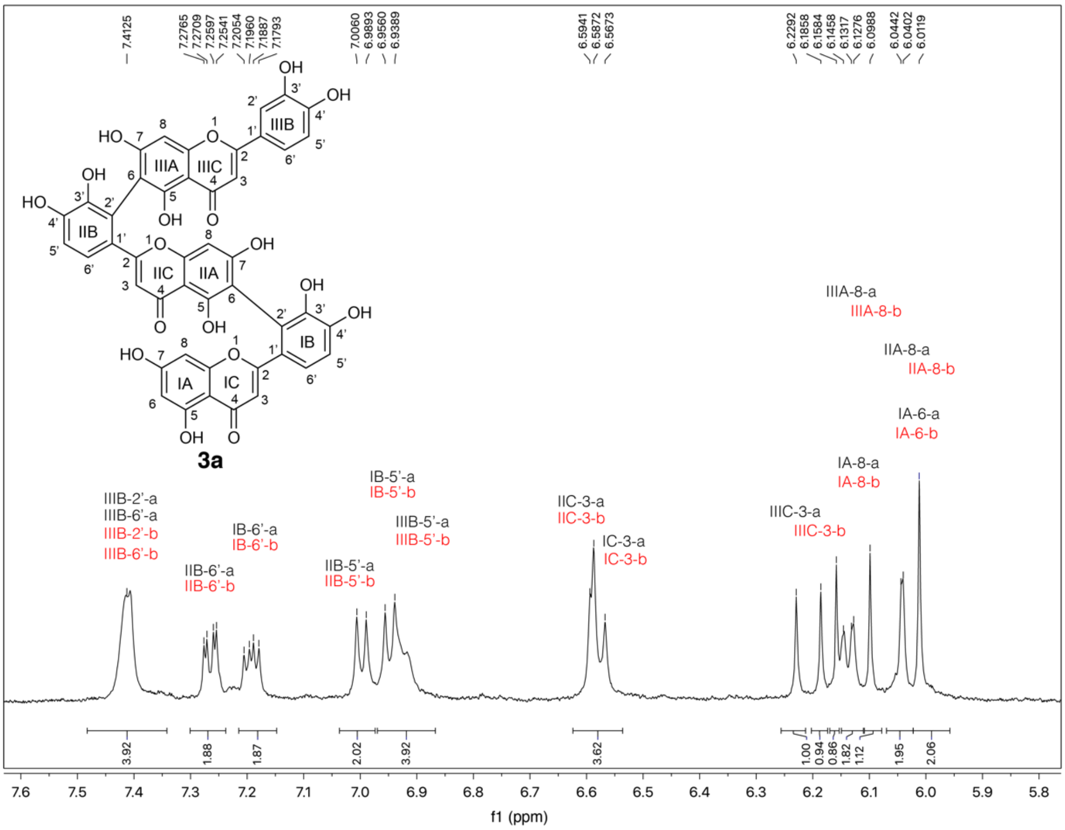

Supplementary Fig. 35. The assignment of the ^{1}H NMR spectral peaks of distichumtriluteolin (3a) atropisomers.

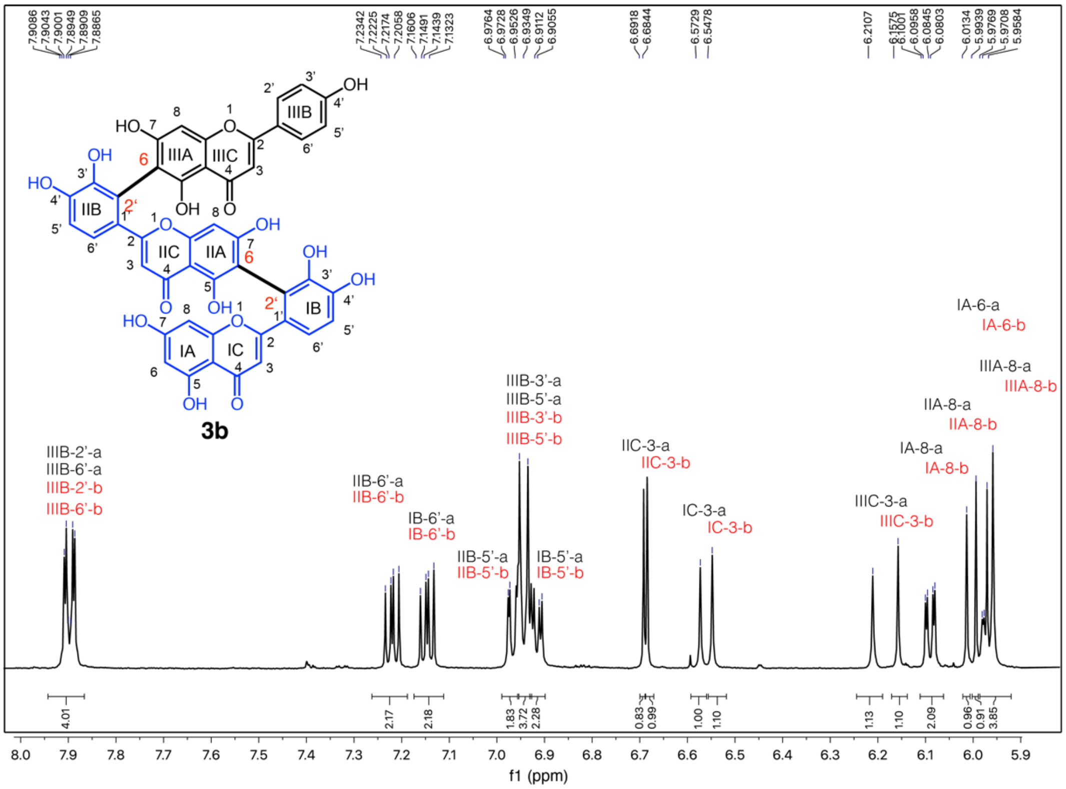

Supplementary Fig. 36. The assignment of ^{1}H NMR spectral peaks of 3b atropisomers.

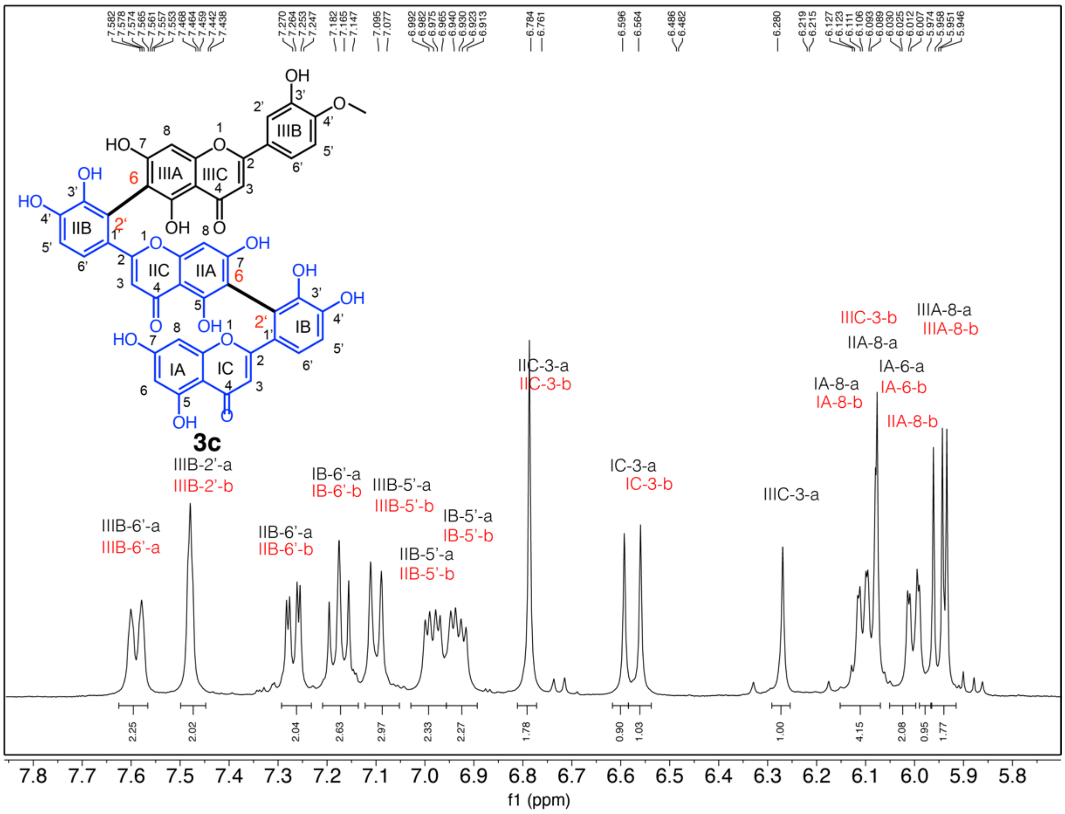

Supplementary Fig. 37. The assignment of the ^{1}H NMR spectral peaks of 3c atropisomers.

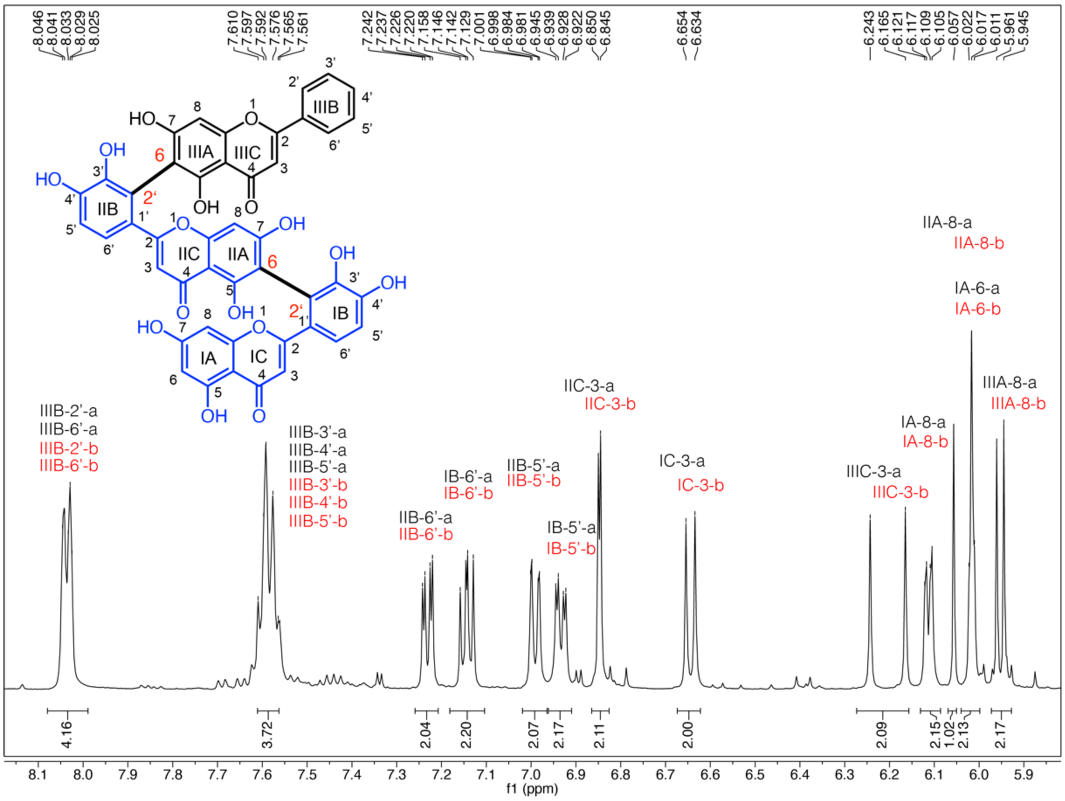

Supplementary Fig. 38. The assignment of the ^{1}H NMR spectral peaks of 3d atropisomers.

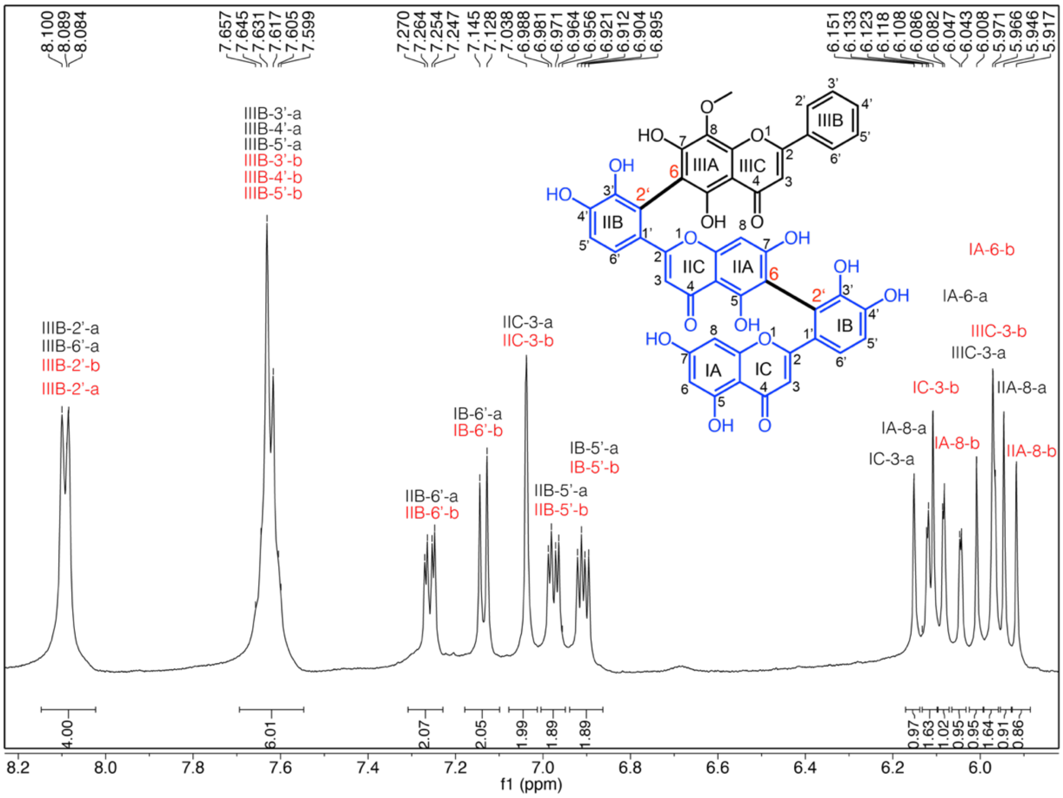

Supplementary Fig. 39. The assignment of the ^{1}H NMR spectral peaks of trimer 3e atropisomers.

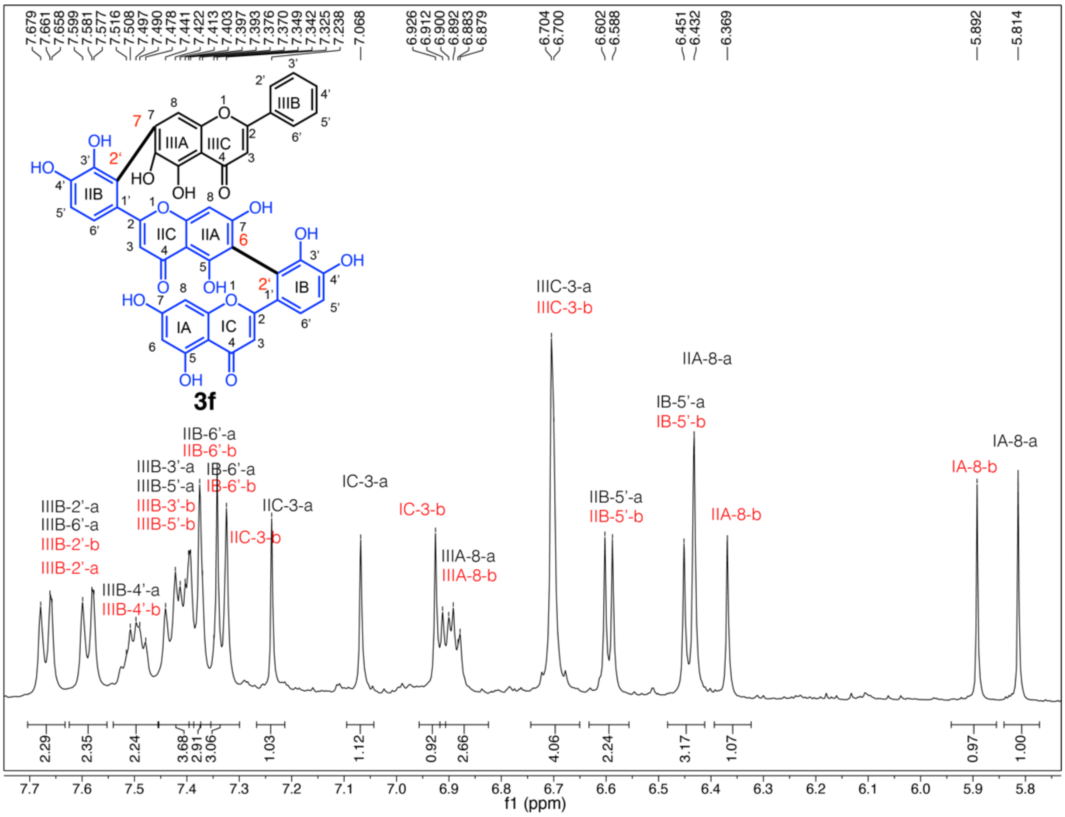

Supplementary Fig. 40. The assignment of the ^{1}H NMR spectral peaks of trimer 3f atropisomers.

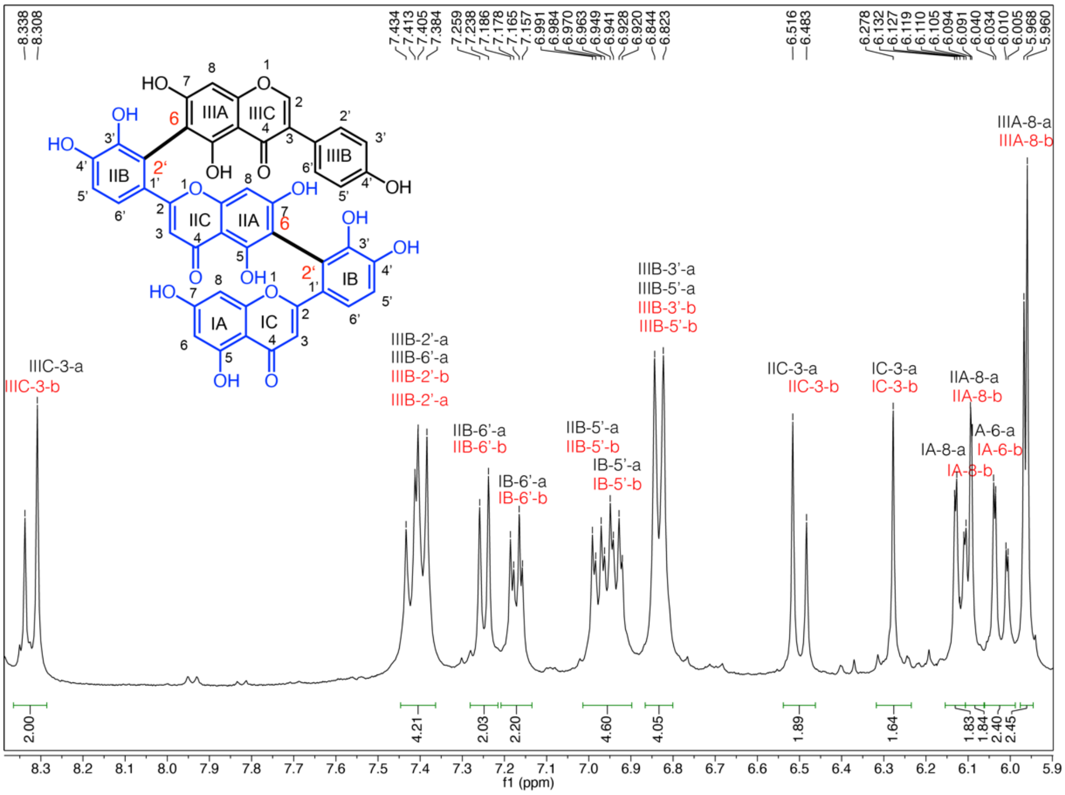

Supplementary Fig. 41. The assignment of the ^{1}H NMR spectral peaks of 3g atropisomers.

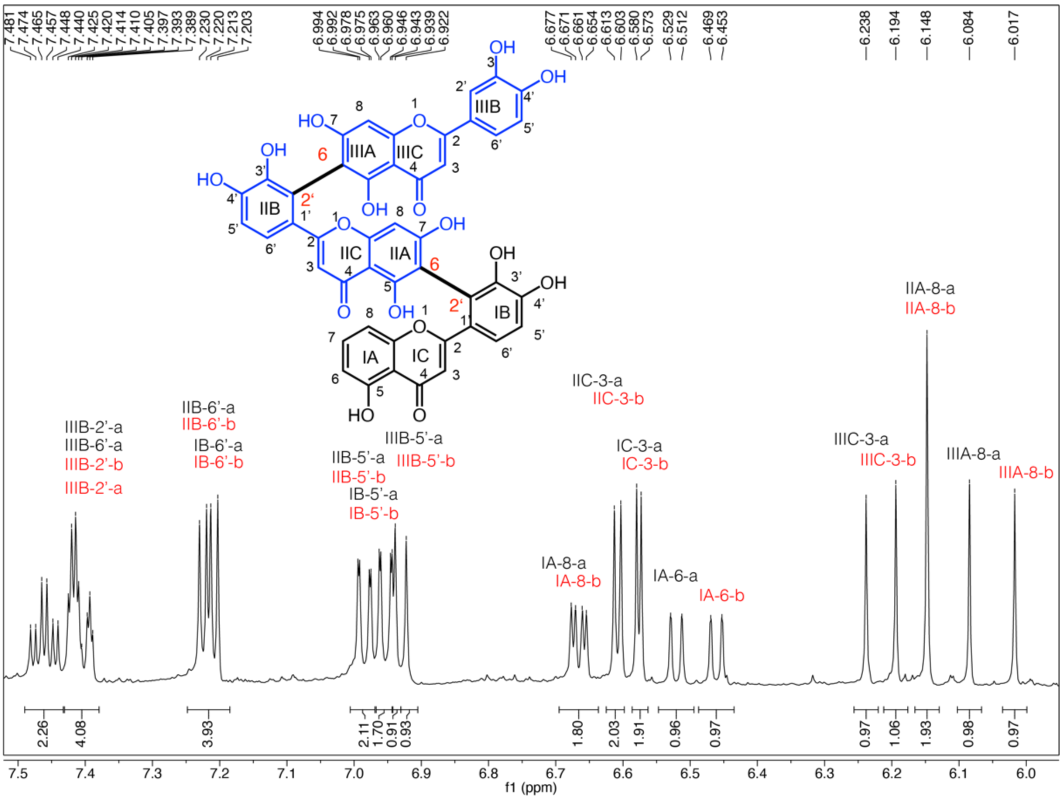

Supplementary Fig. 42. The assignment of the ^{1}H NMR spectral peaks of 3h atropisomers.

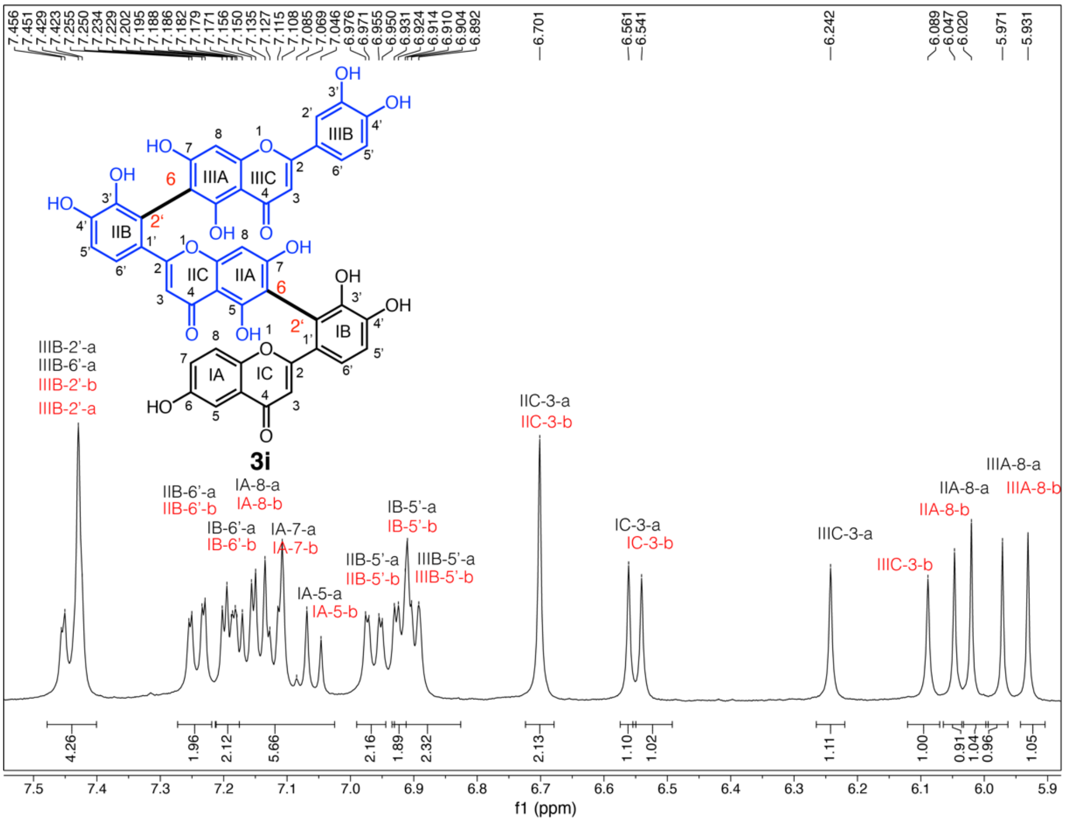

Supplementary Fig. 43. The assignment of the ^{1}H NMR spectral peaks of 3i atropisomers.

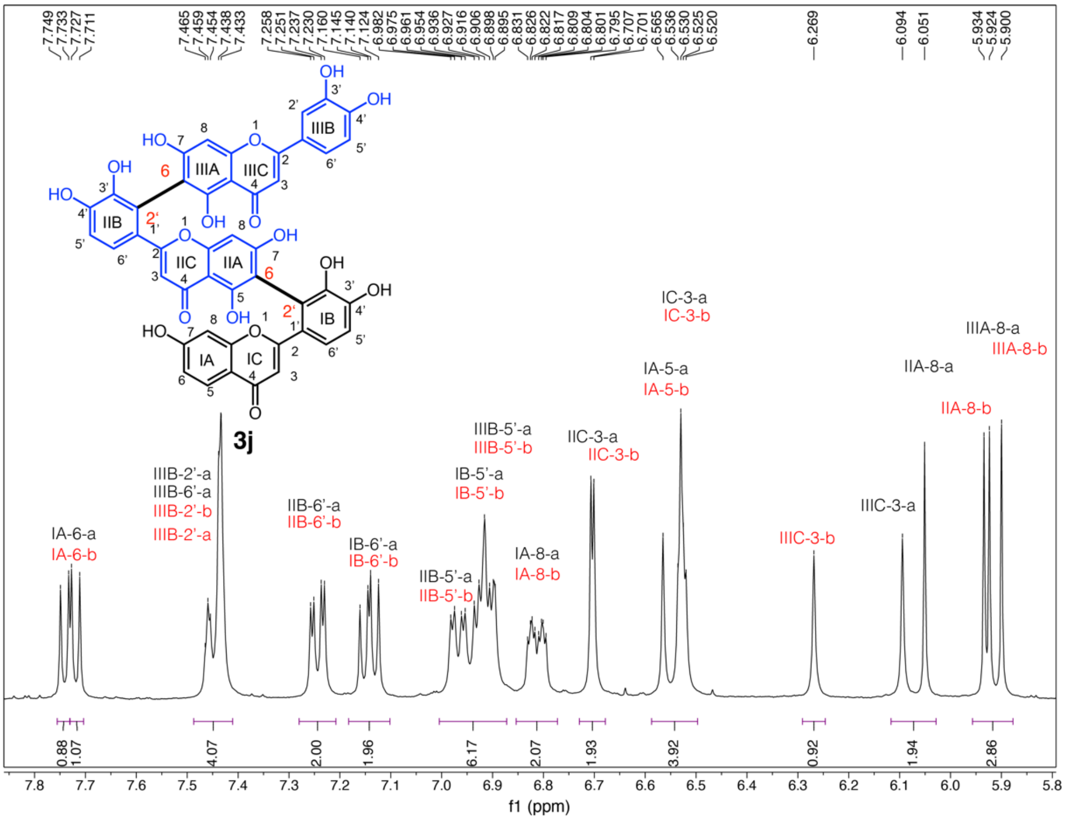

Supplementary Fig. 44. The assignment of the ^{1}H NMR spectral peaks of 3j atropisomers.

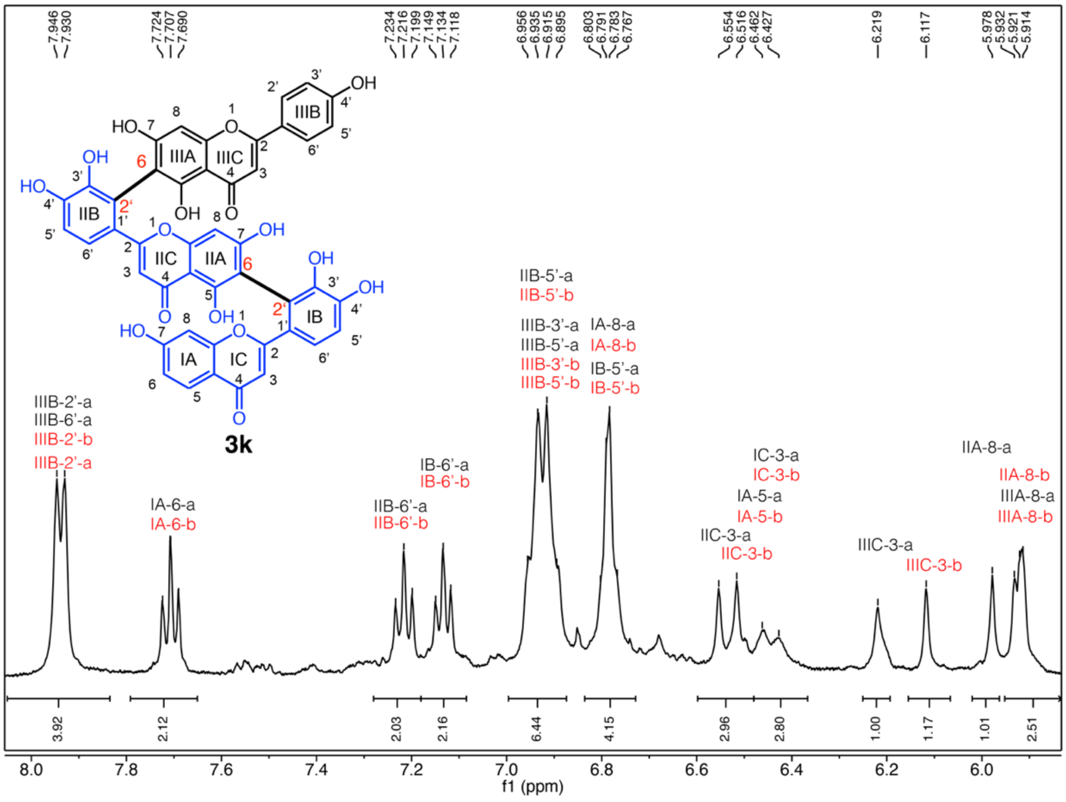

Supplementary Fig. 45. The assignment of the ^{1}H NMR spectral peaks of 3k atropisomers.

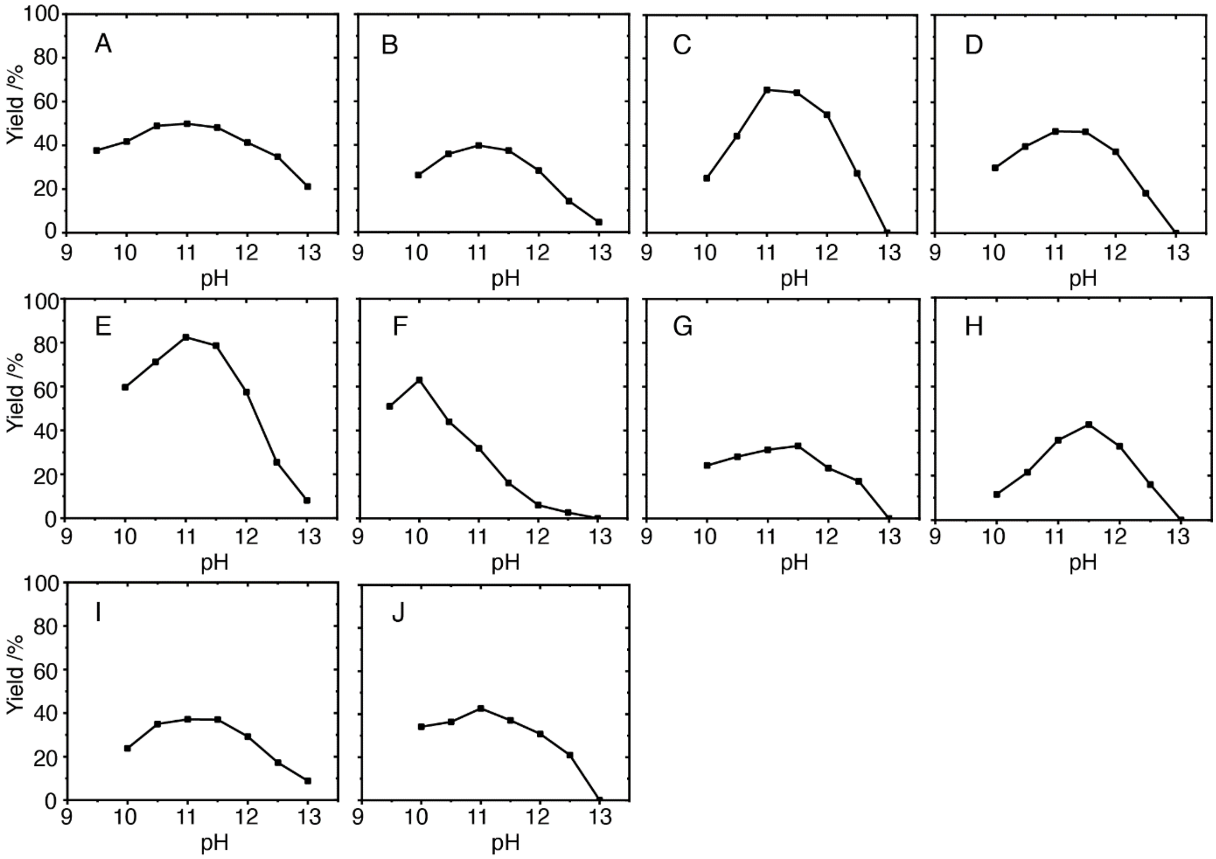

Supplementary Fig. 46. The effects of pH on the yields of luteolin cross-coupling reaction. Luteolin (**A**), apigenin (**B**), diosmetin (**C**), chrysin (**D**), wogonin (**E**), 5,6-dihydroxyflavone (**F**), genistein (**G**), 5, 3′, 4′-trihydroxyflavone (**H**), 6, 3′, 4′-trihydroxyflavone (**I**) and 7, 3′, 4′-trihydroxyflavone (**J**).

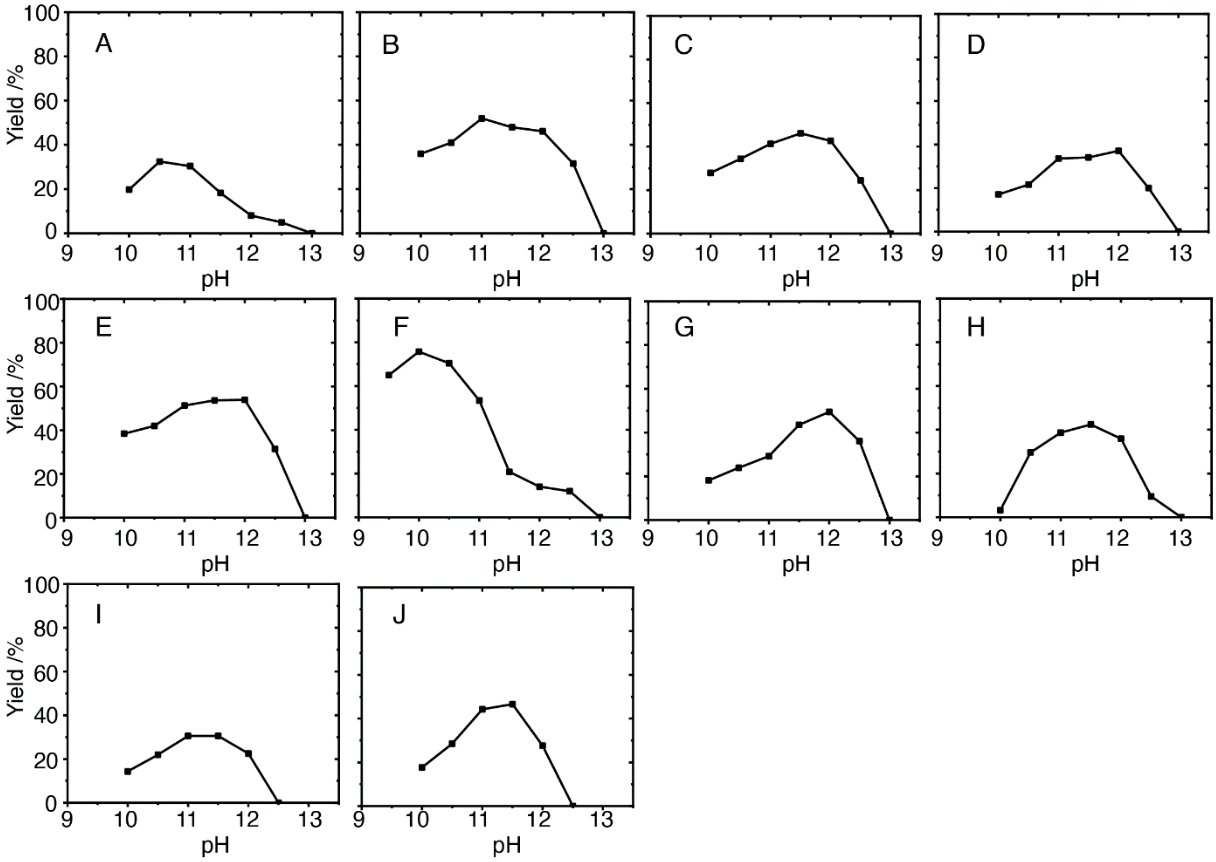

Supplementary Fig. 47. Effects of pH on the yields of dicranolomin cross-coupling reaction. Luteolin (**A**), apigenin (**B**), diosmetin (**C**), chrysin (**D**), wogonin (**E**), 5,6 dihydroxyflavone (**F**), genistein (**G**), 5, 3′, 4′ trihydroxyflavone (**H**), 6, 3′, 4′ trihydroxyflavone (**I**) and 7, 3′, 4′ trihydroxyflavone (**J**).

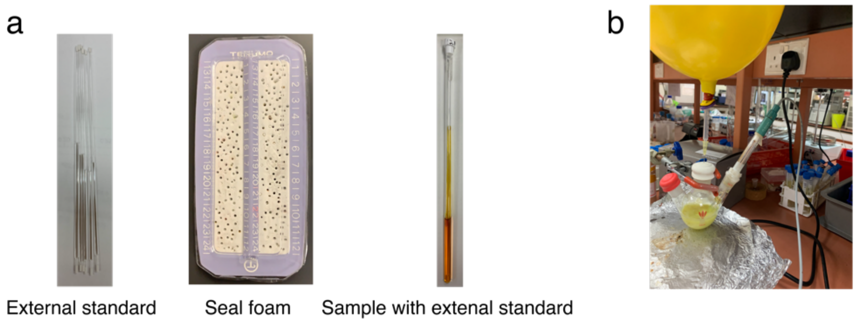

Supplementary Fig. 48. Experimental set up for determination of pK_as of luteolin. (**A**) The sample preparation of luteolin for pK_a calculation, and experimental set (**B**) for sample preparation.

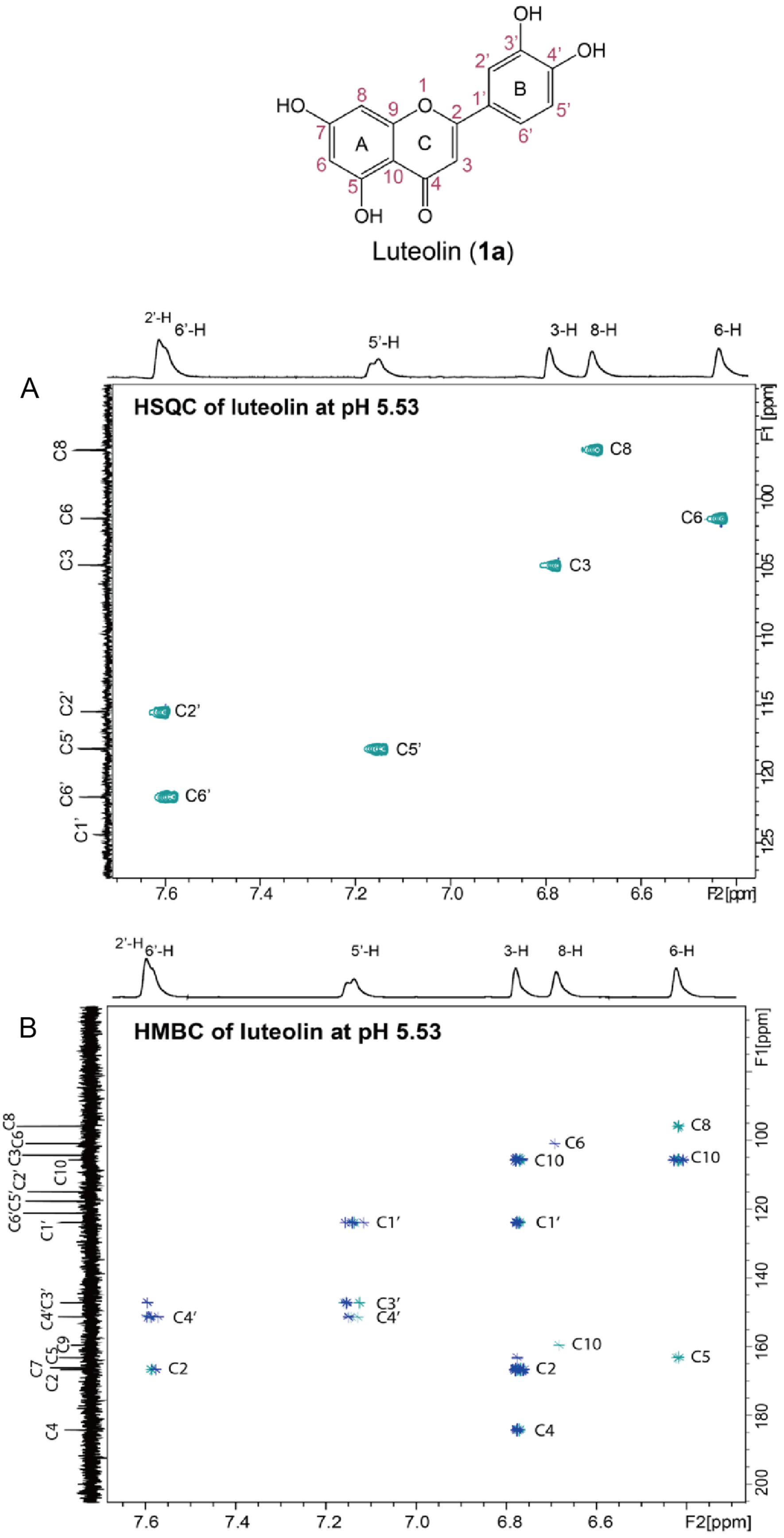

Supplementary Fig. 49. 2D NMR spectrum of luteolin at pH 5.53. **A)** HSQC of luteolin at pH 5.70**, B)** HMBC of luteolin at pH 5.53.

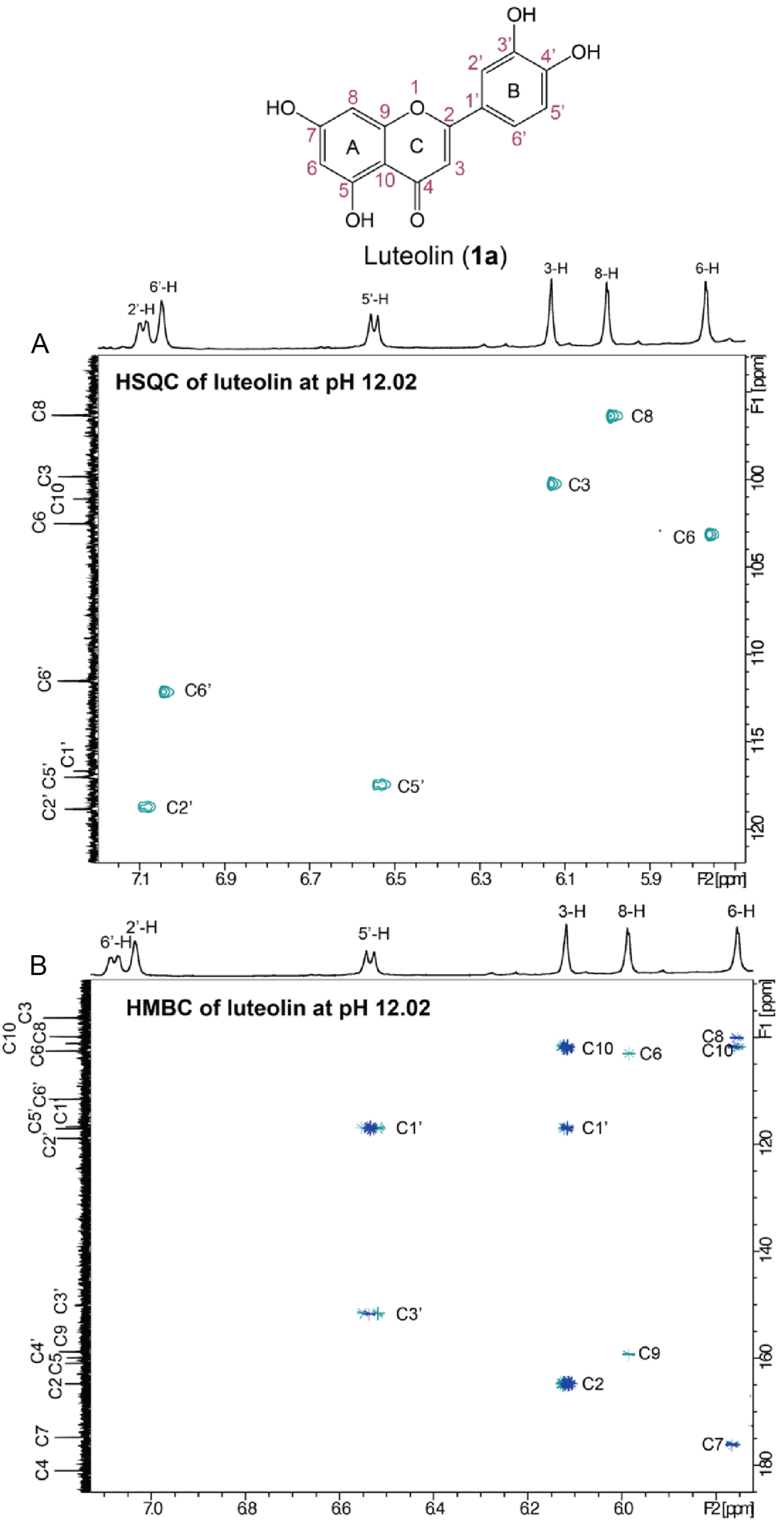

Supplementary Fig. 50. 2D NMR spectrum of luteolin at pH 12.02. **A)** HSQC of luteolin at pH 12.02, **B)** HMBC of of luteolin at pH 12.02**.**

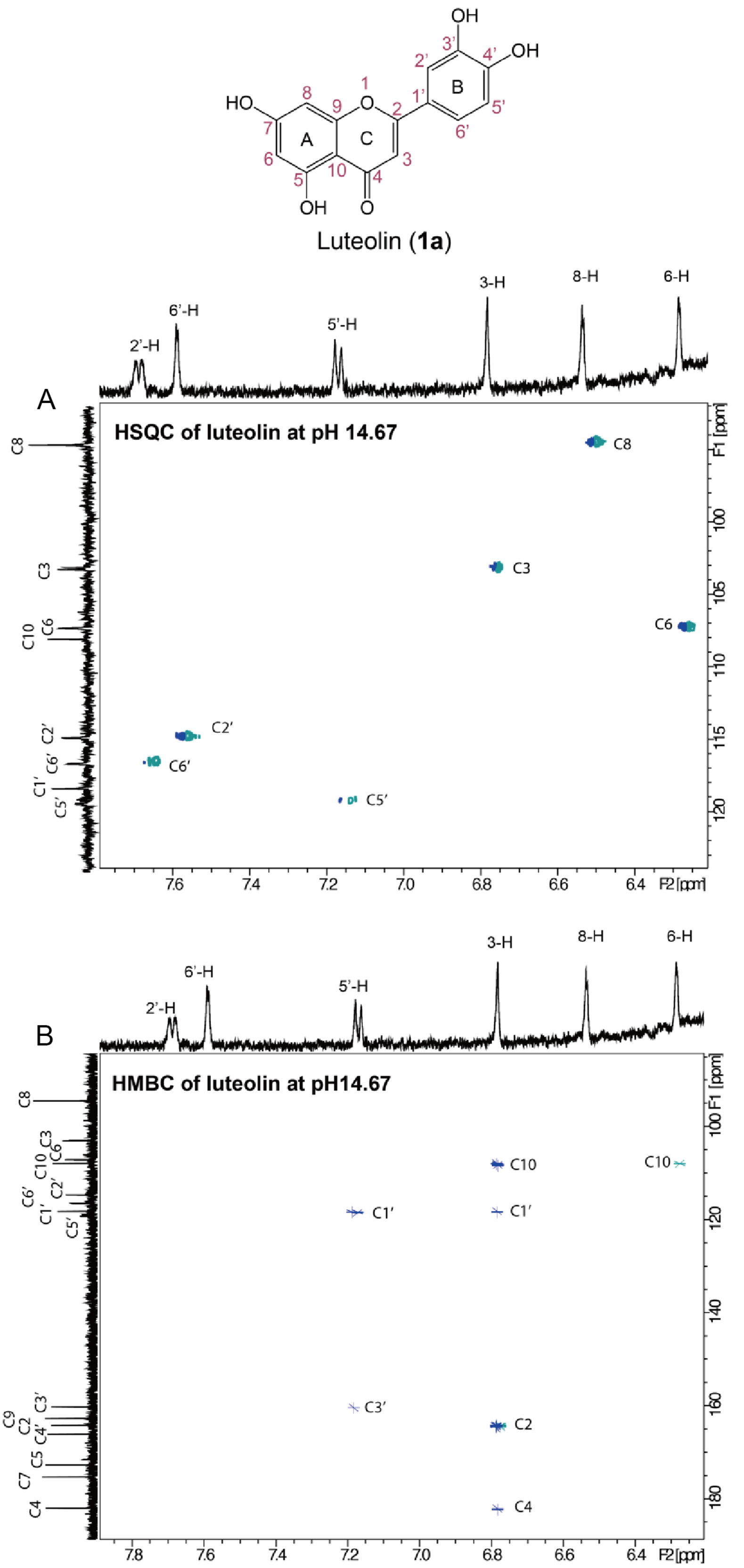

Supplementary Fig. 51. 2D NMR spectrum of luteolin at pH 14.67. **A)** HSQC of luteolin at pH 14.67, **B)** HMBC of of luteolin at pH 14.67**.**

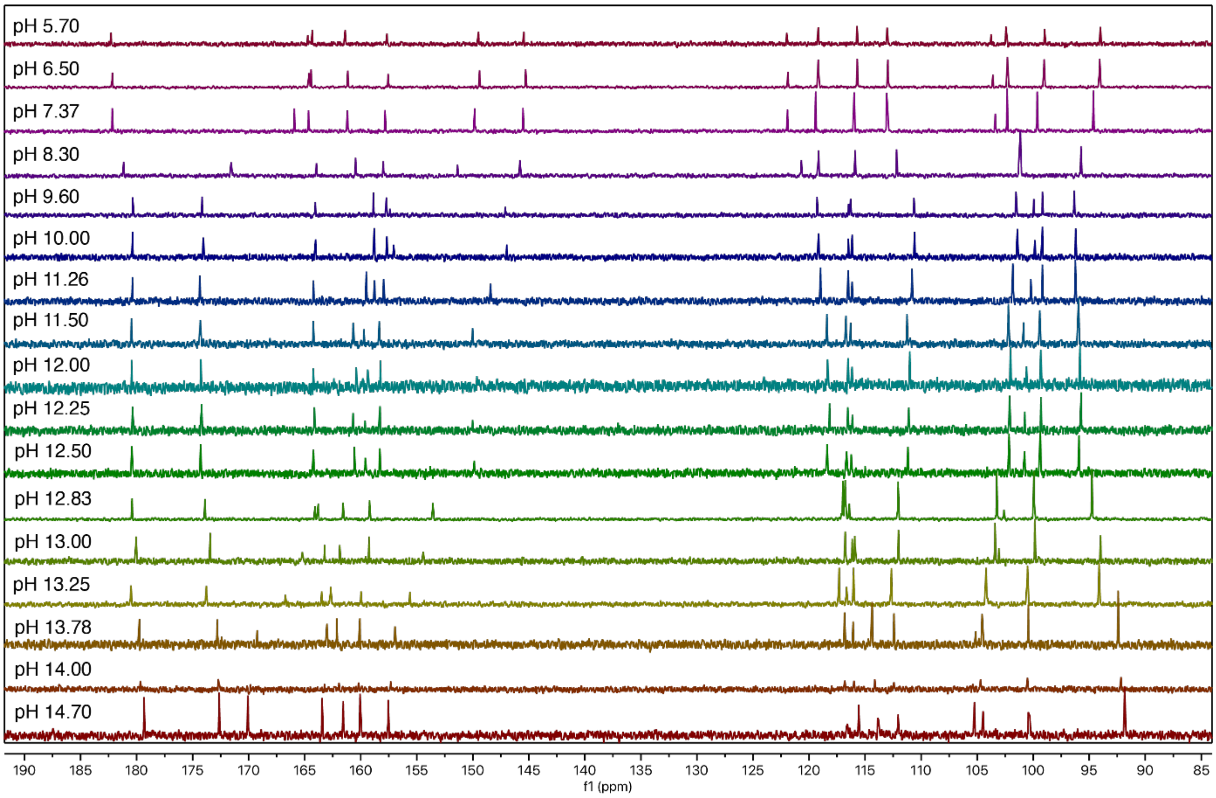

Supplementary Fig. 52. The full spectra of pH dependent ^{13}C NMR stacked of luteolin.

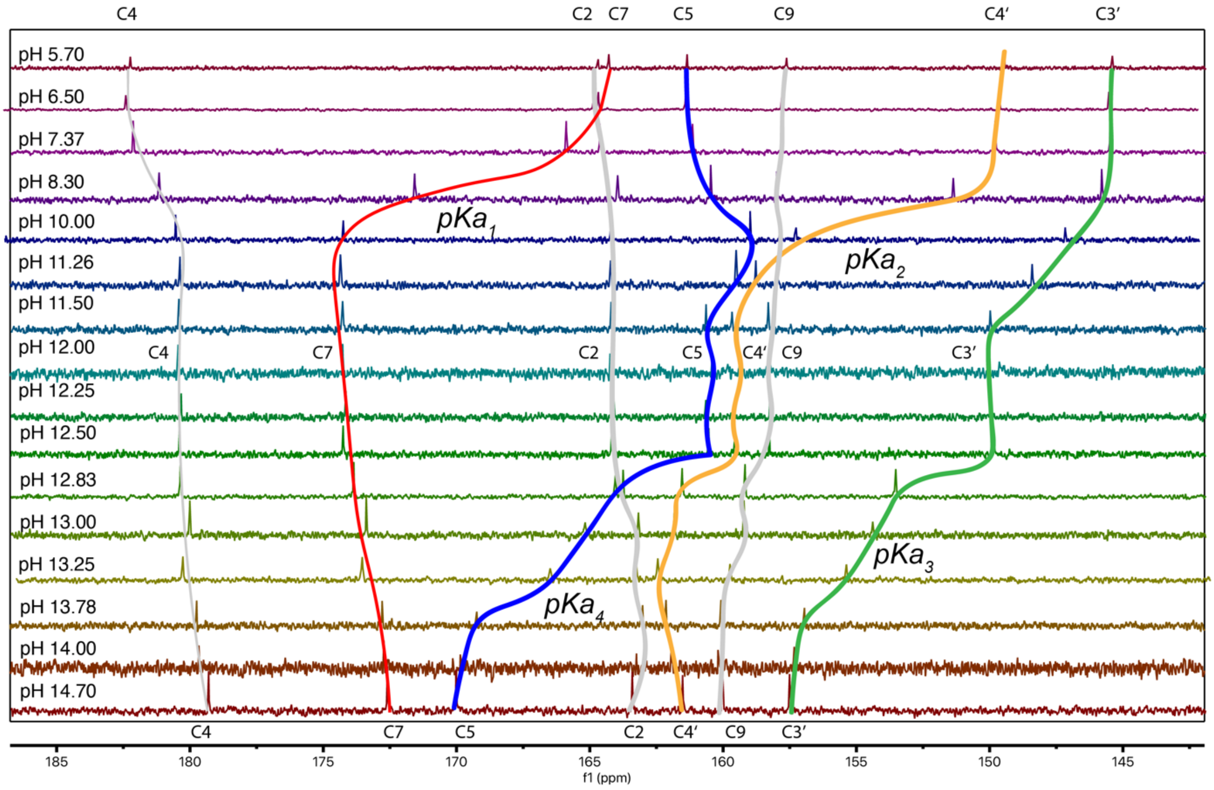

Supplementary Fig. 53. ^{13}C chemical shift dependence on the deprotonation state of luteolin. The assignment of carbons was further confirmed by HMBC with 2D correlation with ^{1}H NMR.

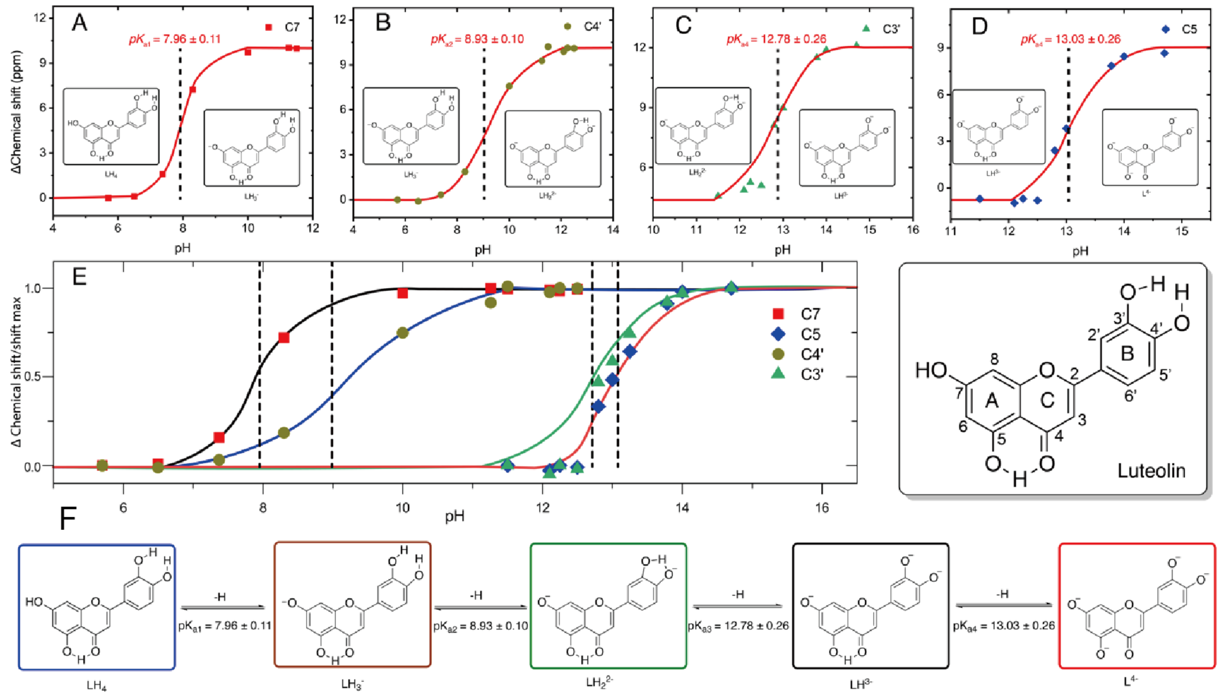

Supplementary Fig. 54. *pKa* calculations from the ^{13}C NMR chemical shifts. pK_{a1}, pK_{a2}, pK_{a3} *and* pK_{a4} determination of luteolin based on dependence of ^{13}C NMR chemical shifts of C7 (**A**), C4′ (**B**), C3′ (**C**) and C5 (**D**) of luteolin on pH. Data are presented as mean ± SD. The lines represent computer fits to the Henderson–Hasselbalch equation with the pK_a values. (**E**) The distribution curve of luteolin species in aqueous solution was generated based on pK_a values (LH_4: blue, LH_3^-: brown, LH_2^{2-}: green, LH^{3-}: black and L^{4-}: red). (**F**) The luteolin species in aqueous solution.

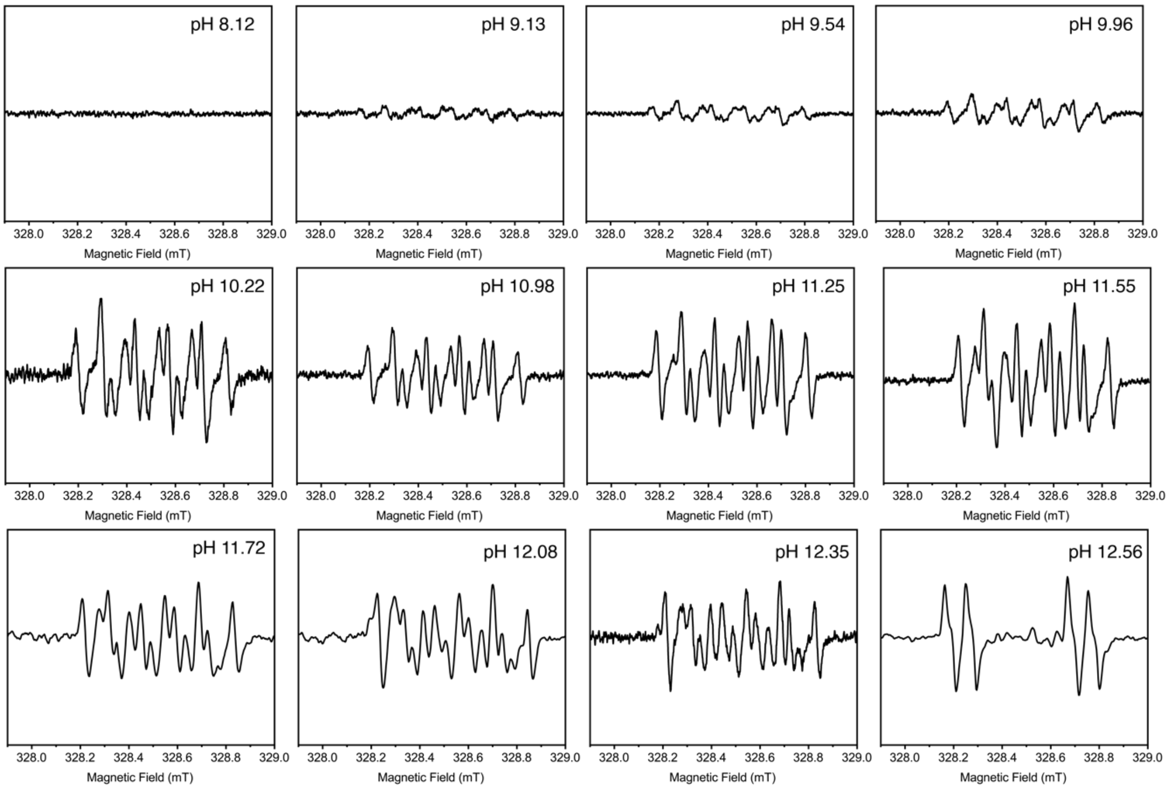

Supplementary Fig. 55. The pH dependent ESR spectra of luteolin (15.0 mM) in aqueous KOH solution. The determination was conducted immediately when luteolin was mixed with KOH solution at specific concentrations, the pH was detected after EPR test.

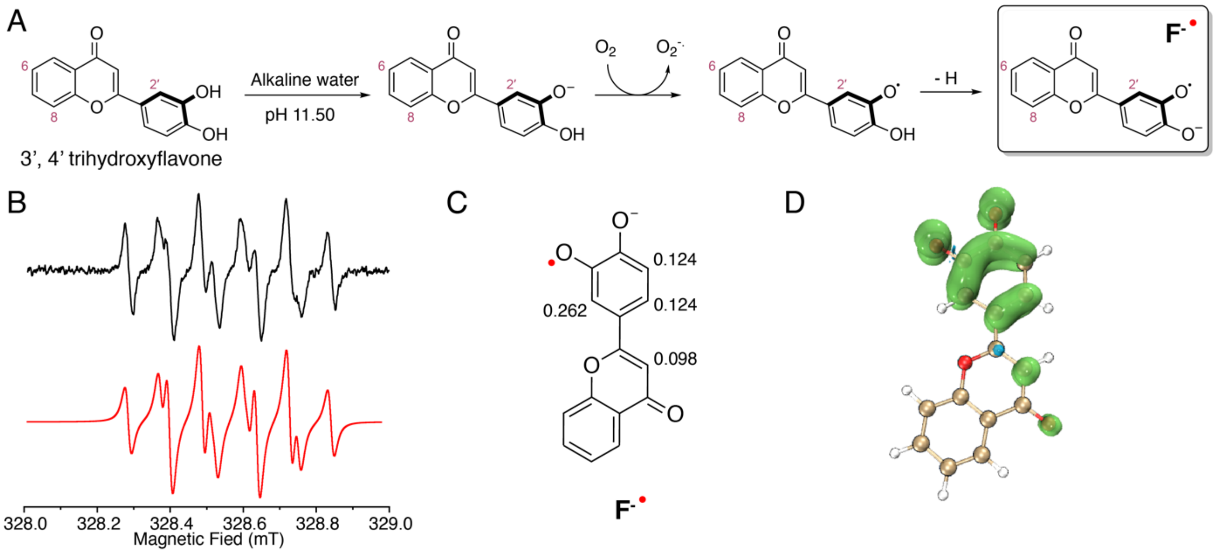

Supplementary Fig. 56. Characterization of 3′,4′-dihydroxyflavone radical anion. (**A**) The formation of 3′, 4′ dihydroxyflavone radical anion. (**B**) Experimental (black) and fitted (red) EPR spectrum of $F^{-\bullet}$ recorded at 295 K. The EPR spectrum of radical was simulated by by JEOL IsoSimu/Fa Version 2.2.0 isotropic simulation program. (**C**) Experimental hyperfine coupling constants for $F^{-\bullet}$. (**D**) Spin-density distribution in $F^{-\bullet}$ predicted with DFT (UM062x/6-311+G(d,p)).

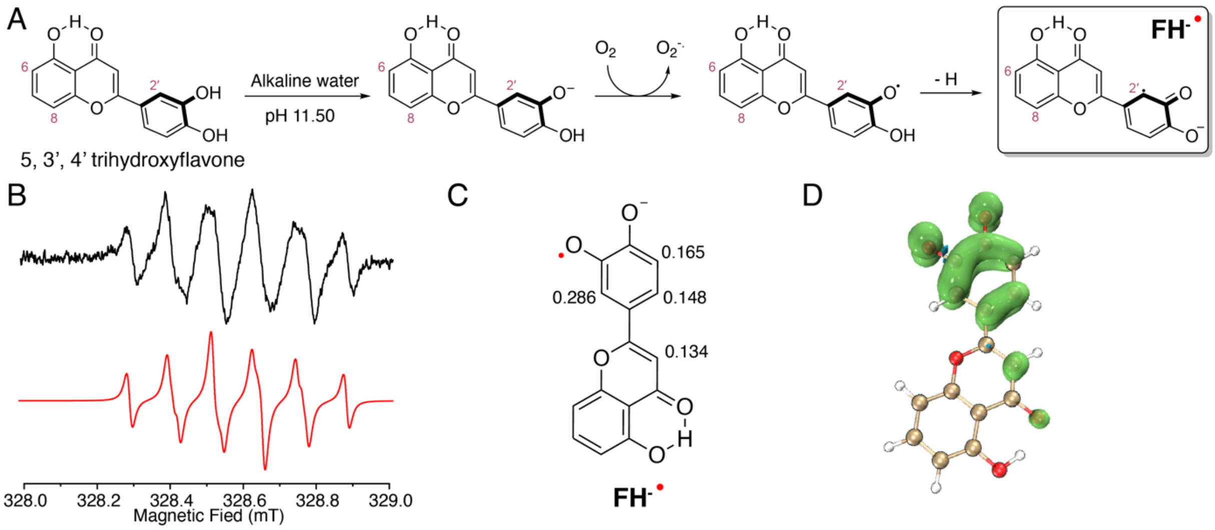

Supplementary Fig. 57. Characterization of 5,3′,4′-trihydroxyflavone radical anion. (**A**) The formation of 5,3′,4′-trihydroxyflavone radical anion. (**B**) Experimental (black) and fitted (red) EPR spectrum of FH$^{-\bullet}$ recorded at 295 K. (**C**) Experimental hyperfine coupling constants for F$^{-\bullet}$. (**D**) Spin-density distribution in FH$^{-\bullet}$ predicted with DFT (UM062x/6-311+G(d,p)).

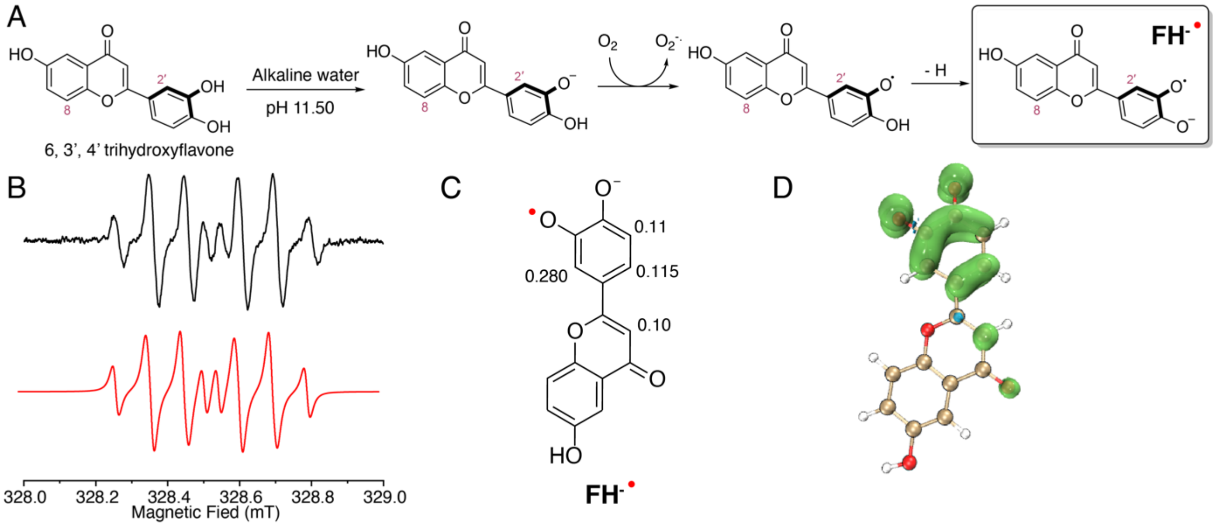

Supplementary Fig. 58. Characterization of 6,3′,4′-trihydroxyflavone radical anion. (**A**) The formation of 6,3′,4′-trihydroxyflavone radical anion. (**B**) Experimental (black) and fitted (red) EPR spectrum of $FH^{-\bullet}$ recorded at 295 K. (**C**) Experimental hyperfine coupling constants for $F^{-\bullet}$. (**D**) Spin-density distribution in $FH^{-\bullet}$ predicted with DFT (UM062x/6-311+G(d,p)).

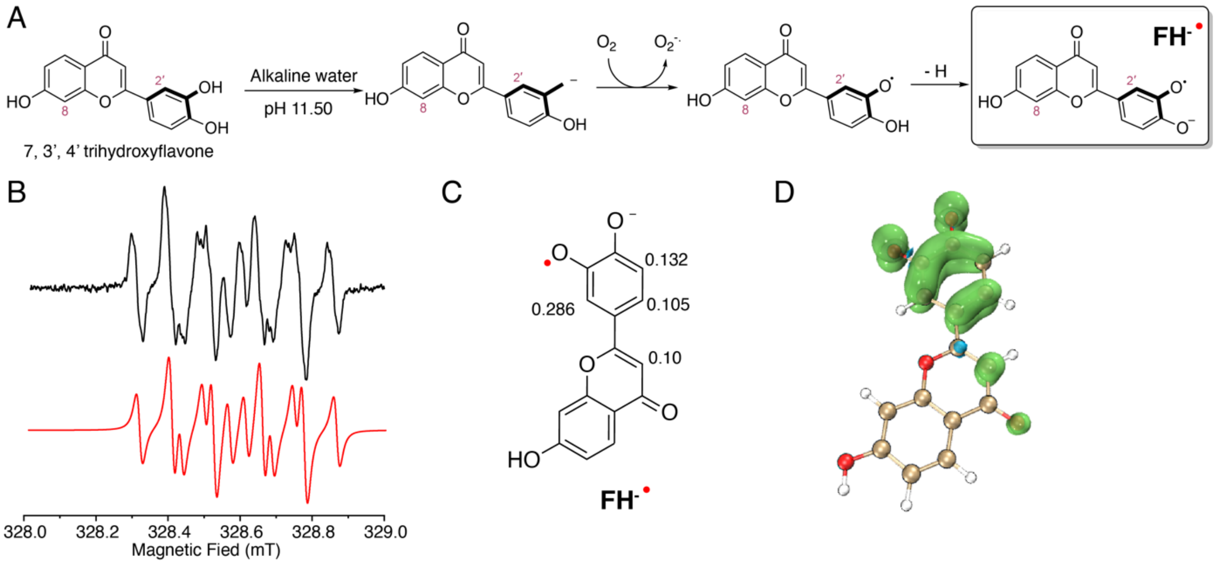

Supplementary Fig. 59. Characterization of 7,3′,4′-trihydroxyflavone radical anion. (**A**) The formation of 7,3′,4′-trihydroxyflavone radical anion. (**B**) Experimental (black) and fitted (red) EPR spectrum of $FH^{-\bullet}$ recorded at 295 K. (**C**) Experimental hyperfine coupling constants for $FH^{-\bullet}$. (**D**) Spin-density distribution in $FH^{-\bullet}$ predicted with DFT (UM062x/6-311+G(d,p)).

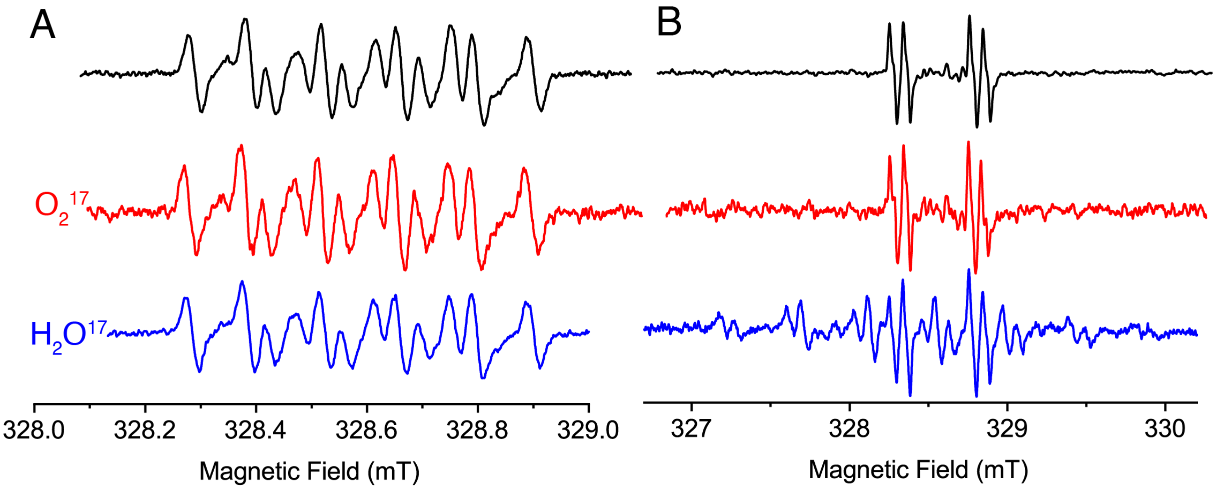

Supplementary Fig. 60. The ESR spectra of luteolin in aqueous KOH solutions under different pH. (**A**) pH 11.5 and (**B**) pH 12.5. Black lines are the EPR spectrum of luteolin dissolved in H_2O^{16} and exposed in oxygen O_2^{16}, red lines are the EPR spectrum of luteolin dissolved in H_2O^{16} and exposed in oxygen O_2^{17} and blue lines are the EPR spectra of luteolin dissolved in H_2O^{17} and exposed in oxygen O_2^{16}.

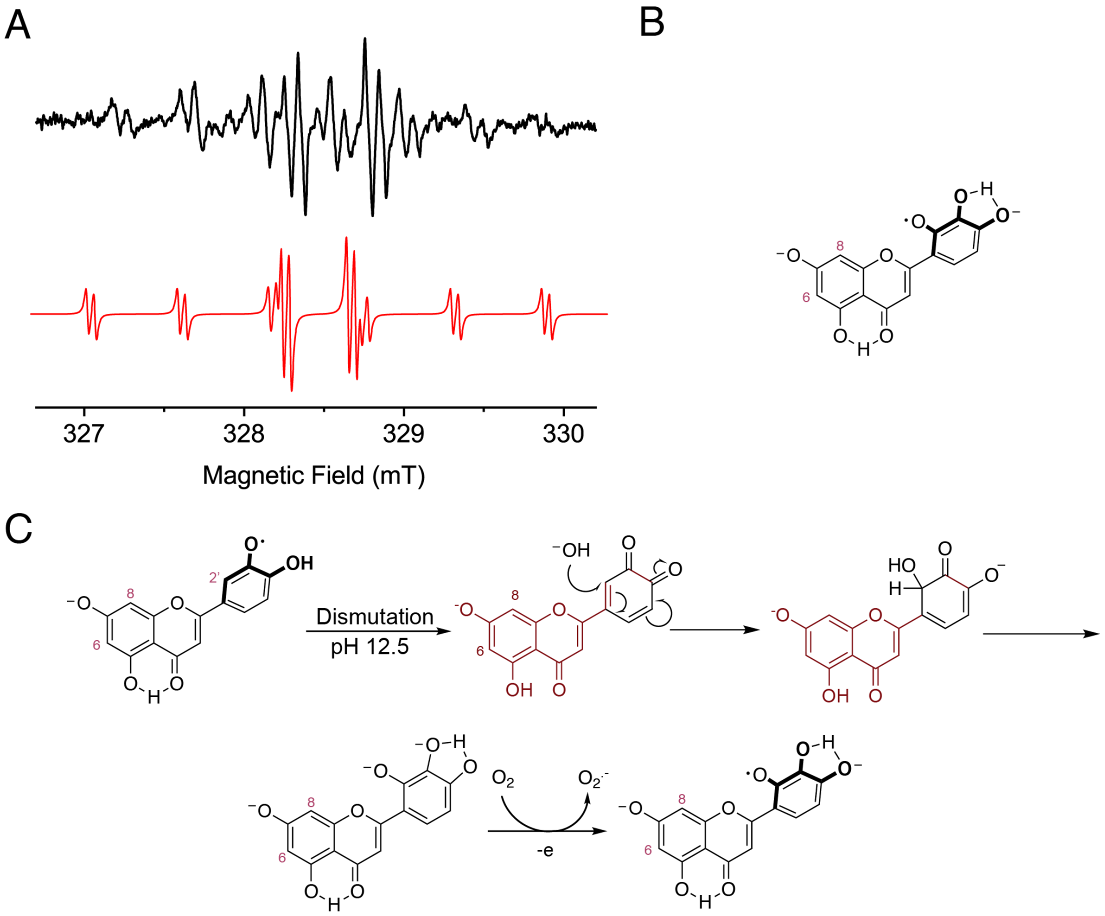

Supplementary Fig. 61. The ESR spectrum of luteolin in aqueous KOH solutions under pH 12.5. (**A**) Experimental (black) and simulated (red) EPR spectra of luteolin dissolved in H_2O^{17}. (**B**) Spin-density distribution in $LH_2O^{-\cdot}$ predicted with DFT (UM062x/6-311+G(d,p)). (**C**) Proposed mechanism of the formation of $LH_2O^{-\cdot}$ radical anion.

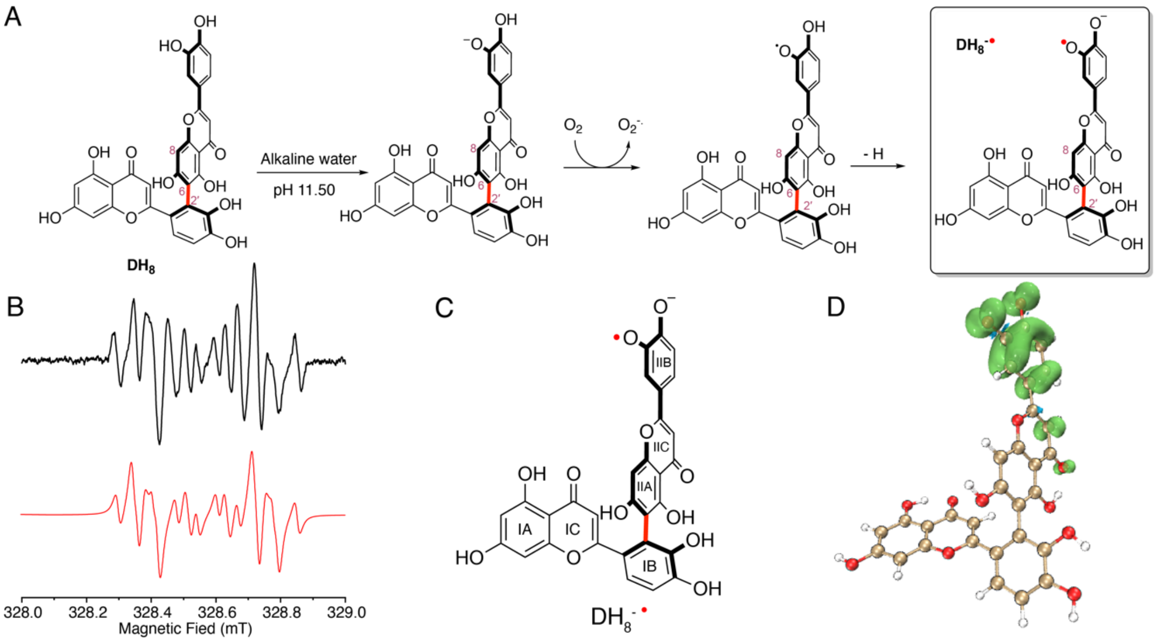

Supplementary Fig. 62. Characterization of dicranolomin radical anion. (**A**) The formation of 3', 4' dihydroxyflavone radical anion. (**B**) Experimental (black) and fitted (red) EPR spectrum of $F^{-\bullet}$ recorded at 295 K. The EPR spectrum of radical was simulated by by JEOL IsoSimu/Fa Version 2.2.0 isotropic simulation program. (**C**) Proposed structure for dicranolomin radical anion $DH_8^{\cdot -}$. (**D**) Spin-density distribution in $DH_8^{\cdot -}$ predicted with DFT (UM062x/6-311+G(d,p)).

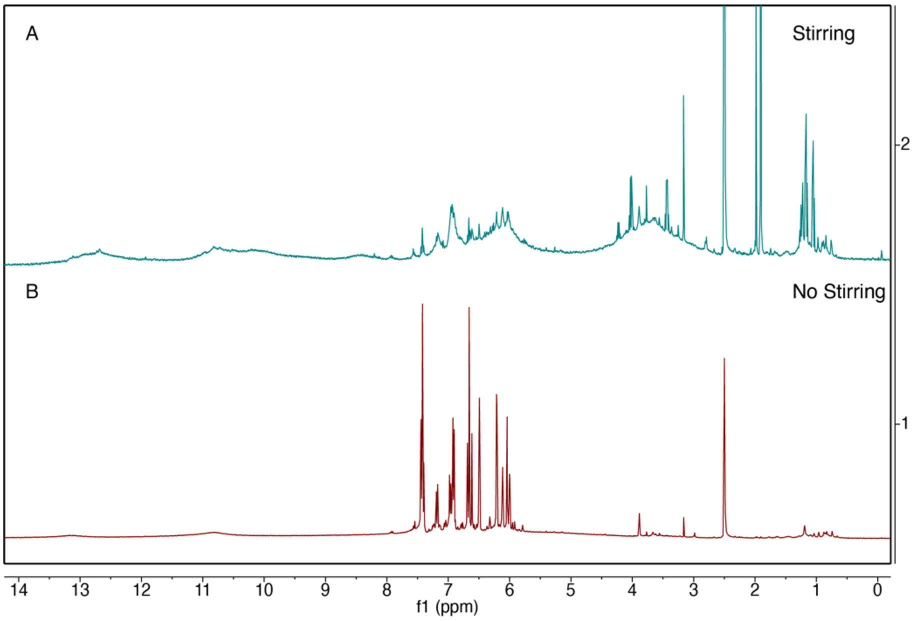

Supplementary Fig. 63. Crude NMR spectrum of luteolin-luteolin cross-coupling. **A**) Reaction under stirring all the time. **B**) Reaction without stirring. Luteolin **1a** (0.045 mmol) were dissolved in 3 mL base solution, incubated in sealed tube 15 mL for 10 h, then solution was acidified with concentrated hydrochloric acid to pH value of 5 ~ 7. After evaporating all aqueous solution, the solid was dissolved in deuterated reagent.

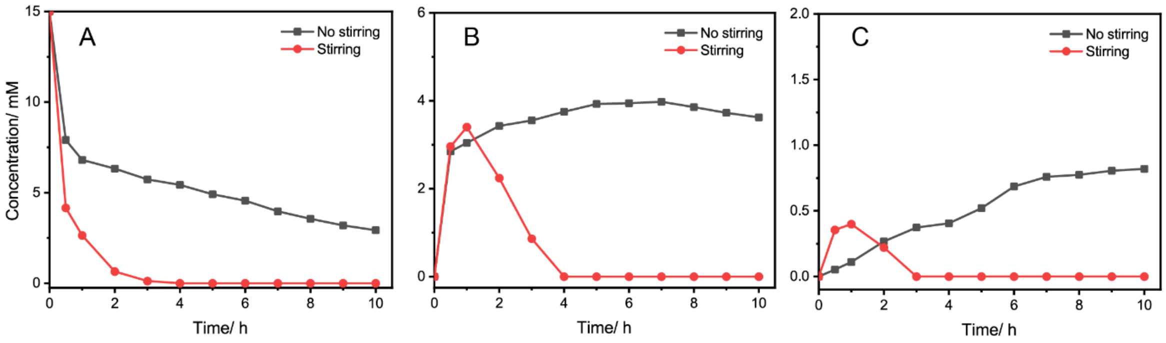

Supplementary Fig. 64. Time course plots of reactant and products in luteolin coupling reaction. (**A)** luteolin (**1a**), (**B)** dicranolomin (**2a**) and (**C)** distichumtriluteolin (**3a**) in luteolin-luteolin cross-coupling reaction with stirring and without stirring.

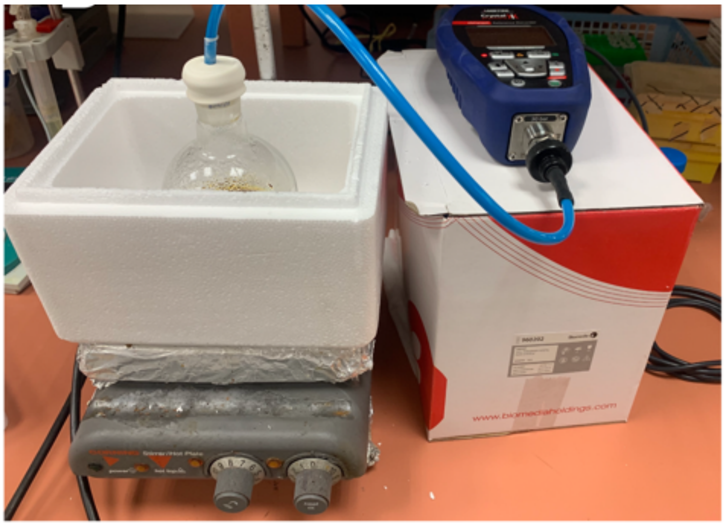

Supplementary Fig. 65. The experimental set-up used for monitoring oxygen consumption rates in the oxidative coupling reaction. The pressure changes of reaction under stirring and no stirring was recorded by pressure gauge (NVISION, pressure recorder with vacuum range of 30 MPa, CRYSTAL engineering corporation).

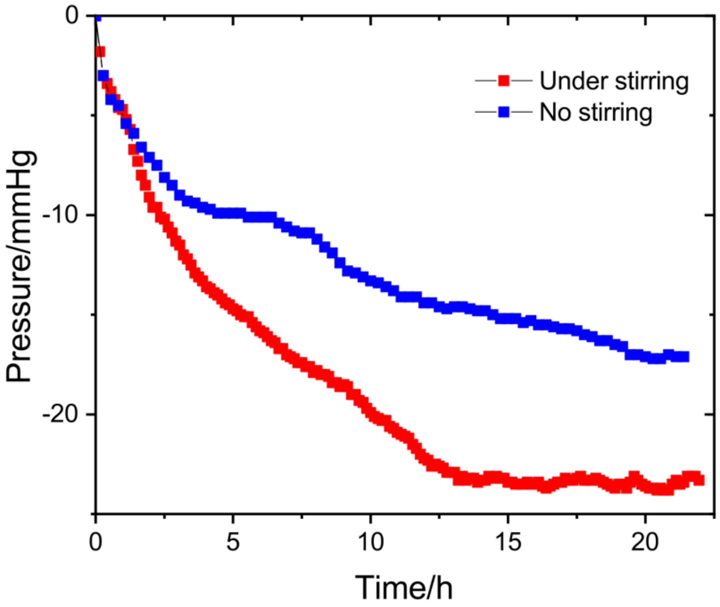

Supplementary Fig. 66. The oxygen consumption kinetic curves of luteolin-luteolin cross-coupling reaction under stirring and no stirring conditions. 57.2 mg luteolin (0.1 mmol) was added 250 mL round bottle flask, then 50.0 mL KOH solution (0.03 M) was introduced to start the reaction. The reaction was conducted in real-time monitoring in an air-tight system.

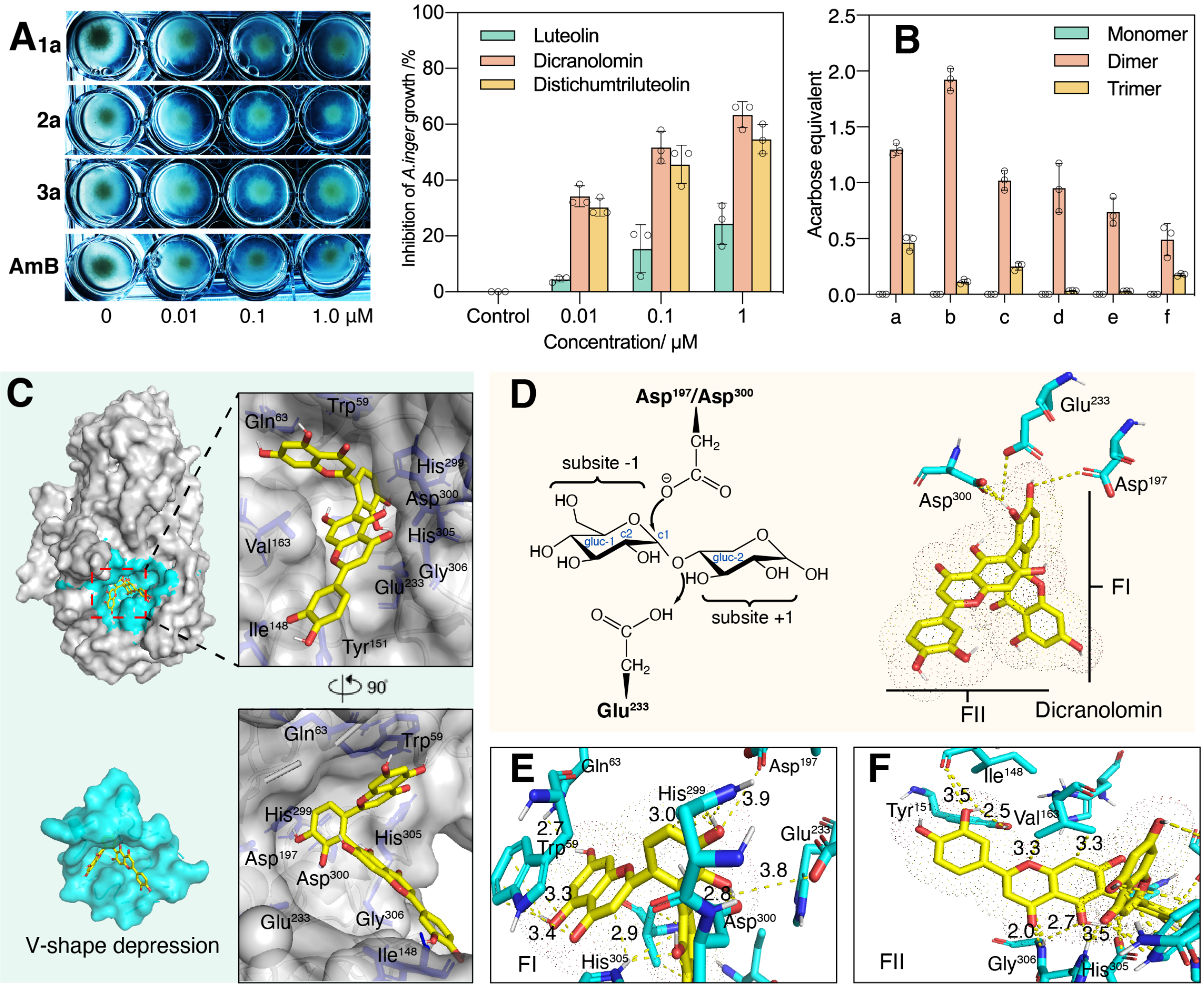

Supplementary Fig. 67. Bioactivity of luteolin and flavone dimers and trimers. (**A**) Image of surviving *Aspergillus niger* colony in the presence of luteolin (**1a**), dicranolomin (**2a**), distichumtriluteolin (**3a**) and amphotericin B (**AmB**), and degrees of inhibition (%) (n = 3). Data are presented as mean ± SD. (**B**) α-Amylase inhibition activities of flavonoids oligomers (monomers, dimers and trimers) with acarbose as a reference standard (n = 3). Data are presented as mean ± SD. (**C**) The docking of **2a** in complex with α-amylase [Protein Data Bank (PDB) 3GBN]. **2a** is shown in a ball-and-stick representation, with whole amylase protein rendered in surface representation. The substrate-binding pocket was highlighted in cyan while the rest in light gray. A magnified view of **2a** binding site in the enzyme is shown with the C and O atoms of **2a** in yellow and red respectively. Overlay of the structure of **2a** (yellow ball and sticks) in complex with interacting loop residues (blue sticks). (**D**) Catalytic mechanism of α-amylase. Reaction occurs with acid/base and nucleophilic assistance provided by amino acid side chains from Asp197, Glu233 and Asp300. (**E**) and (**F**) show polar interactions in α-amylase, with the interactions of FI and FII part of **2a** [labeled in (**D**)] depicted as black dotted lines and measured in Å.

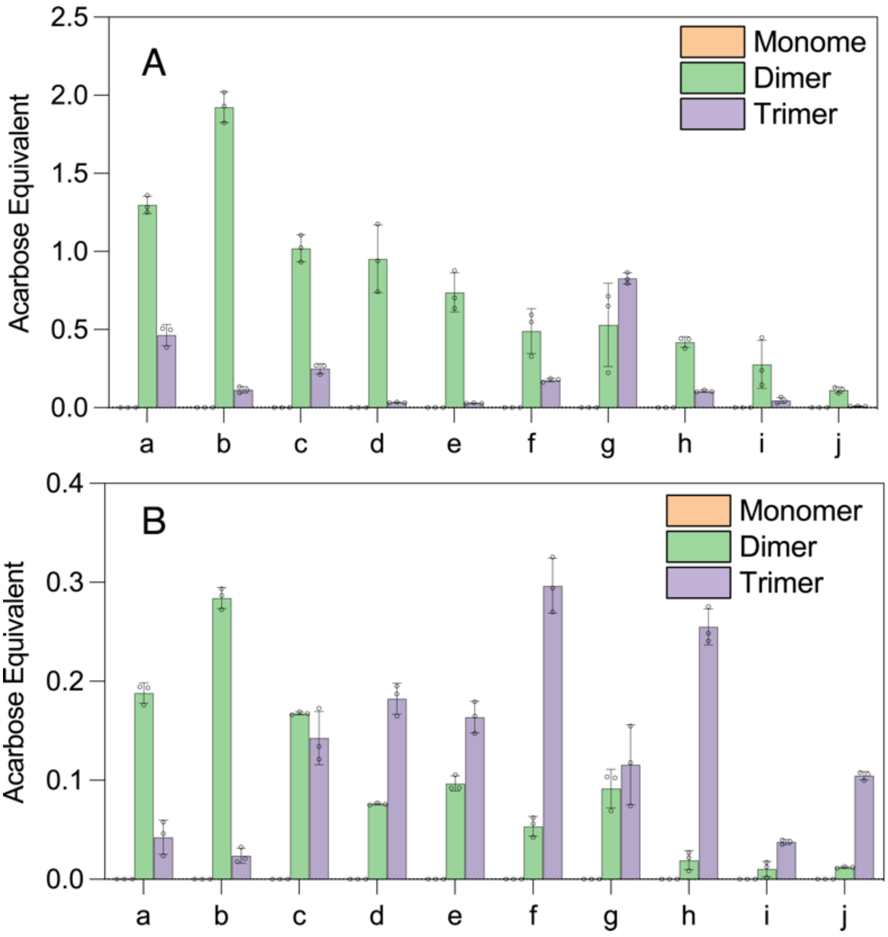

Supplementary Fig. 68. The starch hydrolysis inhibition activities of flavonoid oligomers showed in acarbose equivalent. (**A**) The α-amylase inhibition activity of flavonoids oligomers (n = 3), (**B**) The α-glucosidase inhibition activity of flavonoids oligomers (n = 3). Data are presented as mean ± SD.

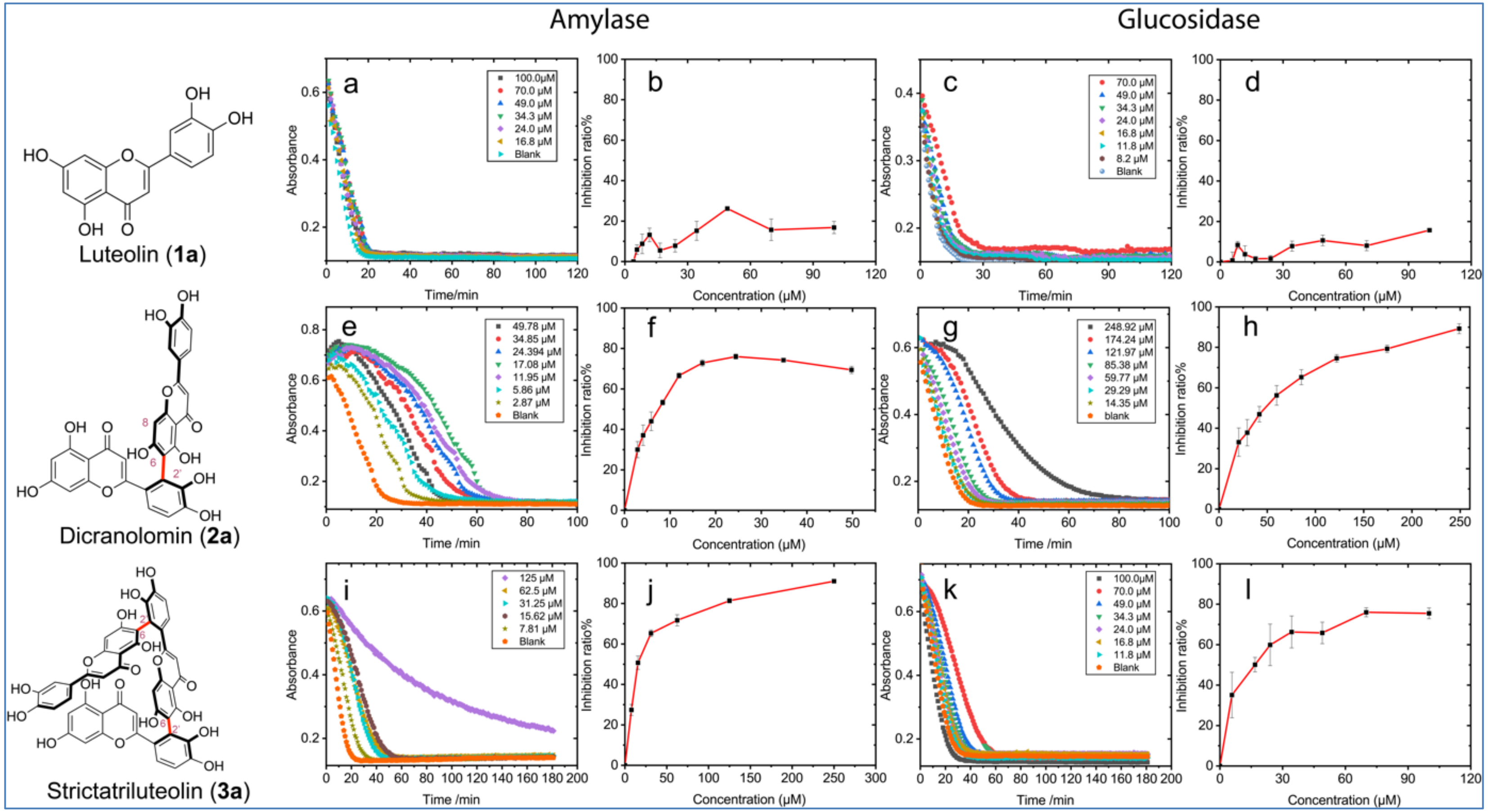

Supplementary Fig. 69. The representative kinetic curves and dose response curve of α-amylase and α-glucosidase in the presence of luteolin (1a), its derivatives, biflavone (2a) and triflavone (3a) (n = 3). a, b, e, f, i and j show the starch hydrolysis activity of flavonoids with α-amylase while c, d, g, h, k and l show the starch hydrolysis activity of flavonoids with α-glucosidase. Data are presented as mean ± SD.

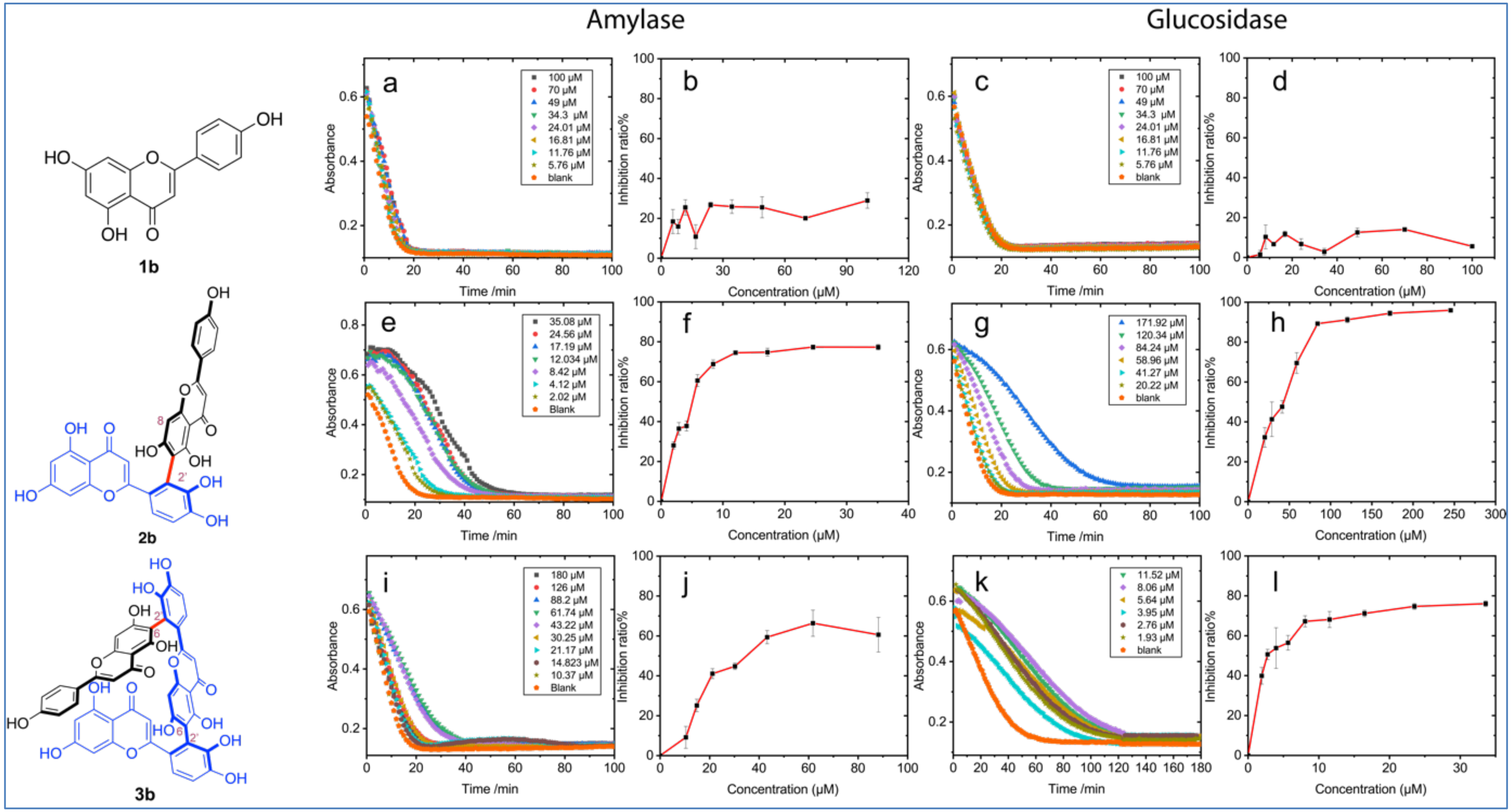

Supplementary Fig. 70. The representative kinetic curves and dose response curve of α-amylase and α-glucosidase in the presence of luteolin (1b), its derivatives, biflavone (2b) and triflavone (3b) (n = 3). a, b, e, f, i and j show the starch hydrolysis activity of flavonoids with α-amylase while c, d, g, h, k and l show the starch hydrolysis activity of flavonoids with α-glucosidase. Data are presented as mean ± SD.

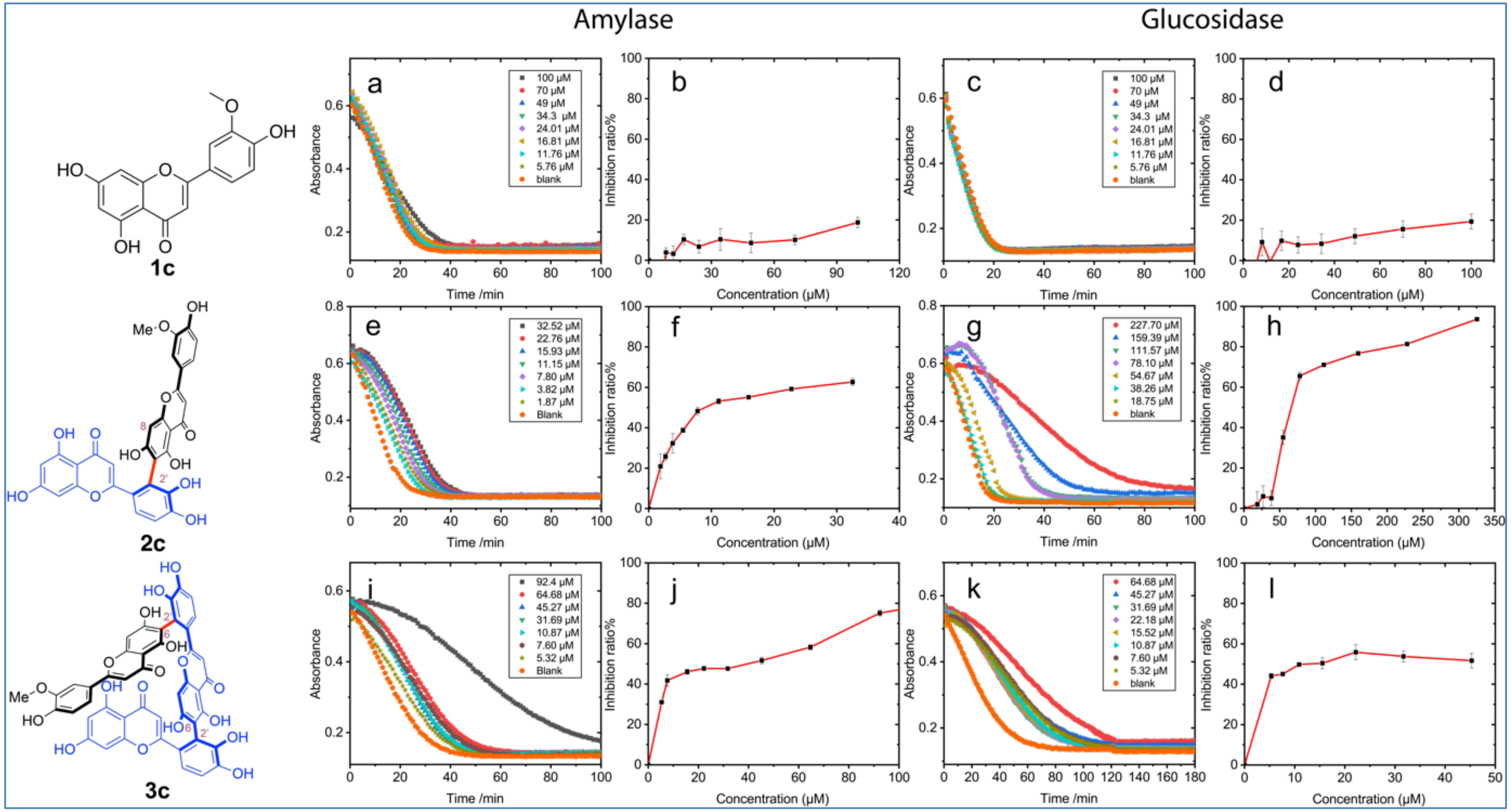

Supplementary Fig. 71. The representative kinetic curves and dose response curve of α-amylase and α-glucosidase in the presence of luteolin (1c), its derivatives, biflavone (2c) and triflavone (3c) (n = 3). a, b, e, f, i and j show the starch hydrolysis activity of flavonoids with α-amylase while c, d, g, h, k and l show the starch hydrolysis activity of flavonoids with α-glucosidase. Data are presented as mean ± SD.

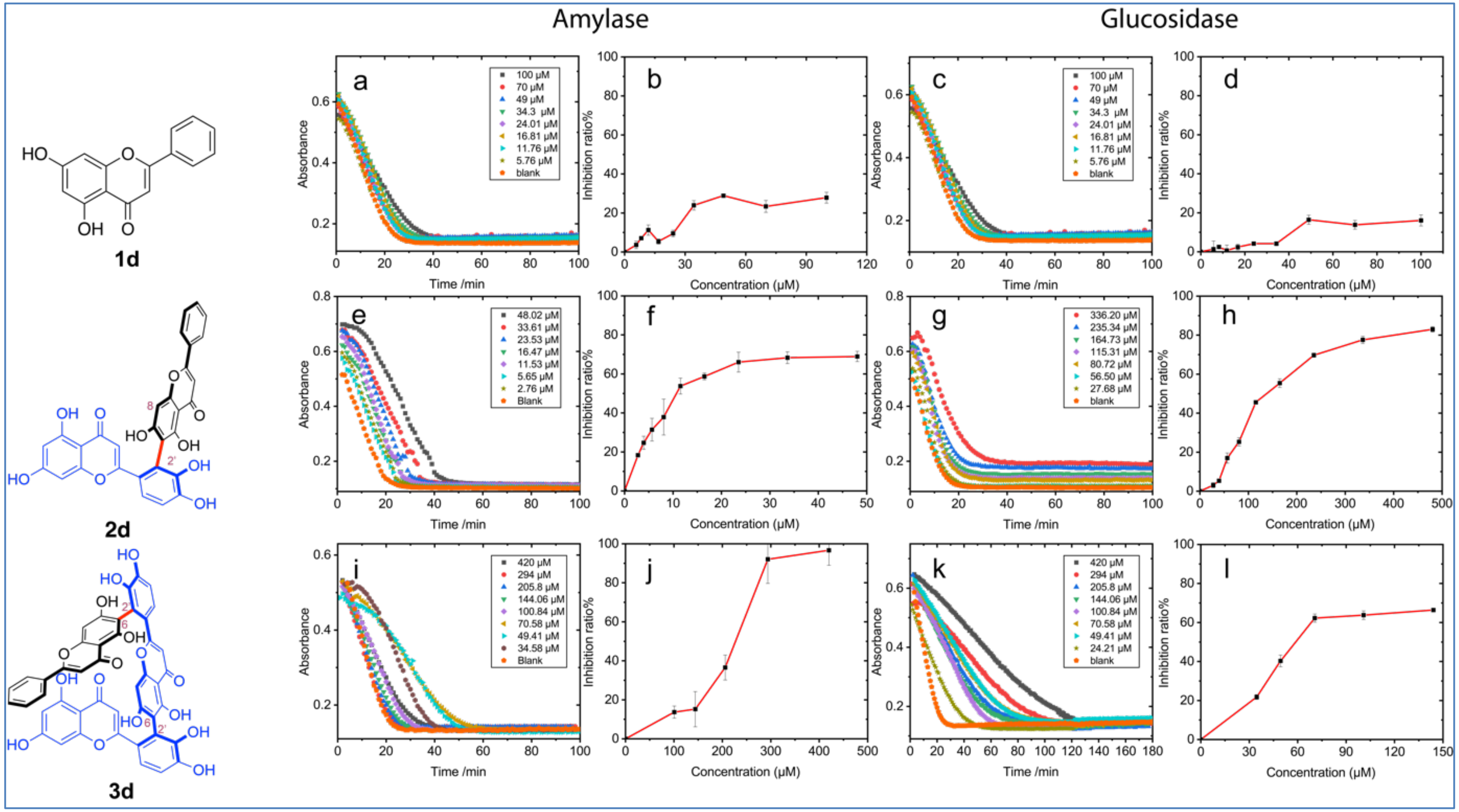

Supplementary Fig. 72. The representative kinetic curves and dose response curve of α-amylase and α-glucosidase in the presence of luteolin (1d), its derivatives, biflavone (2d) and triflavone (3d) (n = 3). a, b, e, f, i and j show the starch hydrolysis activity of flavonoids with α-amylase while c, d, g, h, k and l show the starch hydrolysis activity of flavonoids with α-glucosidase. Data are presented as mean ± SD.

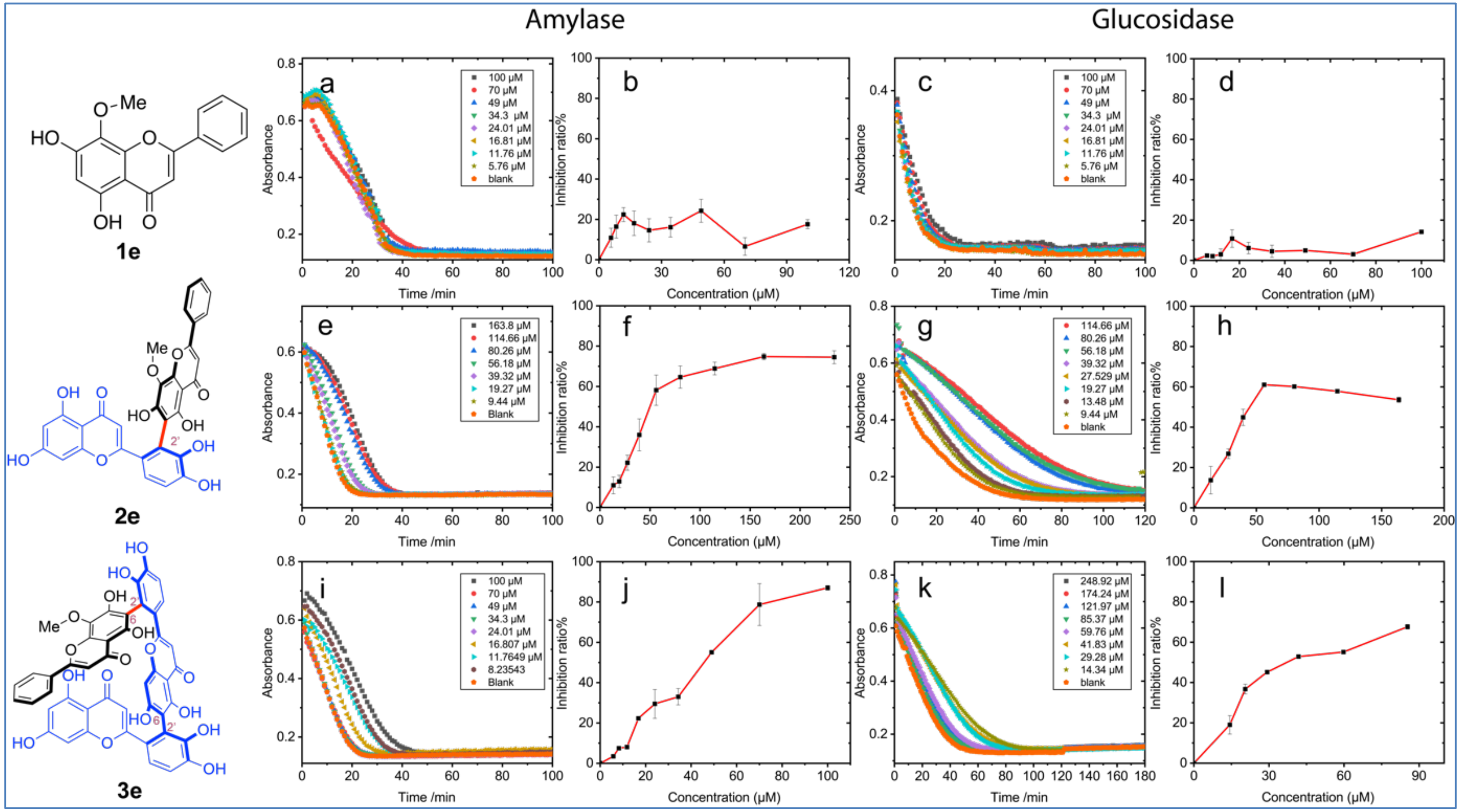

Supplementary Fig. 73. The representative kinetic curves and dose response curve of α-amylase and α-glucosidase in the presence of luteolin (1e), its derivatives, biflavone (2e) and triflavone (3e) (n = 3). a, b, e, f, i and j show the starch hydrolysis activity of flavonoids with α-amylase while c, d, g, h, k and l show the starch hydrolysis activity of flavonoids with α-glucosidase. Data are presented as mean ± SD.

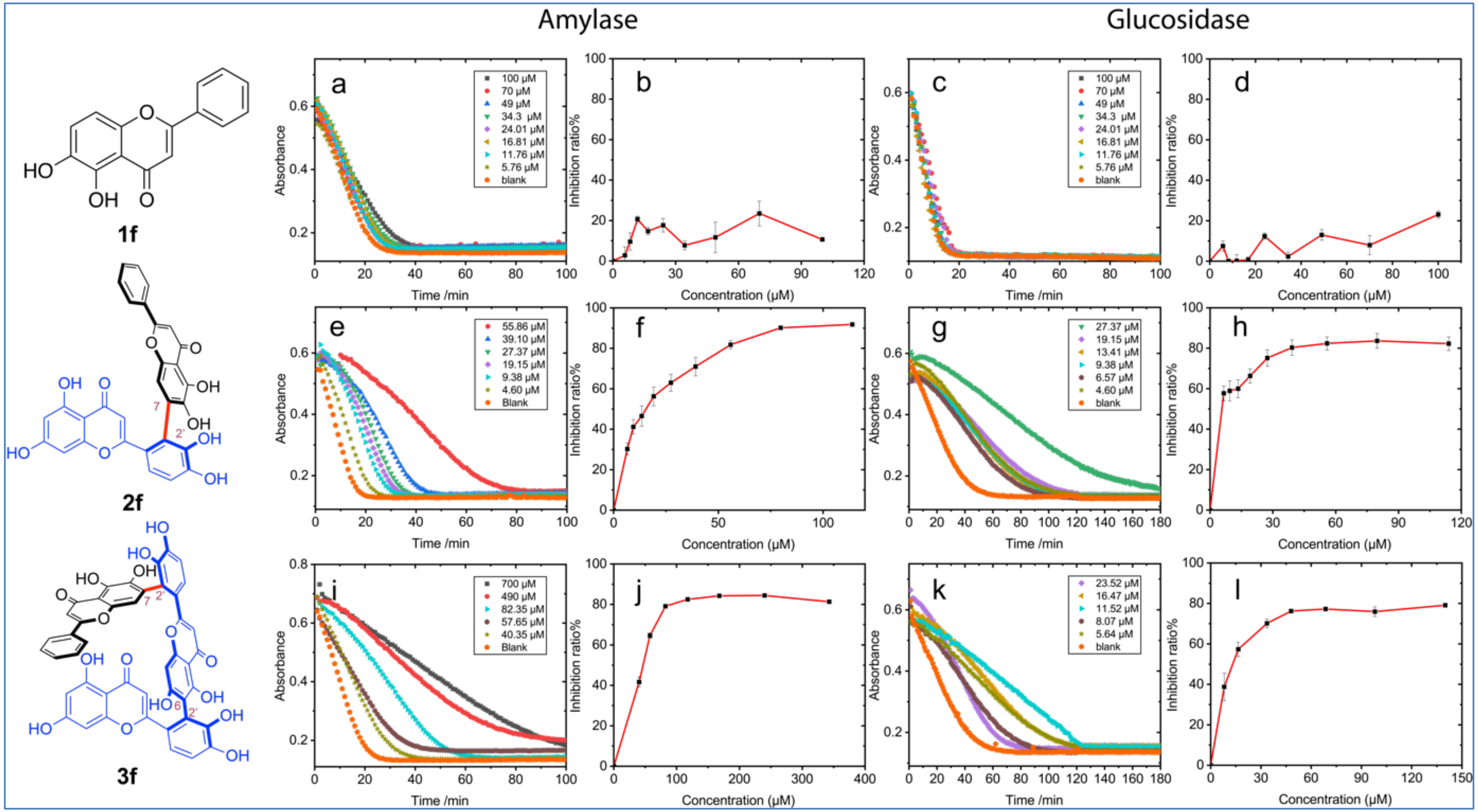

Supplementary Fig. 74. The representative kinetic curves and dose response curve of α-amylase and α-glucosidase in the presence of luteolin (1f), its derivatives, biflavone (2f) and triflavone (3f) (n = 3). a, b, e, f, i and j show the starch hydrolysis activity of flavonoids with α-amylase while c, d, g, h, k and l show the starch hydrolysis activity of flavonoids with α-glucosidase. Data are presented as mean ± SD.

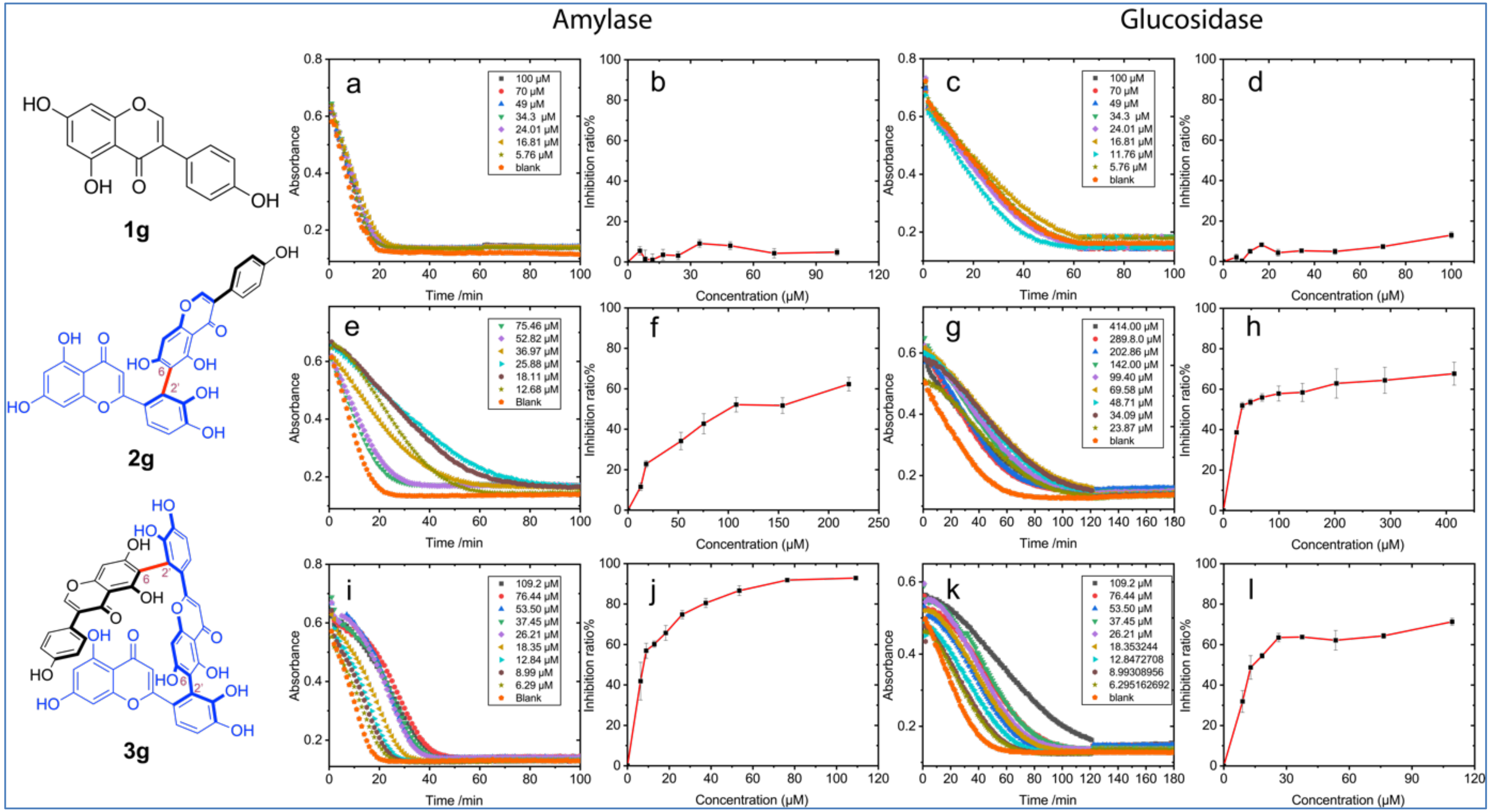

Supplementary Fig. 75. The representative kinetic curves and dose response curve of α-amylase and α-glucosidase in the presence of luteolin (1g), its derivatives, biflavone (2g) and triflavone (3g) (n = 3). a, b, e, f, i and j show the starch hydrolysis activity of flavonoids with α-amylase while c, d, g, h, k and l show the starch hydrolysis activity of flavonoids with α-glucosidase. Data are presented as mean ± SD.

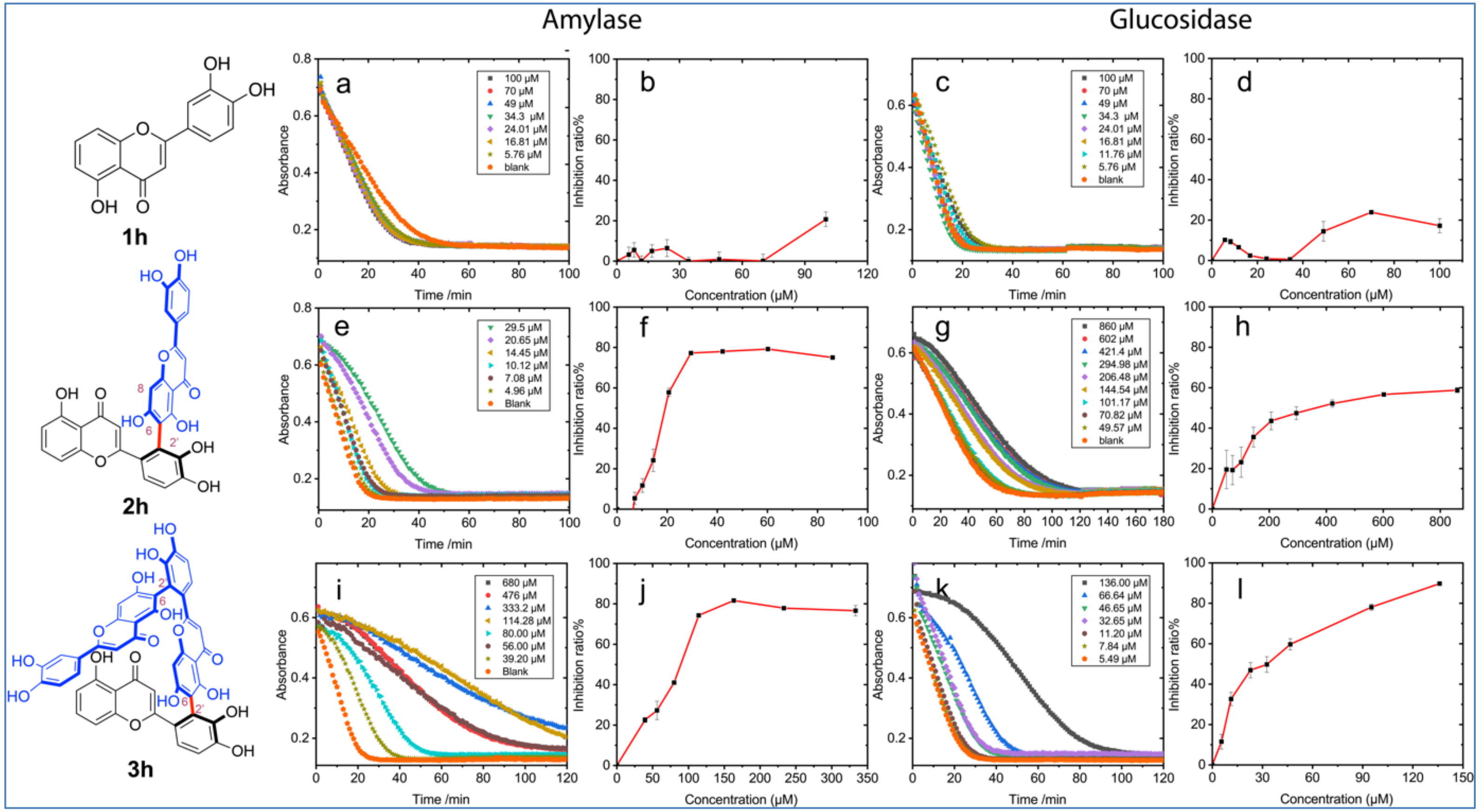

Supplementary Fig. 76. The representative kinetic curves and dose response curve of α-amylase and α-glucosidase in the presence of luteolin (1h), its derivatives, biflavone (2h) and triflavone (3h) (n = 3). a, b, e, f, i and j show the starch hydrolysis activity of flavonoids with α-amylase while c, d, g, h, k and l show the starch hydrolysis activity of flavonoids with α-glucosidase. Data are presented as mean ± SD.

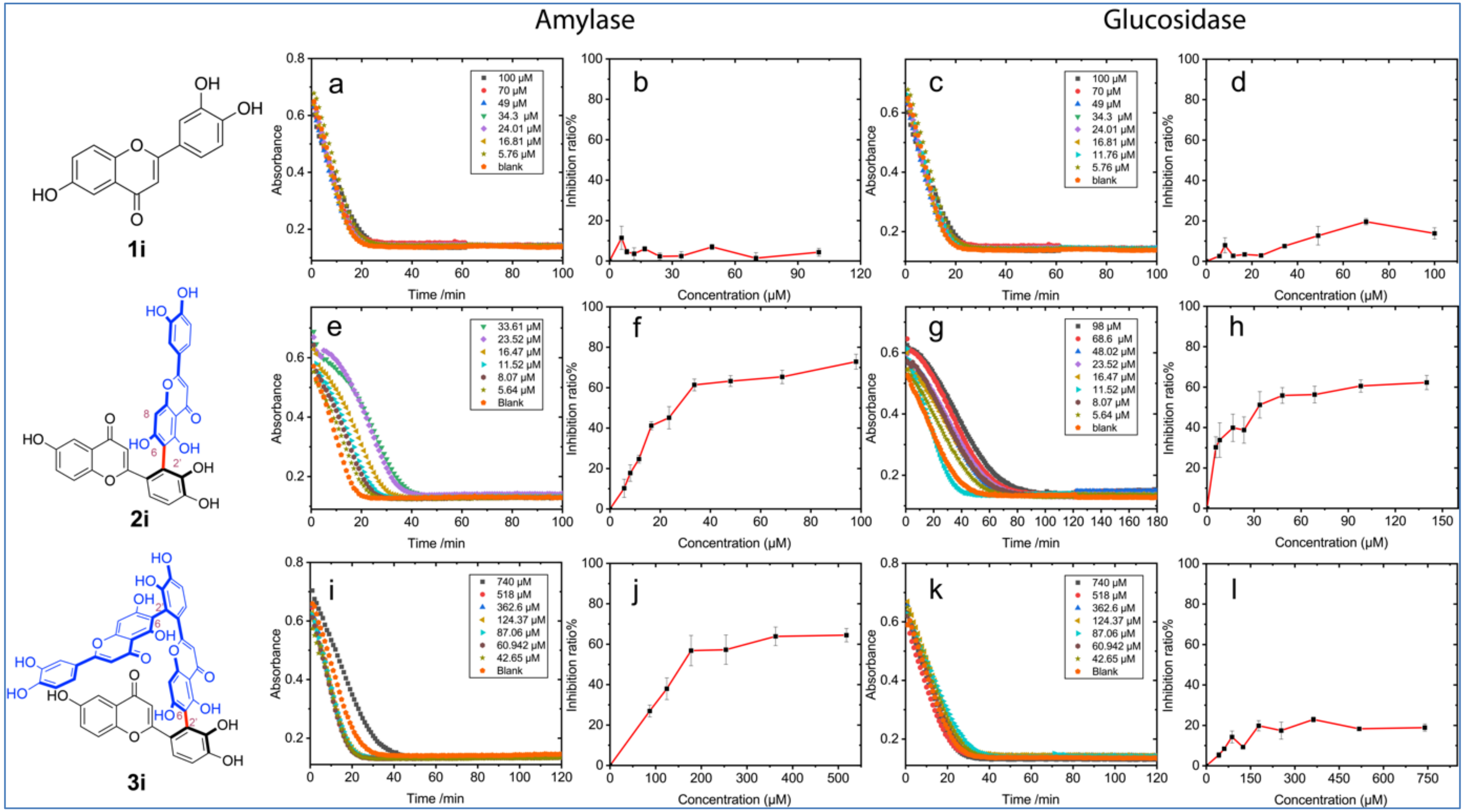

Supplementary Fig. 77. The representative kinetic curves and dose response curve of α-amylase and α-glucosidase in the presence of luteolin (1i), its derivatives, biflavone (2i) and triflavone (3i) (n = 3). a, b, e, f, i and j show the starch hydrolysis activity of flavonoids with α-amylase while c, d, g, h, k and l show the starch hydrolysis activity of flavonoids with α-glucosidase. Data are presented as mean ± SD.

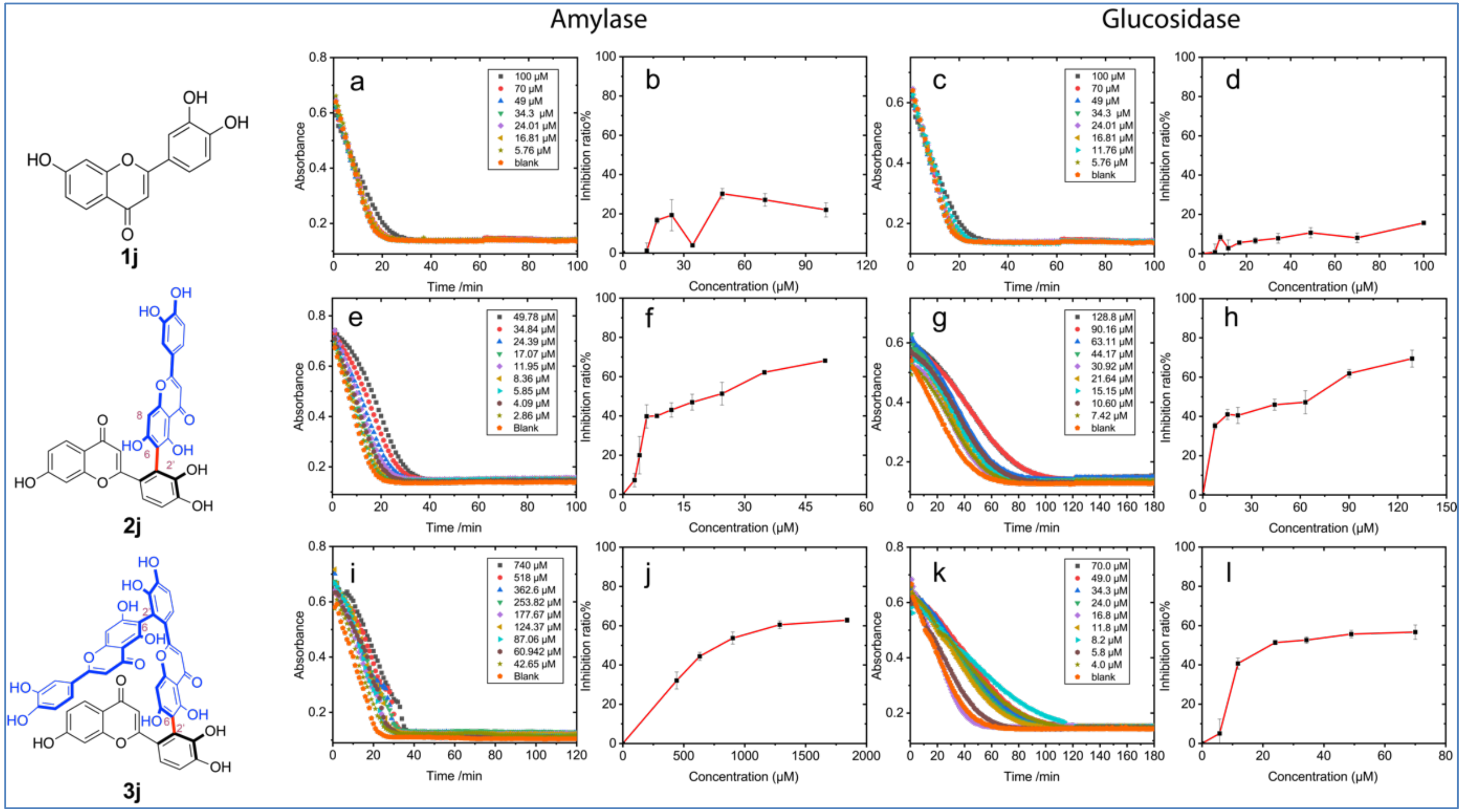

Supplementary Fig. 78. The representative kinetic curves and dose response curve of α-amylase and α-glucosidase in the presence of luteolin (1j), its derivatives, biflavone (2j) and triflavone (3j) (n = 3). a, b, e, f, i and j show the starch hydrolysis activity of flavonoids with α-amylase while c, d, g, h, k and l show the starch hydrolysis activity of flavonoids with α-glucosidase. Data are presented as mean ± SD.

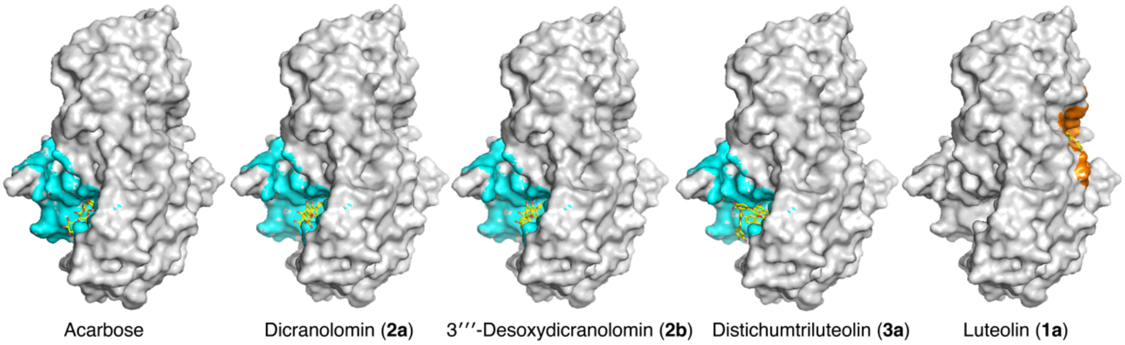

Supplementary Fig. 79. Visualization of the docking result between pancreatic alpha-amylase and ligands. acarbose, dicranolomin (**2a**), 3′′′-desoxydicranolomin (**2b**), luteolin (**1a**) and distichumtriluteolin (**3a**). The cyan part represents the active sites while orange part represents the binding site for luteolin and distichumtriluteolin.

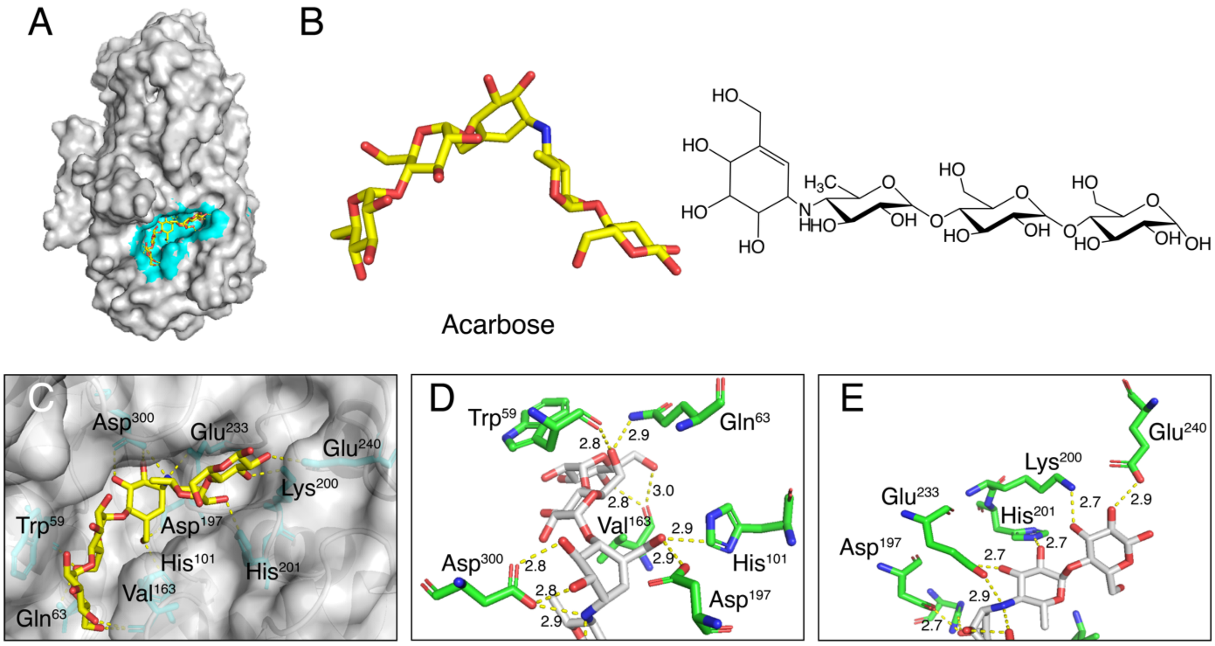

Supplementary Fig. 80. Visualization of the docking result between pancreatic ALPHA-amylase and ligands: acarbose. (**A**) The crystal structure of dicranolomin in complex with α-amylase [Protein Data Bank (PDB) 1ppi]. The pocket was highlighted in cyan while rest in light gray. (**B**) Acarbose is shown in a ball-and-stick representation. (**C**) A magnified view of acarbose binding sites in the enzyme is shown with the C and O atoms of acarbose in yellow and red respectively. The polar interactions in the acarbose-1ppi complex, with the interactions of acarbose (**D**) and (**E**) depicted as black dotted lines and measured in Å.

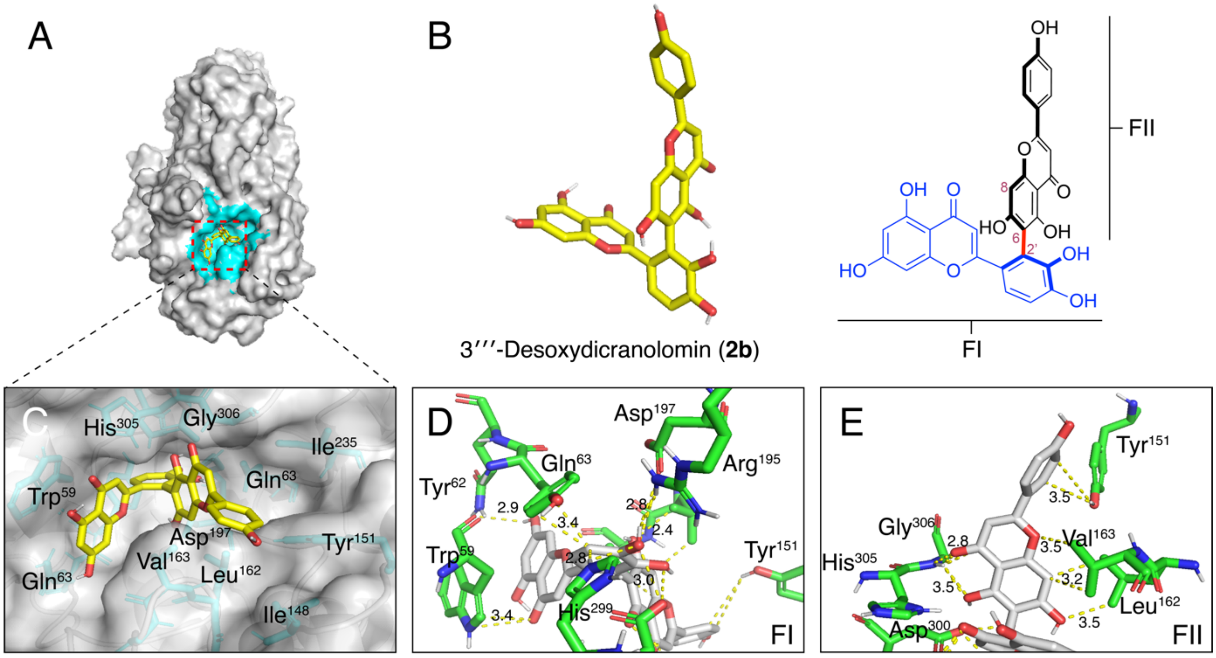

Supplementary Fig. 81. Visualization of the docking result between amylase and ligands: desoxydicranolomin (**2b**). (**A**) The crystal structure of desoxydicranolomin (**2b**) in complex with α-amylase [Protein Data Bank (PDB) 1ppi]. The pocket was highlighted in cyan while rest in light grey. (**B**) desoxydicranolomin (**2b**) is shown in a ball-and-stick representation. (**C**) A magnified view of acarbose binding sites in the enzyme is shown with the C and O atoms of acarbose in yellow and red respectively. The polar interactions in the desoxydicranolomin (**2b**)-1ppi complex, with the interactions of 3‴-desoxydicranolomin (**2b**) (**D**) and (**E**) depicted as black dotted lines and measured in Å

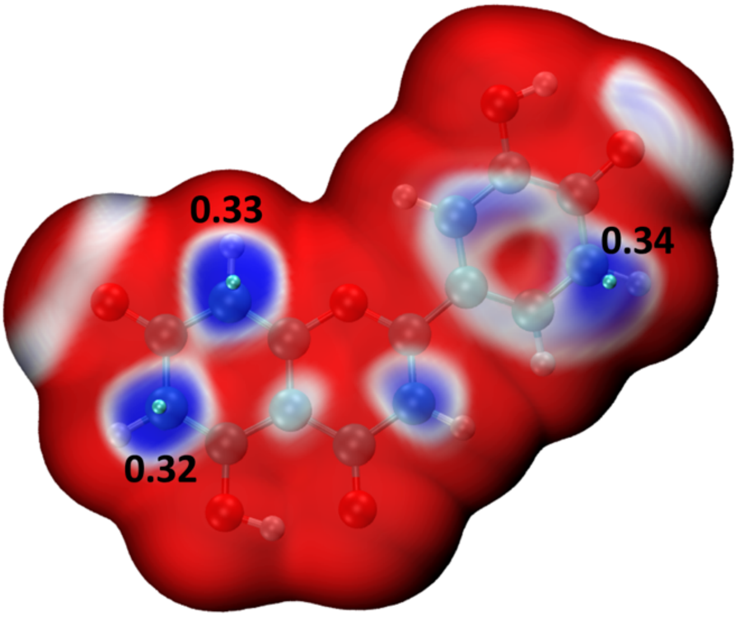

Supplementary Fig. 82. **The computed surface of averaged local ionization energy (ALIE) of LuH$_2^{2-}$**. The cyan spheres correspond to surface minimum of ALIE while the blue color stands for the regions having relatively low ALIE value.

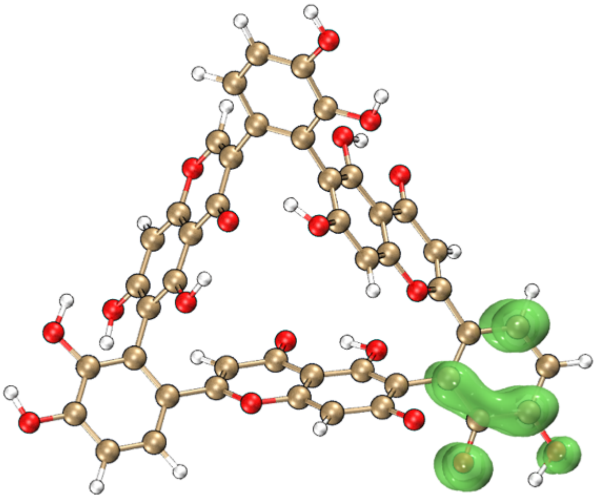

Supplementary Fig. 83. **Spin-density distribution in CTL radical predicted with DFT (UM062x/6-311+G(d,p)).** Isovalue of density = 0.0004.

1.4 Supplementary Tables

Supplementary Table 1. Condition evaluation of aerobic oxidative luteolin-luteolin cross-coupling[a].

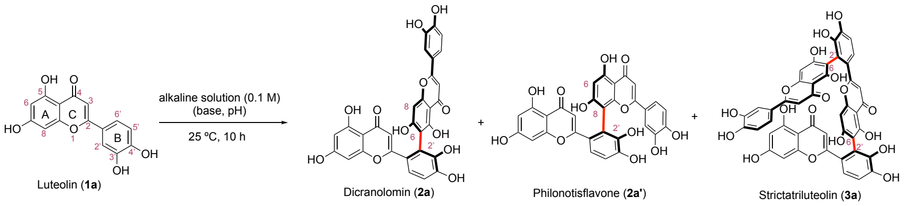

Entry	Base	pH	Atmosphere	Conversion (%)	Yield of **2a** + **2a′** (%)[b]	Ratio of (**2a**:**2a′**)	Yield of **3a** (%)[b]
1	KOH	11.5	Air	76	59	21:1	14
2	Alkaline water (Food grade)	11.5	Air	70	51	20:1	5
3	KOH	9.0	Air	25	22	-	0
4	KOH	9.5	Air	50	40	17:1	6
5	KOH	10.0	Air	53	45	13:1	8
6	KOH	10.5	Air	60	49	20:1	10
7	KOH	11.0	Air	69	52	22:1	13
8	KOH	12.0	Air	70	43	22:1	18
9	KOH	12.5	Air	58	37	17:1	12
10	KOH	13.0	Air	41	23	10:1	6
11	KOH	13.5	Air	26	10	-	0
12	KOH	14.0	Air	11	0	-	0
13	LiOH	11.5	Air	49	41	14:1	5
14	NaOH	11.5	Air	61	49	8:1	6
15	CsOH	11.5	Air	66	51	4:1	9
16	Buffer	11.5	Air	60	50	10:1	7
17	KOH	11.5	Ar	0	0	-	0
18	KOH	11.5	O_2	100	0	-	0
19[c]	KOH	11.5	Air	85	52	15:1	12
20[d]	KOH	11.5	Air	71	55	20:1	11

[a]The reaction condition: luteolin **1a** (0.045 mmol) were dissolved in 3 mL base solution, incubated in sealed tube 15 mL without stir. [b] Determined by HPLC analysis (isocratic elution method (71.5 % of mobile phase A: DI water with 0.1% formic acid and 21.5% mobile phase B: ACN with 0.1% formic acid). Standard curves were built using isolated products. [c]At 10-gram scale. [d]Reaction conducted in dark.

Supplementary Table 2. Condition evaluation of aerobic oxidative luteolin-apigenin cross-coupling [a]

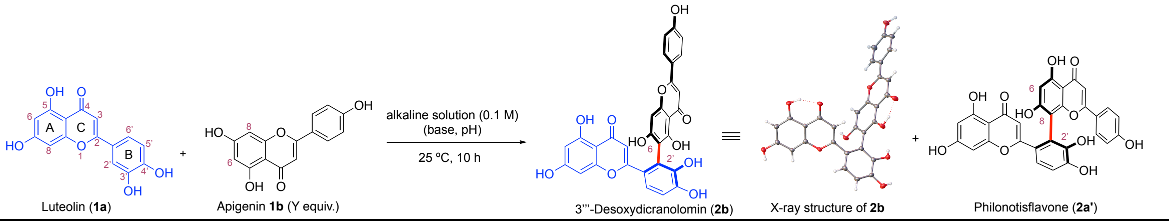

Entry	Y	pH	Conversion (%)[a] 1a	Conversion (%)[a] 1b	2a Yield (%)[b]	2b+2b' Yield (%)[b]	Ratio (2b/2b')	3 Yield (%)[b]	Ratio (3b/3a)
1	1.0	13.5	97	40	5	1	13:1	1	-
2	1.0	12.5	95	46	28	27	16:1	9	3:1
3	1.0	11.5	91	38	31	24	21:1	6	2:1
4	1.0	10.5	82	30	16	16	14:1	3	1:1
5	1.0	9.5	59	27	15	7	13:1	2	-
6	1.0	8.5	27	17	2	1	18:1	1	-
7	1.2	12.5	89	52	20	37	18:1	7	2:1
**8**	**1.5**	**12.5**	**92**	**65**	**13**	**51 (47[c])**	**13:1**	**10**	**3:1**
9	2.0	12.5	92	66	11	50	10:1	12	1:1
10[d]	1.5	12.5	90	50	11	35	12:1	10	1:1

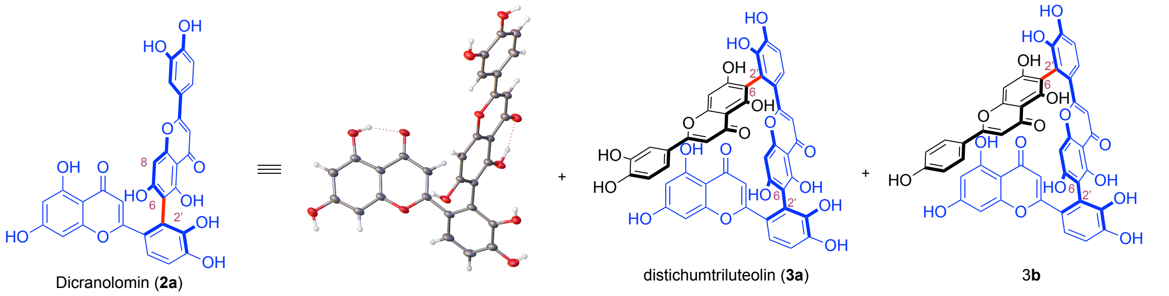

[a]The reaction condition: luteolin **1a** (0.045 mmol) and apigenin **1b** (Y equiv.) were dissolved in 3 mL KOH solution, incubated in sealed tube 15 mL without stir. . [b] Determined by HPLC analysis (isocratic elution method (71.5 % of mobile phase A: DI water with 0.1% formic acid and 21.5% mobile phase B: ACN with 0.1% formic acid). Standard curves were built using isolated products. [c]Isolated yield. [d]pH 12.5 buffer (disodium tetraborate) obtained from Sigma was applied to take place of KOH solution.

Supplementary Table 3. Hyperfine coupling constants (a_{Hn}) of the protons, linewidth (LW) and center field (CF) for flavonoid radicals. The spectra were simulated by JEOL IsoSimu/Fa Version 2.2.0 isotropic simulation program.

Name	pH	G value	a_{H1}	a_{H2}	a_{H3}	a_{H3}	LW/mT	CF/mT
3′,4′-dihydroxyflavone	10	2.0002	0.262	0.124	0.124	0.098	0.02	328.348
5,3′,4′-trihydroxyflavone	11	2.0002	0.286	0.165	0.148	0.134	0.02	328.348
6,3′,4′-trihydroxyflavone	11	2.0002	0.280	0.115	0.110	0.100	0.02	328.348
7,3′,4′-trihydroxyflavone	11	2.0002	0.286	0.132	0.105	1.100	0.02	328.348
$LH^{2-\cdot}$	11.5	2.0002	0.285	0.146	0.111	0.100	0.02	328.348
$[LH^{2-}+O_2]^{\cdot}$	11.5	2.00025	0.43	0.05				328.350
$LH^{2-\cdot}$	12.5	2.0004	0.505	0.084	0.1			
Dicranolomin	11.5	2.0002	0.285	0.146	0.111	0.100	0.02	328.348
		2.00025	0.43	0.07	0.015			

1.5 X-Ray Crystallographic Data

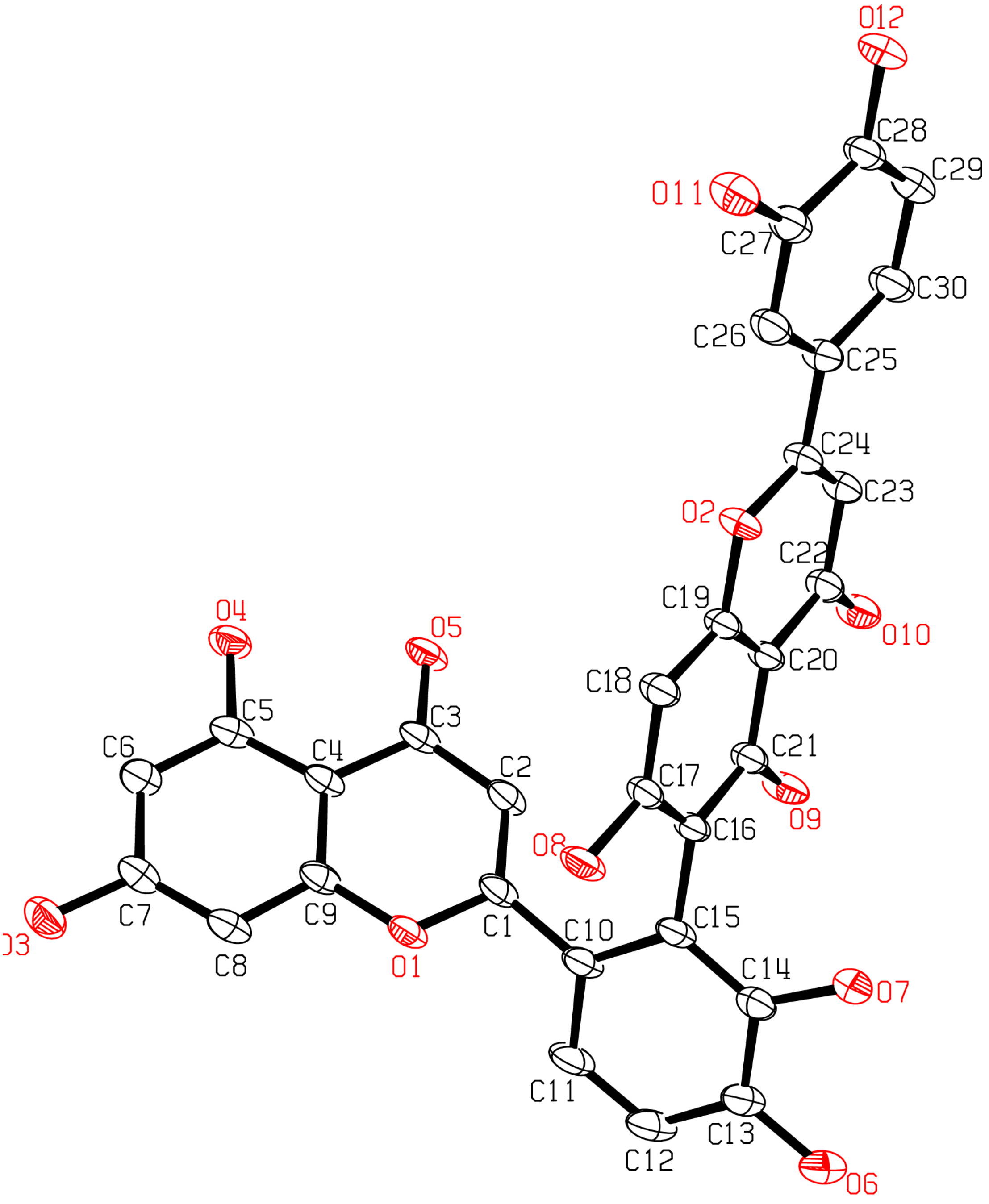

Supplementary Fig. 84. **ORTEP plot of 2a.**

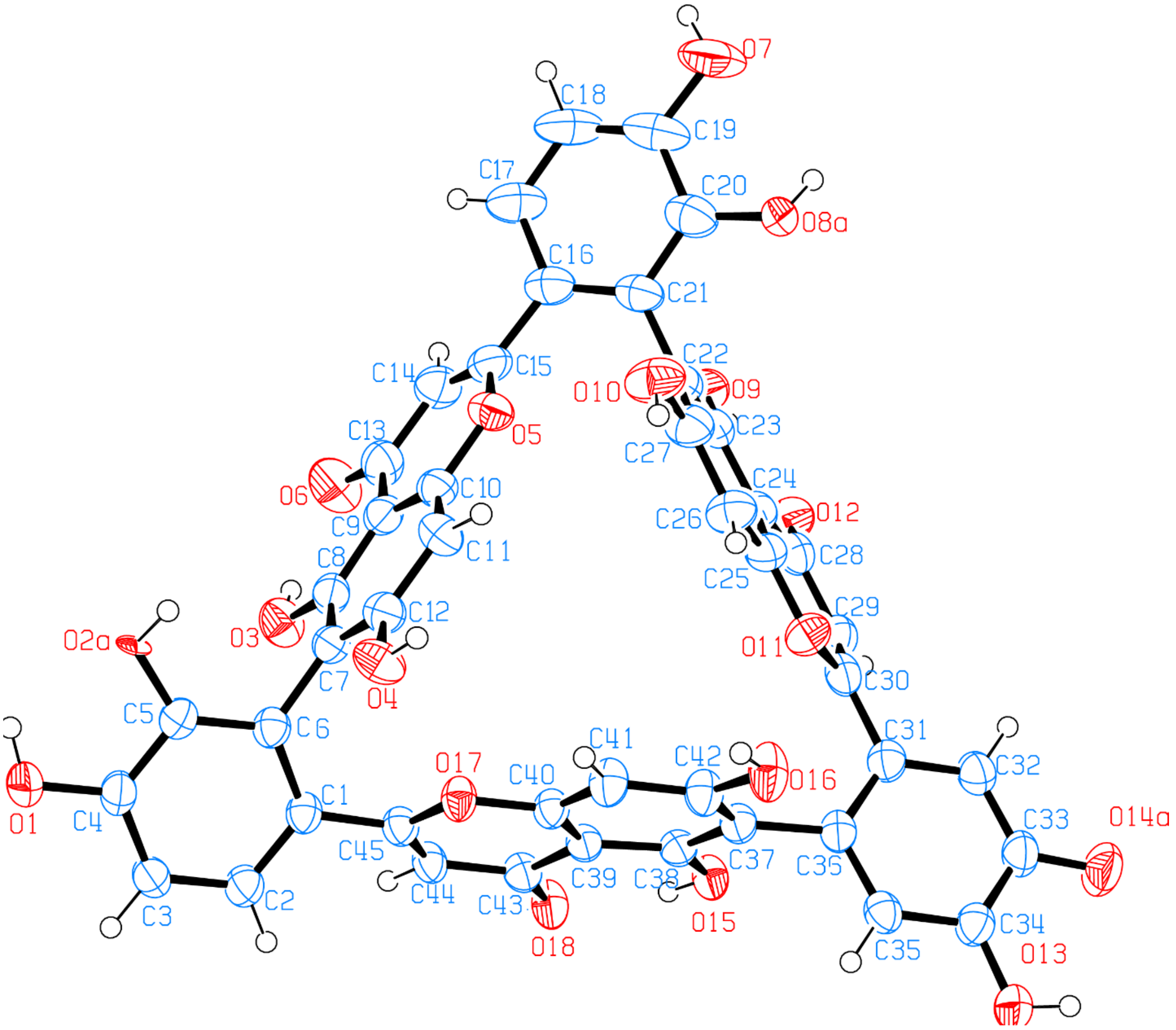

Supplementary Fig. 85. ORTEP plot of 4a′

Dicranolomin (**2a**): suitable for X-ray analysis were obtained by slow evaporation from MeOH. A specimen of $C_{34}H_{32}O_{15}$, approximate dimensions 0.072 mm × 0.123 mm × 0.146 mm, was used for the X-ray crystallographic analysis. The X-ray intensity data were measured (λ = 0.71073 Å). The total exposure time was 5.41 hours. The frames were integrated with the Bruker SAINT software package using a narrow-frame algorithm. The integration of the data using a triclinic unit cell yielded a total of 26930 reflections to a maximum θ angle of 28.31° (0.75 Å resolution), of which 7389 were independent (average redundancy 3.645, completeness = 99.5%, R_{int} = 3.10%, R_{sig} = 2.95%) and 5796 (78.44%) were greater than 2σ (F^2). The final cell constants of a = 8.4524(4) Å, b = 10.0982(4) Å, c = 18.4738(8) Å, α = 95.392(2)°, β = 101.343(2)°, γ = 102.776(2)°, volume = 1492.08(11) $Å^3$, are based upon the refinement of the XYZ-centroids of 9938 reflections above 20σ (I) with 5.070° < 2θ < 56.58°. Data were corrected for absorption effects using the Multi-Scan method (SADABS). The ratio of minimum to maximum apparent transmission was 0.920. The

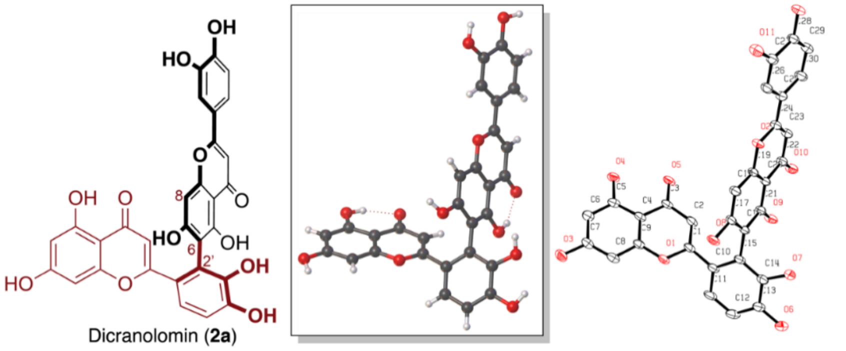

calculated minimum and maximum transmission coefficients (based on crystal size) are 0.6862 and 0.7457.

The structure was solved and refined using the Bruker SHELXTL Software Package, using the space group *P*-1, with Z = 2 for the formula unit, $C_{30}H_{18}O_{12}$. The final anisotropic full-matrix least-squares refinement on F^2 with 543 variables converged at R_1 = 6.80%, for the observed data and wR_2 = 22.47% for all data. The goodness-of-fit was 0.848. The largest peak in the final difference electron density synthesis was 0.564 $e^-/Å^3$ and the largest hole was -0.685 $e^-/Å^3$ with an RMS deviation of 0.082 $e^-/Å^3$. On the basis of the final model, the calculated density was 1.515 g/cm^3 and F(000), 712 e^-.

Crystallographic data have been deposited with the Cambridge Crystallographic Data Centre (CCDC#2044714). Copies of the data can be obtained free of charge on application to the CCDC, 12 Union Road, Cambridge CB21EZ, UK (fax: (+44)-1223-336-033; e-mail: deposit@ccdc.cam.ac.uk).

(3′′′-Desoxydicranolomin, **2b**: Crystals of compound **2b** (3′′′-Desoxydicranolomin): suitable for X-ray analysis were obtained by slow evaporation from MeOH. A

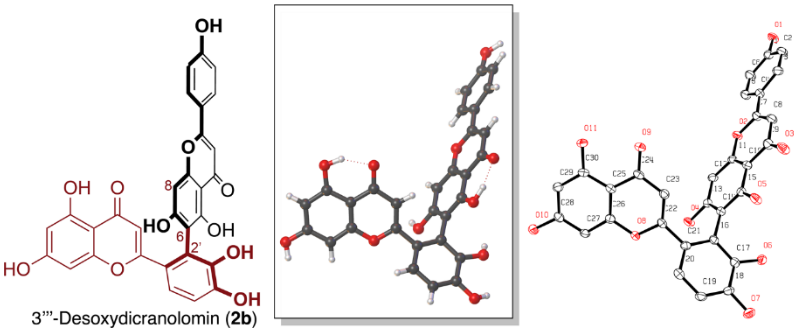

3′′′-Desoxydicranolomin (**2b**)

brown Block-like specimen of $C_{36}H_{32}O_{14}$, approximate dimensions 0.061 mm × 0.063 mm × 0.267 mm, was used for the X-ray crystallographic analysis. The X-ray intensity data were measured. The total exposure time was 19.00 hours. The frames were integrated with the Bruker SAINT software package using a narrow-frame algorithm. The integration of the data using a triclinic unit cell yielded a total of 18025 reflections to a maximum θ angle of 67.11° (0.84 Å resolution), of which 5625 were independent (average redundancy 3.204, completeness = 99.0%, R_{int} = 6.56%, R_{sig} = 6.46%) and 3788 (67.34%) were greater than 2σ (F^2). The final cell constants of a = 8.4765 Å, b = 11.1018 Å, c = 18.6315 Å, α = 98.306°, β = 95.037°, γ = 109.024°, volume = 1623.0 Å^3, are based upon the refinement of the XYZ-centroids of 49 reflections above 20 σ(I) with 8.533° < 2θ < 40.75°. Data were corrected for absorption effects using the Multi-Scan method (SADABS). The ratio of minimum to maximum apparent transmission was 0.883. The structure was solved and refined using the

Bruker SHELXTL Software Package, with Z = 2 for the formula unit, $C_{30}H_{18}O_{11}$. The final anisotropic full-matrix least-squares refinement on F^2 with 491variables converged at R_1 = 4.71%, for the observed data and wR_2 = 12.43% for all data. The goodness-of-fit was 1.029. The largest peak in the final difference electron density synthesis was 0.253 $e^-/Å^3$ and the largest hole was -0.240 $e^-/Å^3$ with an RMS deviation of 0.056 $e^-/Å^3$. On the basis of the final model, the calculated density was 1.434 g/cm^3 and F(000), 720 e^-.

Lu-Wo (2′-6) dimer, 2e: suitable for X-ray analysis were obtained by slow evaporation from

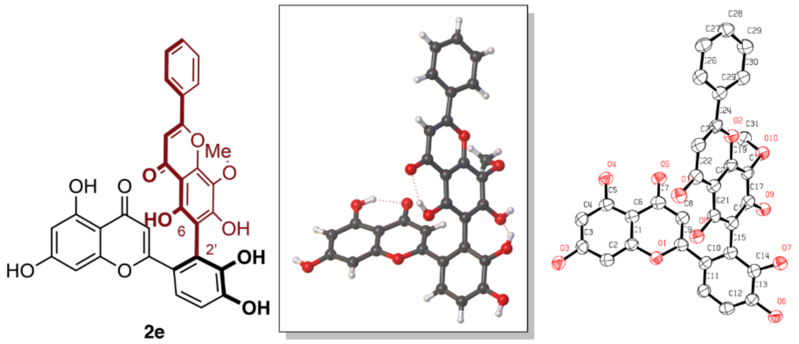

MeOH and acetonitrile. A specimen of formula $C_{35}H_{26}N_2O_{11}$ (containing two CH_3CN) and approximate dimensions 0.049 mm × 0.122 mm × 0.132 mm, was used for the X-ray crystallographic analysis. The crystal is non-merohedral twin. Twin refinement was performed with BASF= 0.43461. The X-ray intensity data were measured (λ = 1.54178 Å). The total exposure time was 17.13 hours. The frames were integrated with the Bruker SAINT software package using a narrow-frame algorithm. The integration of the data using a triclinic unit cell yielded a total of 8055 reflections to a maximum θ angle of 67.03° (0.84 Å resolution), of which 8055 were independent (average redundancy 1.000, completeness = 97.6%, R_{sig} = 10.51%) and 4577 (56.82%) were greater than $2\sigma(F^2)$. The final cell constants of a = 8.6271(6) Å, b = 10.2328(6) Å, c = 17.4619(12) Å, α = 102.701(4)°, β = 102.737(4)°, γ = 90.020(4)°, volume = 1464.87(17) Å^3, are based upon the refinement of the XYZ-centroids of 4286 reflections above 20 $\sigma(I)$ with 8.870° < 2θ < 133.1°. Data were corrected for absorption effects using the Multi-Scan method (SADABS). The ratio of minimum to maximum apparent

transmission was 0.637. The calculated minimum and maximum transmission coefficients (based on crystal size) are 0.4799 and 0.7528. The structure was solved and refined using the Bruker SHELXTL Software Package, using the space group *P* -1, with Z = 2 for the formula unit, $C_{35}H_{26}N_2O_{11}$. The final anisotropic full-matrix least-squares refinement on F^2 with 455 variables converged at R_1 = 9.67%, for the observed data and wR_2 = 32.15% for all data. The goodness-of-fit was 1.065. The largest peak in the final difference electron density synthesis was 0.495 e-/$Å^3$ and the largest hole was -0.499 e-/$Å^3$ with an RMS deviation of 0.104 e-/$Å^3$. On the basis of the final model, the calculated density was 1.475 g/cm^3 and F(000), 676 e-.

Cyclotriluteolin, 4a′: Suitable for X-ray analysis were obtained by slow evaporation from MeOH. A specimen of $C_{48}H_{42}O_{24}$ (containing three methanol and three water but 3 hydrogen cannot be located) approximate dimensions 0.248 × 0.247 × 0.168 mm, was used for the X-ray crystallographic analysis. The X-ray intensity data were measured (λ = 1.54178 Å). The frames were integrated with the Bruker SAINT software package using a narrow-frame algorithm. The integration of the data using a triclinic unit cell yielded a total of 33495 reflections. The final cell constants of a = 10.4383(7) Å, b = 16.9456(11) Å, c = 18.4841(12) Å, α = 116.927(3)°, β = 97.234(3)°, γ = 100.043(3)°, volume = 2791.1(3) $Å^3$, are based upon the refinement of the XYZ-centroids of 3609 reflections above 20 $\sigma(I)$ with 4.395° < 2θ < 51.36°. Data were corrected for absorption effects using the Multi-Scan method (SADABS). The calculated minimum and maximum transmission coefficients (based on crystal size) are 0.5865 and 0.7528. The structure was solved and refined using the Bruker SHELXTL Software Package, using the space group *P* -1, with Z = 2 for the formula unit, $C_{48}H_{42}O_{24}$. The final anisotropic full-matrix least-squares refinement on

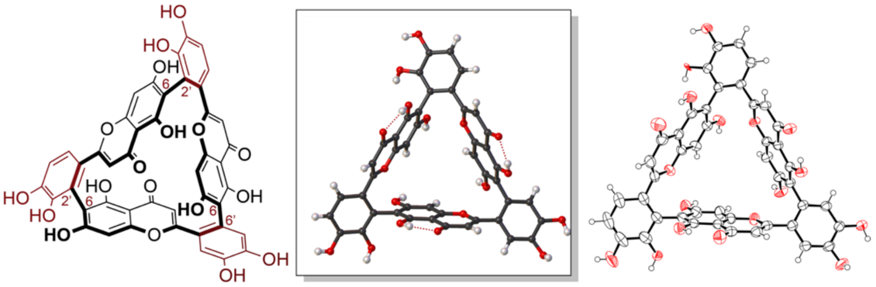

F^2 at R_1 = 7.66%, for the observed data and wR_2 = 23.48% for all data. The goodness-of-fit was 1.078. The largest peak in the final difference electron density synthesis was 0.934 $e^-/Å^3$ and the largest hole was -0.611 $e^-/Å^3$. On the basis of the final model, the calculated density was 1.190 g/cm^3 and F(000), 1038 e^-. The lattice contained the weak and diffused residue peaks due to partially occupied or disordered water and methanol molecules which could not be modelled satisfactorily. Hence Platon Program Squeezed was used for these solvent regions.

Supplementary Table 4. Sample and crystal data for **2a** (dicranolomin).

**Identification code**	J459
**Chemical formula**	$C_{34}H_{32}O_{15}$
**Formula weight**	680.59 g/mol
**Temperature**	100(2) K
**Wavelength**	0.71073 Å
**Crystal size**	0.072 × 0.123 × 0.146 mm
**Crystal system**	triclinic
**Space group**	*P*-1
**Unit cell dimensions**	a = 8.4524(4) α = 95.392(2)°
	b = 10.0982(4) Å β = 101.343(2)°
	c = 18.4738(8) Å. γ = 102.776(2)°
**Volume**	1492.08(11) $Å^3$
**Z**	2
**Density (calculated)**	1.515 g/cm^3

**Absorption coefficient**	0.120 mm^{-1}
**F(000)**	712

Supplementary Table 5. Data collection and structure refinement for **2a** (Dicranolomin).

**Theta range for data collection**	2.27 to 28.31°
**Index ranges**	-11<=h<=11, -13<=k<=13, -23<=l<=24
**Reflections collected**	26930
**Independent reflections**	7389 [R(int) = 0.0310]
**Coverage of independent reflections**	99.50%
**Absorption correction**	Multi-Scan
**Max. and min. transmission**	0.7457 and 0.6862
**Structure solution technique**	Dual space
**Structure solution program**	SHELXS-97 (Sheldrick 2008)
**Refinement method**	Full-matrix least-squares on F^2
**Refinement program**	SHELXL-2017/1 (Sheldrick, 2017)
**Function minimized**	$\Sigma w(F_o^2 - F_c^2)^2$
**Data / restraints / parameters**	7389 / 201 / 543
**Goodness-of-fit on F^2**	0.848
**Final R indices**	5796 data; I > 2σ(I). R_1 = 0.0680, wR_2 = 0.2030
	all data. R_1 = 0.0842, wR_2 = 0.2247
**Weighting scheme**	$w=1/[\sigma^2(F_o^2)+(0.1602P)^2+2.3495P]$, where $P=(F_o^2+2F_c^2)/3$
**Largest diff. peak and hole**	0.564 and -0.685 eÅ^{-3}
**R.M.S. deviation from mean**	0.082 eÅ^{-3}

Supplementary Table 6. Atomic coordinates and equivalent isotropic atomic displacement parameters (Å^2) for J459. U(eq) is defined as one third of the trace of the orthogonalized Uij tensor.

	**x/a**	**y/b**	**z/c**	**U(eq)**
O1	0.6880(2)	0.54829(17)	0.93504(8)	0.0261(4)
O2	0.3249(2)	0.16316(17)	0.57186(9)	0.0270(4)
O3	0.8008(3)	0.3373(2)	0.15090(10)	0.0365(4)
O4	0.9956(2)	0.19908(19)	0.94177(10)	0.0309(4)
O5	0.9301(2)	0.32086(18)	0.82169(9)	0.0296(4)
O6	0.4761(3)	0.01630(19)	0.76816(11)	0.0399(5)
O7	0.3747(2)	0.78006(18)	0.67850(9)	0.0321(4)
O8	0.3012(2)	0.43405(18)	0.79121(9)	0.0307(4)
O9	0.6645(2)	0.62360(18)	0.64458(10)	0.0318(4)
O10	0.6823(2)	0.46963(18)	0.52835(10)	0.0313(4)
O11	0.9960(2)	0.69871(19)	0.46181(10)	0.0330(4)
O12	0.0580(2)	0.64909(18)	0.32356(9)	0.0301(4)
C1	0.7020(3)	0.5660(2)	0.86405(12)	0.0255(5)
C2	0.7791(3)	0.4907(3)	0.82484(12)	0.0270(5)
C3	0.8537(3)	0.3889(2)	0.85668(12)	0.0249(5)
C4	0.8398(3)	0.3721(2)	0.93233(12)	0.0239(4)
C5	0.9099(3)	0.2785(2)	0.97257(13)	0.0260(5)
C6	0.8937(3)	0.2675(3)	0.04506(13)	0.0282(5)
C7	0.8086(3)	0.3507(3)	0.07924(12)	0.0277(5)
C8	0.7381(3)	0.4435(3)	0.04181(12)	0.0268(5)
C9	0.7567(3)	0.4534(2)	0.96940(12)	0.0244(4)
C10	0.6333(3)	0.6796(2)	0.83888(12)	0.0268(5)
C11	0.6821(3)	0.8062(3)	0.88530(13)	0.0334(5)
C12	0.6324(3)	0.9183(3)	0.86118(15)	0.0359(6)
C13	0.5283(3)	0.9057(3)	0.79200(14)	0.0311(5)
C14	0.4761(3)	0.7790(2)	0.74513(12)	0.0269(5)

C15	0.5300(3)	0.6652(2)	0.76771(12)	0.0244(4)
C16	0.4773(3)	0.5326(2)	0.71596(11)	0.0234(4)
C17	0.3633(3)	0.4188(2)	0.72937(12)	0.0245(4)
C18	0.3135(3)	0.2942(2)	0.68160(12)	0.0260(5)
C19	0.3820(3)	0.2849(2)	0.61963(12)	0.0238(4)
C20	0.5003(3)	0.3925(2)	0.60528(11)	0.0242(4)
C21	0.5472(3)	0.5179(2)	0.65500(12)	0.0241(4)
C22	0.5703(3)	0.3755(2)	0.54107(12)	0.0255(4)
C23	0.5019(3)	0.2476(2)	0.49326(12)	0.0275(5)
C24	0.3820(3)	0.1487(2)	0.50862(12)	0.0256(5)
C25	0.2982(3)	0.0175(2)	0.46029(12)	0.0268(5)
C26	0.1829(3)	0.9182(2)	0.48360(12)	0.0273(5)
C27	0.1044(3)	0.7944(2)	0.43743(13)	0.0266(5)
C28	0.1397(3)	0.7713(2)	0.36659(12)	0.0256(5)
C29	0.2549(3)	0.8695(3)	0.34419(13)	0.0311(5)
C30	0.3352(3)	0.9917(3)	0.39053(13)	0.0316(5)
O1W	0.8349(2)	0.42752(19)	0.41621(10)	0.0330(4)
O1X	0.2305(4)	0.1955(2)	0.83997(13)	0.0556(6)
C1X	0.3640(4)	0.1948(3)	0.90082(19)	0.0507(8)
C2X	0.3334(8)	0.0640(5)	0.9320(2)	0.0876(18)
O1V	0.0876(18)	0.9899(15)	0.7177(9)	0.0700(10)
C1V	0.964(3)	0.8985(19)	0.7423(16)	0.0699(10)
C2V	0.989(3)	0.7675(17)	0.7598(12)	0.0700(10)
O1Y	0.1185(8)	0.9155(7)	0.7888(4)	0.0700(10)
C1Y	0.9801(14)	0.8532(12)	0.7349(6)	0.0700(10)
C2Y	0.9990(13)	0.8583(11)	0.6584(5)	0.0697(10)
O1U	0.1232(11)	0.9739(10)	0.6781(6)	0.0700(11)
C1U	0.0305(17)	0.9076(15)	0.7117(7)	0.0699(10)
C2U	0.9353(17)	0.8677(16)	0.7651(8)	0.0699(10)

Supplementary Table 7. Sample and crystal data for **2b** ($3'''$-desoxydicranolomin).

**Identification code**	J456
**Chemical formula**	$C_{36}H_{32}O_{14}$
**Formula weight**	688.61 g/mol
**Temperature**	100(2) K
**Wavelength**	1.54184 Å
**Crystal size**	0.267 × 0.063 × 0.061 mm
**Crystal system**	brown Block
**Space group**	triclinic
**Unit cell dimensions**	a = 8.4307(5)Å. α = 98.338(4)°
	b = 11.0300(6) Å. β = 95.059(4)°
	c = 18.5298(10) Å. γ = 108.976(4)°.
**Volume**	1595.28(16) $Å^3$
**Z**	2
**Density (calculated)**	1.434 g/cm^3
**Absorption coefficient**	0.942 mm^{-1}
**F(000)**	720

Supplementary Table 8. Data collection and structure refinement for **2b** (3‴-Desoxydicranolomin).

**Theta range for data collection**	2.44 to 67.11°
**Index ranges**	-9<=h<=10, -13<=k<=12, -22<=l<=22
**Reflections collected**	18025
**Independent reflections**	5625 [R(int) = 0.0656]
**Coverage of independent reflections**	99.00%
**Absorption correction**	Multi-Scan
**Structure solution technique**	Dual space
**Structure solution program**	XT, VERSION 2014/5
**Refinement method**	Full-matrix least-squares on F^2
**Refinement program**	SHELXL-2016/6 (Sheldrick, 2016)
**Function minimized**	$\Sigma w(F_o^2 - F_c^2)^2$
**Data / restraints / parameters**	5625 / 0 / 491
**Goodness-of-fit on F^2**	1.029
**Final R indices**	3788 data; $I > 2\sigma(I)$. $R_1 = 0.0471$, $wR_2 = 0.1063$
	all data. $R_1 = 0.0798$, $wR_2 = 0.1243$
**Weighting scheme**	$w = 1/[\sigma^2(F_o^2)+(0.0578P)^2+0.0599P]$, where $P = (F_o^2+2F_c^2)/3$
**Largest diff. peak and hole**	0.253 and -0.240 eÅ^{-3}
**R.M.S. deviation from mean**	0.056 eÅ^{-3}

Supplementary Table 9. Atomic coordinates and equivalent isotropic atomic displacement parameters (Å^2) for J456 (**2b**). U(eq) is defined as one third of the trace of the orthogonalized Uij tensor.

	**x/a**	**y/b**	**z/c**	**U(eq)**
O1	0.9467(2)	0.33636(16)	0.67274(11)	0.0269(4)
O2	0.6535(2)	0.83317(15)	0.42898(9)	0.0214(4)
O3	0.3037(3)	0.52146(16)	0.48655(10)	0.0309(5)
O4	0.6755(2)	0.58067(16)	0.20405(9)	0.0236(4)
O5	0.3117(2)	0.38040(15)	0.36460(10)	0.0260(4)
O6	0.5785(2)	0.25212(16)	0.30762(10)	0.0229(4)
O7	0.4776(3)	0.02891(16)	0.20906(12)	0.0287(5)
O8	0.2971(2)	0.44829(15)	0.06509(9)	0.0200(4)
O9	0.0822(2)	0.67766(15)	0.18511(9)	0.0237(4)
O10	0.2263(2)	0.62713(17)	0.85267(10)	0.0255(4)
O11	0.0483(2)	0.78418(16)	0.06829(10)	0.0243(4)
C1	0.8532(3)	0.2193(2)	0.62904(14)	0.0227(6)
C2	0.7167(4)	0.1303(2)	0.65196(15)	0.0249(6)
C3	0.6306(4)	0.0108(2)	0.60738(15)	0.0245(6)
C4	0.6780(3)	0.9771(2)	0.53900(14)	0.0212(6)
C5	0.8101(4)	0.0697(2)	0.51523(15)	0.0252(6)
C6	0.8973(4)	0.1896(2)	0.55990(15)	0.0283(6)
C7	0.5950(3)	0.8454(2)	0.49487(14)	0.0214(6)
C8	0.4771(3)	0.7449(2)	0.51503(14)	0.0240(6)
C9	0.4117(3)	0.6186(2)	0.46892(14)	0.0237(6)
C10	0.4772(3)	0.6064(2)	0.39976(13)	0.0191(5)
C11	0.5956(3)	0.7150(2)	0.38172(13)	0.0177(5)
C12	0.6637(3)	0.7106(2)	0.31664(14)	0.0203(5)
C13	0.6111(3)	0.5915(2)	0.26870(14)	0.0194(5)
C14	0.4916(3)	0.4780(2)	0.28277(13)	0.0186(5)
C15	0.4266(3)	0.4869(2)	0.34909(14)	0.0190(5)
C16	0.4335(3)	0.3524(2)	0.22842(13)	0.0179(5)

C17	0.4809(3)	0.2480(2)	0.24422(14)	0.0194(5)
C18	0.4305(3)	0.1300(2)	0.19377(15)	0.0216(6)
C19	0.3323(3)	0.1155(2)	0.12715(14)	0.0234(6)
C20	0.2887(3)	0.2193(2)	0.10934(15)	0.0232(6)
C21	0.3372(3)	0.3372(2)	0.15929(14)	0.0190(5)
C22	0.2800(3)	0.4401(2)	0.13727(13)	0.0183(5)
C23	0.2115(3)	0.5163(2)	0.17812(14)	0.0199(5)
C24	0.1506(3)	0.6080(2)	0.14789(14)	0.0191(5)
C25	0.1701(3)	0.6155(2)	0.07159(13)	0.0187(5)
C26	0.2421(3)	0.5343(2)	0.03218(14)	0.0186(5)
C27	0.2663(3)	0.5357(2)	0.95974(14)	0.0195(5)
C28	0.2139(3)	0.6218(2)	0.92416(14)	0.0204(5)
C29	0.1411(3)	0.7054(2)	0.96083(14)	0.0215(6)
C30	0.1195(3)	0.7031(2)	0.03384(14)	0.0196(5)
O1S	0.7523(3)	0.03701(18)	0.29848(11)	0.0341(5)
C1S	0.9389(4)	0.2276(3)	0.26425(17)	0.0338(7)
C2S	0.8981(4)	0.1064(3)	0.29708(16)	0.0308(7)
C3S	0.0436(4)	0.0718(3)	0.3274(2)	0.0477(9)
O2S	0.8363(3)	0.81958(16)	0.17516(10)	0.0294(5)
C4S	0.8367(4)	0.9840(3)	0.10535(18)	0.0359(7)
C5S	0.7560(4)	0.8598(2)	0.13223(16)	0.0275(6)
C6S	0.5743(4)	0.7894(3)	0.10549(19)	0.0431(8)
O3S	0.8282(3)	0.45074(18)	0.38282(11)	0.0289(5)

Supplementary Table 10. Sample and crystal data for **2e**.

**Identification code**	J357
**Chemical formula**	$C_{35}H_{26}N_2O_{11}$
**Formula weight**	650.58 g/mol
**Temperature**	100(2) K
**Wavelength**	1.54178 Å
**Crystal size**	0.049 × 0.122 × 0.132 mm
**Crystal system**	triclinic
**Space group**	*P*-1
**Unit cell dimensions**	a = 8.6271(6) Å. α = 102.701(4)°
	b = 10.2328(6) Å. β = 102.737(4)°
	c = 17.4619(12) Å. γ = 90.020(4)°
**Volume**	1464.87(17) $Å^3$
**Z**	2
**Density (calculated)**	1.475 g/cm^3
**Absorption coefficient**	0.936 mm^{-1}
**F(000)**	676

Supplementary Table 11. Data collection and structure refinement for **2e**.

**Theta range for data collection**	2.66 to 67.03°
**Index ranges**	-10<=h<=10, -11<=k<=12, -20<=l<=20
**Reflections collected**	8055
**Coverage of independent reflections**	97.60%
**Absorption correction**	Multi-Scan
**Max. and min. transmission**	0.7528 and 0.4799
**Structure solution technique**	Dual space
**Structure solution program**	SHELXS-97 (Sheldrick 2008)
**Refinement method**	Full-matrix least-squares on F^2
**Refinement program**	SHELXL-2017/1 (Sheldrick, 2017)
**Function minimized**	Σ w(F_o^2 - F_c^2)^2
**Data / restraints / parameters**	8055 / 6 / 455
**Goodness-of-fit on F^2**	1.065
**Final R indices**	4577 data; I > 2σ(I). R_1 = 0.0967, wR_2 = 0.2670
	all data. R_1 = 0.1466, wR_2 = 0.3215
**Weighting scheme**	w = 1/[σ^2(F_o^2)+(0.1943P)^2], where P = (F_o^2+2F_c^2)/3
**Largest diff. peak and hole**	0.495 and -0.499 eÅ^{-3}
**R.M.S. deviation from mean**	0.104 eÅ^{-3}

Supplementary Table 12. Atomic coordinates and equivalent isotropic atomic displacement parameters (Å^2) for **2e**. U(eq) is defined as one third of the trace of the orthogonalized Uij tensor.

	**x/a**	**y/b**	**z/c**	**U(eq)**
O1	0.5470(5)	0.1328(4)	0.8847(2)	0.0397(10)
O2	0.2969(5)	0.2518(4)	0.4914(2)	0.0396(10)
O3	0.9576(5)	0.3840(4)	0.0831(3)	0.0467(11)
O4	0.5791(5)	0.6137(4)	0.9259(3)	0.0495(11)
O5	0.3462(5)	0.4678(4)	0.8222(3)	0.0453(11)
O6	0.1452(5)	0.5950(4)	0.7217(3)	0.0442(10)
O7	0.1277(5)	0.7429(4)	0.6147(2)	0.0417(10)
O8	0.5584(5)	0.9941(4)	0.6773(3)	0.0422(10)
O9	0.0045(5)	0.0345(4)	0.6309(3)	0.0410(10)
O10	0.0134(4)	0.2033(4)	0.5284(2)	0.0394(10)
O11	0.7115(5)	0.1071(4)	0.5959(3)	0.0445(10)
C1	0.6189(7)	0.2571(6)	0.9230(3)	0.0393(13)
C2	0.7542(7)	0.2598(6)	0.9823(3)	0.0389(13)
C3	0.8285(7)	0.3834(7)	0.0226(4)	0.0433(14)
C4	0.7718(7)	0.5030(7)	0.0030(4)	0.0436(14)
C5	0.6354(7)	0.4973(6)	0.9430(4)	0.0432(14)
C6	0.5552(7)	0.3740(6)	0.9011(4)	0.0400(14)
C7	0.4091(7)	0.3633(7)	0.8408(4)	0.0430(14)
C8	0.3408(7)	0.2314(6)	0.8062(4)	0.0408(14)
C9	0.4083(7)	0.1222(6)	0.8277(4)	0.0411(14)
C10	0.3445(7)	0.9833(6)	0.7976(4)	0.0368(13)
C11	0.3516(7)	0.9033(6)	0.8534(4)	0.0428(14)
C12	0.2840(7)	0.7746(6)	0.8280(4)	0.0437(14)
C13	0.2097(7)	0.7214(6)	0.7494(4)	0.0385(13)
C14	0.1999(6)	0.8023(6)	0.6926(4)	0.0376(13)
C15	0.2724(7)	0.9314(6)	0.7153(4)	0.0376(13)
C16	0.2804(7)	0.0097(6)	0.6530(3)	0.0375(13)

C17	0.1431(7)	0.0627(6)	0.6140(3)	0.0363(13)
C18	0.1503(7)	0.1472(6)	0.5612(3)	0.0377(13)
C19	0.2958(7)	0.1732(6)	0.5453(3)	0.0381(13)
C20	0.4358(7)	0.1211(6)	0.5822(4)	0.0372(13)
C21	0.4253(7)	0.0401(6)	0.6387(4)	0.0391(13)
C22	0.5833(7)	0.1502(6)	0.5629(4)	0.0394(13)
C23	0.5730(7)	0.2297(6)	0.5036(4)	0.0397(13)
C24	0.4350(7)	0.2787(6)	0.4716(4)	0.0375(13)
C25	0.4122(7)	0.3626(6)	0.4113(3)	0.0386(13)
C26	0.5437(7)	0.4211(6)	0.3950(4)	0.0416(14)
C27	0.5228(8)	0.5009(6)	0.3391(4)	0.0446(14)
C28	0.3726(8)	0.5237(6)	0.2992(4)	0.0445(14)
C29	0.2416(7)	0.4650(7)	0.3144(4)	0.0449(15)
C30	0.2589(7)	0.3852(6)	0.3710(4)	0.0430(14)
C31	0.0048(8)	0.3408(6)	0.5716(4)	0.0464(15)
C32	0.0672(8)	0.7611(8)	0.1792(4)	0.0492(16)
C33	0.0771(9)	0.9054(7)	0.1826(4)	0.0531(17)
C34	0.8008(10)	0.8628(8)	0.9359(6)	0.067(2)
C35	0.7708(9)	0.8023(8)	0.8511(5)	0.0619(19)

Supplementary Table 13. Sample and crystal data for **4a′**.

**Identification code**	J552
**Chemical formula**	$C_{48}H_{42}O_{24}$
**Formula weight**	999.77 g/mol
**Temperature**	100(2) K
**Wavelength**	1.54178 Å
**Crystal size**	0.248 × 0.247 × 0.168 mm
**Crystal system**	triclinic
**Space group**	P -1
**Unit cell dimensions**	a = 10.4383(7) Å. α = 116.927(3)°
	b = 16.9456(11) Å. β = 97.234(3)°
	c = 18.4841(12) Å. γ = 100.043(3)°
**Volume**	2791.1(3) $Å^3$
**Z**	1
**Density (calculated)**	1.190 g/cm^3
**Absorption coefficient**	0.834 mm^{-1}
**F(000)**	1038

Supplementary Table 14. Data collection and structure refinement for **4a′**.

**Theta range for data collection**	2.943 to 66.885°
**Index ranges**	-12<=h<=12, -20<=k<=20, -19<=l<=22
**Reflections collected**	33459
**Coverage of independent reflections**	98.7%
**Absorption correction**	Multi-Scan
**Max. and min. transmission**	0.7528 and 0.5865
**Structure solution technique**	Dual space
**Structure solution program**	SHELXS-97 (Sheldrick 2008)
**Refinement method**	Full-matrix least-squares on F^2
**Refinement program**	SHELXL-2019/1 (Sheldrick, 2019)
**Function minimized**	Σ w(F_o^2 - F_c^2)^2
**Data / restraints / parameters**	9802 / 79 / 758
**Goodness-of-fit on F^2**	1.078
**Final R indices**	R_1 = 0.0766, wR_2 = 0.2303
	all data. R_1 = 0.0806, wR_2 = 0.2348
**Weighting scheme**	w = 1/[σ^2(F_o^2)+(0.1278P)^2+3.0576P], where P = (F_o^2+2F_c^2)/3
**Largest diff. peak and hole**	0.934 and -0.611 eÅ^{-3}

Supplementary Table 15. Atomic coordinates and equivalent isotropic atomic displacement parameters (Å^2) for **4a′**. U(eq) is defined as one third of the trace of the orthogonalized Uij tensor.

	x/a	y/b	z/c	U(eq)
O(1)	11872(3)	9104(2)	10090(1)	51(1)
O(2A)	10220(40)	7680(30)	10018(15)	35(3)
O(8A)	3010(20)	775(17)	7325(19)	35(4)
O(14A)	3968(11)	350(7)	1417(6)	67(3)
O(2B)	10030(30)	7750(20)	9988(11)	35(3)
O(8B)	5249(9)	1240(7)	9805(6)	82(3)
O(14B)	5820(30)	3447(12)	3060(20)	30(3)
O(2C)	12618(9)	9013(5)	8748(5)	63(2)
O(8C)	3270(20)	694(16)	7385(18)	43(4)
O(14C)	6030(30)	3417(14)	3030(30)	30(3)
O(3)	11052(2)	5601(2)	8909(2)	49(1)
O(4)	7135(2)	6346(2)	8169(2)	45(1)
O(5)	6727(2)	3712(1)	8485(1)	42(1)
O(6)	10777(3)	4206(2)	9171(2)	64(1)
O(7)	3244(3)	436(2)	8616(2)	80(1)
O(9)	6476(2)	906(2)	6787(1)	45(1)
O(10)	3821(3)	3001(2)	7530(2)	56(1)
O(11)	5039(2)	2037(2)	4951(1)	42(1)
O(12)	7049(2)	347(2)	5342(2)	51(1)
O(13)	4761(2)	2011(2)	1560(1)	49(1)
O(15)	8611(2)	3015(1)	4213(1)	37(1)
O(16)	4558(2)	3684(2)	4788(1)	48(1)
O(17)	8582(2)	5609(1)	6777(1)	35(1)
O(18)	10801(2)	4182(1)	5247(1)	43(1)
C(1)	10441(3)	6713(2)	7832(2)	34(1)
C(2)	11392(3)	7442(2)	7905(2)	39(1)
C(3)	11869(3)	8244(2)	8663(2)	44(1)

C(4)	11408(3)	8318(2)	9349(2)	40(1)
C(5)	10463(3)	7574(2)	9280(2)	37(1)
C(6)	9968(3)	6771(2)	8527(2)	34(1)
C(7)	9094(3)	5958(2)	8506(2)	35(1)
C(8)	9701(3)	5400(2)	8714(2)	37(1)
C(9)	8925(3)	4626(2)	8720(2)	40(1)
C(10)	7544(3)	4448(2)	8502(2)	38(1)
C(11)	6904(3)	4991(2)	8292(2)	41(1)
C(12)	7690(3)	5761(2)	8318(2)	37(1)
C(13)	9525(3)	4050(2)	8955(2)	48(1)
C(14)	8605(4)	3293(2)	8924(2)	50(1)
C(15)	7283(3)	3152(2)	8696(2)	43(1)
C(16)	6232(4)	2407(2)	8649(2)	48(1)
C(17)	6327(4)	2244(3)	9324(3)	60(1)
C(18)	5322(5)	1584(3)	9321(3)	69(1)
C(19)	4256(5)	1095(3)	8656(3)	67(1)
C(20)	4178(4)	1232(2)	7959(3)	52(1)
C(21)	5163(3)	1886(2)	7948(2)	43(1)
C(22)	5141(3)	1950(2)	7169(2)	42(1)
C(23)	5804(3)	1427(2)	6600(2)	38(1)
C(24)	5786(3)	1432(2)	5835(2)	38(1)
C(25)	5102(3)	1999(2)	5680(2)	39(1)
C(26)	4455(3)	2540(2)	6239(2)	46(1)
C(27)	4459(3)	2505(2)	6975(2)	46(1)
C(28)	6427(3)	865(2)	5218(2)	39(1)
C(29)	6299(3)	942(2)	4470(2)	41(1)
C(30)	5640(3)	1509(2)	4363(2)	38(1)
C(31)	5436(3)	1634(2)	3623(2)	37(1)
C(32)	4825(3)	878(2)	2846(2)	42(1)
C(33)	4588(3)	981(2)	2140(2)	43(1)
C(34)	4959(3)	1845(2)	2212(2)	39(1)

C(35)	5577(3)	2599(2)	2989(2)	36(1)
C(36)	5826(3)	2508(2)	3696(2)	34(1)
C(37)	6570(3)	3330(2)	4509(2)	34(1)
C(38)	7952(3)	3555(2)	4734(2)	31(1)
C(39)	8680(3)	4335(2)	5496(2)	31(1)
C(40)	7950(3)	4859(2)	6023(2)	32(1)
C(41)	6570(3)	4659(2)	5818(2)	38(1)
C(42)	5888(3)	3904(2)	5050(2)	38(1)
C(43)	10124(3)	4625(2)	5728(2)	34(1)
C(44)	10703(3)	5426(2)	6518(2)	36(1)
C(45)	9942(3)	5878(2)	7011(2)	33(1)
O(1W)	3410(2)	4683(2)	5909(2)	47(1)
O(2W)	2829(3)	4228(2)	7183(2)	72(1)
O(5W)	1630(5)	-1026(2)	6997(3)	115(2)
C(3S)	1842(5)	1113(4)	10234(4)	95(2)
O(3S)	2896(2)	746(2)	10208(2)	51(1)
C(4S)	5856(8)	3561(5)	1139(4)	127(3)
O(4S)	5551(3)	3984(2)	1864(2)	71(1)
C(6S)	7469(14)	7279(8)	10841(8)	205(6)
O(6S)	8100(4)	6811(3)	10319(3)	90(1)

1.6 ^{1}H and ^{13}C Spectra of products

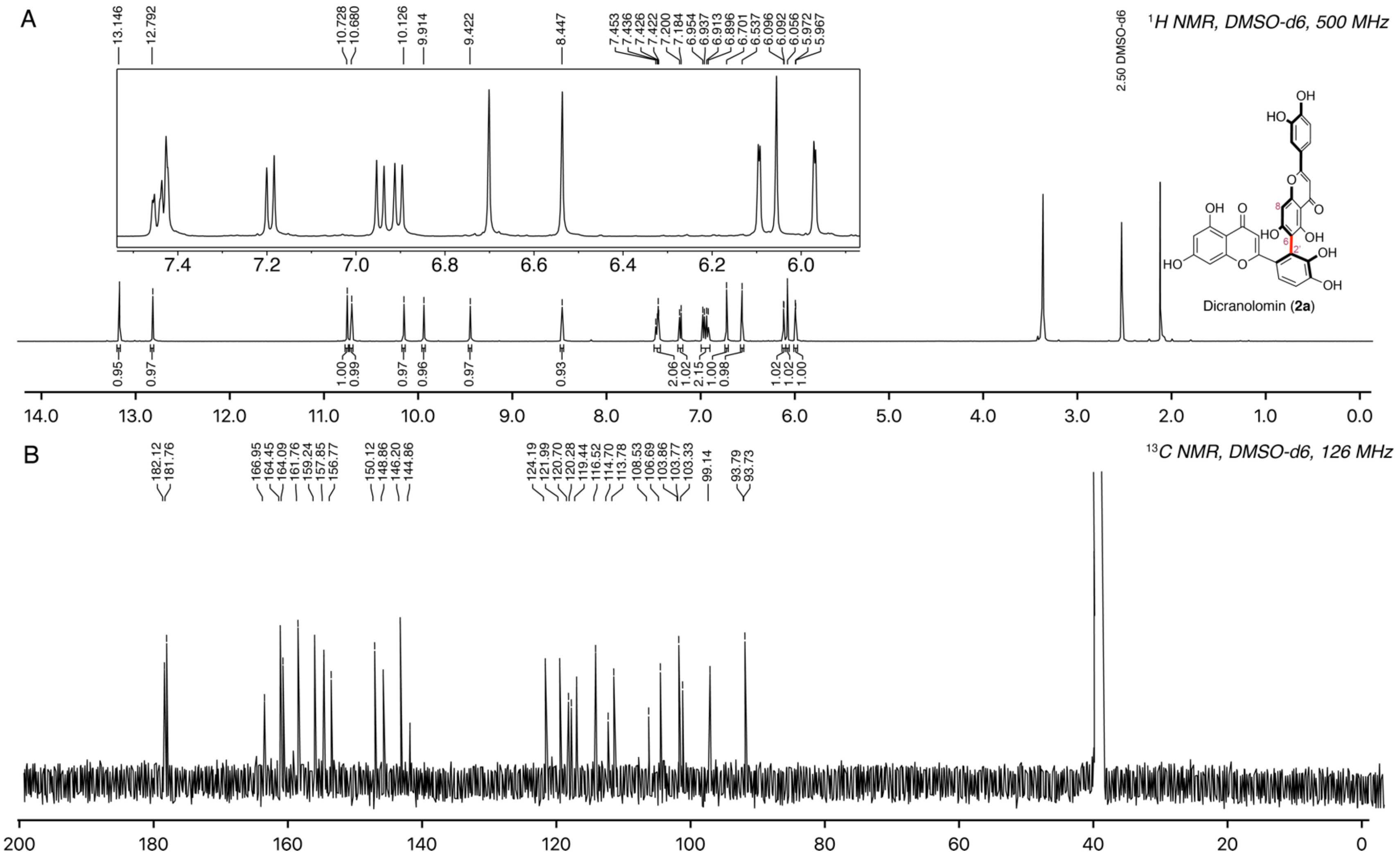

Supplementary Fig. 86. ^{1}H and ^{13}C NMR spectra of dicranolomin, 2a.

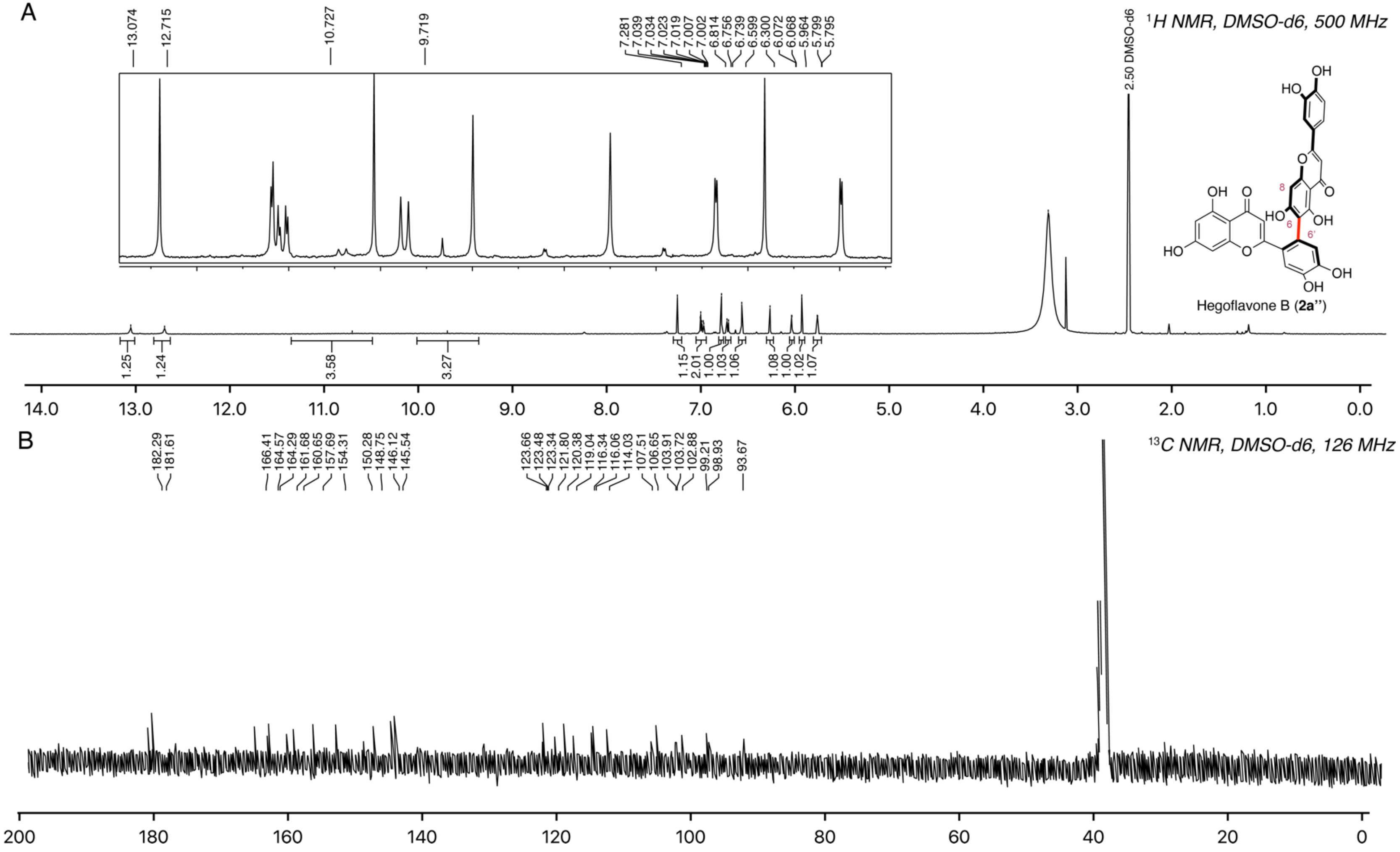

Supplementary Fig. 87. ¹H and ¹³C NMR spectra of hegoflavone B 2a''

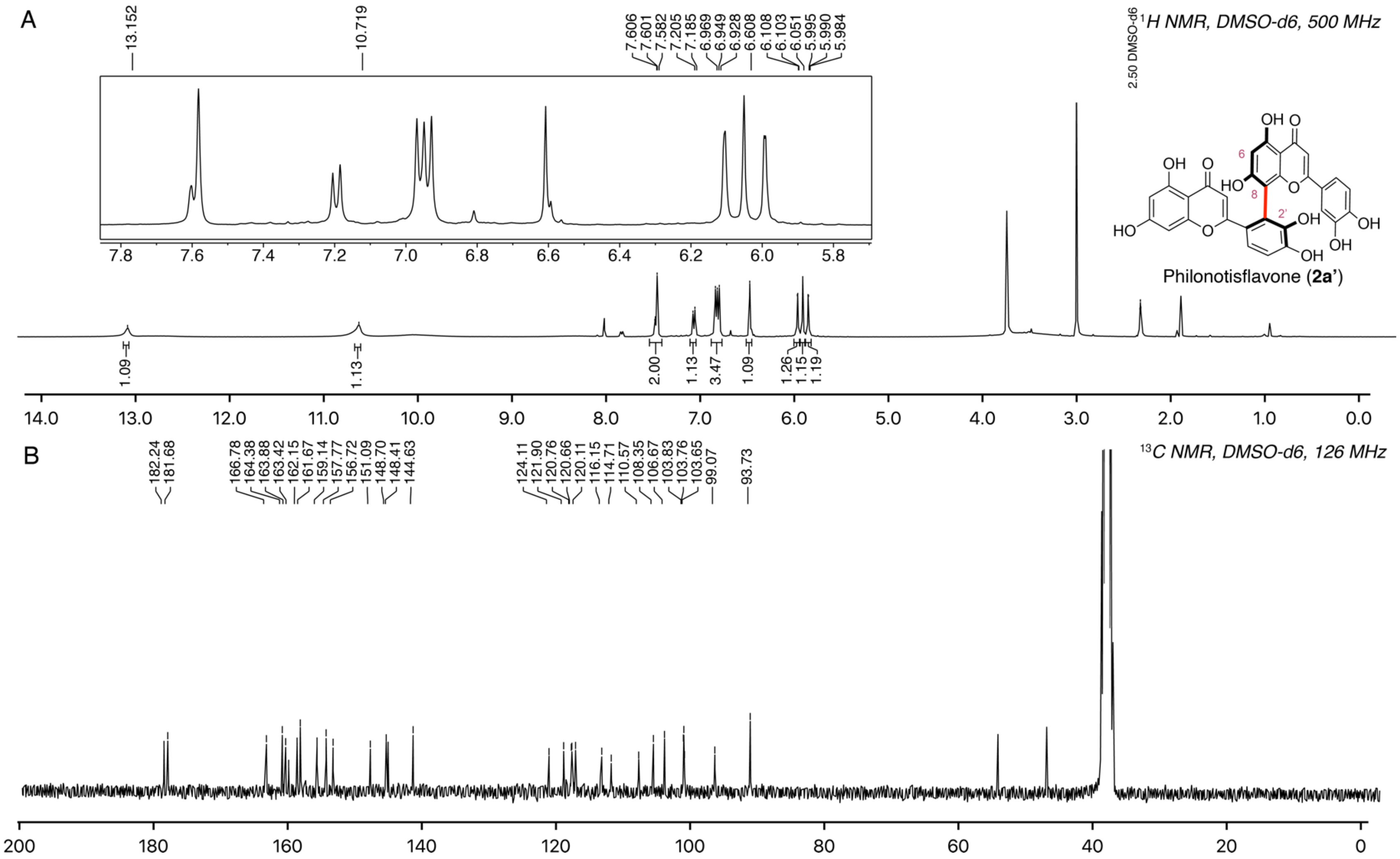

Supplementary Fig.88. ^{1}H and ^{13}C NMR spectra of philonotisflavone 2a'.

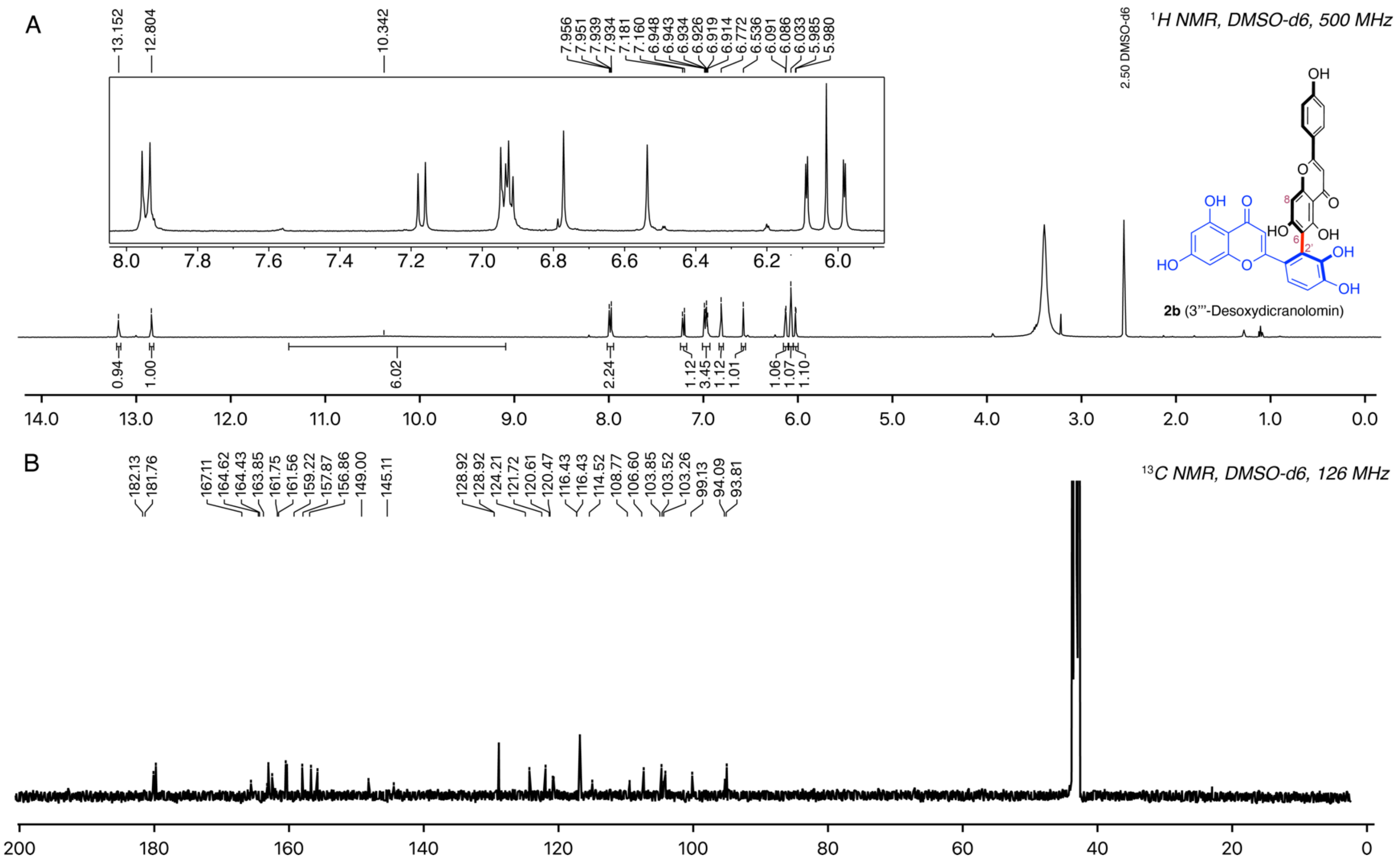

Supplementary Fig.89. ^{1}H and ^{13}C NMR spectra of 3'''-desoxydicranolomin, 2b.

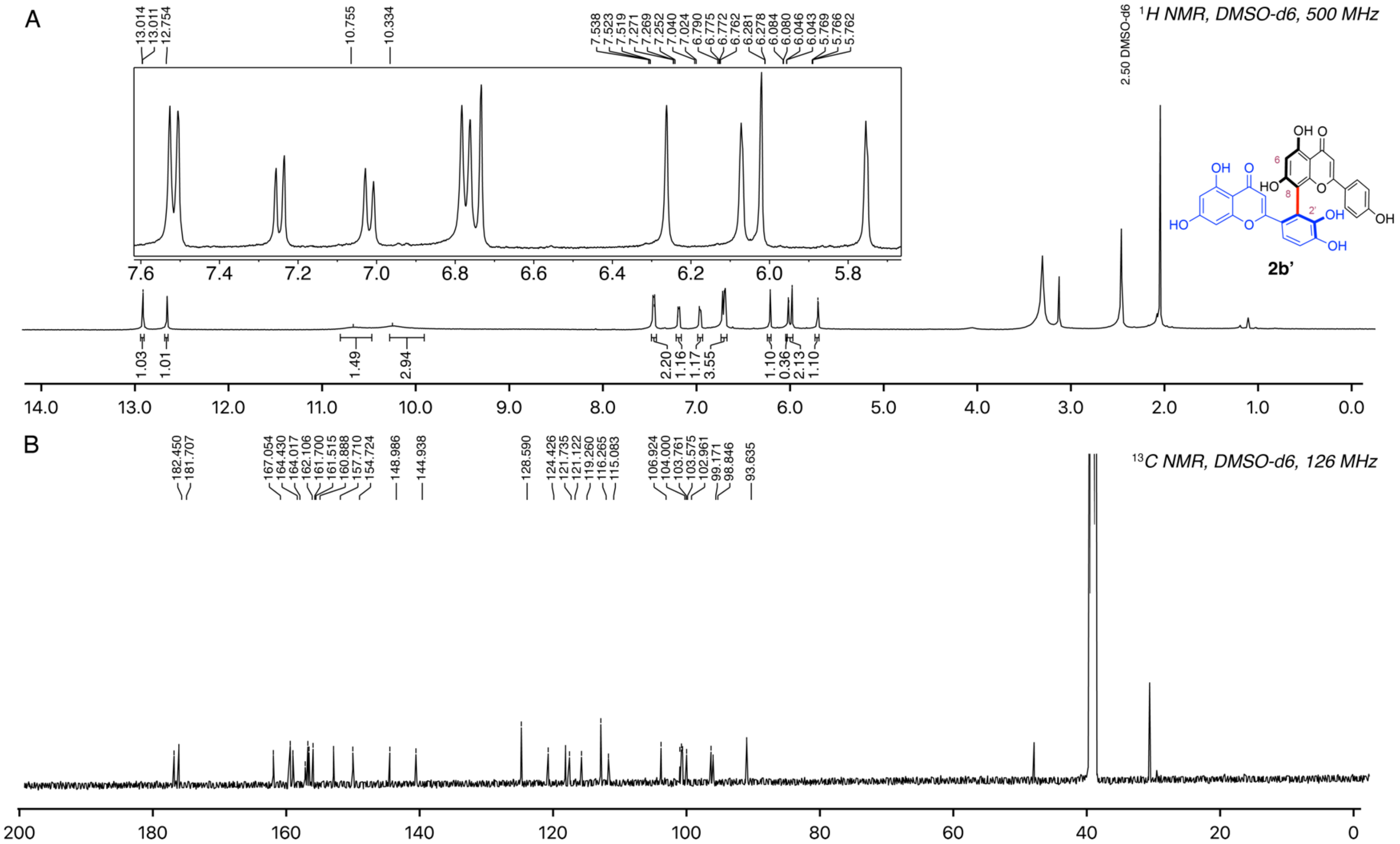

Supplementary Fig. 90. ^{1}H and ^{13}C NMR spectra of 2b'.

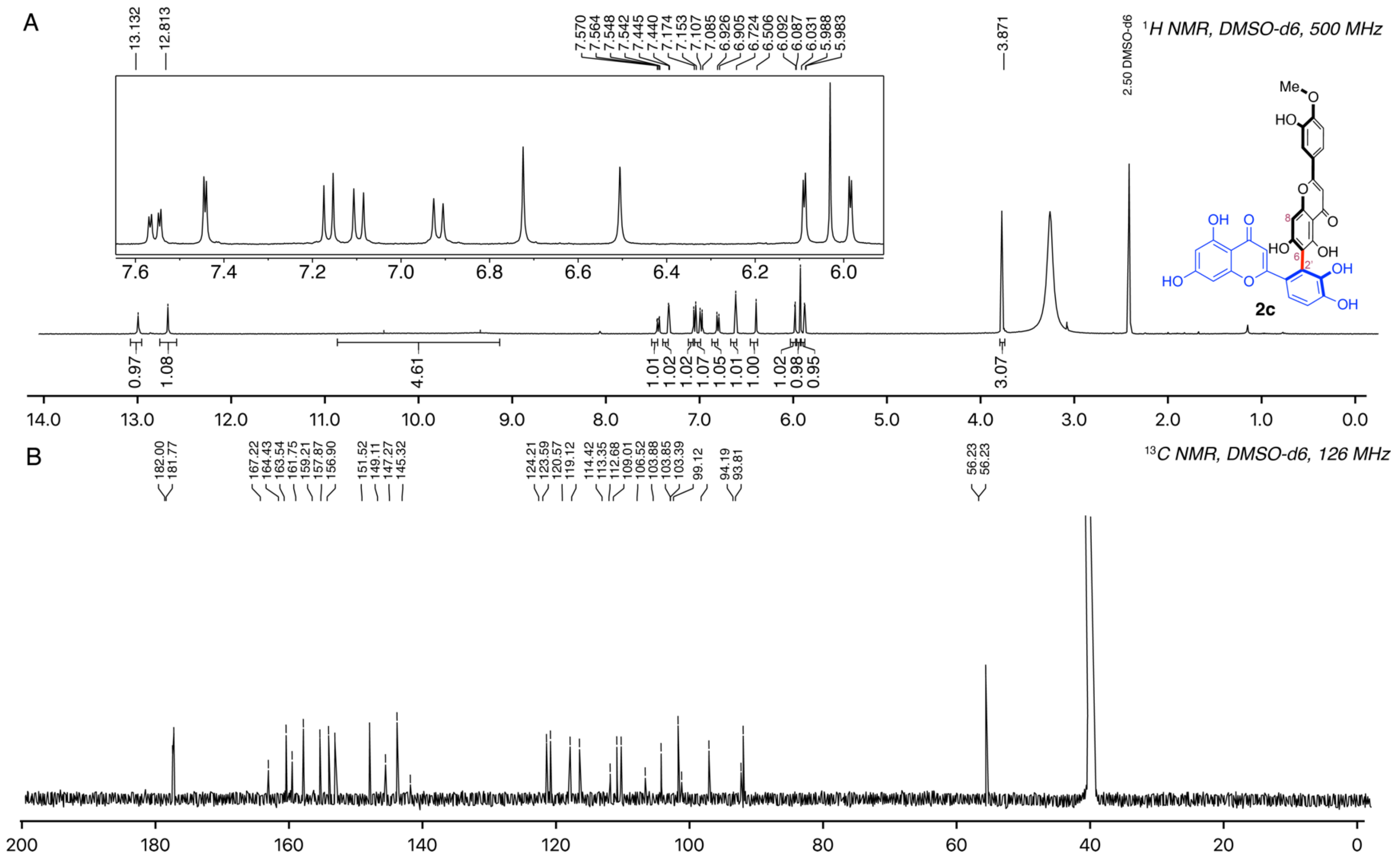

Supplementary Fig. 91. ^{1}H and ^{13}C NMR spectra of 2c.

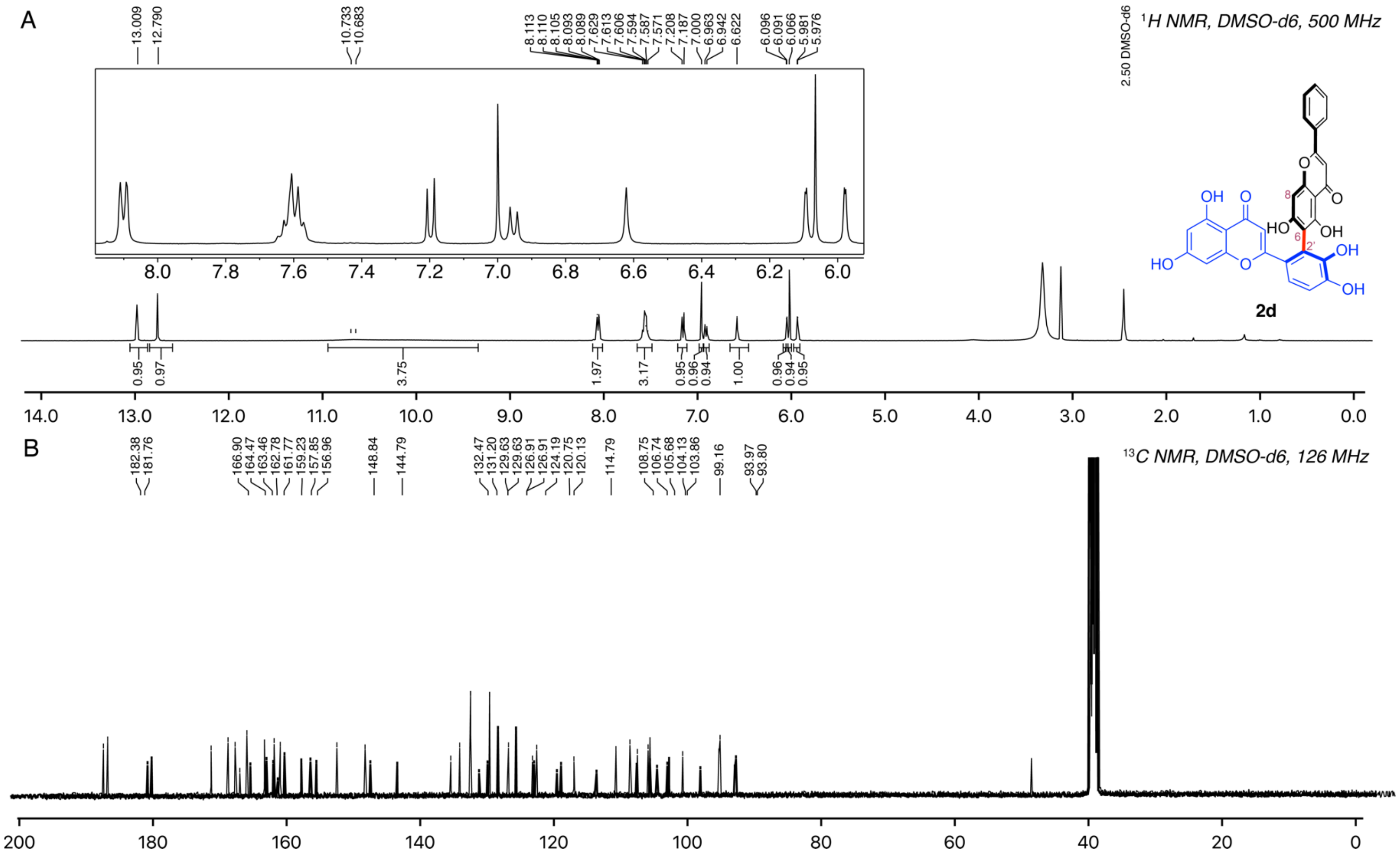

Supplementary Fig. 92. ^{1}H and ^{13}C NMR spectra of 2d.

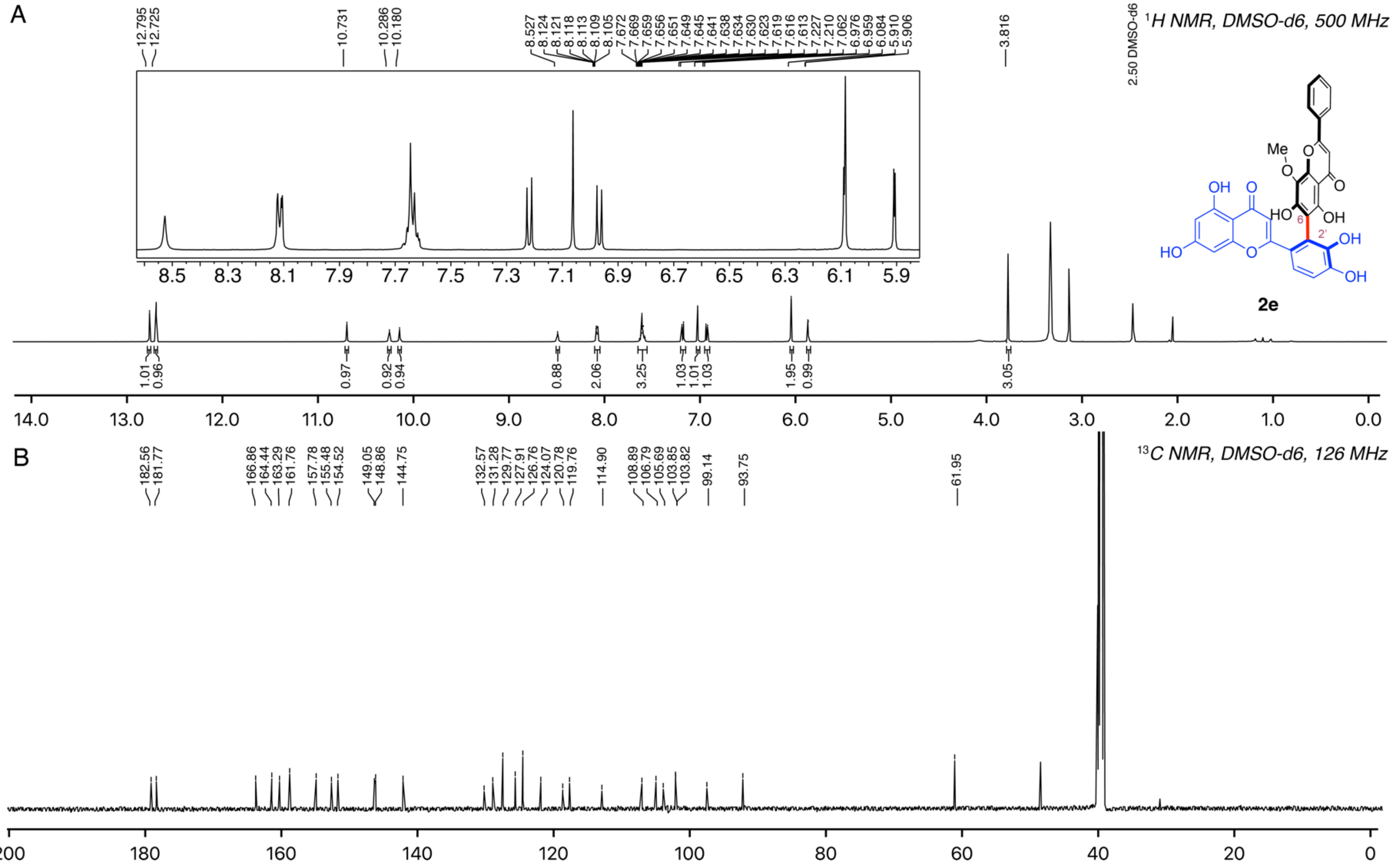

Supplementary Fig. 93. ^{1}H and ^{13}C NMR spectra of 2e.

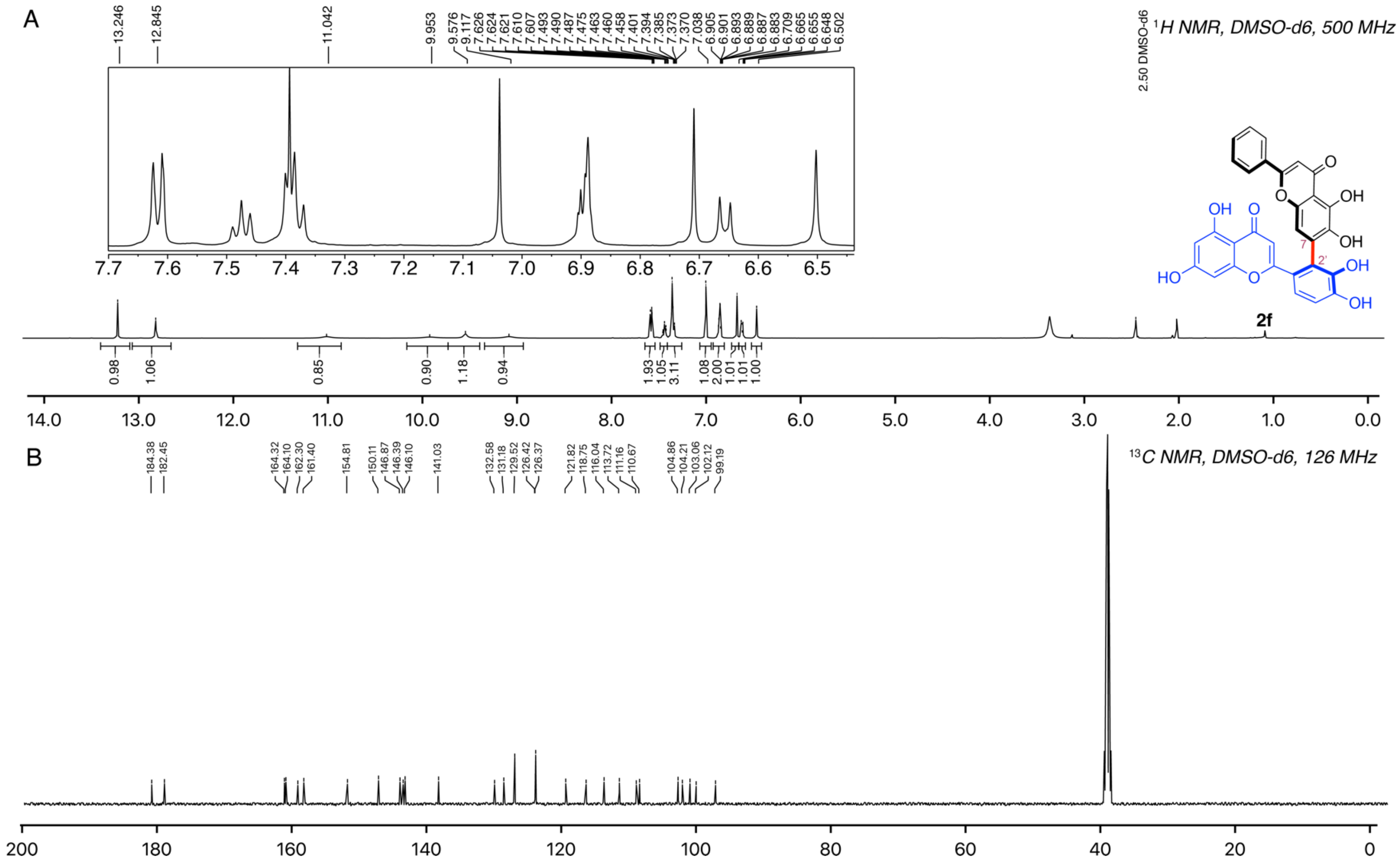

Supplementary Fig. 94. ^{1}H and ^{13}C NMR spectra of 2f.

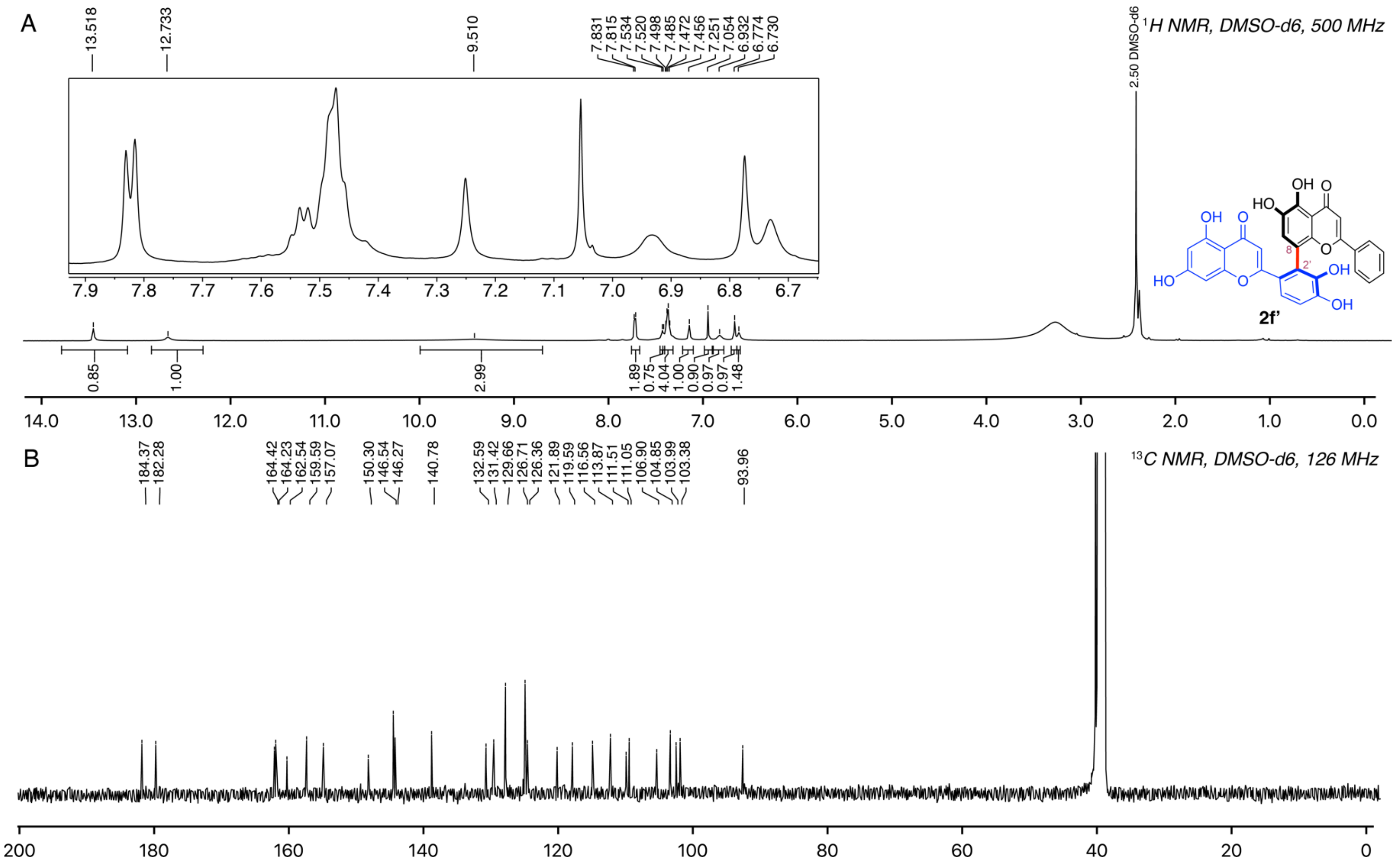

Supplementary Fig. 95. ^{1}H and ^{13}C NMR spectra of 2f'.

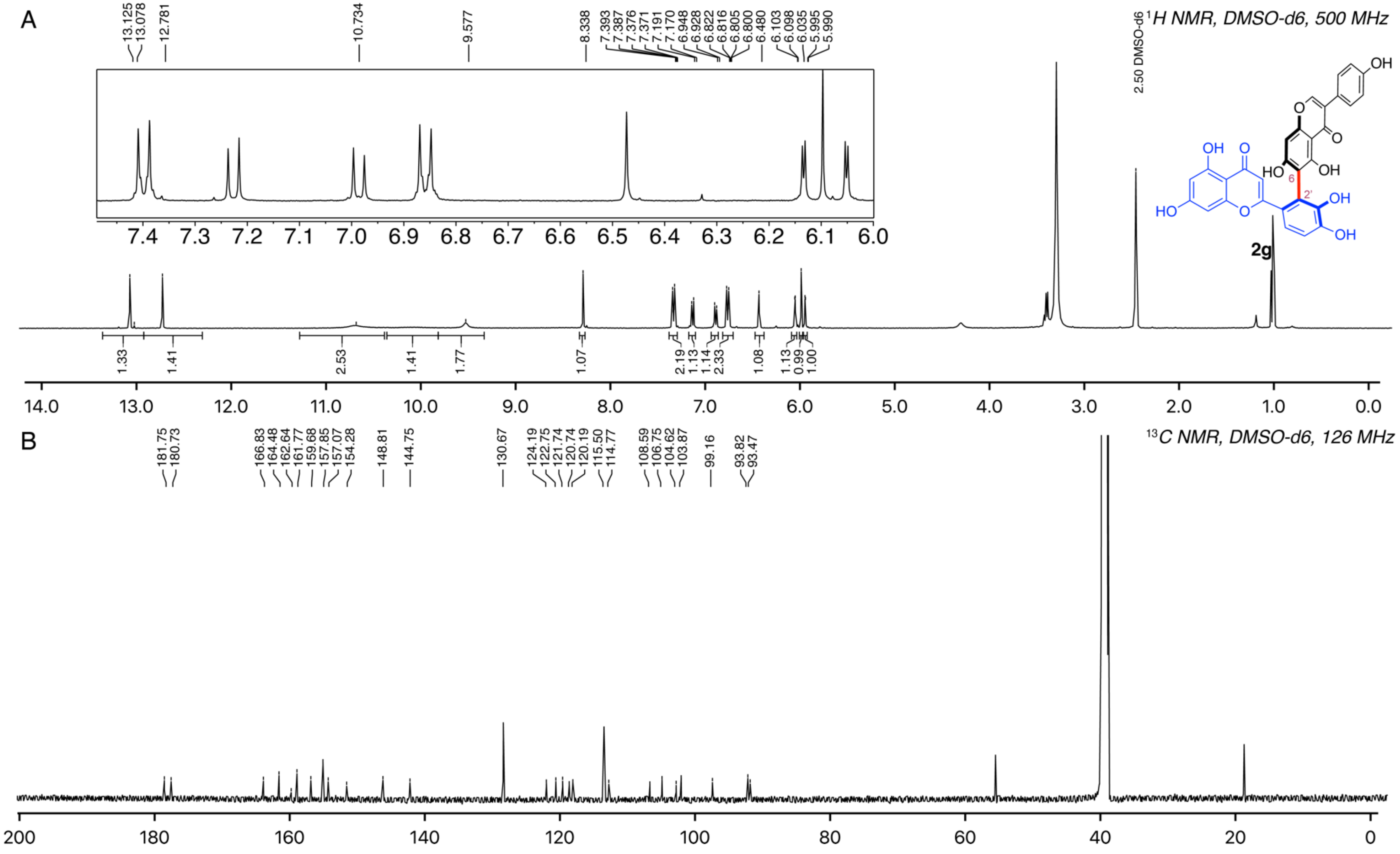

Supplementary Fig. 96. ^{1}H and ^{13}C NMR spectra of 2g.

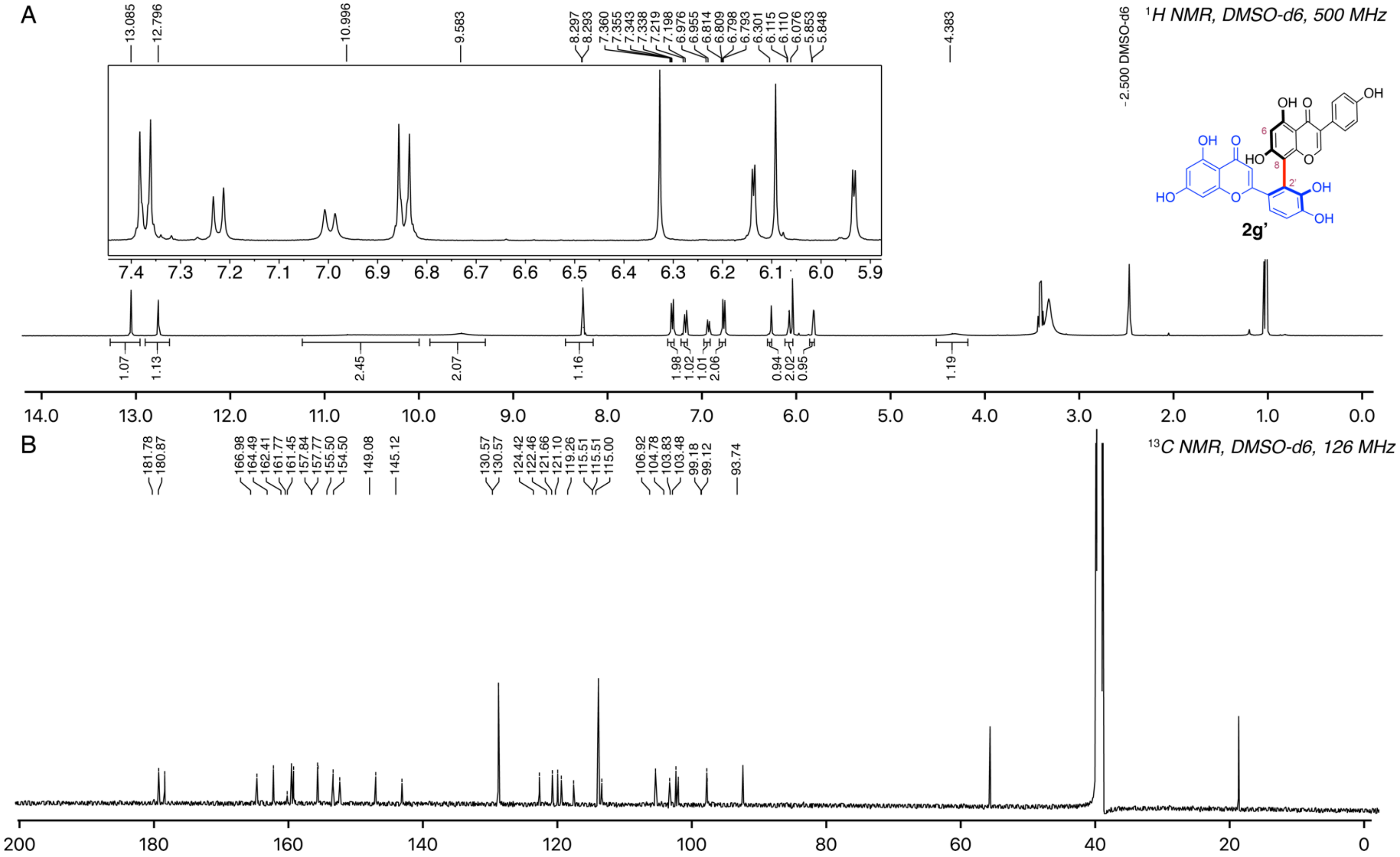

Supplementary Fig. 97. ^{1}H and ^{13}C NMR spectra of 2g'.

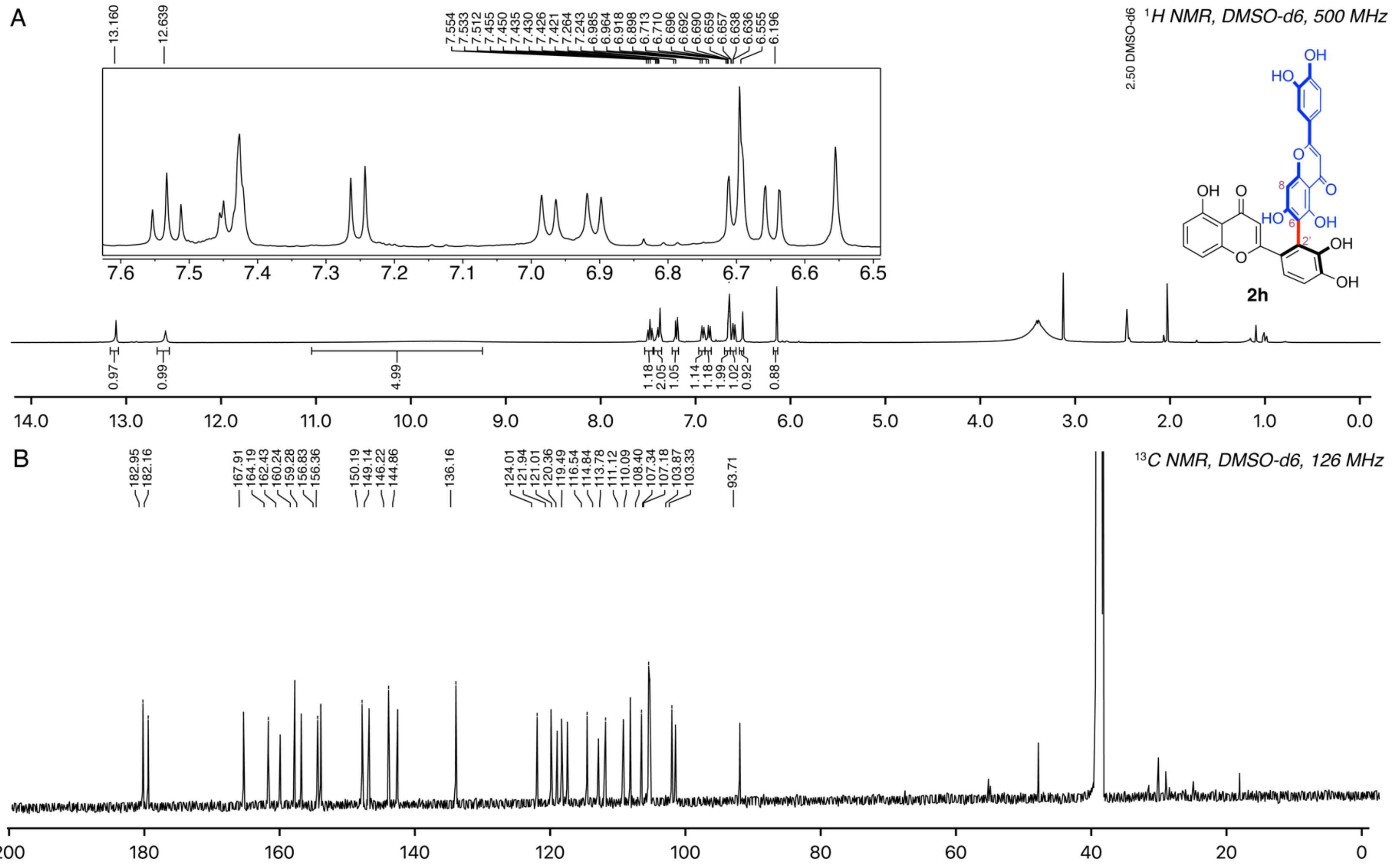

Supplementary Fig. 98. ^{1}H and ^{13}C NMR spectra of 2h.

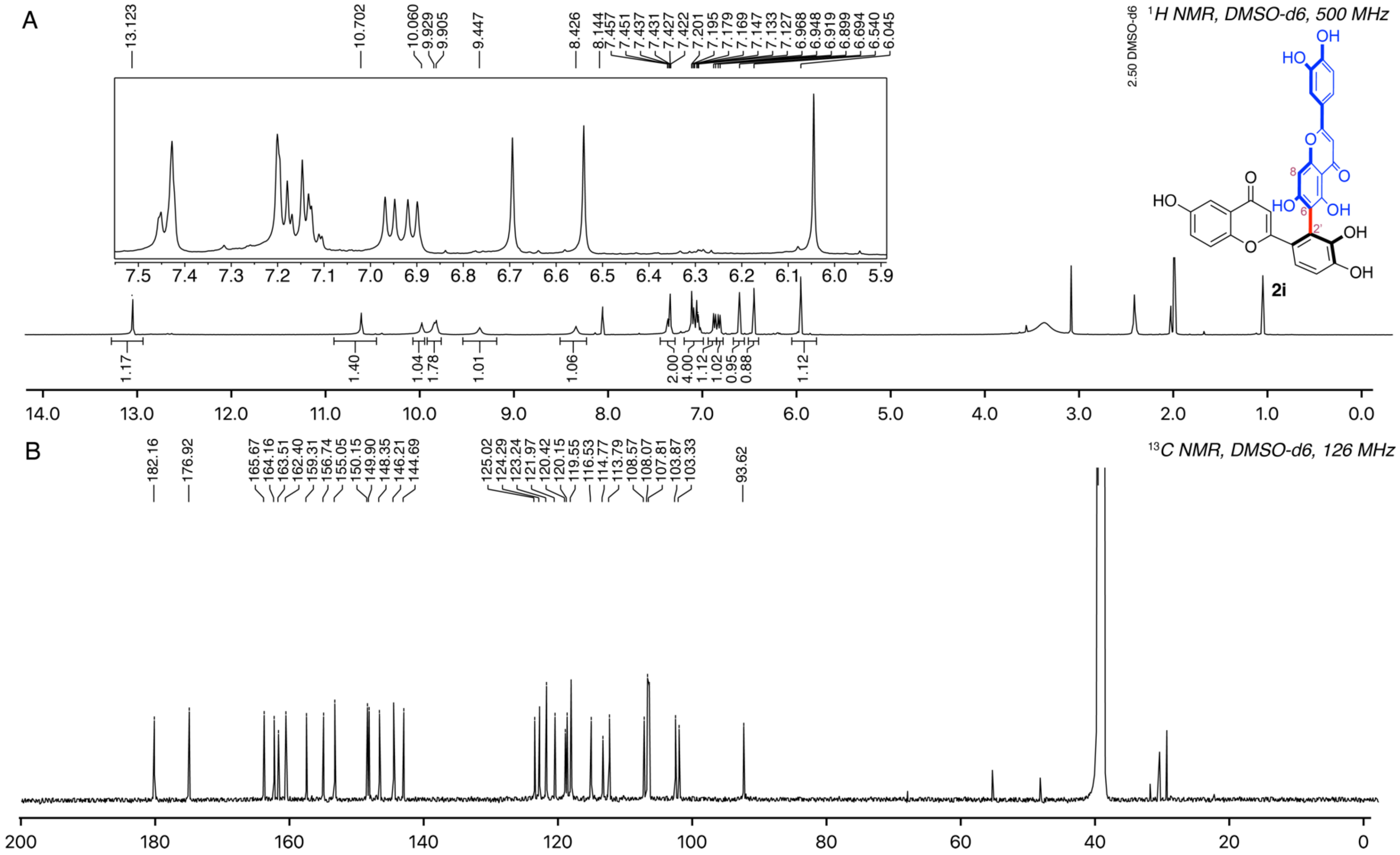

Supplementary Fig.99. ^{1}H and ^{13}C NMR spectra of 2i.

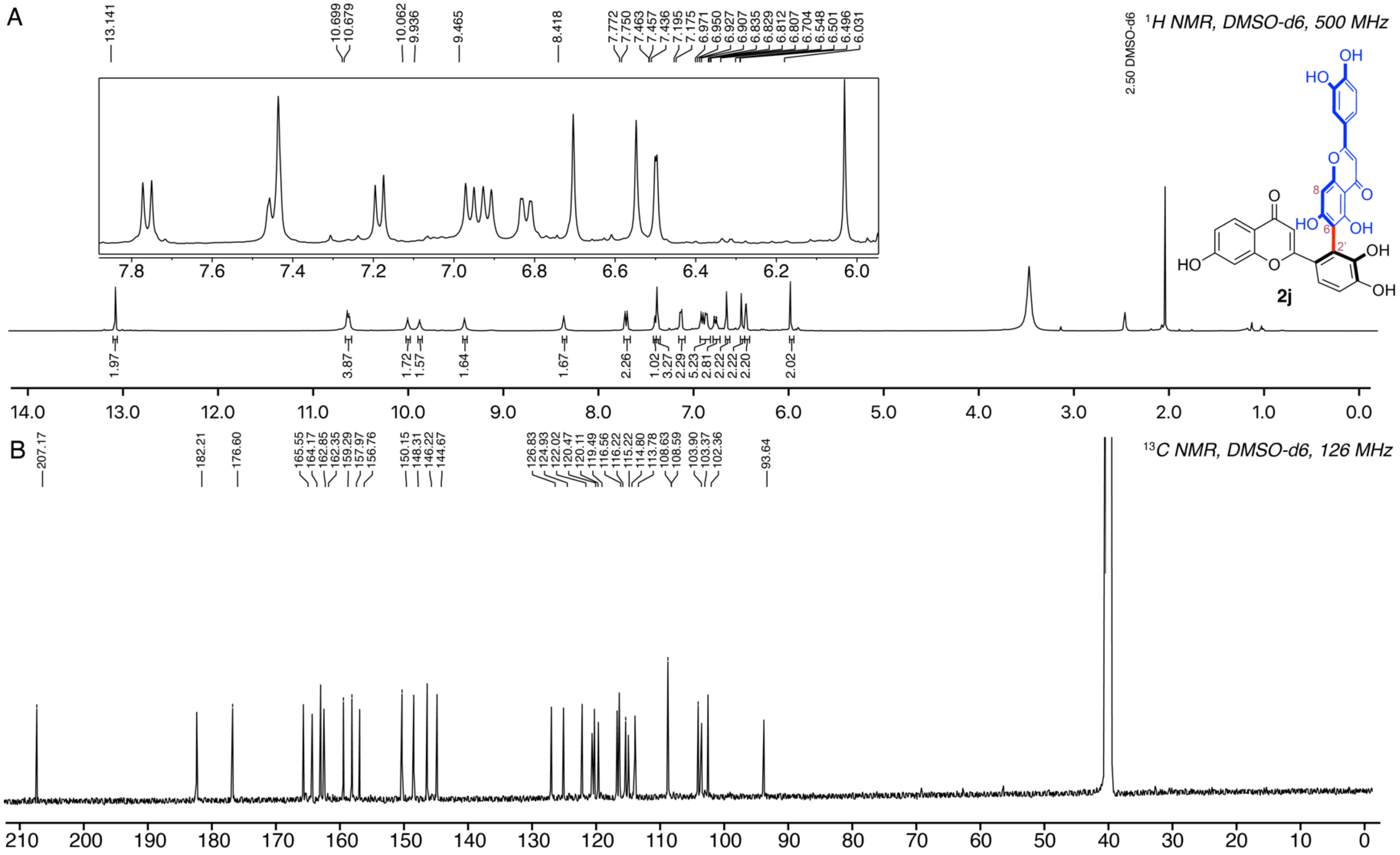

Supplementary Fig. 100. ^{1}H and ^{13}C NMR spectra of 2j.

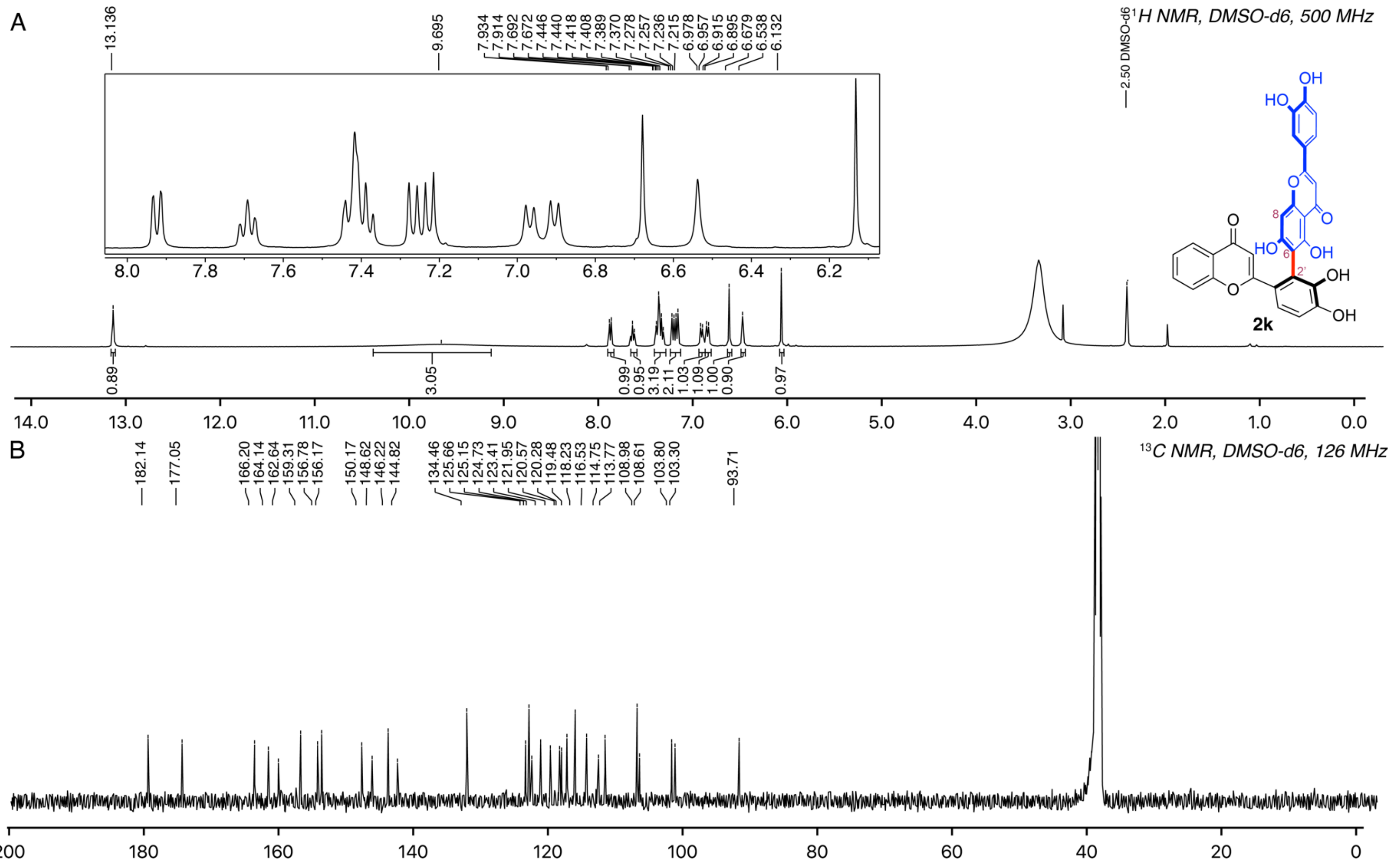

Supplementary Fig. 101. ^{1}H and ^{13}C NMR spectra of 2k.

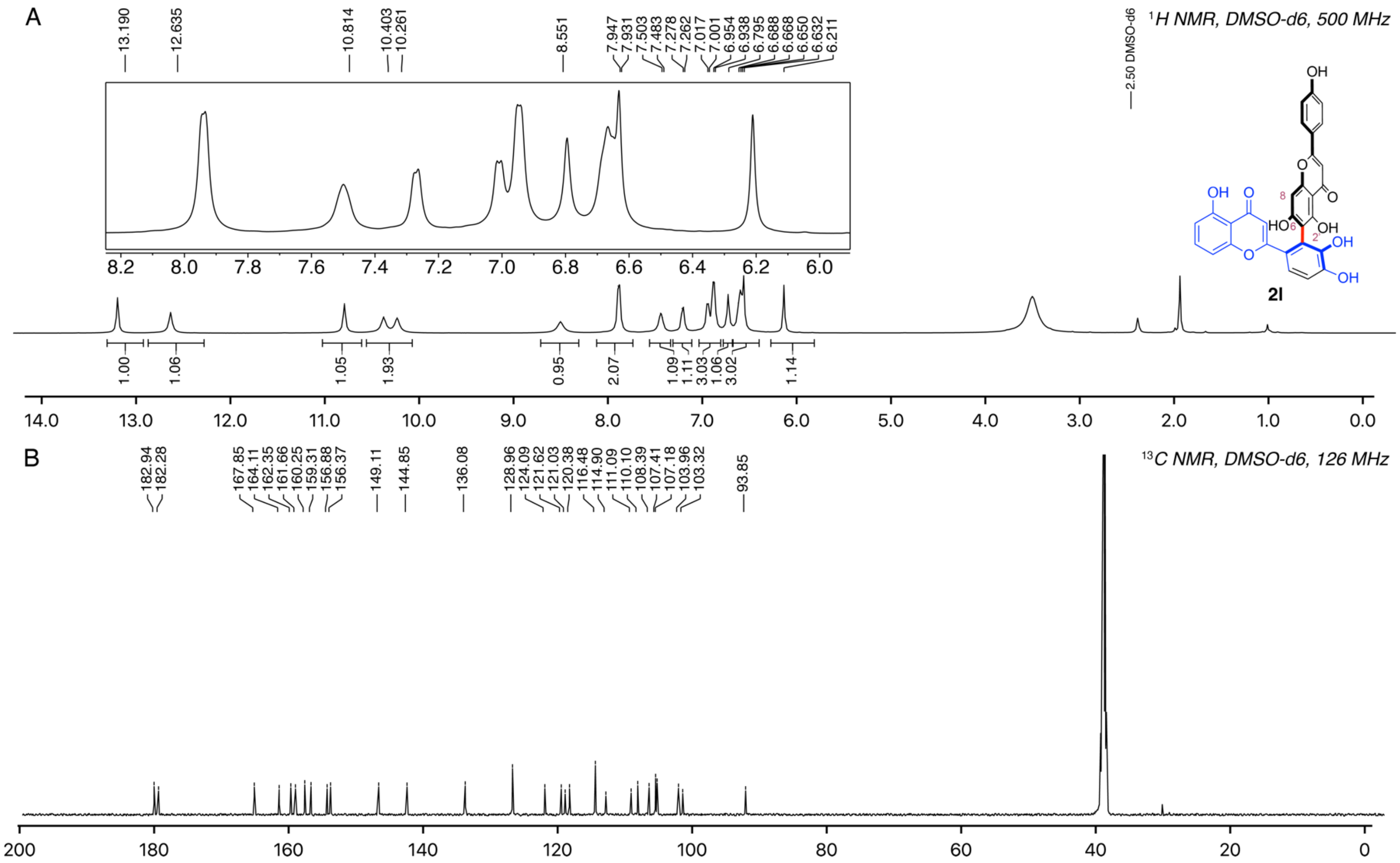

Supplementary Fig.102. ^{1}H and ^{13}C NMR spectra of 2l.

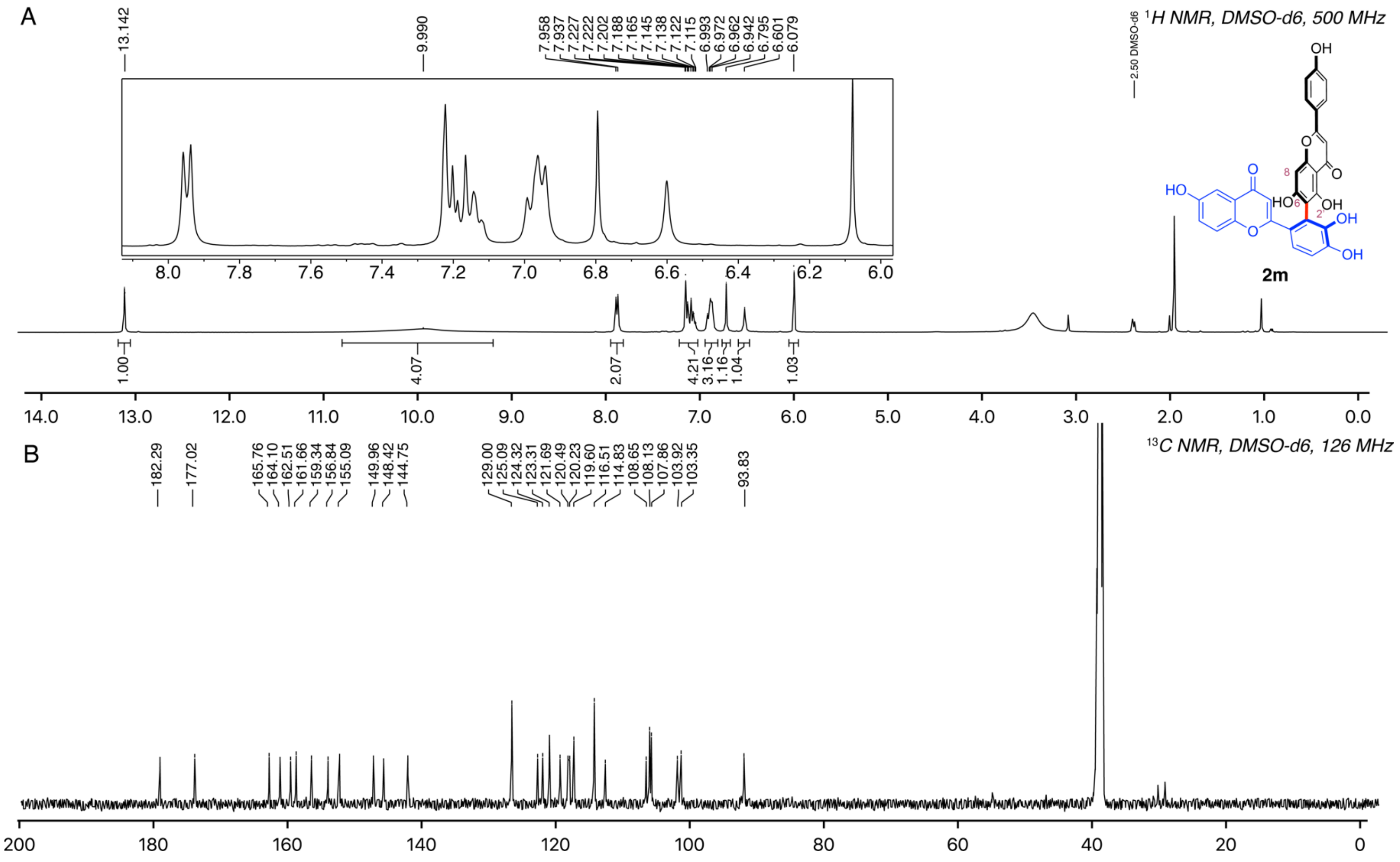

Supplementary Fig.103. ^{1}H and ^{13}C NMR spectra of 2m.

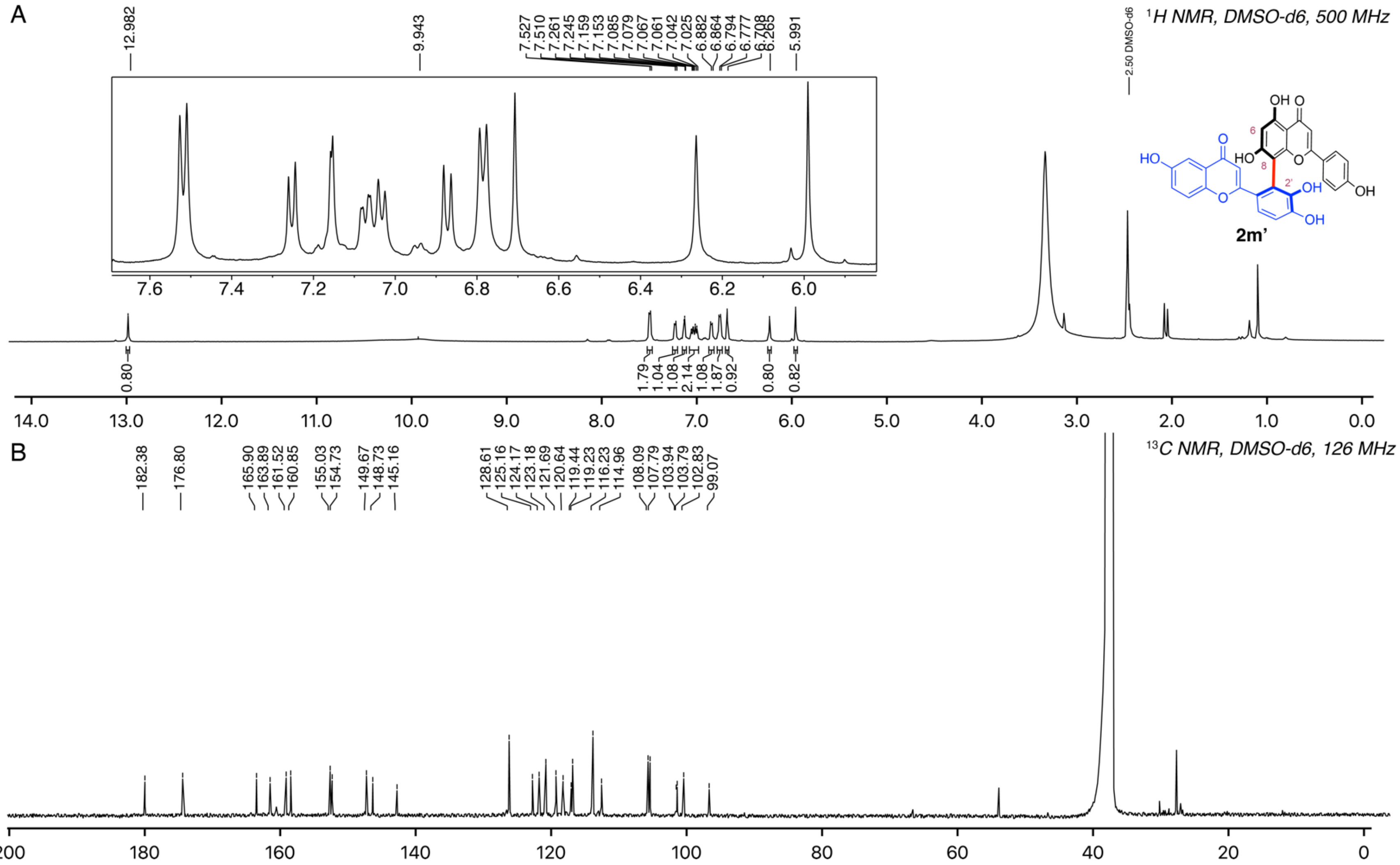

Supplementary Fig. 104. ^{1}H and ^{13}C NMR spectra of 2m'.

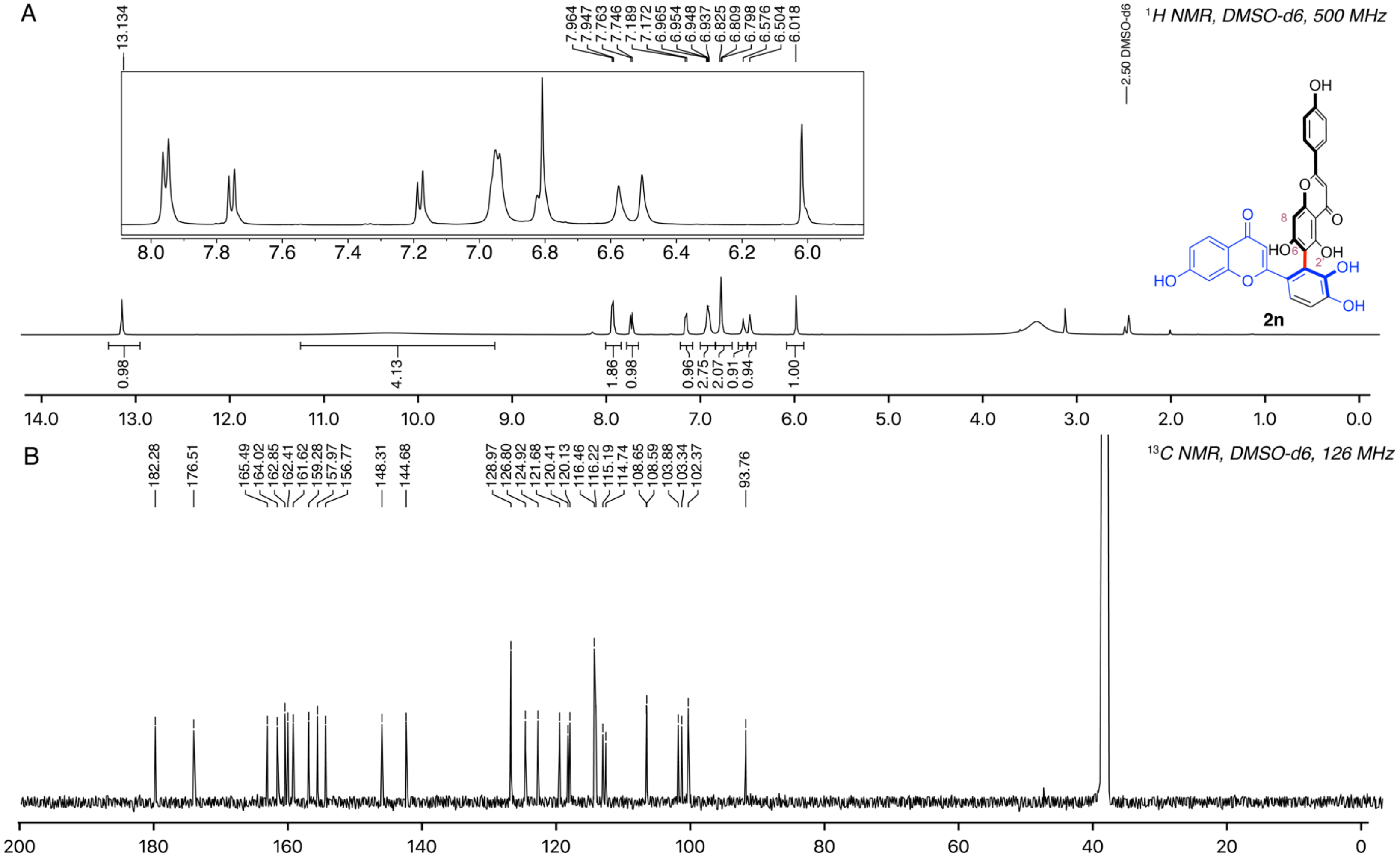

Supplementary Fig. 105. ^{1}H and ^{13}C NMR spectra of 2n.

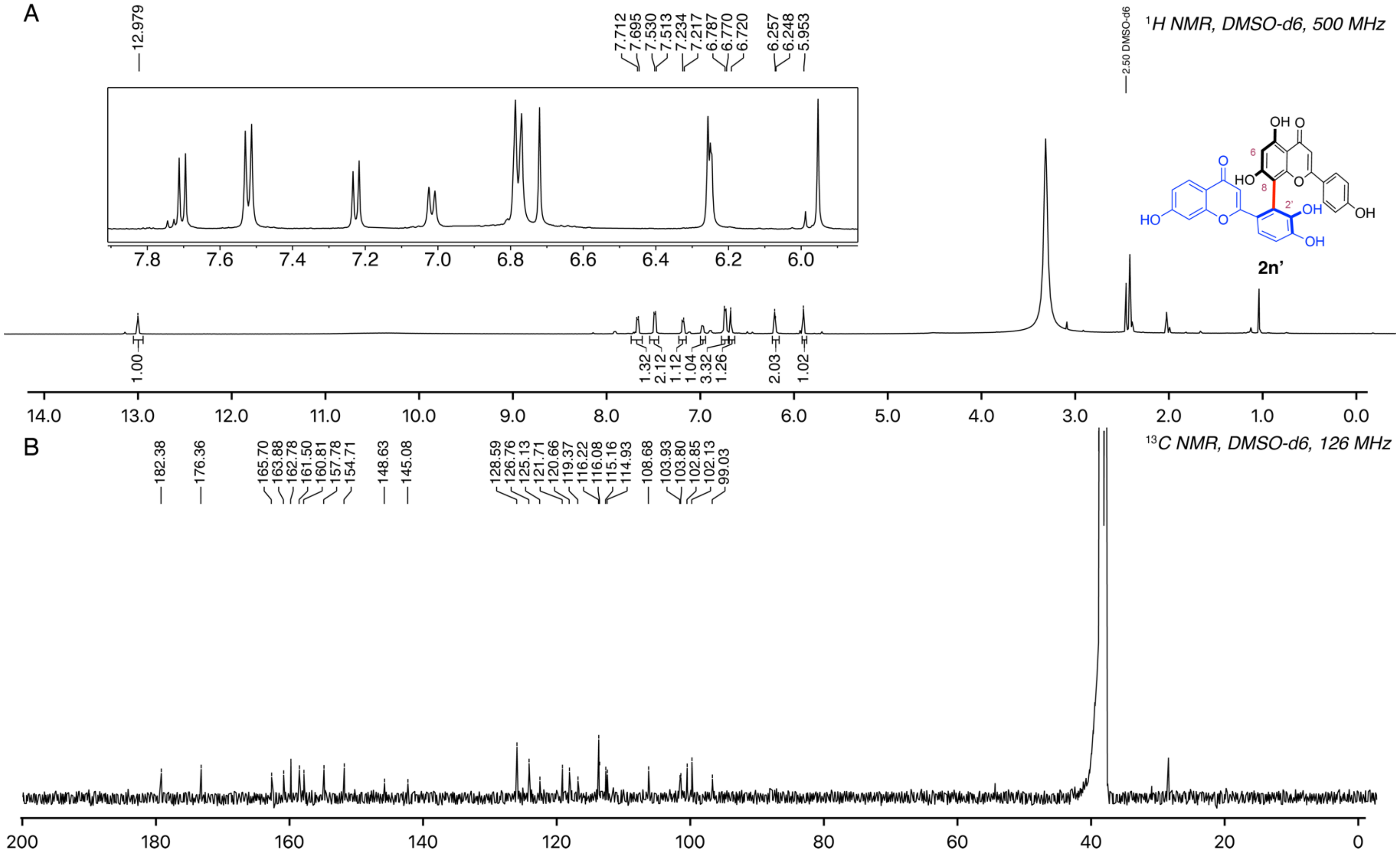

Supplementary Fig. 106. ¹H and ¹³C NMR spectra of 2n'.

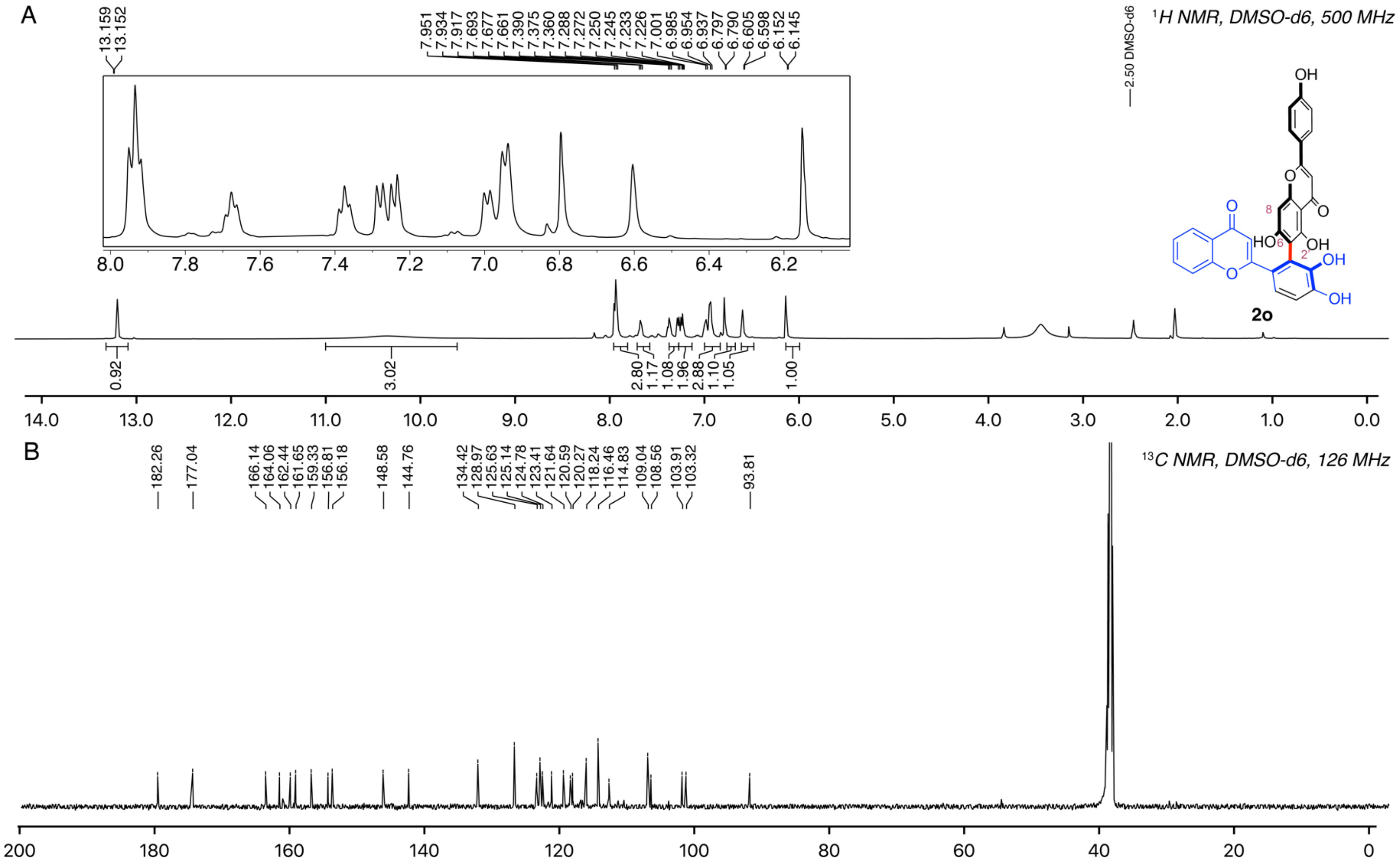

Supplementary Fig. 107. ^{1}H and ^{13}C NMR spectra of 2o.

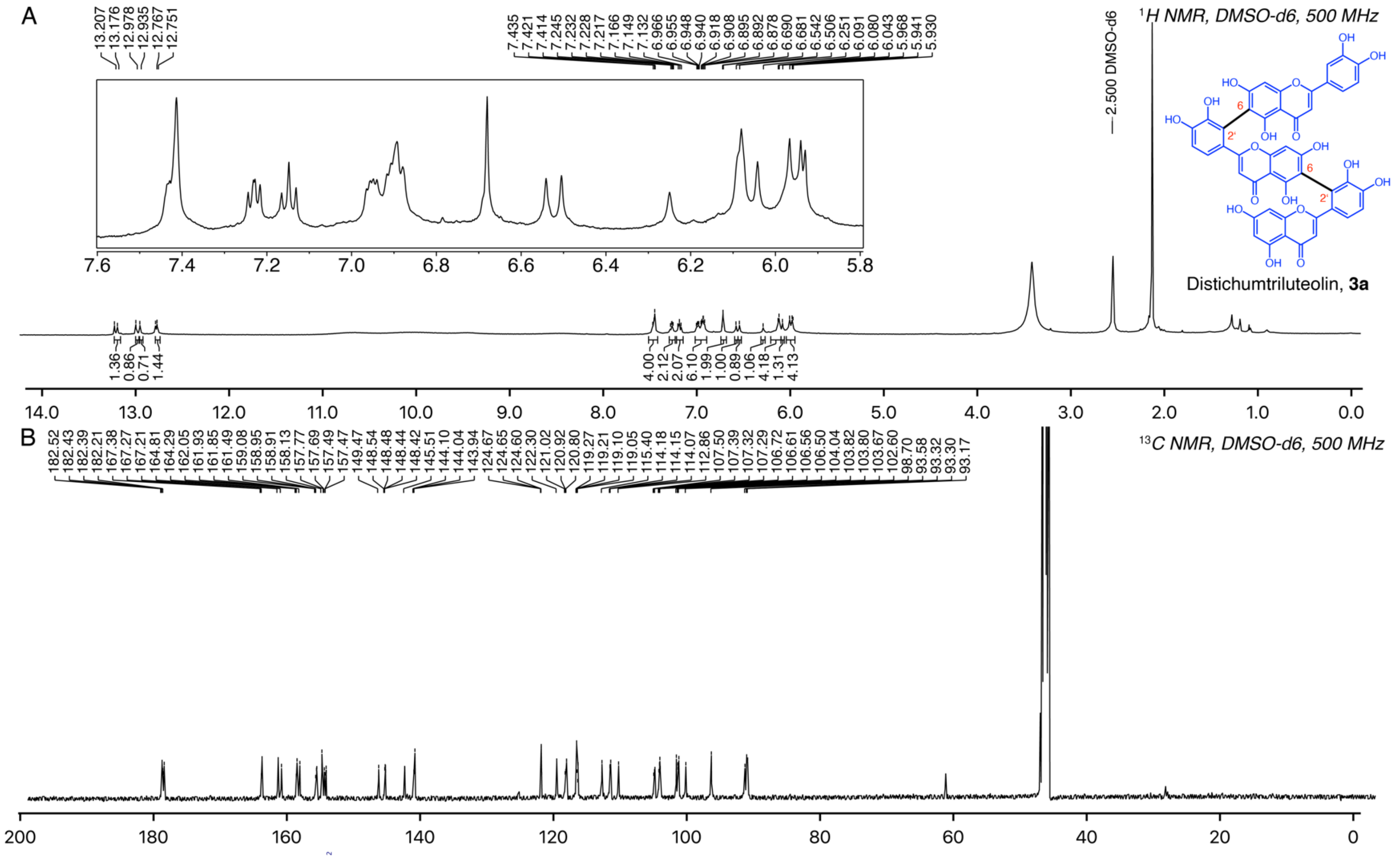

Supplementary Fig. 108. ^{1}H and ^{13}C NMR spectra of distichumtriluteolin, 3a.

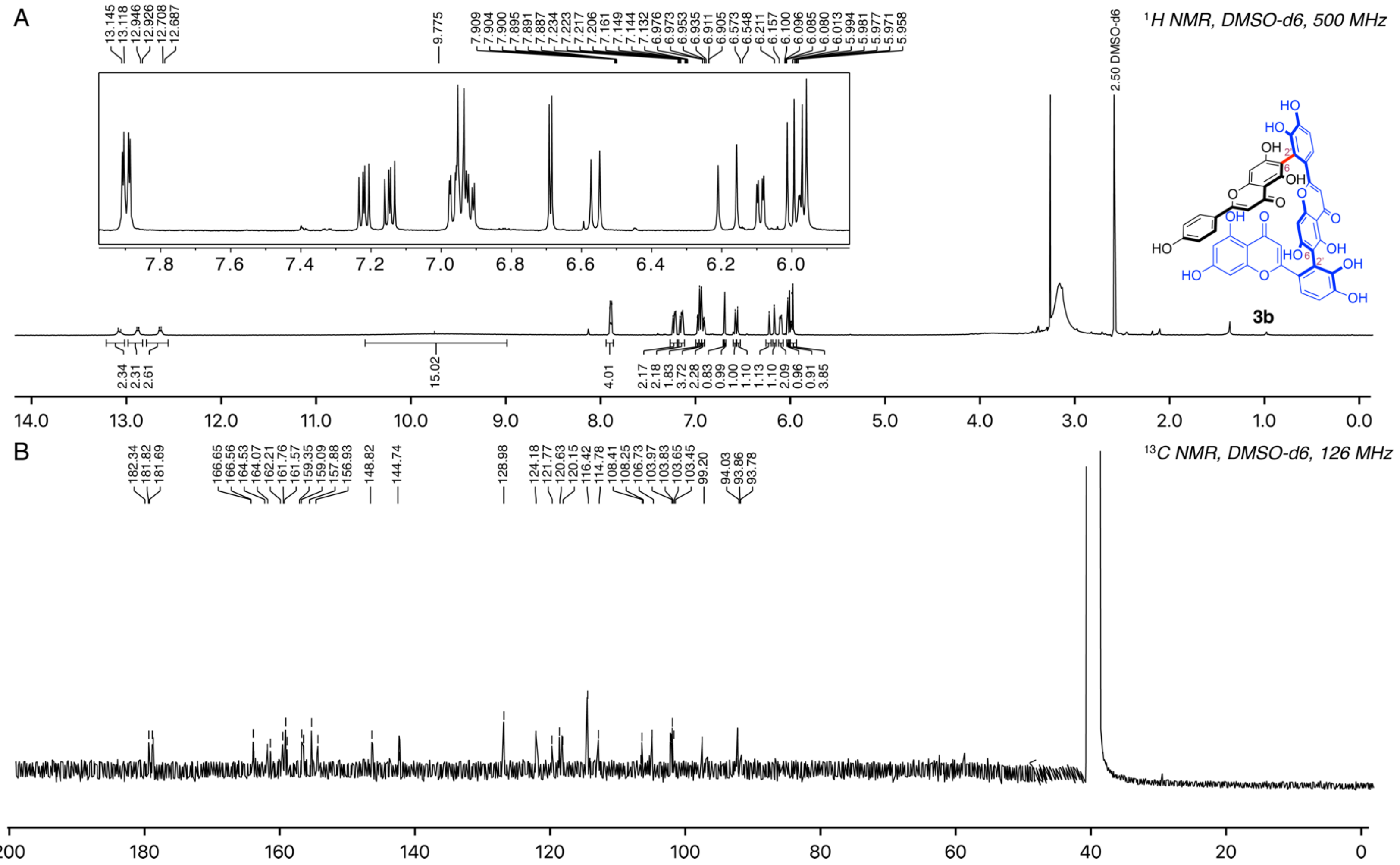

Supplementary Fig. 109. ^{1}H and ^{13}C NMR spectra of 3b.

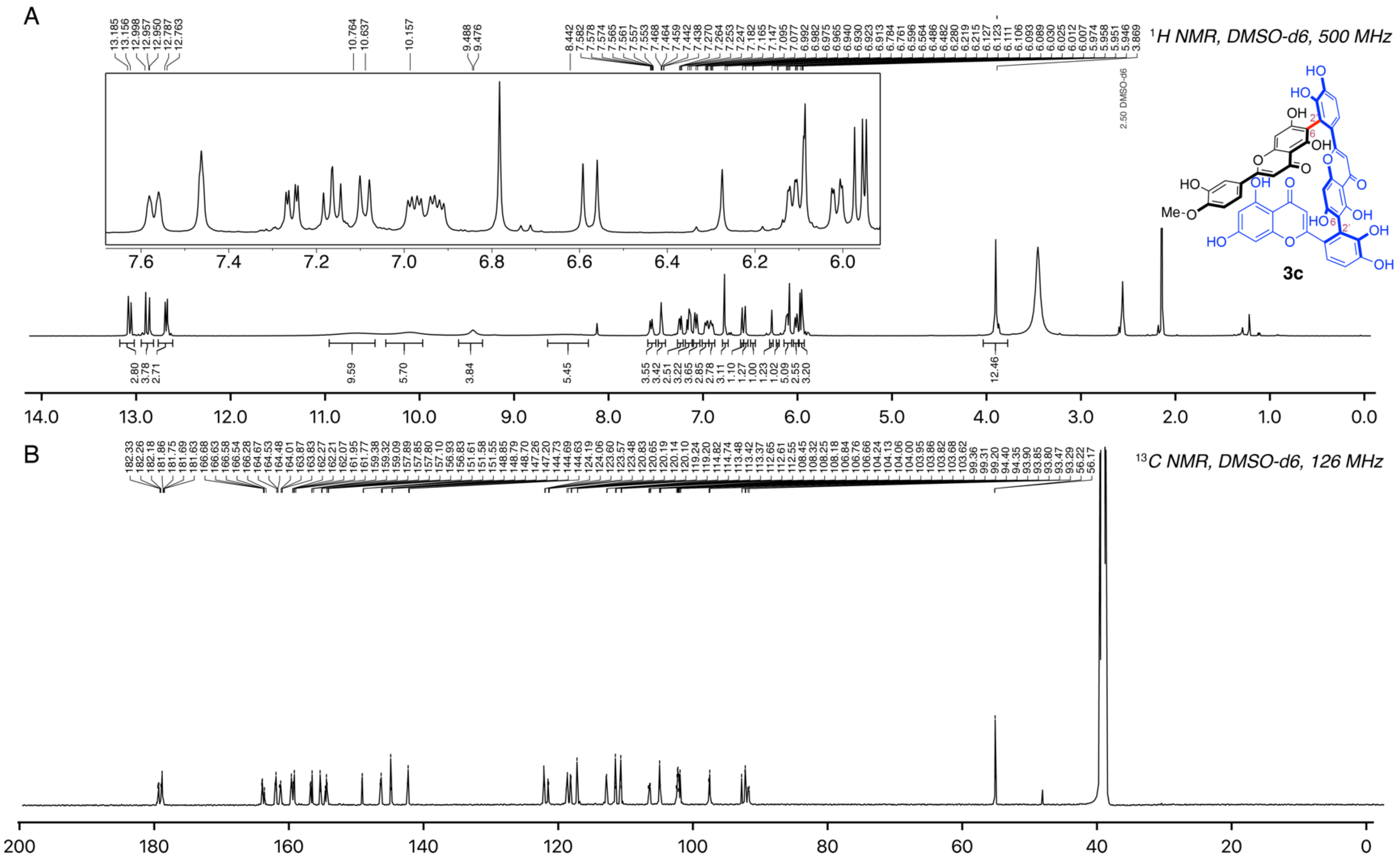

Supplementary Fig. 110. ^{1}H and ^{13}C NMR spectra of 3c.

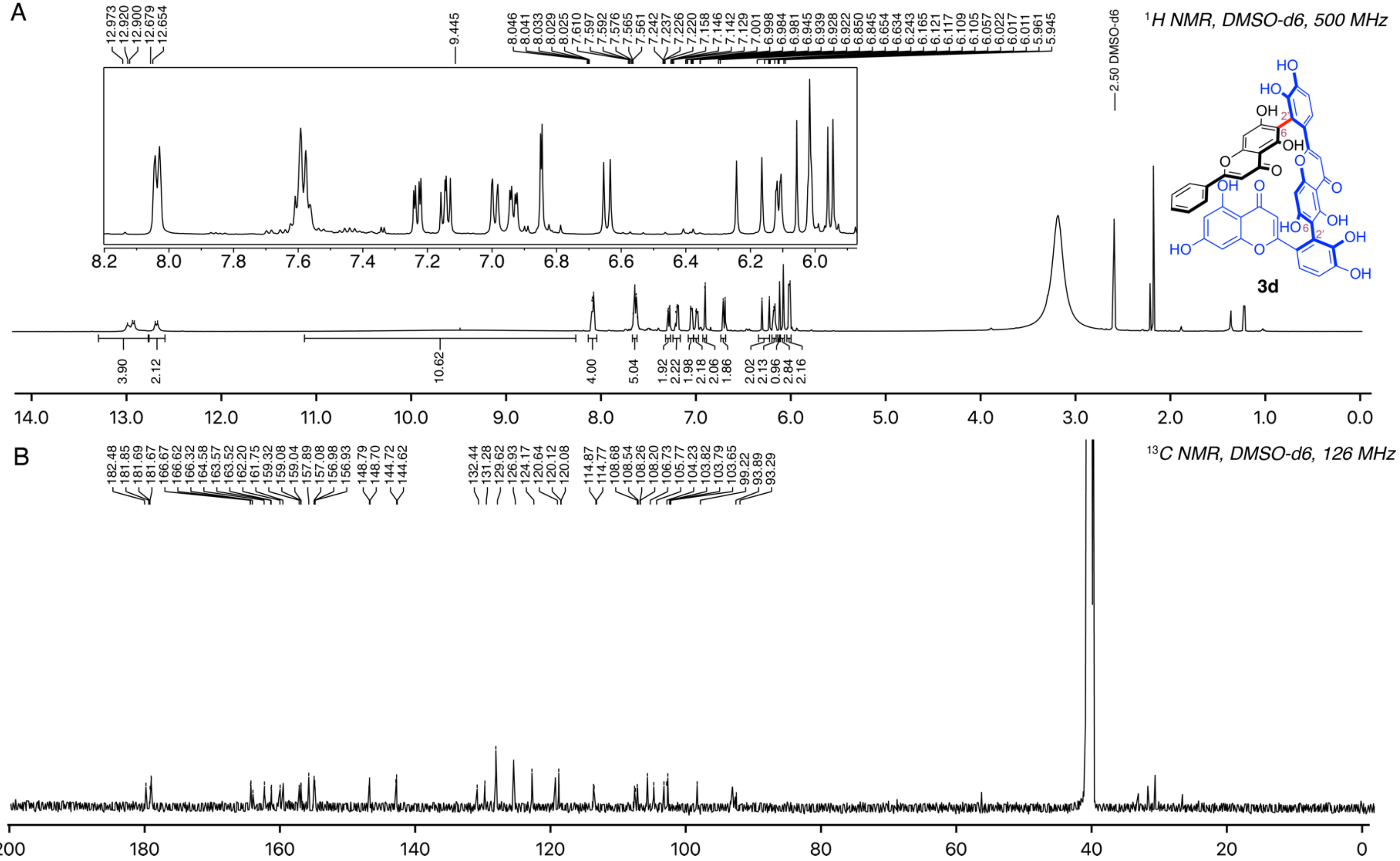

Supplementary Fig. 111. ¹H and ¹³C NMR spectra of 3d.

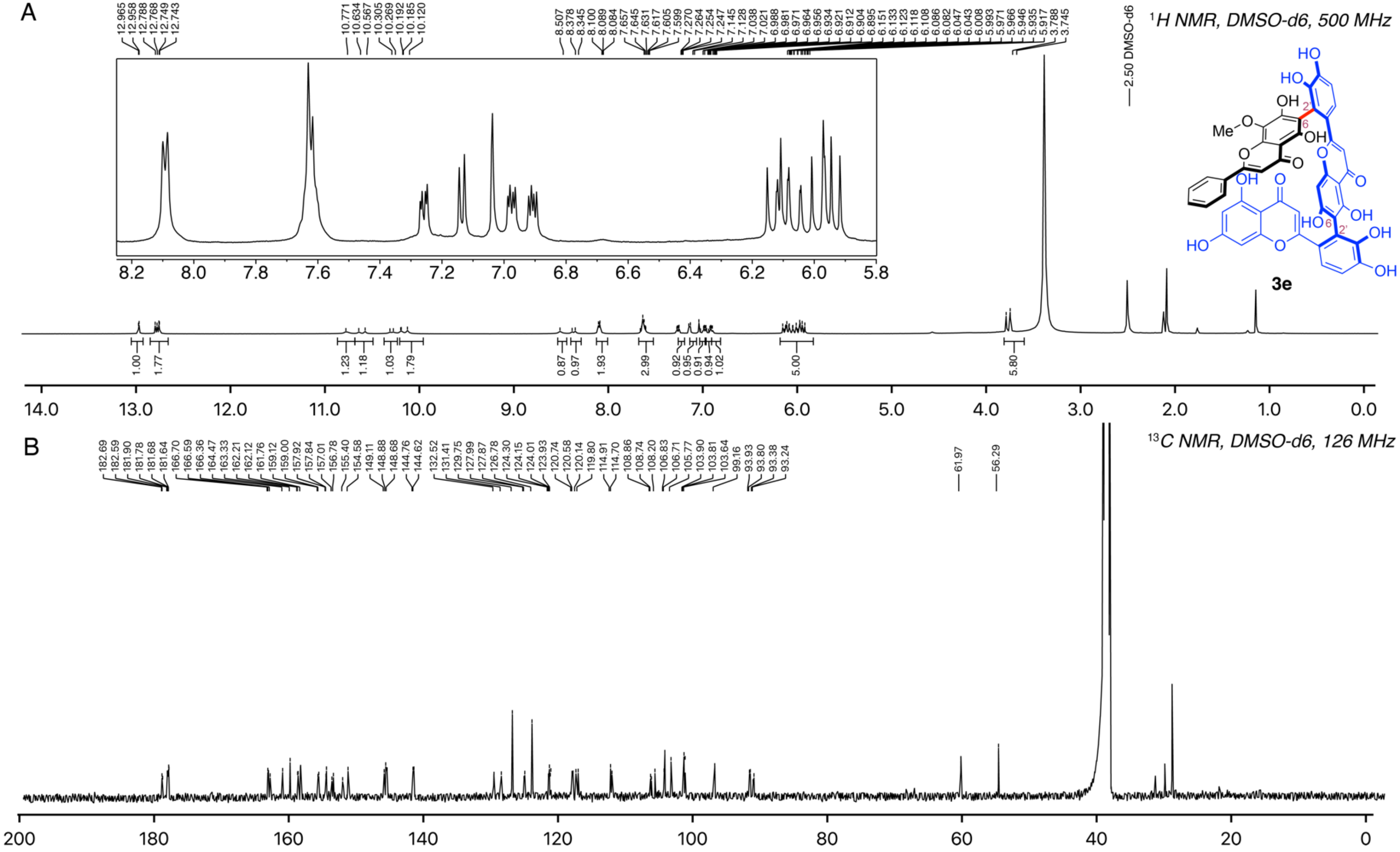

Supplementary Fig. 112. ^{1}H and ^{13}C NMR spectra of 3e.

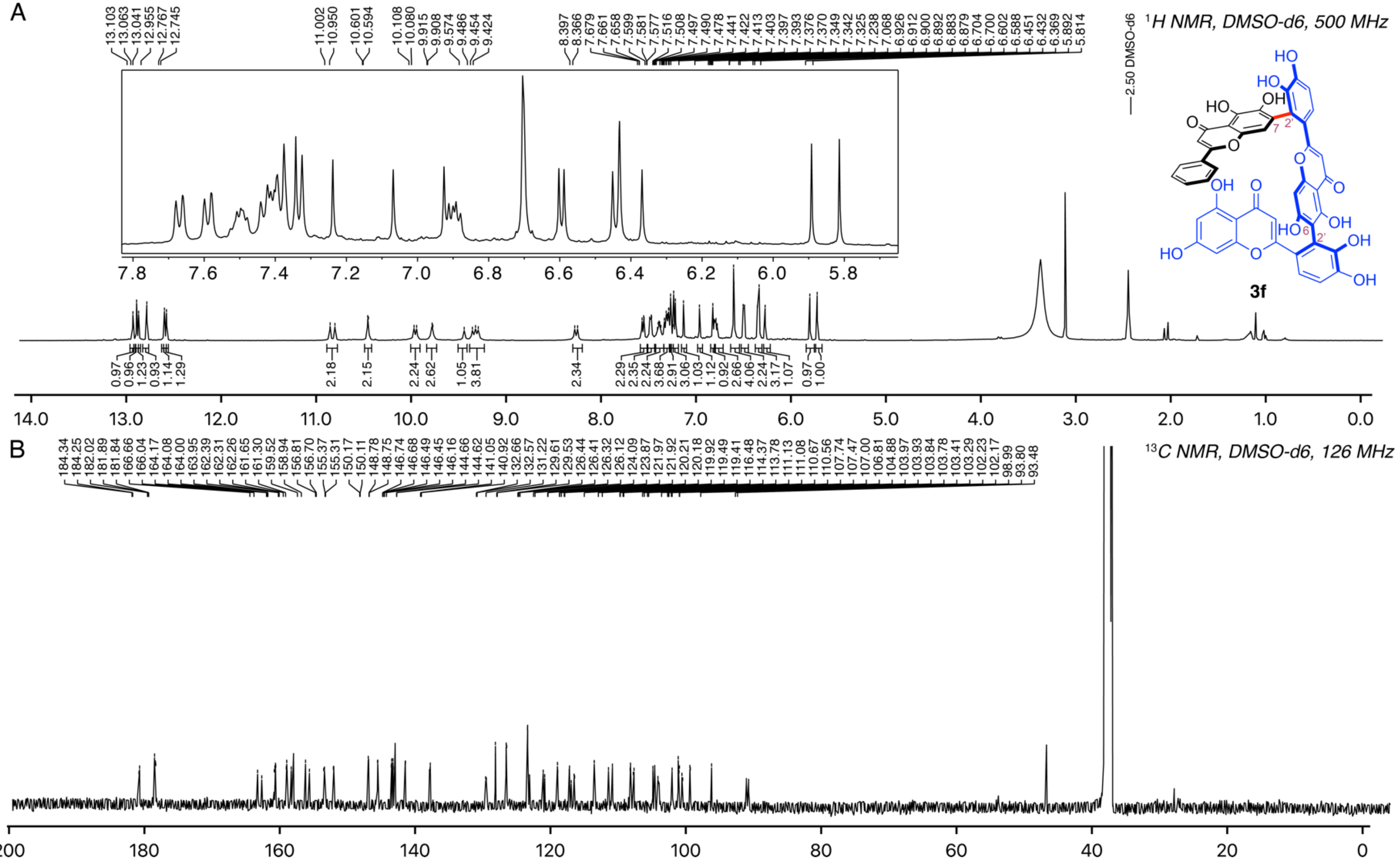

Supplementary Fig. 113. ^{1}H and ^{13}C NMR spectra of 3f.

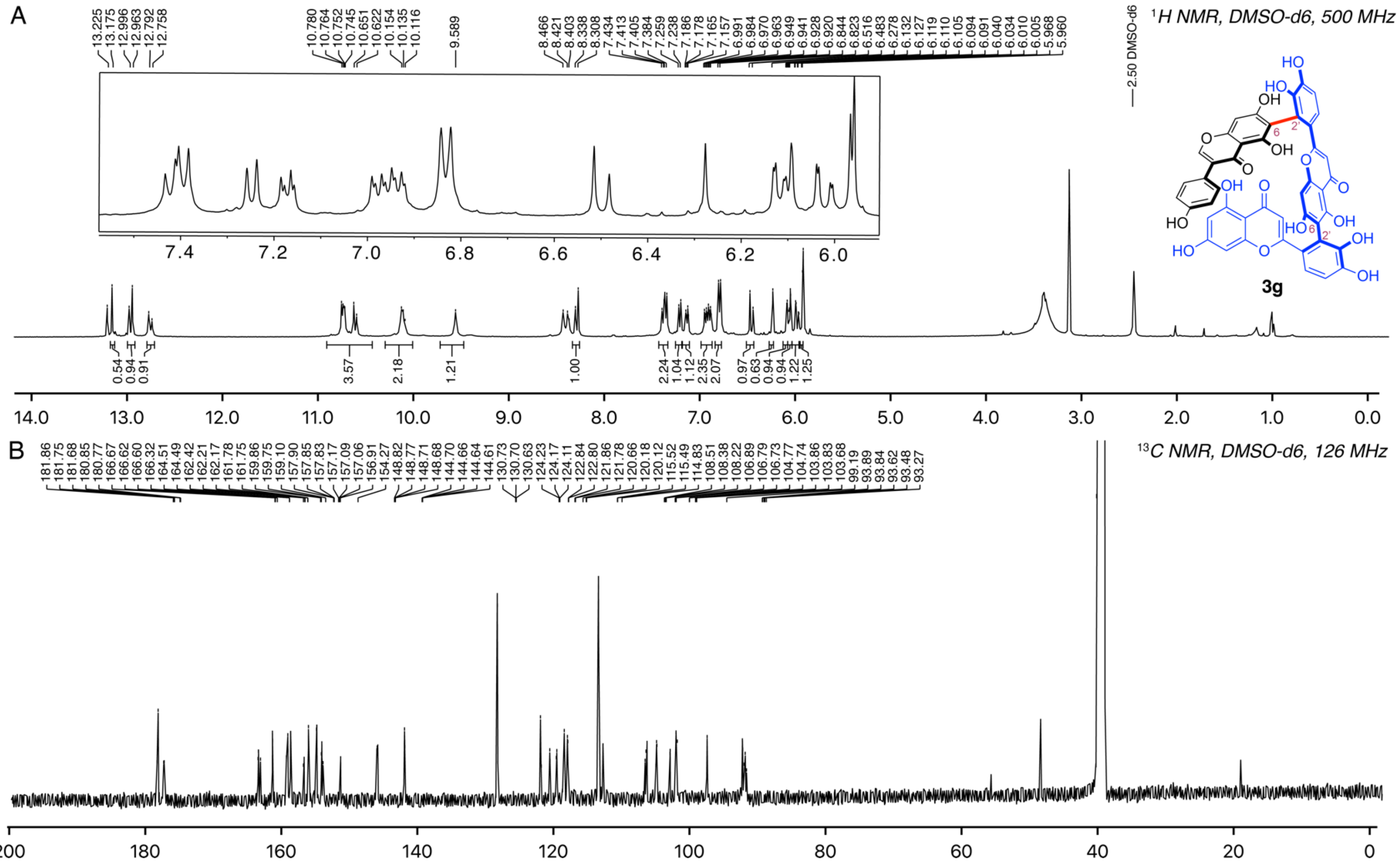

Supplementary Fig. 114. ^{1}H and ^{13}C NMR spectra of 3g.

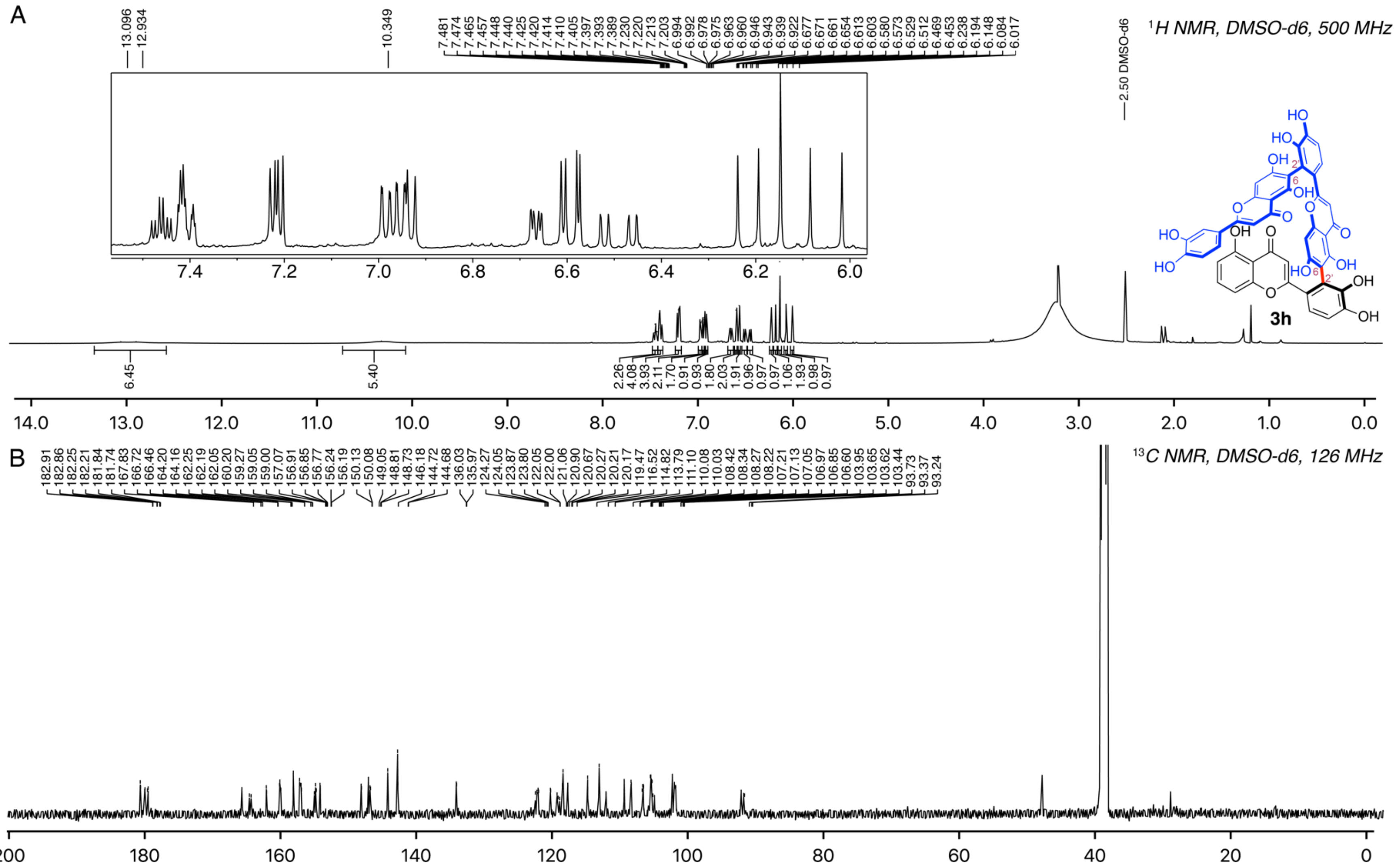

Supplementary Fig. 115. ^{1}H and ^{13}C NMR spectra of 3h.

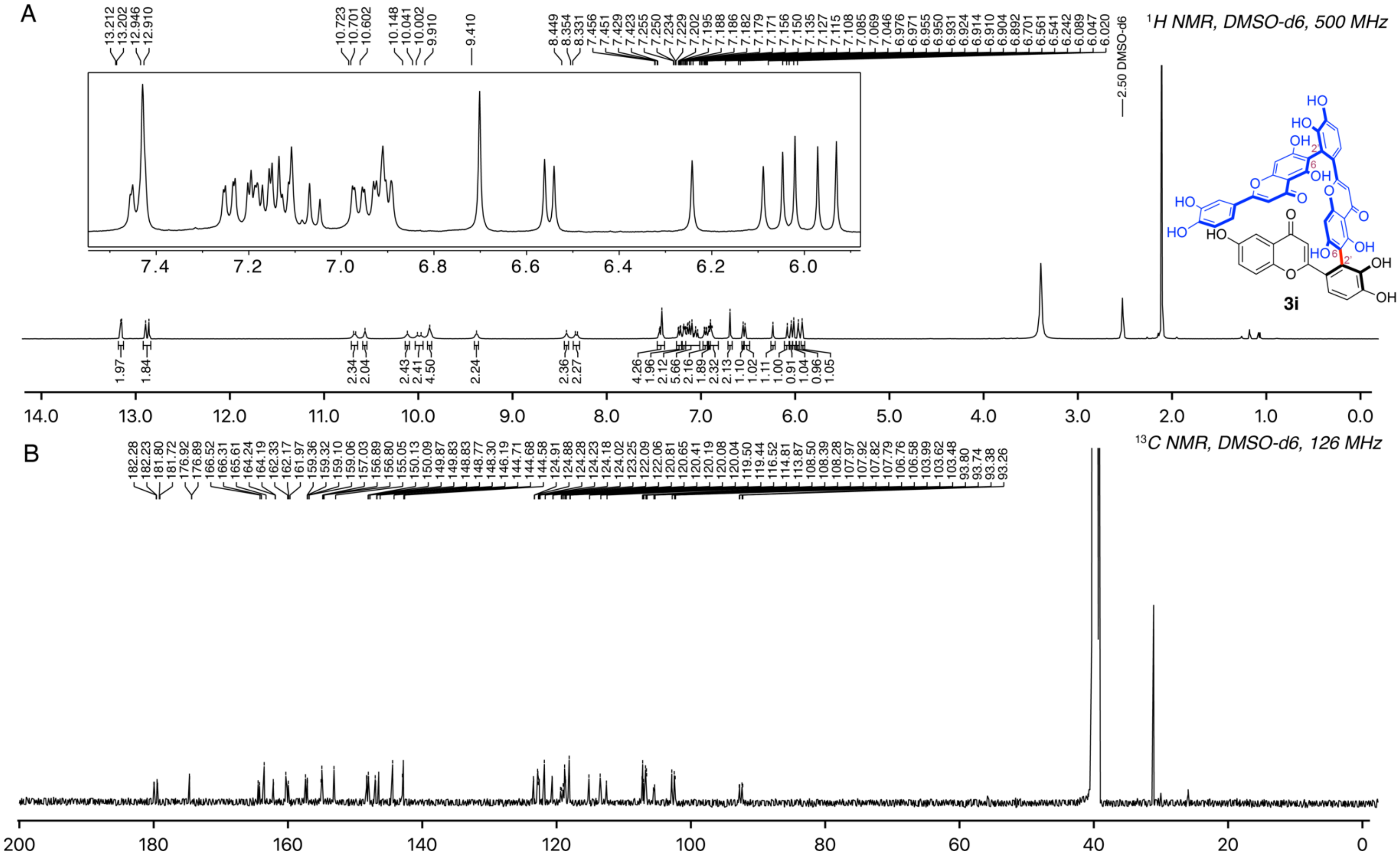

Supplementary Fig. 116. ^{1}H and ^{13}C NMR spectra of 3i.

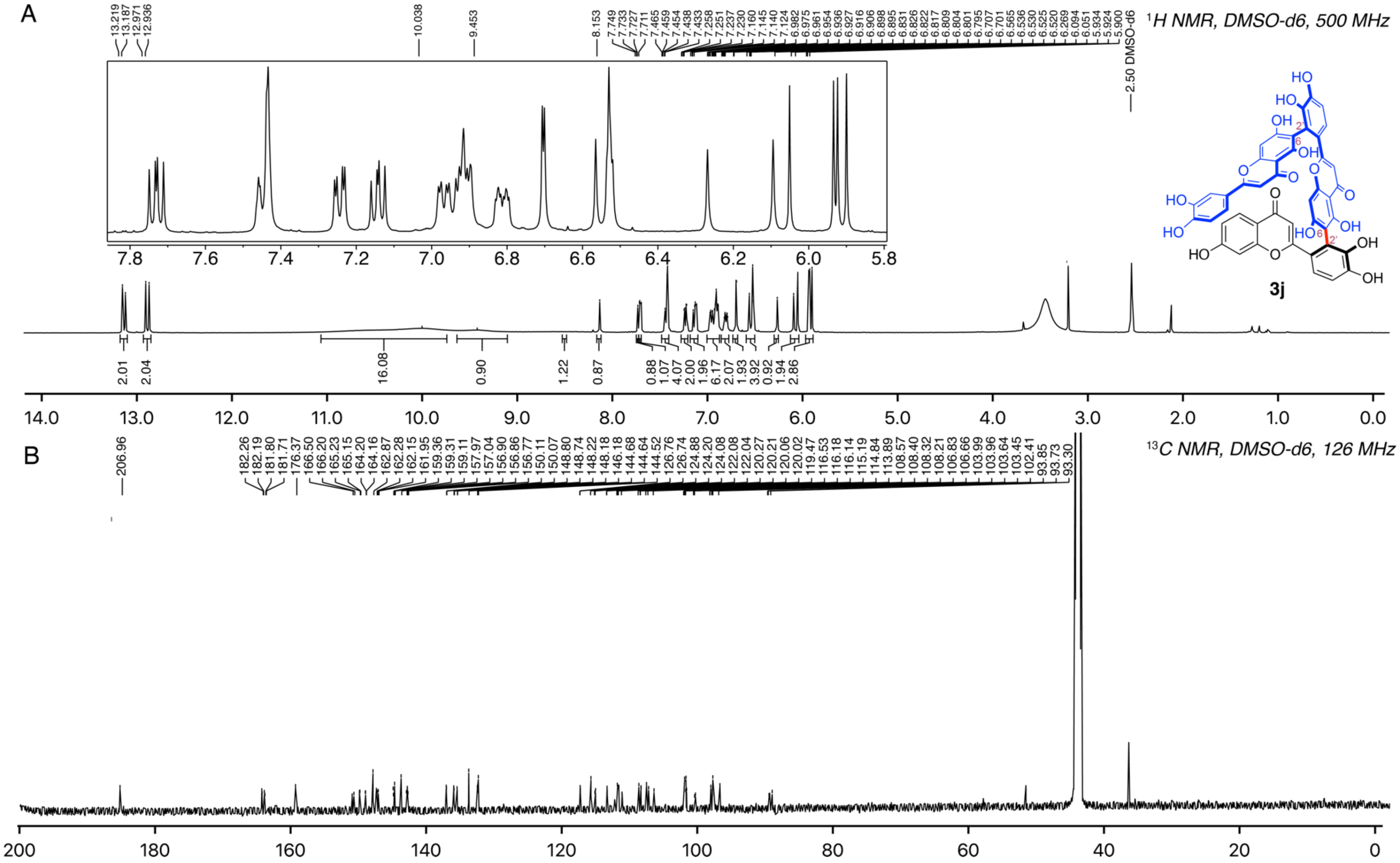

Supplementary Fig. 117. ^{1}H and ^{13}C NMR spectra of 3j.

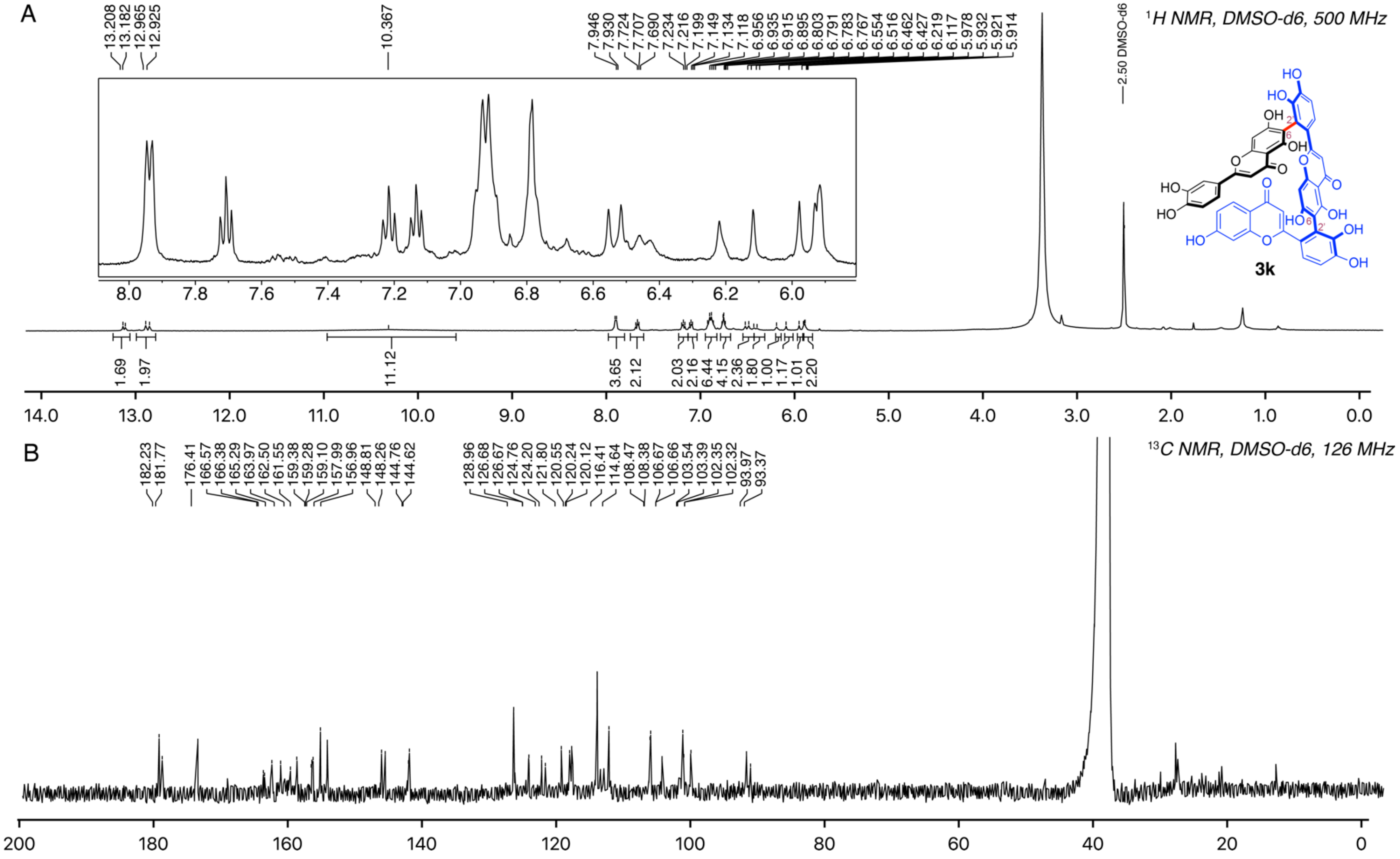

Supplementary Fig. 118. ¹H and ¹³C NMR spectra of 3k.

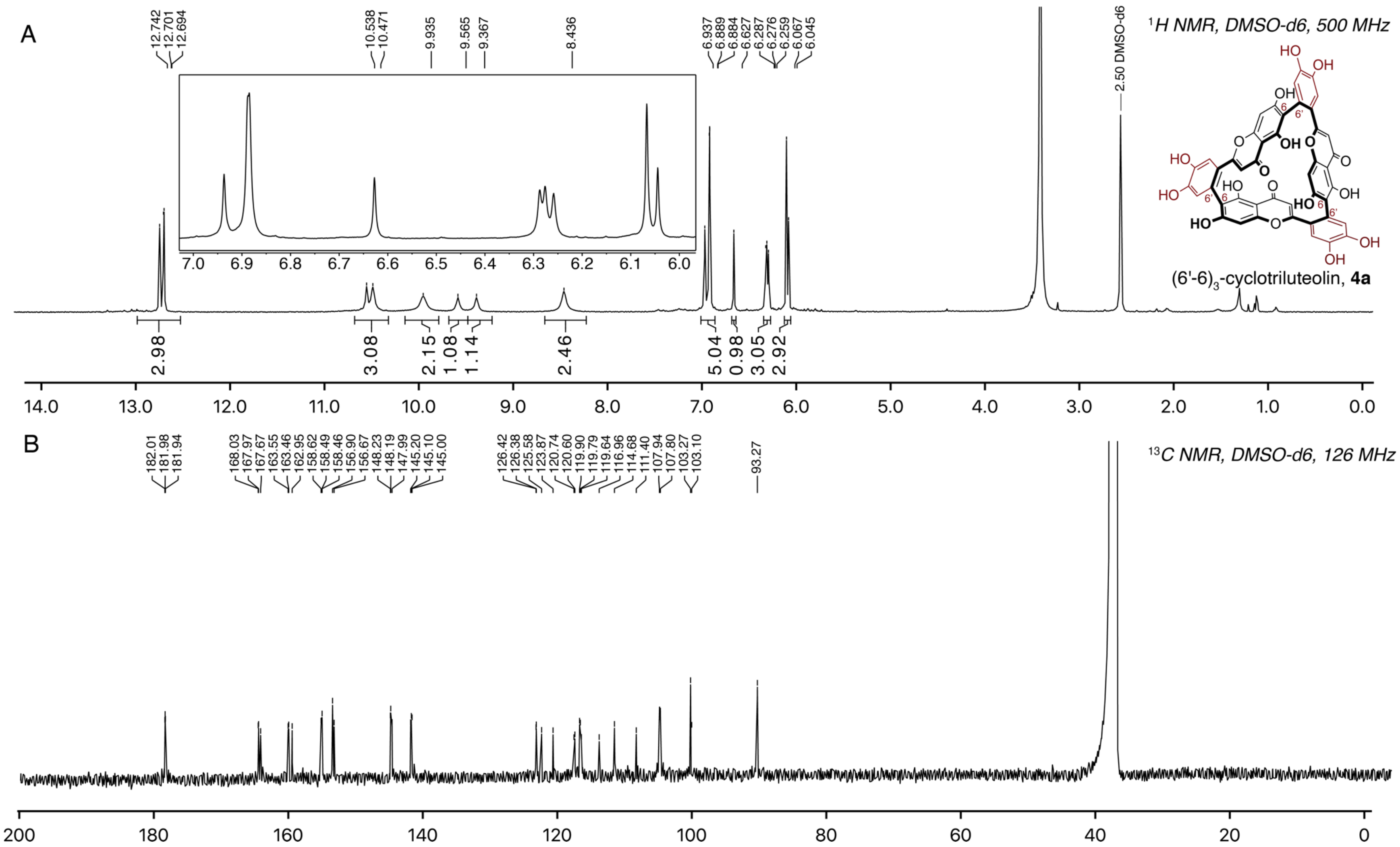

Supplementary Fig. 119. ^{1}H and ^{13}C NMR spectra of 4a.

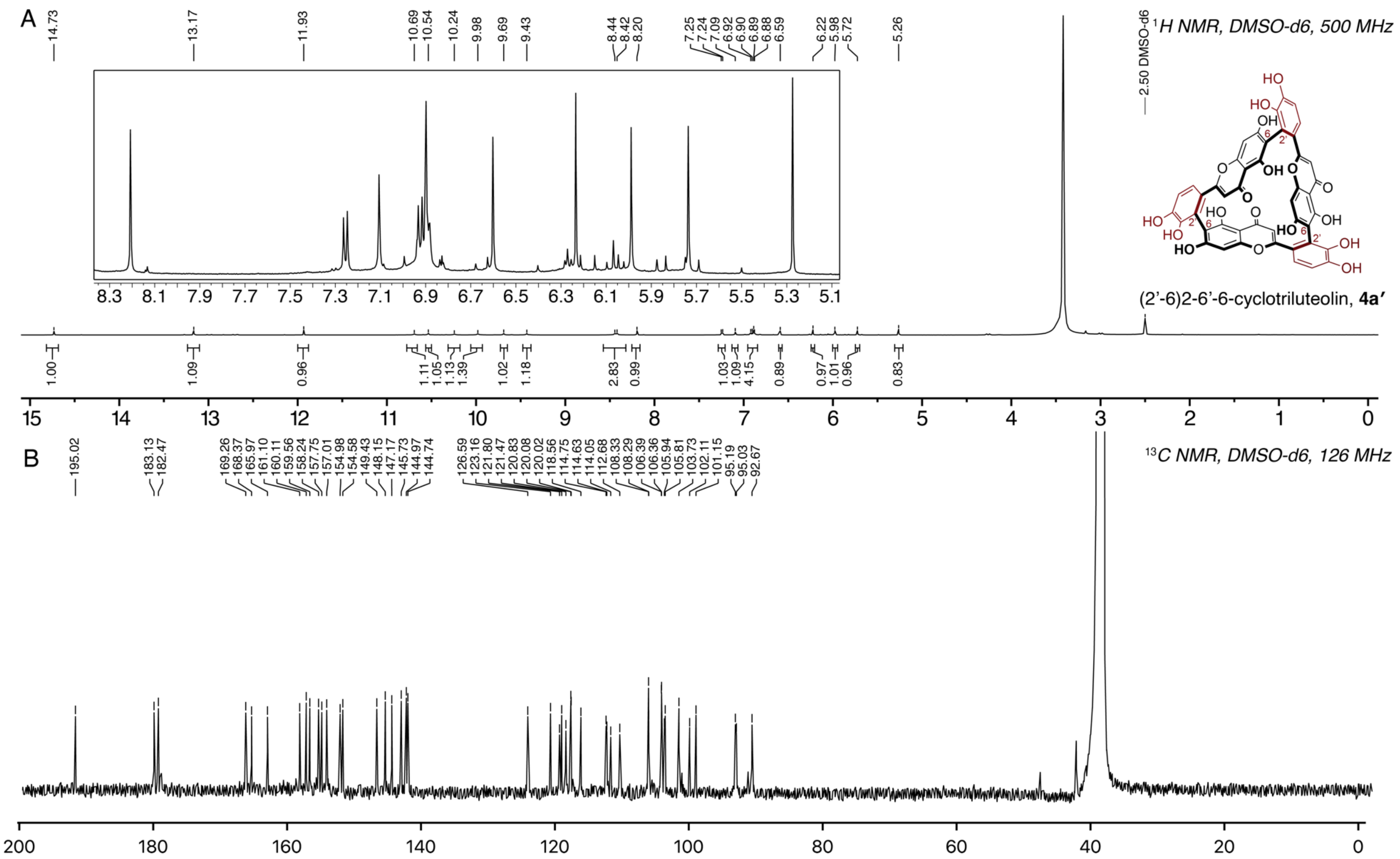

Supplementary Fig. 120. ^{1}H and ^{13}C NMR spectra of 4a′.

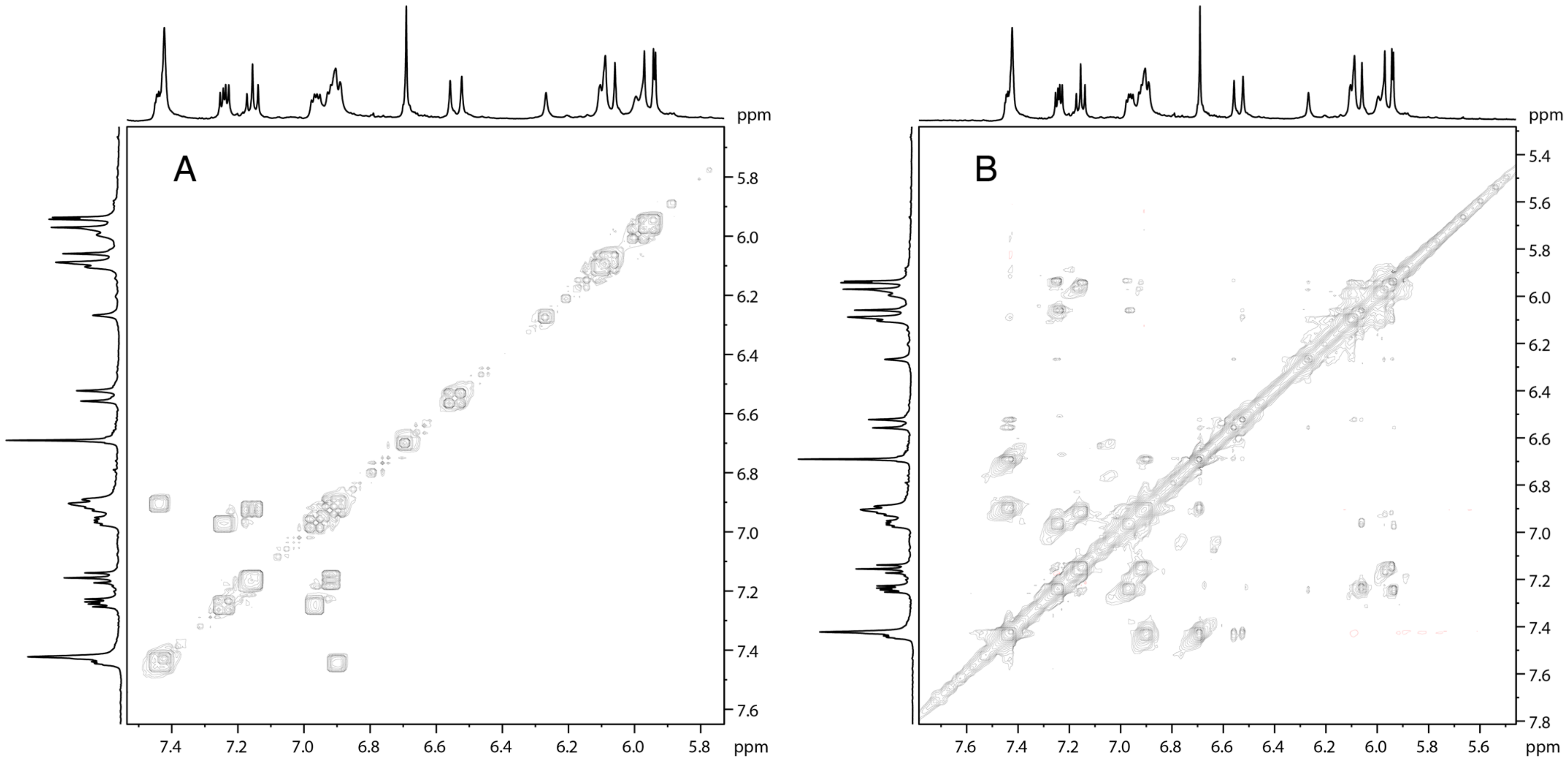

Supplementary Fig. 121. 2D NMR spectra of 3a; A) COSY and B) NOESY.

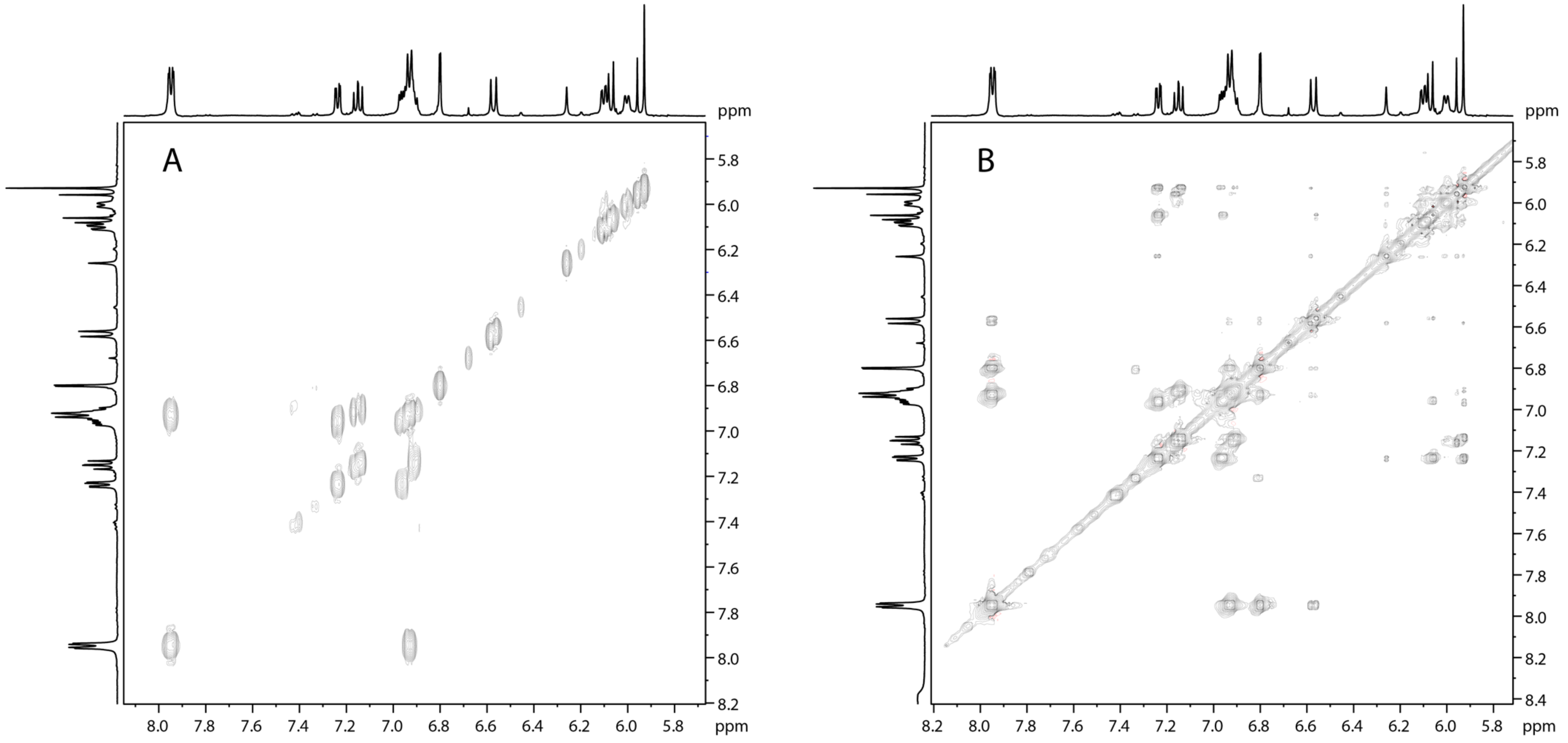

Supplementary Fig. 122. 2D NMR spectra of 3b; A) COSY and B) NOESY.

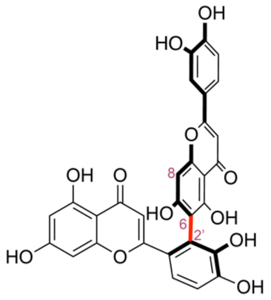

Dicranolomin (**2a**)

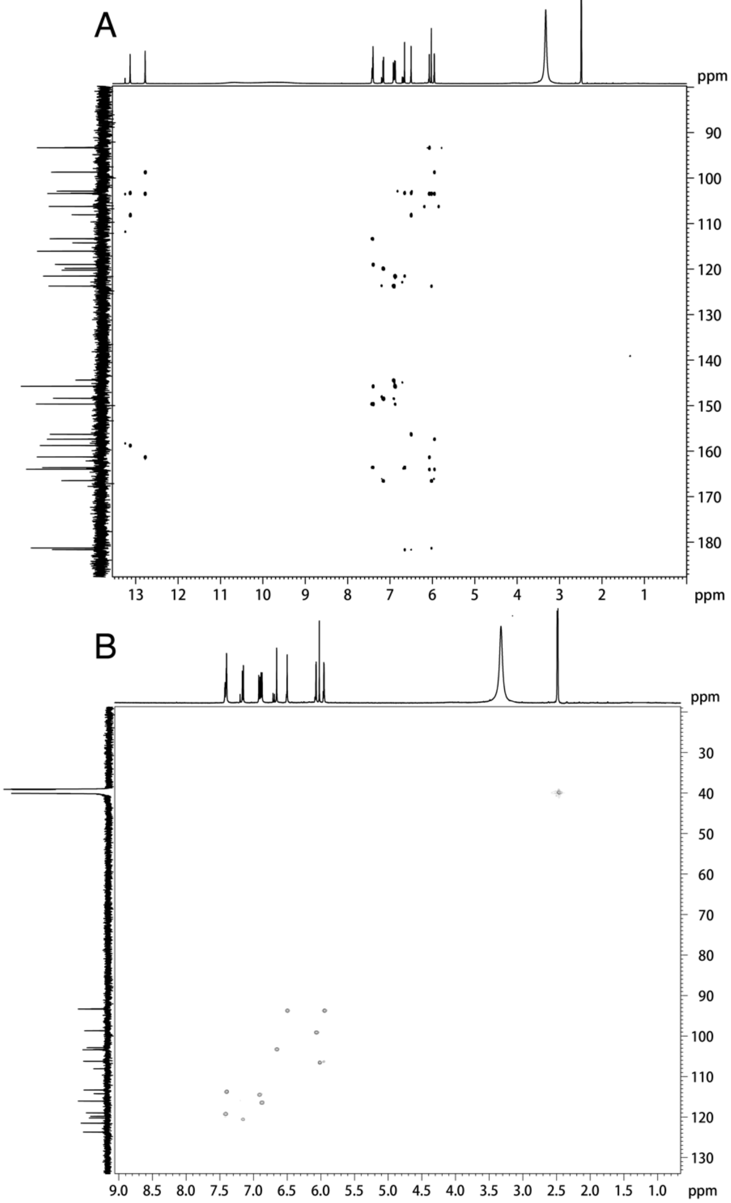

Supplementary Fig. 123. 2D NMR spectrums of 2a; A) HMBC and B) HSQC.

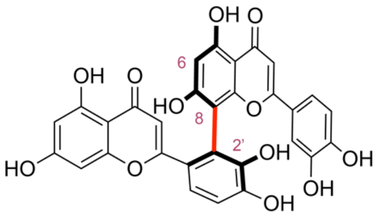

Philonotisflavone (**2a'**)

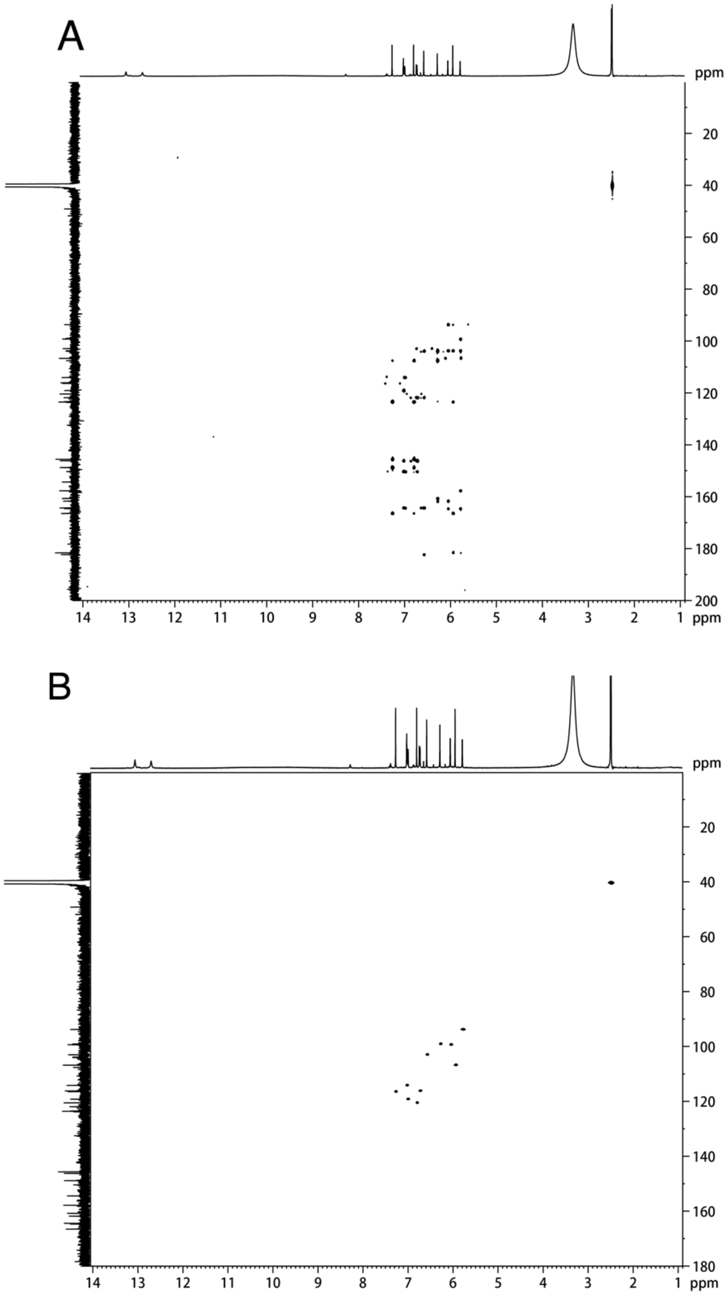

Supplementary Fig. 124. 2D NMR spectra of 2a'; A) HMBC and B) HSQC.

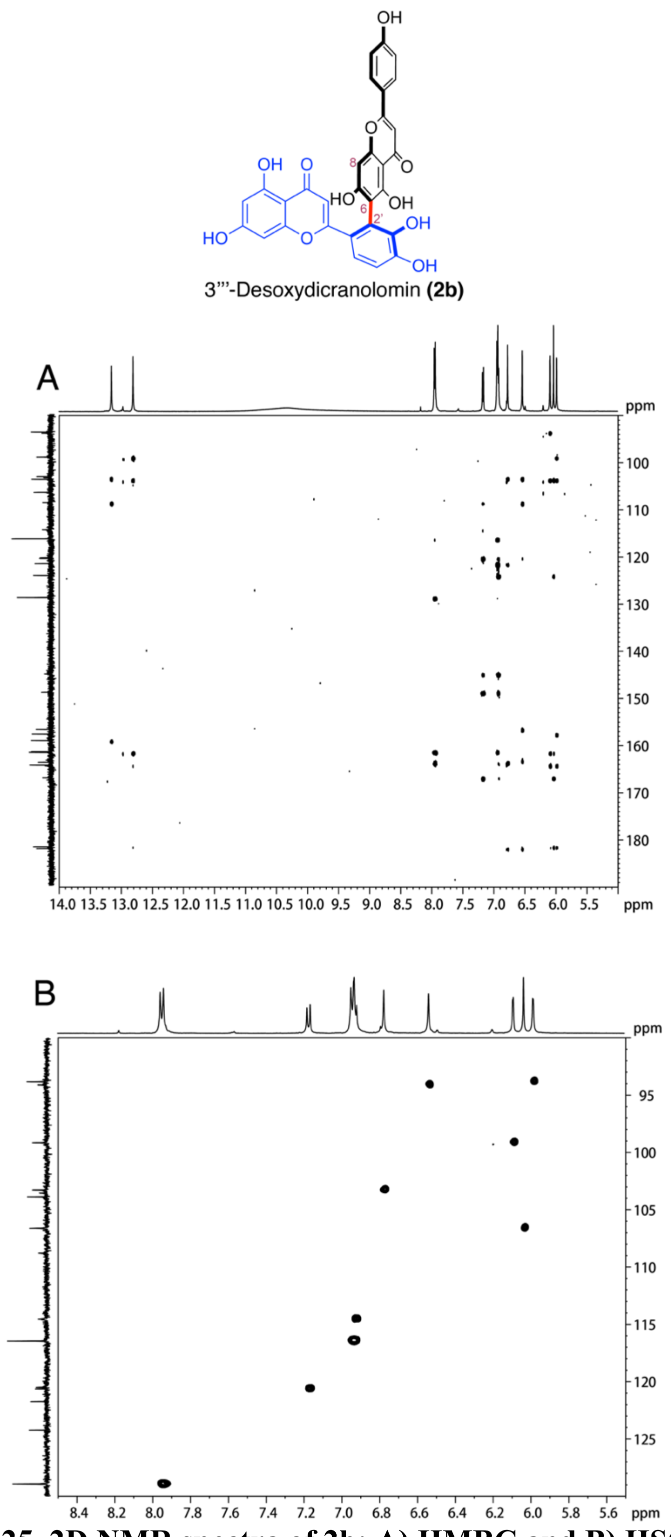

Supplementary Fig. 125. 2D NMR spectra of 2b; A) HMBC and B) HSQC.

2D NMR spectrums of **2c**; **A**) HMBC and **B**) HSQC.

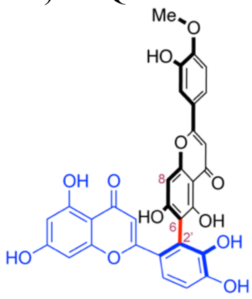

2c

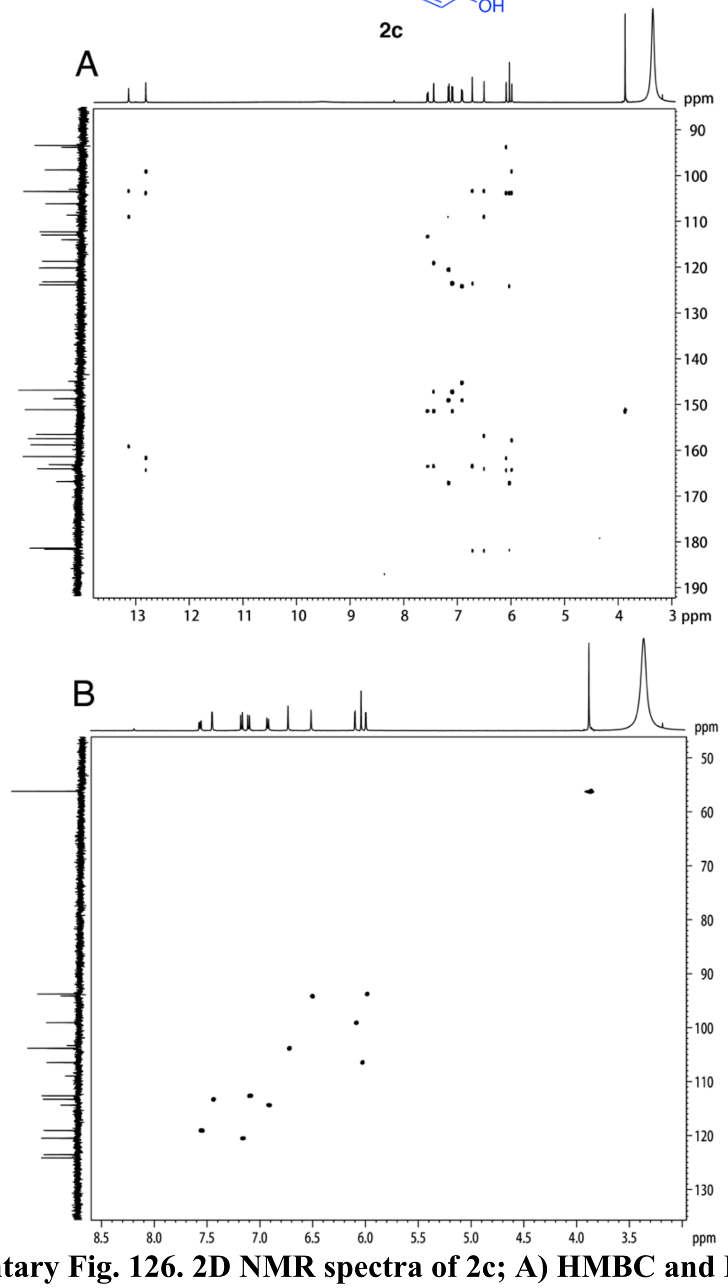

Supplementary Fig. 126. 2D NMR spectra of 2c; A) HMBC and B) HSQC.

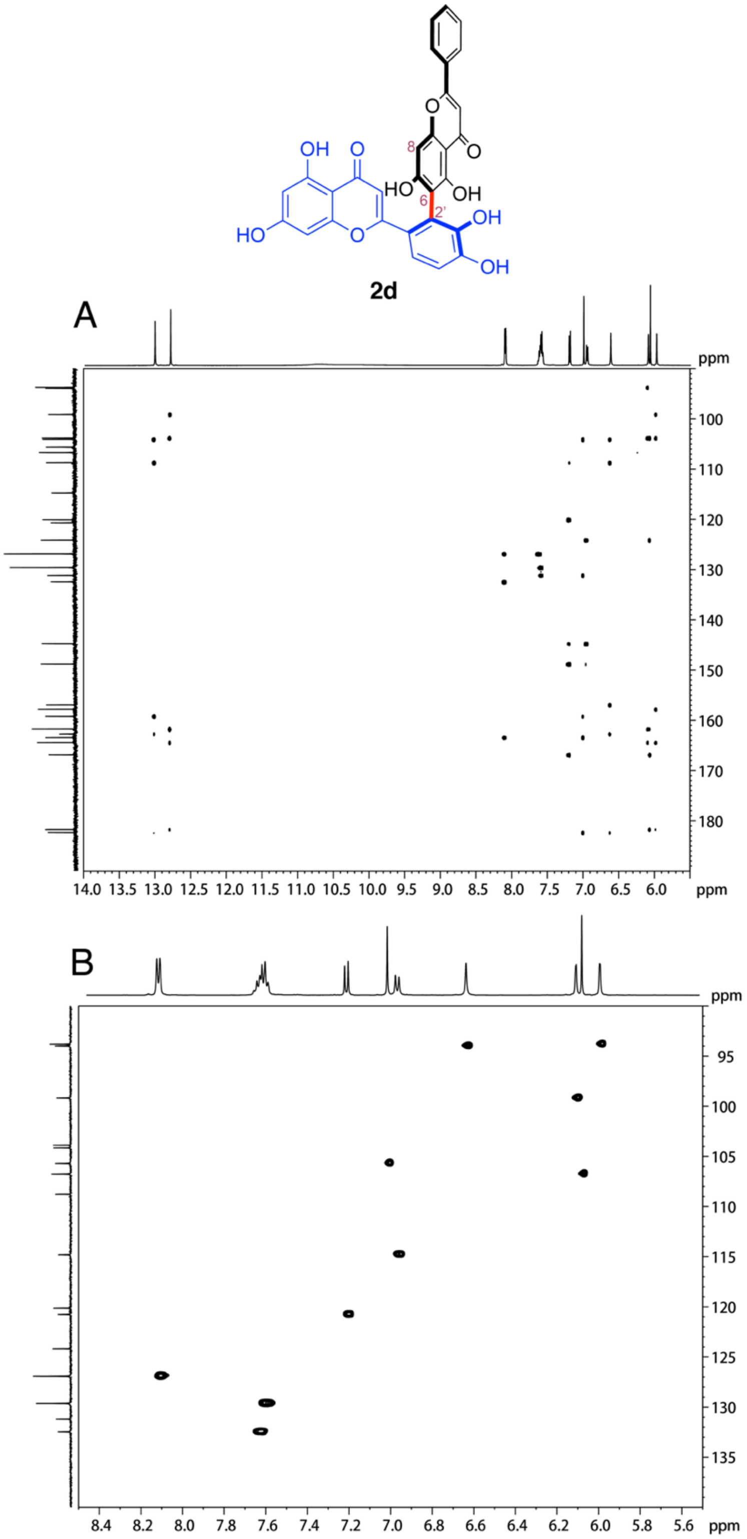

Supplementary Fig. 127. 2D NMR spectra of 2d; A) HMBC and B) HSQC.
