# Supplementary material for: Oxygen mediated oxidative couplings of flavones in alkaline water
Source: Nat Commun. 2022 Oct 28;13:6424. doi: 10.1038/s41467-022-34123-w (PMC9614196; doi:10.1038/s41467-022-34123-w)
Supplement: Supplementary file 3 — Description of Additional Supplementary Files [file 41467_2022_34123_MOESM3_ESM.pdf]

Supplementary Data 1: (a) optimized Gaussian Structures of flavonoid radical anions data; (b) Cartesian coordinated and energies of 2a, 2a', and isomers of cyclotriluteolin 4a.
