# Supplementary material for: Oxygen mediated oxidative couplings of flavones in alkaline water
Source: Nat Commun. 2022 Oct 28;13:6424. doi: 10.1038/s41467-022-34123-w (PMC9614196; doi:10.1038/s41467-022-34123-w)
Supplement: Supplementary file 4 — Supplementary data 1 [file 41467_2022_34123_MOESM4_ESM.pdf]

Supplementary Data 1

1. Optimized Gaussian Structures of flavonoid radical anions

1.1. Luteolin radical dianion

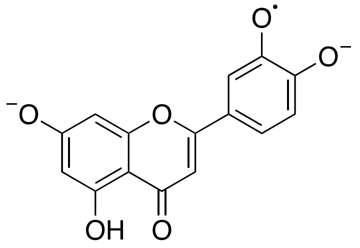

E(SCF) = -1027.35325067

-2 2

C	3.610738	1.960895	0.062608
C	4.348529	0.723908	0.010065
C	3.716884	-0.494361	-0.026053
C	2.293674	-0.593729	-0.012273
C	1.576424	0.622021	0.040842
C	2.178228	1.85537	0.078302
C	1.598785	-1.844271	-0.060031
C	0.154343	-1.764295	-0.052003
C	-0.484889	-0.56847	0.012729
O	0.201844	0.601342	0.06129
C	-1.938011	-0.364687	0.026878
C	-2.81437	-1.488773	0.175199
C	-4.170399	-1.329864	0.177454
C	-4.78795	-0.03623	0.034175
C	-3.883658	1.139608	-0.106366
C	-2.471724	0.903078	-0.103148
O	4.456005	-1.637307	-0.077077
O	2.192749	-2.958198	-0.112144
O	-4.379144	2.29679	-0.224863
O	-6.035009	0.126574	0.029245
O	4.199402	3.087148	0.09335
H	5.433456	0.765372	-0.002299
H	1.574771	2.75588	0.118662
H	-0.403315	-2.689666	-0.110167
H	-2.397383	-2.482005	0.303944
H	-4.830026	-2.18506	0.295649
H	-1.82932	1.77025	-0.21577
H	3.805409	-2.386771	-0.098705

1.2. 3′, 4′ dihydroxyflavone radical anion

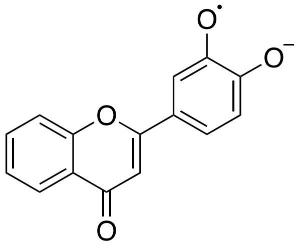

E(SCF) = -877.347360282

-1 2

C	-4.803719	-0.958431	-0.02818
C	-4.216058	0.291811	0.029644
C	-2.819309	0.419916	0.018505
C	-2.040881	-0.735512	-0.051739
C	-2.617965	-2.005219	-0.110242
C	-3.998708	-2.106594	-0.097468
H	-5.883968	-1.055854	-0.019202
H	-4.815156	1.19515	0.085211
C	-2.154936	1.731688	0.089114
H	-1.976637	-2.878444	-0.162425
H	-4.460039	-3.087848	-0.141214
C	-0.030835	0.506805	-0.004048
C	-0.712722	1.682251	0.079121
H	-0.178062	2.620132	0.152616
C	1.425867	0.353246	-0.02246
C	2.264641	1.505849	-0.173169
C	2.000141	-0.89769	0.104887
C	3.624565	1.391545	-0.180996
H	1.815383	2.484907	-0.301499
C	3.418397	-1.087687	0.103499
H	1.387043	-1.785522	0.219861
C	4.284458	0.117894	-0.039462
H	4.255294	2.267657	-0.302478
O	-2.794795	2.789981	0.159551
O	3.951414	-2.227568	0.219463
O	5.534292	-0.005522	-0.03826
O	-0.676096	-0.684317	-0.069868

1.3. 5, 3′, 4′ trihydroxyflavone radical anion

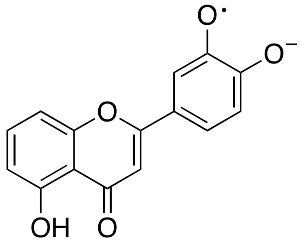

E(SCF) = -952.583156265

-1 2			
C	-3.598444	2.357666	-0.061953
C	-4.462742	1.262685	-0.026151
C	-3.939429	-0.02505	0.006238
C	-2.538141	-0.22043	0.001615
C	-1.710853	0.907365	-0.034207
C	-2.217885	2.201778	-0.066716
C	-1.936665	-1.549584	0.040163
C	-0.500683	-1.584002	0.039046
C	0.238759	-0.440035	-0.003632
O	-0.352159	0.778665	-0.040797
C	1.699238	-0.35164	-0.009827
C	2.491154	-1.543988	-0.094588
C	3.854376	-1.485733	-0.100763
C	4.566318	-0.234083	-0.022441
C	3.749202	1.010741	0.064817
C	2.324945	0.879368	0.064456
O	-4.7811	-1.081856	0.042537
O	-2.63584	-2.5873	0.076443
O	4.326948	2.131966	0.135639
O	5.819001	-0.160685	-0.027828
H	-5.539107	1.394117	-0.022143
H	-1.539012	3.046036	-0.093715
H	-0.021443	-2.552834	0.079844
H	2.005022	-2.511033	-0.165968
H	4.44869	-2.392366	-0.170204
H	1.749425	1.796601	0.130721
H	-4.21665	-1.896067	0.063482
H	-4.016625	3.358862	-0.086318

1.4. 6, 3′, 4′ trihydroxyflavone radical anion

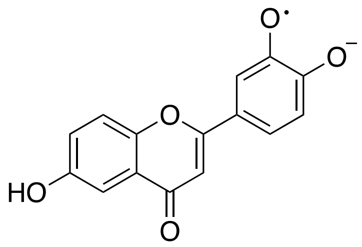

E(SCF) = -952.578149755

-1 2

C	3.7243	-1.891465	-0.101659
C	4.475204	-0.705957	-0.020326
C	3.835755	0.520532	0.041669
C	2.437645	0.577731	0.023116
C	1.705177	-0.605717	-0.056847
C	2.342742	-1.845268	-0.120415
C	1.718551	1.861697	0.094084
C	0.2814	1.751823	0.07894
C	-0.353255	0.549098	-0.010665
O	0.336895	-0.612707	-0.080448
C	-1.80364	0.341938	-0.029247
C	-2.683116	1.462655	-0.186672
C	-4.038104	1.299687	-0.190851
C	-4.651751	0.00445	-0.038906
C	-3.743159	-1.168673	0.10994
C	-2.332396	-0.927901	0.107113
O	2.316262	2.944295	0.169037
O	-4.23509	-2.325893	0.235204
O	-5.896614	-0.162568	-0.033321
H	1.747809	-2.750084	-0.181786
H	-0.291562	2.666576	0.155224
H	-2.269052	2.455956	-0.32356
H	-4.700169	2.151651	-0.317391
H	-1.68722	-1.79201	0.227728
H	4.240745	-2.846088	-0.149487
H	4.409604	1.439083	0.105329
O	5.841758	-0.722078	0.001797
H	6.152033	-1.641266	-0.049844

1.5. 7, 3′, 4′ trihydroxyflavone radical anion

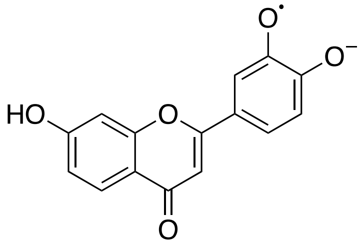

E(SCF) = -952.581304549

-1 2			
C	-3.90315	-1.519468	-0.067623
C	-4.590804	-0.293103	0.005756
C	-3.873438	0.883195	0.056351
C	-2.470214	0.876114	0.036075
C	-1.818868	-0.358257	-0.036548
C	-2.514986	-1.561196	-0.08974
C	-1.679017	2.108371	0.094292
C	-0.247312	1.910662	0.075175
C	0.308196	0.672111	-0.005735
O	-0.457228	-0.448624	-0.062818
C	1.741546	0.369264	-0.028034
C	2.68937	1.429033	-0.208336
C	4.030812	1.179191	-0.220459
C	4.561606	-0.150426	-0.053595
C	3.581905	-1.259948	0.126084
C	2.189026	-0.929393	0.126646
O	-2.20259	3.230273	0.162032
O	4.000532	-2.443161	0.27345
O	5.793493	-0.395163	-0.058787
H	-5.676679	-0.291934	0.022153
H	-1.984602	-2.505	-0.146016
H	0.382196	2.788398	0.140315
H	2.337711	2.444706	-0.356137
H	4.7453	1.984619	-0.365287
H	1.490811	-1.748293	0.265965
H	-4.38368	1.839372	0.113886
O	-4.561555	-2.705931	-0.119433
H	-5.520289	-2.547903	-0.092297

1.6. Dicranolomin radical anion

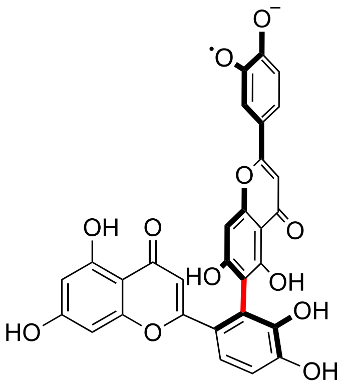

E(SCF) = -2055.54050190

-1 2

C	3.087936	-0.077203	0.353529
O	4.00167	0.695161	-0.284322
C	2.185644	0.434407	1.223481
O	-3.065253	-0.126628	-0.510394
C	2.129749	1.852294	1.488384
O	5.955675	4.905986	-1.232359
C	3.110773	2.656821	0.782401
O	2.334421	4.709547	1.757664
C	3.19188	4.065032	0.939034
O	1.300644	2.354884	2.278953
C	4.148494	4.796695	0.256682
O	3.834502	-5.518417	-0.841844
C	5.043179	4.133575	-0.592507
O	1.362693	-4.551834	-0.886161
C	4.999834	2.75238	-0.773287
O	1.402417	-0.294577	-2.045644
C	4.029689	2.044415	-0.079735
O	0.056382	-3.224153	1.38588
C	3.245055	-1.50164	0.006615
O	-2.424019	-3.110532	2.181209
C	4.532567	-2.036418	-0.023452
O	-6.606478	2.833243	-2.062883
C	4.734644	-3.38502	-0.297818
O	-8.855613	2.207429	-0.618707
C	3.64449	-4.198785	-0.56392
C	2.347156	-3.66082	-0.567228
C	2.126102	-2.31511	-0.278897
C	0.742292	-1.776843	-0.344989
C	0.409777	-0.736353	-1.241691
C	-0.863698	-0.182593	-1.293842
C	-1.828667	-0.686335	-0.432241

C	-1.56364	-1.715278	0.474051
C	-0.252413	-2.249858	0.508865
C	-2.619504	-2.192472	1.350472
C	-3.893696	-1.543949	1.198897
C	-4.079648	-0.546757	0.290467
C	-5.320733	0.186827	0.043742
C	-5.367252	1.18313	-0.914226
C	-6.561003	1.922097	-1.188996
C	-7.78542	1.587784	-0.406173
C	-7.671144	0.54166	0.580196
C	-6.500933	-0.125363	0.796284
H	1.495213	-0.22331	1.737493
H	4.206714	5.872153	0.378795
H	5.692569	2.241461	-1.432871
H	5.382327	-1.397529	0.192269
H	5.729871	-3.816316	-0.308925
H	-1.103451	0.616359	-1.98682
H	-4.704549	-1.875533	1.833466
H	-4.489455	1.440886	-1.497149
H	-8.561928	0.299569	1.15286
H	-6.470018	-0.903395	1.551411
H	6.525803	4.349296	-1.789848
H	1.740598	4.021296	2.152671
H	2.96692	-5.925836	-1.014459
H	0.510761	-4.091808	-0.98142
H	1.070236	0.422431	-2.613314
H	-0.777019	-3.428523	1.890109

2. Cartesian coordinates and energies

2.1. Dicranolomin (2a, Lu-(2′-6)-Lu)

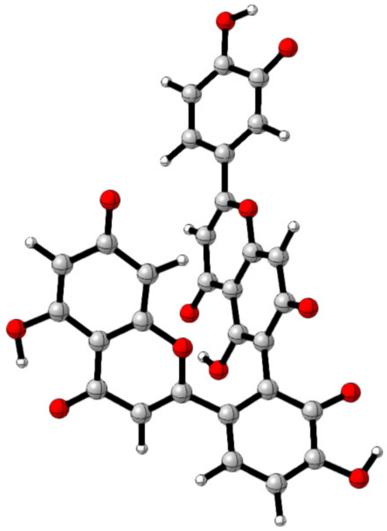

Thermal correction to Gibbs Free Energy= -2053.794327
Sum of electronic and thermal Free Energies= 0.31606
G_sol = -2054.7693473Hartree

C	0.006708	3.452866	-1.964746
C	0.727617	2.218975	-1.829453
C	1.773525	2.125354	-0.942828
C	2.198013	3.190653	-0.118128
C	1.482179	4.41767	-0.247136
C	0.43805	4.54594	-1.129259
C	3.272875	3.006696	0.812265
C	3.841472	1.677618	0.858035
C	3.407466	0.700699	0.023568
C	3.934387	-0.675597	-0.01696
C	5.316814	-0.841461	0.067224
C	3.060608	-1.784302	-0.15306
C	5.870055	-2.126905	0.004436
C	3.601036	-3.089966	-0.22699
C	5.027615	-3.207244	-0.144154
C	-1.191905	-1.239373	-0.190991
C	0.835927	-1.919292	-1.359291
C	-0.485216	-0.940272	0.99205
C	1.582042	-1.628271	-0.147499
C	-3.242169	-0.613482	0.838669
C	-1.185404	-0.469118	2.149826
C	0.927351	-1.150353	0.968016
C	-2.617078	-0.335786	2.010705
O	3.687234	3.93062	1.568857
O	2.432637	0.923738	-0.890619

O	1.847992	5.478126	0.526717
O	-0.962181	3.581263	-2.779545
O	2.923174	-4.191989	-0.343196
O	1.41627	-2.33036	-2.412986
O	-2.553516	-1.05412	-0.244383
O	-0.60423	-0.19467	3.239803
C	-4.686246	-0.498047	0.583621
C	-5.466195	0.336908	1.386704
C	-5.273732	-1.232608	-0.465845
C	-6.843636	0.432449	1.150416
C	-6.652103	-1.162816	-0.731164
C	-7.410587	-0.297887	0.126402
O	1.639125	-0.858881	2.094808
O	-8.751395	-0.247841	-0.154796
O	-7.293451	-1.801136	-1.66284
O	5.494928	-4.496178	-0.211039
C	-0.582452	-1.710417	-1.327428
H	0.446752	1.359579	-2.430064
H	-0.085661	5.494237	-1.204058
H	4.609102	1.458043	1.590179
H	5.96214	0.027306	0.146267
H	6.943977	-2.280425	0.058653
H	2.606231	5.162491	1.084743
H	-5.013646	0.933368	2.171279
H	-4.664016	-1.88271	-1.085534
H	-7.468874	1.081767	1.75665
H	-3.190334	-0.022137	2.87392
H	0.973956	-0.547978	2.765532
H	-8.819086	-0.865603	-0.920319
H	4.65448	-5.005199	-0.299671
H	-1.169825	-1.917127	-2.215842

2.2. Philonotisflavone (2a′, Lu-(2′-8)-Lu)

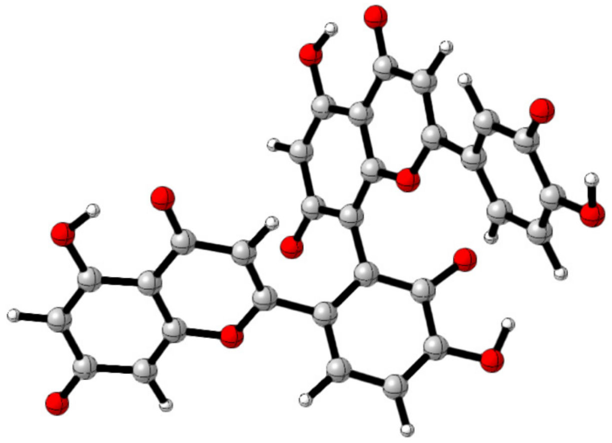

Thermal correction to Gibbs Free Energy= -2053.796191
Sum of electronic and thermal Free Energies= 0.318122
G_sol= -2054.76935Hartree

C	6.896188	-1.088714	-0.739833
C	5.677019	-1.580671	-0.164296
C	4.47837	-0.976699	-0.460806
C	4.358855	0.134109	-1.324428
C	5.568079	0.62704	-1.898382
C	6.780774	0.046237	-1.619971
C	3.073052	0.700805	-1.610042
C	1.949492	0.064413	-0.962579
C	2.126668	-0.977294	-0.115101
C	1.059571	-1.71966	0.583372
C	1.095398	-3.110906	0.501133
C	0.018128	-1.039975	1.264057
C	0.064068	-3.87177	1.066159
C	-1.011298	-1.796672	1.876934
C	-0.961384	-3.218964	1.714442
C	0.021039	0.435465	1.417918
C	-1.041295	1.201562	0.978335
C	1.150314	1.110728	2.02459
C	-1.114865	2.609359	1.090245
C	1.079488	2.542669	2.13876
C	-3.173972	1.170036	-0.080102
C	-2.259032	3.322853	0.602707
C	-0.003572	3.260642	1.69667
C	-3.306841	2.516486	0.021259
O	2.918839	1.684839	-2.3889
O	3.354834	-1.502487	0.131757
O	5.508345	1.694081	-2.743848
O	8.022528	-1.626159	-0.490309

O	-2.009018	-1.323201	2.564712
O	2.170803	0.474572	2.437169
O	-2.064125	0.529998	0.353053
O	-2.365282	4.581031	0.68725
C	-4.17249	0.242613	-0.6326
C	-4.218516	-1.073945	-0.16841
C	-5.085011	0.697731	-1.605018
C	-5.194319	-1.944279	-0.67549
C	-6.071552	-0.148649	-2.136684
C	-6.083492	-1.49116	-1.627349
O	-0.020295	4.616466	1.832646
O	-7.054115	-2.293743	-2.169231
O	-6.953061	0.157645	-3.040366
O	-2.011096	-3.891529	2.28948
H	5.705836	-2.431631	0.507848
H	7.682301	0.445566	-2.074882
H	0.950642	0.437204	-1.153412
H	1.907543	-3.600858	-0.025818
H	0.057328	-4.955384	0.991946
H	4.54773	1.940364	-2.806397
H	-3.540877	-1.409117	0.611234
H	-5.021175	1.714831	-1.982522
H	-5.260879	-2.970546	-0.324996
H	-4.216986	3.000671	-0.310428
H	-0.884367	4.917969	1.444898
H	-7.496448	-1.674946	-2.797729
H	1.916477	3.062627	2.595102
H	-2.527172	-3.149207	2.684897

2.3. (2,6′)3-CTL, CTL1

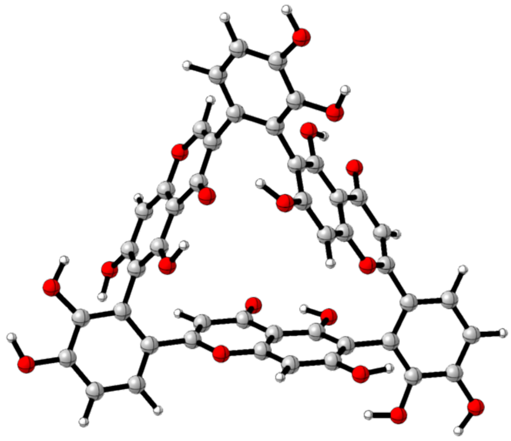

Thermal correction to Gibbs Free Energy= -3081.696561
Sum of electronic and thermal Free Energies= 0.539862
Gsol = -3083.1633997Hartree

0	1		
C	5.79985	-5.52804	2.34674
C	6.25993	-5.66414	3.65418
C	5.58135	-4.98465	4.65165
C	4.4631	-4.17691	4.3957
C	4.01623	-4.0775	3.05392
C	4.67906	-4.74001	2.02432
C	3.82187	-3.44747	5.47086
C	4.38185	-3.65869	6.80045
C	5.44605	-4.4781	6.93745
C	3.8385	-2.89866	7.9488
C	4.65457	-2.01294	8.64786
C	2.4772	-3.01966	8.29046
C	4.1349	-1.22911	9.67826
C	1.95936	-2.21444	9.30318
C	2.78919	-1.31749	9.99452
O	6.465	-6.21743	1.38964
O	2.92749	-3.34801	2.74696
O	6.05033	-5.13077	5.92593
O	2.17181	-0.5845	10.97022
C	0.75238	-6.33185	7.56264
C	1.58003	-4.0333	7.67115
C	0.80721	-7.72636	7.96806
C	-0.28902	-5.88235	6.74061
C	0.52381	-3.65139	6.81905
C	-0.24656	-8.56524	7.44311
C	-0.41404	-4.56049	6.34306

C	-1.19954	-8.05357	6.6315
C	1.69828	-5.37703	8.01622
O	2.71356	-5.75517	8.81673
O	0.38273	-2.35578	6.44451
O	-1.24119	-6.74476	6.28625
C	-2.30032	-8.82584	6.02551
C	-3.10337	-9.62646	6.8308
C	-2.53897	-8.73952	4.63573
C	-4.17089	-10.33409	6.28059
C	-3.60075	-9.46158	4.09755
C	-4.42606	-10.24848	4.92152
O	-5.4635	-10.94214	4.37969
O	2.86584	-2.66929	5.26654
O	1.71079	-8.17028	8.70909
C	0.58877	-7.64989	2.76361
C	-1.63035	-7.97688	3.73729
C	1.97586	-8.04833	2.60083
C	0.10363	-6.50033	2.12446
C	-2.04895	-6.79381	3.094
C	2.76749	-7.18389	1.75975
C	-1.20006	-6.05631	2.27247
C	2.2198	-6.0985	1.16812
C	-0.30887	-8.39083	3.5687
O	0.10229	-9.50819	4.19134
O	-3.3092	-6.32599	3.2484
O	0.91856	-5.75223	1.3229
C	2.94684	-5.19828	0.25225
C	4.16974	-4.62262	0.62988
C	2.41226	-4.95811	-1.01715
C	4.86651	-3.84619	-0.30286
C	3.11005	-4.18644	-1.93144
C	4.35195	-3.63769	-1.57507
O	4.9639	-2.89819	-2.5422
O	2.44605	-9.07063	3.14502
O	0.63895	-2.31526	9.6308
O	-3.92603	-9.47492	2.77109
H	7.11724	-6.285	3.88361
H	5.92682	-4.68963	7.88621
H	5.69964	-1.91684	8.37114
H	6.05973	-6.05044	0.51926
H	2.61587	-2.92131	3.58538
H	2.81562	0.01094	11.38905
H	-0.24661	-9.62391	7.67332

H	2.6118	-6.73042	8.96968
H	1.26764	-1.94114	6.46345
H	-2.91119	-9.67767	7.89745
H	-5.47136	-10.78637	3.41835
H	-1.21414	-4.24925	5.68208
H	3.80733	-7.43058	1.57855
H	1.06175	-9.63109	3.96208
H	-3.78971	-6.86692	3.90133
H	1.46171	-5.3924	-1.31098
H	5.8059	-2.54363	-2.21003
H	-1.54394	-5.15395	1.78202
H	0.45216	-1.68338	10.34756
H	-3.19989	-9.1008	2.24098
H	-4.81396	-10.95238	6.89769
H	4.76546	-0.53417	10.22552
O	6.10371	-3.22722	0.05926
H	6.31454	-3.44167	0.97094
H	2.70243	-4.01888	-2.90646

2.4. (2,6′)2-6,6′-CTL, CTL2

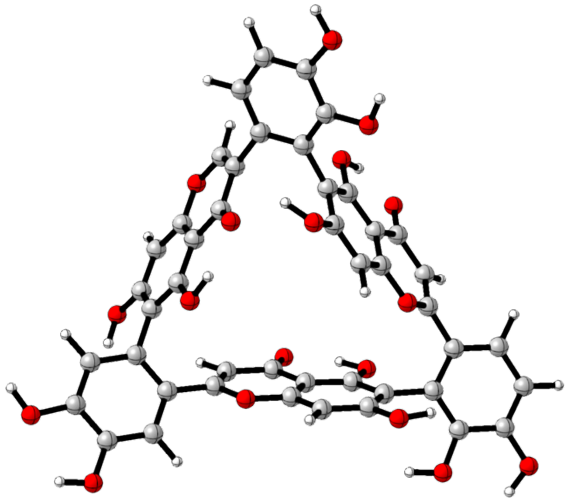

Thermal correction to Gibbs Free Energy= -3081.695956
Sum of electronic and thermal Free Energies= 0.539359
Gsol = -3083.1609781Hartree
0 1

C	3.85608	-1.84321	2.123
C	3.80484	-0.62198	2.78902
C	3.49696	0.50462	2.04296
C	3.25598	0.45645	0.661
C	3.30611	-0.80736	0.01815
C	3.60849	-1.95808	0.74446
C	2.96428	1.66836	-0.08122
C	2.91146	2.8933	0.71374
C	3.13188	2.81517	2.04107
C	2.66413	4.18335	0.03345
C	3.64659	5.16745	0.01631
C	1.45274	4.37246	-0.65606
C	3.44636	6.3515	-0.69447
C	1.26636	5.54884	-1.37869
C	2.26881	6.53235	-1.3968
O	4.11314	-2.94277	2.86586
O	3.04662	-0.9298	-1.28435
O	3.43053	1.68786	2.71245
O	1.96252	7.64196	-2.1391
C	-1.63919	2.59955	0.64775
C	0.32087	3.41264	-0.56177
C	-2.49734	2.59809	1.8239
C	-1.99958	1.87314	-0.49494
C	-0.07436	2.6349	-1.66865
C	-3.69232	1.77864	1.70536
C	-1.23177	1.8596	-1.64689
C	-3.94657	1.08648	0.57521

C	-0.44891	3.36967	0.59992
O	-0.06042	4.08376	1.66306
O	0.66459	2.61832	-2.79275
O	-3.14162	1.12654	-0.51328
C	-5.11643	0.2115	0.36116
C	-6.39822	0.69007	0.61065
C	-4.9355	-1.10274	-0.12022
C	-7.51752	-0.10682	0.37397
C	-6.06194	-1.88719	-0.34899
C	-7.35639	-1.39471	-0.11185
O	-8.43046	-2.1819	-0.34437
O	2.78749	1.66388	-1.31291
O	-2.22359	3.23728	2.85122
C	-1.43534	-2.40155	0.61763
C	-3.58838	-1.70501	-0.30253
C	-0.52911	-2.5574	1.74793
C	-1.02583	-2.74696	-0.67724
C	-3.11043	-2.04417	-1.58469
C	0.78597	-3.06164	1.4006
C	-1.84286	-2.58723	-1.78527
C	1.0975	-3.36826	0.12355
C	-2.73979	-1.88112	0.79556
O	-3.18122	-1.54802	2.00699
O	-3.88049	-1.86391	-2.67714
O	0.22039	-3.25383	-0.90187
C	2.40344	-3.91411	-0.3045
C	3.60177	-3.28261	0.06368
C	2.42532	-5.08598	-1.06581
C	4.81487	-3.87983	-0.30375
C	3.63058	-5.66493	-1.42866
C	4.83219	-5.05847	-1.03186
O	5.96195	-5.71228	-1.43288
O	-0.85826	-2.28203	2.90982
O	3.64738	-6.80751	-2.15016
O	0.10987	5.75242	-2.04894
O	-6.00774	-3.17452	-0.80255
H	3.97376	-0.56043	3.85608
H	3.08287	3.67094	2.70447
H	4.58392	5.00059	0.53705
H	4.12142	-3.71959	2.28116
H	2.86744	-0.02178	-1.63689
H	2.67462	8.29227	-2.06983
H	-4.35039	1.68274	2.55974

H	-0.7539	3.95464	2.36228
H	1.58887	2.80183	-2.53992
H	-6.5233	1.70646	0.96957
H	-8.10593	-3.0528	-0.6274
H	-1.51351	1.25978	-2.50288
H	1.51253	-3.20058	2.19212
H	-2.44228	-1.72629	2.64822
H	-4.67525	-1.36185	-2.42782
H	1.50266	-5.56672	-1.37314
H	6.74808	-5.2004	-1.19662
H	-1.49675	-2.83583	-2.78003
H	5.74977	-3.38605	-0.04707
H	4.57366	-7.04301	-2.32226
H	0.16404	6.62041	-2.48068
H	-5.09382	-3.49792	-0.73283
H	-8.5222	0.25765	0.55689
H	4.21359	7.12152	-0.71426

2.5. 2,6′-(6,6′)$_2$-CTL, CTL3

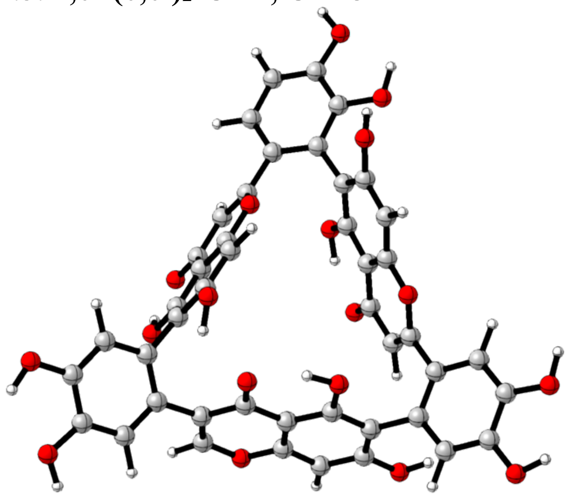

Thermal correction to Gibbs Free Energy= -3081.697273
Sum of electronic and thermal Free Energies= 0.537599
Gsol = -3083.1620439Hartree

0	1		
C	-3.07812	3.04515	2.09948
C	-3.48998	1.87165	2.72685
C	-3.52582	0.71411	1.96728
C	-3.18703	0.69009	0.60513
C	-2.75581	1.90023	0.00382
C	-2.70024	3.07875	0.74614
C	-3.28335	-0.53837	-0.15897
C	-3.69348	-1.71965	0.59839
C	-3.95944	-1.58382	1.91313
C	-3.85887	-3.00924	-0.10652
C	-5.12289	-3.60518	-0.17636
C	-2.77435	-3.60323	-0.7631
C	-5.30762	-4.77204	-0.8981
C	-2.97942	-4.76949	-1.50682
C	-4.23413	-5.3556	-1.58314
O	-3.00561	4.15738	2.8618
O	-2.36938	1.93653	-1.27239
O	-3.90778	-0.42927	2.6011
O	-4.41834	-6.48708	-2.30567
C	0.70786	-2.96393	0.62139
C	-1.3874	-3.07363	-0.63176
C	1.49805	-3.26589	1.80694
C	1.31115	-2.38301	-0.50179
C	-0.7339	-2.45453	-1.71739
C	2.90083	-2.90031	1.71249
C	0.61364	-2.10481	-1.66514

C	3.39017	-2.31266	0.59983
C	-0.66952	-3.29455	0.54402
O	-1.29267	-3.84987	1.58998
O	-1.40055	-2.18886	-2.85778
O	2.63543	-2.05717	-0.49171
C	4.80144	-1.91513	0.40311
C	5.7898	-2.84777	0.69989
C	5.1426	-0.64693	-0.12336
C	7.13435	-2.56893	0.46112
C	6.48617	-0.40219	-0.38304
C	7.47583	-1.35172	-0.09856
O	8.74724	-0.95548	-0.41093
O	-3.04788	-0.57817	-1.38009
O	1.00934	-3.79085	2.81928
C	2.23288	1.6797	0.59929
C	4.12405	0.41886	-0.31815
C	1.35642	2.01878	1.71122
C	2.0292	2.24798	-0.66423
C	3.90077	1.06091	-1.55462
C	0.29811	2.95452	1.38258
C	2.85147	1.96211	-1.7392
C	0.16921	3.45142	0.13342
C	3.30628	0.77208	0.76275
O	3.54561	0.22989	1.95897
O	4.66688	0.8114	-2.63759
O	1.01276	3.1363	-0.87765
C	-0.84207	4.45307	-0.26128
C	-2.19005	4.32456	0.11086
C	-0.42385	5.57452	-0.98597
C	-3.08287	5.35753	-0.19756
C	-1.32017	6.58206	-1.29194
C	-2.65858	6.48813	-0.88259
O	-3.53243	7.4768	-1.17177
O	1.50812	1.55601	2.85125
O	-1.01131	7.7231	-1.97787
O	6.85402	0.78967	-0.94517
O	-6.50246	-5.42985	-1.02836
H	-3.74799	1.86307	3.77769
H	-4.24324	-2.41262	2.55176
H	-5.96984	-3.13332	0.3175
H	-2.71985	4.90283	2.30701
H	-2.49953	1.02966	-1.64741
H	-5.35226	-6.74042	-2.22869

H	3.53923	-3.04845	2.57433
H	-0.60979	-3.96359	2.30192
H	-2.33305	-2.02542	-2.61972
H	5.50169	-3.8175	1.09135
H	9.38398	-1.62758	-0.13107
H	1.09406	-1.61979	-2.50516
H	-0.37472	3.26876	2.17068
H	2.85115	0.57471	2.58248
H	5.57586	0.62265	-2.347
H	0.61587	5.65672	-1.29276
H	-3.06061	8.15672	-1.68034
H	2.70018	2.42837	-2.70405
H	-4.13571	5.26856	0.05295
H	-0.09311	7.69305	-2.28061
H	7.82416	0.80579	-1.008
H	7.90226	-3.30498	0.68272
H	-2.15137	-5.24465	-2.0226
H	-7.1932	-4.95792	-0.54372

2.6. (6,6′)3-CTL, CTL4

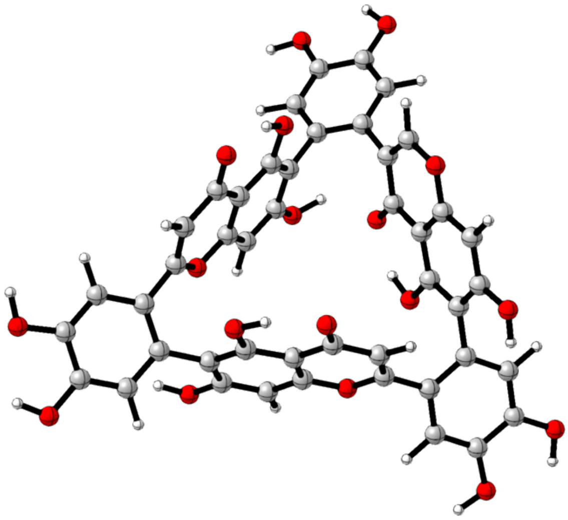

Thermal correction to Gibbs Free Energy= -3081.699263
Sum of electronic and thermal Free Energies= 0.53761
Gsol = -3083.1639092Hartree

0	1		
C	3.87008	-2.1078	2.11287
C	3.90723	-0.87027	2.7508
C	3.59623	0.25099	1.99971
C	3.26562	0.18392	0.63696
C	3.22019	-1.09514	0.02496
C	3.52392	-2.24185	0.75701
C	2.99207	1.39189	-0.11714
C	3.03829	2.63557	0.64787
C	3.3379	2.57496	1.96147
C	2.83848	3.92796	-0.0453
C	3.88412	4.85648	-0.07692
C	1.63942	4.19786	-0.71547
C	3.75274	6.04691	-0.77458
C	1.52364	5.39186	-1.43686
C	2.56476	6.30353	-1.47101
O	4.1361	-3.19632	2.86615
O	2.86285	-1.23543	-1.25299
O	3.62099	1.45107	2.64251
O	2.53916	7.49416	-2.14704
C	-1.57681	2.61061	0.54849
C	0.45026	3.30402	-0.62935
C	-2.45969	2.66892	1.70578
C	-1.95166	1.8874	-0.59143
C	0.03106	2.53737	-1.73622
C	-3.68705	1.90047	1.57475

C	-1.16657	1.82554	-1.73002
C	-3.94704	1.19834	0.45198
C	-0.34499	3.31432	0.51756
O	0.05783	4.0234	1.57772
O	0.78109	2.48296	-2.85457
O	-3.12766	1.19693	-0.62397
C	-5.14347	0.35945	0.23141
C	-6.40677	0.90744	0.47101
C	-5.02829	-0.96253	-0.2301
C	-7.54965	0.16551	0.22661
C	-6.19396	-1.69566	-0.4826
C	-7.44953	-1.14198	-0.26778
O	-8.56316	-1.86541	-0.51885
O	2.75355	1.36778	-1.33887
O	-2.18062	3.31388	2.72738
C	-1.56886	-2.33969	0.55461
C	-3.70119	-1.61267	-0.39179
C	-0.66007	-2.46102	1.68554
C	-1.1897	-2.79301	-0.71554
C	-3.2619	-2.07774	-1.64548
C	0.6249	-3.05213	1.36589
C	-2.01613	-2.67861	-1.82082
C	0.9128	-3.4563	0.10999
C	-2.84924	-1.7544	0.70575
O	-3.26039	-1.32598	1.90113
O	-4.03424	-1.95227	-2.74566
O	0.03331	-3.36797	-0.91605
C	2.18042	-4.10656	-0.27896
C	3.42206	-3.57816	0.10879
C	2.12721	-5.29675	-1.01245
C	4.58875	-4.29001	-0.19387
C	3.28939	-5.98393	-1.31153
C	4.5327	-5.49165	-0.88712
O	5.66736	-6.16759	-1.16959
O	-0.96246	-2.09384	2.83015
O	-8.82831	0.61256	0.41221
O	4.77032	6.94166	-0.80087
O	3.34746	-7.16038	-2.00439
H	4.15158	-0.79277	3.80209
H	3.37877	3.4478	2.60322
H	4.82568	4.65035	0.422
H	4.09648	-3.98814	2.30356
H	2.71756	-0.32792	-1.62054

H	1.69147	7.59559	-2.6013
H	-4.35716	1.83697	2.42297
H	-0.65118	3.93907	2.26675
H	1.71546	2.55573	-2.57881
H	-6.48766	1.93323	0.82312
H	-9.33369	-1.32407	-0.28093
H	-1.46073	1.22786	-2.5833
H	1.3472	-3.18119	2.16246
H	-2.52079	-1.49789	2.54341
H	-4.83817	-1.45838	-2.51372
H	1.16405	-5.68784	-1.33085
H	5.4288	-6.95616	-1.68416
H	-1.69863	-3.01608	-2.79882
H	-8.81993	1.50059	0.79539
H	4.48837	7.69438	-1.34543
H	0.5948	5.60817	-1.96004
H	-6.13512	-2.73006	-0.80903
H	5.56302	-3.88844	0.06831
H	2.46459	-7.41528	-2.30638
