# Supplementary material for: Oxygen mediated oxidative couplings of flavones in alkaline water
Source: Nat Commun. 2022 Oct 28;13:6424. doi: 10.1038/s41467-022-34123-w (PMC9614196; doi:10.1038/s41467-022-34123-w)
Supplement: Supplementary file 5 — Reporting Summary [file 41467_2022_34123_MOESM5_ESM.pdf]

nature portfolio

Corresponding author(s): Huang Dejian, K. N. Houk

Last updated by author(s): Oct 3, 2022

Reporting Summary

Nature Portfolio wishes to improve the reproducibility of the work that we publish. This form provides structure for consistency and transparency in reporting. For further information on Nature Portfolio policies, see our Editorial Policies and the Editorial Policy Checklist.

Statistics

For all statistical analyses, confirm that the following items are present in the figure legend, table legend, main text, or Methods section.

n/a	Confirmed	
☐	☒	The exact sample size (*n*) for each experimental group/condition, given as a discrete number and unit of measurement
☒	☐	A statement on whether measurements were taken from distinct samples or whether the same sample was measured repeatedly
☒	☐	The statistical test(s) used AND whether they are one- or two-sided<br>*Only common tests should be described solely by name; describe more complex techniques in the Methods section.*
☒	☐	A description of all covariates tested
☒	☐	A description of any assumptions or corrections, such as tests of normality and adjustment for multiple comparisons
☐	☒	A full description of the statistical parameters including central tendency (e.g. means) or other basic estimates (e.g. regression coefficient) AND variation (e.g. standard deviation) or associated estimates of uncertainty (e.g. confidence intervals)
☒	☐	For null hypothesis testing, the test statistic (e.g. *F*, *t*, *r*) with confidence intervals, effect sizes, degrees of freedom and *P* value noted<br>*Give P values as exact values whenever suitable.*
☒	☐	For Bayesian analysis, information on the choice of priors and Markov chain Monte Carlo settings
☒	☐	For hierarchical and complex designs, identification of the appropriate level for tests and full reporting of outcomes
☒	☐	Estimates of effect sizes (e.g. Cohen's *d*, Pearson's *r*), indicating how they were calculated

Our web collection on statistics for biologists contains articles on many of the points above.

Software and code

Policy information about availability of computer code

Data collection	Empower 3 software was applied for waters HPLC data collection.<br>TopSpin 4.0.5 software was applied for NMR data collection.
Data analysis	MestReNova v14.2.1 was applied for NMR data analysis.<br>GraphPad Prism. 9 was used to generate all graphs and plots.<br>MicroSoft Excel v16 was used for analyzing plate reader data.

For manuscripts utilizing custom algorithms or software that are central to the research but not yet described in published literature, software must be made available to editors and reviewers. We strongly encourage code deposition in a community repository (e.g. GitHub). See the Nature Portfolio guidelines for submitting code & software for further information.

Data

Policy information about availability of data

All manuscripts must include a data availability statement. This statement should provide the following information, where applicable:

- Accession codes, unique identifiers, or web links for publicly available datasets
- A description of any restrictions on data availability
- For clinical datasets or third party data, please ensure that the statement adheres to our policy

1. Supplementary information include these data: (a) yields and spectroscopic data of the flavonoids synthesized; (b) ESR spectra data of the flavonoid radicals; (c)

single crystal X-ray diffraction data for compounds 2a, 2b, 2e, and 4a, which were also deposited in Cambridge Data Center under deposition numbers of 2044714 (2a), 2044715 (2b), 2044716 (2e), and 2182228 (4a) and can be found at (https://www.ccdc.cam.ac.uk/structures/); (e) Semi-preparative HPLC separation conditions of the products; (f) LC-MS and 1H NMR data for atropisomers of triflavonoids; (g) 13C NMR data of luteolin under various pH conditions; (h) bioactivity data on alpha-amylase inhibition activity and antifungal activity; (i) molecular docking simulation data for flavone dimers with alpha-amylase; (j) Reaction condition optimization data; (k) 1H and 13C NMR spectra of all the synthesized compounds.
2. Supplementary dataset includes: (a) optimized Gaussian Structures of flavonoid radical anions data; (b) Cartesian coordinated and energies of 2a, 2a', and isomers of cyclotriluteolin 4a.
These data are also available from the corresponding authors upon request.

Human research participants

Policy information about studies involving human research participants and Sex and Gender in Research.

Reporting on sex and gender	N/A
Population characteristics	N/A
Recruitment	N/A
Ethics oversight	N/A

Note that full information on the approval of the study protocol must also be provided in the manuscript.

Field-specific reporting

Please select the one below that is the best fit for your research. If you are not sure, read the appropriate sections before making your selection.

[x] Life sciences [ ] Behavioural & social sciences [ ] Ecological, evolutionary & environmental sciences

For a reference copy of the document with all sections, see nature.com/documents/nr-reporting-summary-flat.pdf

Life sciences study design

All studies must disclose on these points even when the disclosure is negative.

Sample size	No sample size calculation was performed. Experiments were performed in triplicates (n=3) or greater.
Data exclusions	No data were excluded.
Replication	All experiments were performed in duplicates or triplicates and all attempts at replication were successful (size indicated in Figure legend).
Randomization	Aspergillus niger colonies were chosen at random from plates and no data was excluded.
Blinding	The study does not contain experiments where blinding would be applicable.

Reporting for specific materials, systems and methods

We require information from authors about some types of materials, experimental systems and methods used in many studies. Here, indicate whether each material, system or method listed is relevant to your study. If you are not sure if a list item applies to your research, read the appropriate section before selecting a response.

Materials & experimental systems

n/a	Involved in the study	
[x]	[ ]	Antibodies
[x]	[ ]	Eukaryotic cell lines
[x]	[ ]	Palaeontology and archaeology
[x]	[ ]	Animals and other organisms
[x]	[ ]	Clinical data
[x]	[ ]	Dual use research of concern

Methods

n/a	Involved in the study	
[x]	[ ]	ChIP-seq
[x]	[ ]	Flow cytometry
[x]	[ ]	MRI-based neuroimaging
